# SulfoxFluor-enabled deoxyazidation of alcohols with NaN_3_

**DOI:** 10.1038/s41467-022-30132-x

**Published:** 2022-05-18

**Authors:** Junkai Guo, Xiu Wang, Chuanfa Ni, Xiaolong Wan, Jinbo Hu

**Affiliations:** Key Laboratory of Organofluorine Chemistry, Center for Excellence in Molecular Synthesis, Shanghai Institute of Organic Chemistry, University of Chinese Academy of Sciences, Chinese Academy of Sciences, 345 Ling-Ling Road, Shanghai, 200032 China

**Keywords:** Synthetic chemistry methodology, Chemical synthesis

## Abstract

Direct deoxyazidation of alcohols with NaN_3_ is a straightforward method for the synthesis of widely used alkyl azides in organic chemistry. However, known methods have some limitations such as high reaction temperatures and narrow substrate scope. Herein, a general and practical method for the preparation of alkyl azides from alcohols using NaN_3_ has been developed. *N*-tosyl-4-chlorobenzenesulfonimidoyl fluoride (SulfoxFluor) plays an important role in this deoxyazidation process, which converts a broad range of alcohols into alkyl azides at room temperature. The power of this deoxyazidation protocol has been demonstrated by successful late-stage deoxyazidation of natural products and pharmaceutically relevant molecules.

## Abstract (web summary)

Azides are common moieties in chemical synthesis. Here the authors present a methodology to synthesize alkyl azides in one step from simple alcohols under mild conditions.

## Introduction

**Fig. 1:**
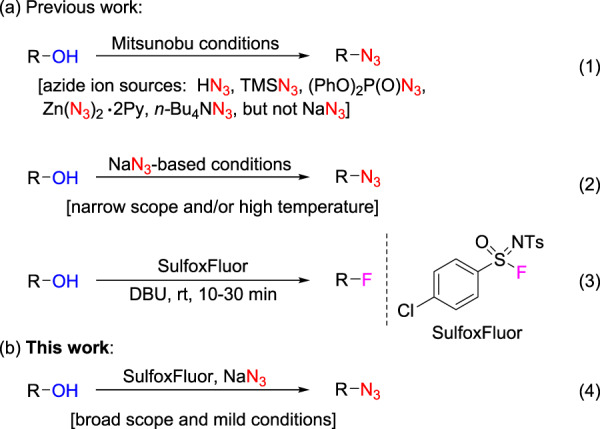
Deoxyazidation of alcohols. **a** Illustration of previous work on deoxyazidation of alcohols (Eqs 1–2) and deoxyfluorination of alcohols with SulfoxFluor (Eq 3). **b** Illustration of this work. Eq 4 refers to the SulfoxFluor-mediated deoxyazidation of alcohols with NaN_3_. Eqs 1–3 refer to the previously reported deoxyazidation of alcohols (previous work), and eq 4 shows the SulfoxFluor-mediated deoxyazidation of alcohols with NaN_3_ (this work).

Organic azides are a class of important compounds that have been used as precursors for nitrenes and in the synthesis of amines, and more popularly in the copper-catalyzed azide-alkyne cycloadditions (known as click chemistry)^1–10^. Alkyl azides are typically prepared by nucleophilic substitution (S_N_2) with an azide ion (N_3_^−^), and the diazo-transfer reaction to primary amines using triflyl azide (CF_3_SO_2_N_3_) or fluorosulfuryl azide (FSO_2_N_3_) has emerged as a powerful method for the preparation of alkyl azides from primary amines^1,11^. On the other hand, given the ready availability of structurally diverse alcohols, direct conversion of alcohols to alkyl azides via deoxyazidation would be an attractive synthetic strategy, which avoids the use of genotoxic alkyl halides and sulfonates in azidation reactions^12^. Unfortunately, the alcoholic hydroxyl group is a poor leaving group, so its direct displacement by azide ion is generally difficult. Previously, Mitsunobu conditions have been investigated by using different azide ion sources such as HN_3_, TMSN_3_, (PhO)_2_P(O)N_3_, Zn(N_3_)_2_•2Py, or *n*-Bu_4_NN_3_, but the Mitsunobu conditions are not amenable to the most readily available and cost-effective azide source—NaN_3_ (Fig. 1, Eq 1)^13–26^. Indeed, the currently known NaN_3_-based deoxyazidation methods are sparse (Fig. 1, Eq 2). Both NaN_3_/BF_3_•Et_2_O^27^ and NaN_3_/triphosgene^28^ systems are only applicable to allylic and benzylic alcohols, and other NaN_3_-based methods (using NaN_3_/TsIm^29^, (2,4-Cl_2_C_6_H_3_O)_2_P(O)Cl/NaN_3_^30^, or halocarbon/Ph_3_P/NaN_3_^31–34^) suffer from the high reaction temperatures and/or narrow substrate scope. Therefore, the development of a general method for efficient and direct conversion of alcohols to alkyl azides with NaN_3_ is highly desirable.

Sulfonimidoyl compounds possess diverse reactivity (compared with sulfonyl compounds) due to the modulation by the nitrogen substituent^35–37^. During the past decade, our group has been interested in developing fluoroalkyl sulfoximines as versatile fluoroalkylation reagents^38,39^. Recently, we reported that *N*-tosyl-4-chlorobenzenesulfonimidoyl fluoride (SulfoxFluor) can serve as a deoxyfluorination reagent for converting alcohols to alkyl fluorides (Fig. 1, Eq 3)^40^. In this fluorination process, the in situ formed alkyl sulfonimidate (from SulfoxFluor and alcohol) serves as the real electrophilic alkylating agent to react with hydrogen-bonded fluoride ion, affording the desired alkyl fluoride^40,41^. We envisioned that since fluoride ion is a weak nucleophile^42^, if there is a strong nucleophile (namely, azide ion) existing in the reaction system, the deoxyazidation of alcohol could become the dominating reaction pathway, giving an alkyl azide as a major product.

Herein, we show a general and practical protocol for deoxyazidation of readily available alcohols with NaN_3_ using SulfoxFluor as an activator (Fig. 1, Eq 4). A wide range of alkyl azides could be obtained successfully under mild reaction conditions.

## Results

### Optimization of reaction conditions

At the onset of our investigation, we chose the primary alcohol **2a** as a model substrate, NaN_3_ as an azidation agent, SulfoxFluor as an activator, 1,8-diazabicyclo-[5.4.0]undec-7-ene (DBU) as a base^40,43–46^, and DMF as a solvent; and the reaction was carried out at room temperature. The preliminary result showed that the use of SulfoxFluor (1.0 equiv) afforded azide** 3a** in 59% yield and a majority of **2a** (31%) remained (Table 1, entry 1). Further optimization of the equiv of SulfoxFluor, NaN_3_, and DBU showed that azide **3a** was formed in 84% yield without fluorination and elimination by-products (Table 1, entry 4). Reducing the equiv of NaN_3_ resulted in the formation of alkyl fluoride **4** (Table 1, entries 5–6). No azide **3a** was formed when triethylamine and pyridine were used as bases (Table 1, entries 7–8). For secondary alcohol **2b**, it was found that the use of 2.2 equiv of SulfoxFluor was not enough, and the desired alkyl azide **3b** was formed in only 38% yield, along with a significant amount of **2b** (44%) remained (Table 2, entry 1). Changing the amounts of both SulfoxFluor (2.8 equiv) and DBU (4.0 equiv) resulted in a higher yield (65%) of **3b** (Table 2, entries 2–4). Further screening of the reaction conditions showed that an 84% yield of **3b** could be obtained in 12 h by performing the reaction with **2b** (1.0 equiv), NaN_3_ (2.0 equiv), SulfoxFluor (2.8 equiv), and DBU (4.0 equiv) at room temperature; and remarkably, alcohol **2b** was completely consumed and no fluorination and elimination side products were formed (Table 2, entry 5). Notably, the use of perfluorobutanesulfonyl fluoride (instead of SulfoxFluor) resulted in a decrease of the yield of **3b** (68%), with 7% of elimination side product **7** being formed (Table 2, entry 6)^45–47^. Shortening the reaction time to 6 h or using other solvents (such as DMSO, toluene, and CH_3_CN) did not give better yields of product **3b** (Table 2, entries 7–10).

**Table 1:** Screening of reaction conditions for primary alcohol 2a.


Entry^a^	2a/SulfoxFluor/NaN_3_/Base	Base	2a (%)^b^	3a (%)^b^	4 (%)^b^	5 (%)^b^
1	1.0: 1.0: 1.0: 1.0	DBU	31	59	2	0
2	1.0: 1.3: 4.0: 1.8	DBU	17	66	trace	0
3	1.0: 1.8: 4.0: 1.8	DBU	8	79	0	0
4	1.0: 2.2: 4.0: 1.8	DBU	trace	84	0	0
5	1.0: 2.2: 3.0: 1.8	DBU	trace	75	8	0
6	1.0: 2.2: 2.0: 1.8	DBU	trace	69	10	0
7	1.0: 2.2: 4.0: 1.8	NEt_3_	92	0	0	0
8	1.0: 2.2: 4.0: 1.8	pyridine	90	0	0	0

^a^Reactions were conducted on a 0.1 mmol scale.

^b^Yields were determined by ^19^F NMR using 1-fluoronaphthalene as an internal standard.

**Table 2:** Screening of reaction conditions for secondary alcohol 2b.


Entry^a^	2b/SulfoxFluor/NaN_3_/DBU	Solvent	2b (%)^b^	3b (%)^b^	6 (%)^b^	7 (%)^b^
1	1.0: 2.2: 4.0: 1.8	DMF	44	38	0	0
2	1.0: 1.3: 4.0: 1.8	DMF	59	27	0	0
3	1.0: 2.2: 4.0: 4.0	DMF	25	50	0	0
4	1.0: 2.8: 4.0: 4.0	DMF	17	65	0	0
5	1.0: 2.8: 2.0: 4.0	DMF	0	84	0	0
6^c^	1.0: 2.8: 2.0: 4.0	DMF	0	68	0	7
7^d^	1.0: 2.8: 2.0: 4.0	DMF	0	82	0	0
8	1.0: 2.8: 2.0: 4.0	DMSO	0	81	0	0
9	1.0: 2.8: 2.0: 4.0	toluene	2	8	42	0
10	1.0: 2.8: 2.0: 4.0	CH_3_CN	2	17	21	0

^a^Reactions were conducted on a 0.1-mmol scale.

^b^Yields were determined by ^19^F NMR using 1-fluoronaphthalene as an internal standard.

^c^Perfluorobutanesulfonyl fluoride (PBSF) was used instead of SulfoxFluor.

^d^The reaction time was 6 h.

### Comparison of various sulfonyl fluorides and sulfonimidoyl fluorides in deoxyazidation of alcohols

To demonstrate the uniqueness of our reagent in the deoxyazidation reaction, several sulfonyl fluorides and sulfonimidoyl fluorides were compared to show their reactivity. 2,2,2-Trifluoroethanol (**2c**) was chosen as a model substrate to react with these reagents under standard conditions. The results are shown in Table 3. An excellent yield of azide **3c** (93%) was formed by using SulfoxFluor as an activator, along with a small amount of **2c** (4%) remained (Table 3, entry 1). Changing the *S*-substituent to an electron-neutral or more electron-deficient 4-nitrophenyl group resulted in a decrease in the yield of **3c** (Table 3, entries 2 and 3). Moreover, in the case of **1d** with an *N*-alkyl substituent, no azide **3c** was formed and nearly half of **2c** was converted to the sulfonimidoyl ester intermediate** 8d** (Table 3, entry 4). When 2-pyridylsulfonyl fluoride (PyFluor) **1e** and tosyl fluoride **1f** were used, a full conversion to the corresponding sulfonyl ester intermediates was observed (Table 3, entries 5 and 6). Replacing the *N*-substituent from tosyl to tertiary butyl led to no azide formation, and a recovery of **2c** (82%) was observed (Table 3, entry 7). In the case of perfluorobutanesulfonyl fluoride (PBSF), a lower yield (82%) of **3c** was obtained (Table 3, entry 8); however, PBSF was found to give an elimination by-product as mentioned before (Table 2, entry 6). Finally, when SO_2_F_2_ was used under similar conditions, a low yield (12%) of azide **3c** was formed (Table 3, entry 9). Clearly, SulfoxFluor was superior to other sulfonimidoyl fluorides and sulfonyl fluorides in the present deoxyazidation reaction. It is interesting to note that the use of bis(2,4-dichlorophenyl) chlorophosphate ((2,4-Cl_2_C_6_H_3_O)_2_P(O)Cl, **1j**)/NaN_3_/DMAP, a state-of-the-art method for deoxyazidation of alcohols at room temperature^30^, failed to convert 2,2,2-trifluoroethanol (**2c**) into azide **3c** (Table 3, entry 10; for details, see the Supplementary Methods).

**Table 3:** Azidation of CF_3_CH_2_OH with sulfonyl fluorides and sulfonimidoyl fluorides.


Entry^a^	1	2c (%)^*b*^	8 (%)^b^	3c (%)^b^
1	**1a**	4	0	93
2	**1b**	4	12	73
3	**1c**	16	trace	67
4	**1d**	53	41	0
5	**1e**	0	99^c^	trace
6	**1f**	0	>99^c^	0
7	**1g**	82	trace	0
8	**1h**	2	0	82
9^d^	**1i**	0	0	12
10^e^	**1j**	8	-^f^	0

*DMAP* 4-dimethylaminopyridine.

^a^Reactions were conducted on 0.1 mmol under an N_2_ atmosphere.

^b^Yields were determined by ^19^F NMR using PhCF_3_ as the internal standard.

^c^The existence of 8e and 8f was proved by GC-MS.

^d^SO_2_F_2_ was dissolved in DMF at a concentration of 0.0616 M.

^e^Conditions: **2c** (0.2 mmol), **1j** (1.05 equiv), NaN_3_ (4.0 equiv), DMAP (1.2 equiv), DMF (0.2 M), rt, 12 h.

^f^Unidentified products.

### Deoxyazidation of alcohols

**Fig. 2:**
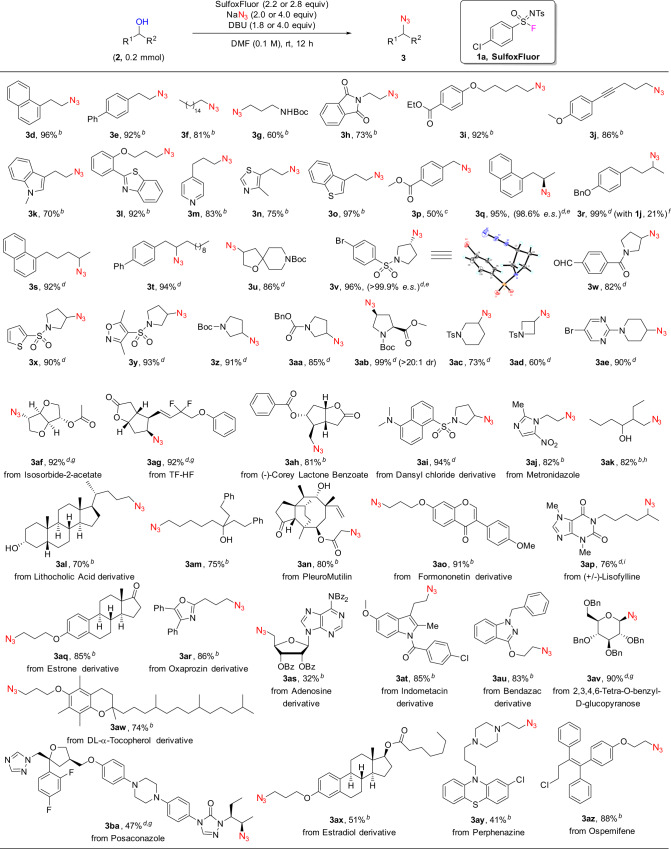
Azidation of alcohols using SulfoxFluor^a^. ^a^Isolated yields. ^b^For primary alcohols: reactions were conducted on 0.2 mmol scale using 2.2 equiv of SulfoxFluor, 4.0 equiv of NaN_3_ and 1.8 equiv of DBU. ^c^Reaction was conducted on 0.2 mmol scale using 2.5 equiv of SulfoxFluor, 5.0 equiv of NaN_3_, and 1.8 equiv of DBU. ^d^For secondary alcohols: reactions were conducted on 0.2 mmol scale using 2.8 equiv of SulfoxFluor, 2.0 equiv of NaN_3_, and 4.0 equiv of DBU. ^e^The abbreviation e.s. refers to enantiospecifity, e.s. = (e.e. of **3**)/(e.e. of **2**) × 100%. ^f^Reaction was conducted on a 0.2 mmol scale using 1.05 equiv of **1j**, 4.0 equiv of NaN_3_, and 1.2 equiv of DMAP in DMF (0.2 M) at rt for 12 h. ^g^Epimer ratio >20:1. ^h^Reaction was conducted on a 0.4 mmol scale. ^i^Reaction was conducted on a 0.16 mmol scale.

With the optimized conditions (Table 1, entry 4 for primary alcohols; Table 2, entry 5 for secondary alcohols) in hand, we investigated the substrate scope of this SulfoxFluor-mediated deoxyazidation reaction using NaN_3_ as an azide source. The results are shown in Fig. 2. Fifty structurally diverse primary and secondary alcohols were applied in this reaction, nearly half of which are pharmaceutically important molecules. In most cases, the corresponding alkyl azides were obtained in good or excellent yields. The reaction tolerates a variety of functional groups, such as aldehydes, alkenes, alkynes, ketones, esters, amides, halides, nitro, and sulfonyl groups (see Fig. 2). It has been known that aldehydes are not amenable to Mitsunobu reactions owing to the condensation of the aldehyde functionality with Huisgen zwitterions^48^; however, it is remarkable that under our current azidation reaction conditions, desired product **3w** was obtained in 82% yield. Our reaction is also compatible with the majority of heterocycles; heteroaromatic substrates such as indole, benzothiazole, pyridine, thiazole, thiophene, benzothiophene, and pyrimidine are all suitable substrates for this reaction (see **3k**–**3o**, **3x**, **3y**, and **3ae**). The reaction of enantiomerically enriched secondary alcohols **2q** and **2v** proceeded smoothly to give products **3q** and **3v** in excellent yields (95% and 96%) and stereospecificity (98.6% and >99.9% e.s.) respectively, which is in accordance with an inversion of configuration resulting from an S_N_2 mechanism (CCDC 2005774 (**3v**) contains the supplementary crystallographic data for this paper. These data are provided free of charge by The Cambridge Crystallographic Data Centre.)^40,42^. Similarly, the stereogenic centers of **3ab**, **3af**, **3ag**, **3av**, and **3ba** were assigned by analogy. Cyclic alcohols such as four-, five-, and six-membered rings could also undergo efficient deoxyazidation under the standard conditions, giving corresponding products in good to excellent yields (**3u**–**3ae**). Notably, the carbamate group in primary alcohol **2g** is also compatible without elimination and intramolecular cyclization by-products under the present reaction conditions, giving azide **3g** in 60% yield.

Late-stage modification of structurally complex molecules (such as natural products and drugs) can rapidly generate new pharmaceutical candidates with potentially improved pharmacological profiles^49–58^. Late-stage azidation is particularly attractive in this regard because the incorporation of azide functionality (followed by click reaction) can quickly build modular molecular libraries. Inspired by these considerations, we applied our deoxyazidation method in the late-stage functionalization of complex molecules such as isosorbide-2-acetate (**2af**), TF-HF (**2ag**) and (-)-Corey lactone benzoate (**2ah**), and the corresponding azidation products **3af**, **3ag**, and **3ah** were obtained in 92%, 92%, and 81% yields, respectively (Fig. 2). The dansyl chloride derivative **2ai** was also efficiently deoxyazidated to give product **3ai** in 94% yield, indicating that tertiary amine functionality is compatible with the current azidation conditions. It is interesting to mention that, the current deoxyazidation reaction has good selectivity toward multiple alcohols (such as **2ak**-**2an**), that is, the azidation occurs predominantly on the less hindered hydroxyl group and affords the mono-azidation products in good yields (see **3ak**-**3an**). The drug derivatives estrone (**2aq)**, oxaprozin (**2ar)**, adenosine (**2as)**, indometacin (**2at)**, bendazac (**2au)**, DL-α-tocopherol (**2aw)**, estradiol (**2ax)**, and glucose derivative **2av** were all able to undergo deoxyazidation smoothly to afford the corresponding products **3aq**-**3ax** in moderate to good yields. Most remarkably, when antifungal drug posaconazole (**2ba)**, the most complex molecule among the fifty substrates shown in Fig. 2, was subjected to the current deoxyazidation process, product **3ba** was isolated in satisfactory yield (47%).

Of note that our synthetic method is also advantageous over the previously reported deoxyazidation system (2,4-Cl_2_C_6_H_3_O)_2_P(O)Cl/NaN_3_/DMAP in converting normal secondary alcohols to organoazides. For example, starting from alcohol **2r**, the use of SulfoxFluor could afford the corresponding azide **3r** in nearly quantitative yield, whereas the use of (2,4-Cl_2_C_6_H_3_O)_2_P(O)Cl under reported standard conditions^30^ provided **3r** in low yield (21%) even prolonging the reaction time to 12 h. In the latter case, both the bis(2,4-dichlorophenyl) phosphate intermediate and unreacted alcohol **2r** were isolated (for detail, see the Supplementary Methods), indicating the low efficiency of (2,4-Cl_2_C_6_H_3_O)_2_P(O)Cl in the activation of normal secondary alcohols.

### Synthetic applications

**Fig. 3:**
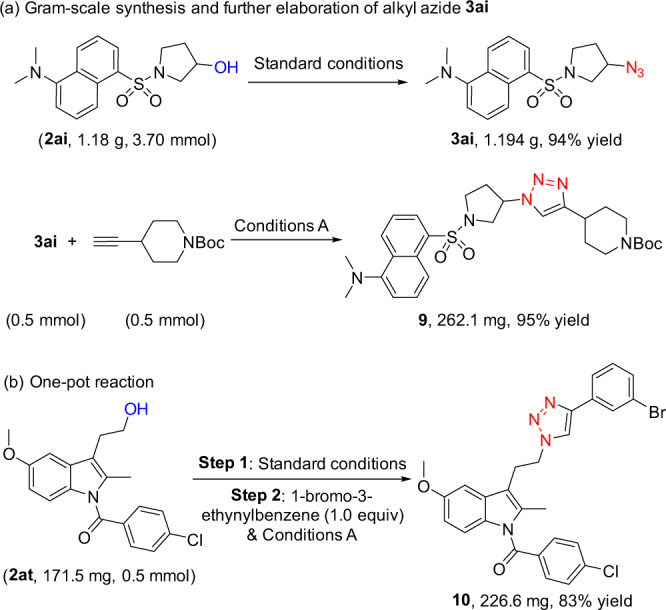
Synthetic applications. **a** Gram-scale synthesis of alkyl azide 3ai and its further elaboration via Click reaction. **b** One-pot deoxyazidation and subsequent Click reaction. Conditions A: CuSO_4_•5H_2_O (1 mol%), sodium ascorbate (10 mol%), *t*BuOH/H_2_O = 1:1, rt, 24 h.

To further demonstrate the synthetic utility of the current deoxyazidation protocol, we carried out the gram-scale synthesis. As shown in Fig. 3a, the deoxyazidation reaction of dansyl chloride derivative **2ai** was carried out under standard conditions. This reaction proceeded well and afforded the desired azide **3ai** in 94% yield. Remarkably, the azide product **3ai** could be converted to triazole **9** under copper catalysis^59^ in nearly quantitative yield, which significantly increased the complexity of the molecule and demonstrated the potential application of this azidation protocol in drug discovery. Furthermore, indometacin derivative **2at** was subjected to the deoxyazidation reaction and subsequent click reaction in one pot, and triazole **10** was obtained in 83% overall yield (Fig. 3b; for details, see the Supplementary Methods).

### Experimental investigation of a reaction mechanism

**Fig. 4:**
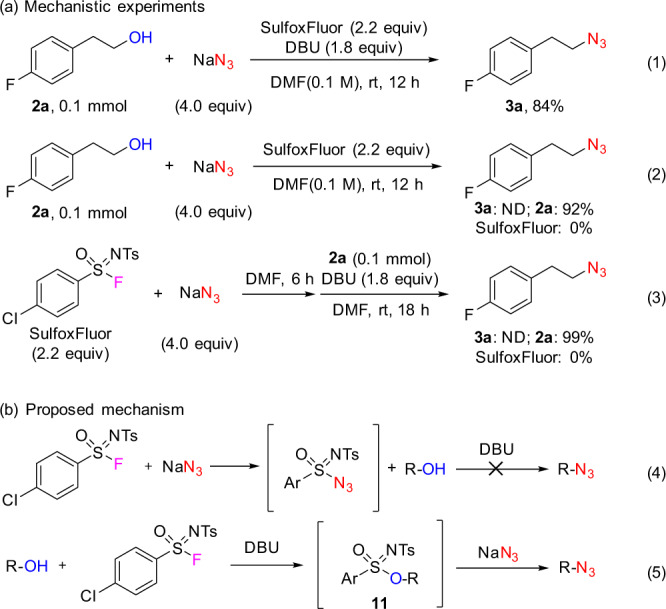
Mechanistic experiments. **a** The comparison of the standard experiment and the control experiments. Eq 1 refers to the deoxyazidation reaction conducted under the standard conditions. Eq 2 refers to the control experiment performed in the absence of DBU. Eq 3 refers to the control experiment performed via reverse addition of the reactants. **b** Proposed mechanism of competitive consumption of SulfoxFluor by NaN_3_ (Eq 4) and the desired deoxyazidation of alcohols (Eq 5). ND not detected.

Control experiments were performed to investigate the reaction mechanism (Fig. 4). Alcohol **2a** reacted under standard conditions to give azide **3a** in 84% yield (determined by ^19^F NMR; Fig. 4, Eq 1). However, when DBU was not added, the expected product **3a** was not detected and the alcohol **2a** remained, along with the complete consumption of SulfoxFluor (Fig. 4, Eq 2). This result indicates that SulfoxFluor itself could react with NaN_3_. Further experiments showed the pre-formed sulfonimidoyl azide intermediate was not able to undergo the desired deoxyazidation reaction (Fig. 4, Eqs 3 and 4). Based on the above-mentioned experiments, a plausible reaction mechanism is proposed for the deoxyazidation of alcohols with SulfoxFluor (Fig. 4, Eq 5). The activated alcohol (by DBU) reacts with SulfoxFluor to form sulfonimidate ester **11**, which undergoes a nucleophilic displacement of the sulfonimidate group by azide ion to give the corresponding alkyl azide. The success of the azidation reaction lies in better nucleophilicity of azide ion over that of fluoride ion.

## Discussion

We have developed a general protocol for the direct deoxyazidation of alcohols with NaN_3_, which provides a powerful tool to synthesize structurally diverse alkyl azides from readily available alcohols under mild conditions. Our previously developed SulfoxFluor reagent^60,61^ plays an important role in this efficient deoxyazidation reaction. To our knowledge, the substrate scope and functional group tolerance of this method are superior to those of other deoxyazidation reactions (starting from alcohols) reported to date. Moreover, we have shown that this method can be applied in the late-stage modification of natural products and pharmaceutically relevant molecules, showcasing that this protocol promises to find practical applications in life sciences and related fields. Further exploration in this direction is underway in our laboratory.

## Methods

### General

The general procedures for deoxyazidation of primary alcohols **2** with SulfoxFluor **1a** are as follows. In a typical experiment, into a 25-mL Schlenk tube (glass) were sequentially added 2-(naphthalen-1-yl)ethan-1-ol** 2c** (34.4 mg, 0.2 mmol), SulfoxFluor (152.9 mg, 0.44 mmol, 2.2 equiv), NaN_3_ (52.0 mg, 0.8 mmol), DMF (2.0 mL), and DBU (54 μL, 0.36 mmol, 1.8 equiv) under N_2_ atmosphere. The mixture was stirred at room temperature for 12 h. Then water (2–5 mL) was added and the mixture was extracted with Et_2_O (3 × 2 mL). The combined organic layers were dried over Na_2_SO_4_, filtered, concentrated under reduced pressure, and purified by chromatography on silica gel to afford alkyl azide **3c** (37.7 mg, 96%). The deoxyazidation of secondary alcohols **2** with SulfoxFluor **1a** were carried out similarly and the procedures are presented in Supplementary Methods.

## Supplementary information

Supplementary Information

Peer Review File

## Data Availability

The authors declare that the data supporting the findings of this study are available within the article and its Supplementary Information files. For the experimental procedures, and spectroscopic and physical data of compounds, see Supplementary Methods. For NMR analysis of the compounds in this article, see Supplementary Figs. 1–134. The CCDC 2005774 [10.5517/ccdc.csd.cc25b5d3] contains the crystallographic data for compound **3v** (Supplementary Fig. 139; Supplementary Table 6). These data can be obtained free of charge from the Cambridge Crystallographic Data Center (www.ccdc.cam.ac.uk).

## References

[1] Bräse, S. & Banert, K. *Organic Azides: Syntheses and Applications* (Wiley, 2010).

[2] Patai, S. *The Chemistry of the Azide Group* (Interscience, 1971).

[3] Bayley H, Knowles JR (1977). Photoaffinity labeling. Methods Enzymol..

[4] Bayley, H. *Photogenerated Reagents in Biochemistry and Molecular Biology* (Elsevier, 1983).

[5] Fedan JS, Hogaboom GK, O’Donnell JP (1984). Photoafflnity labeling as pharmacological tools. Biochem. Pharmacol..

[6] Radominska A, Rdrake R (1994). Synthesis and uses of azido-substituted nucleoside diphosphate sugar photoaffinity analogs. Methods Enzymol..

[7] Singh A, Thornton ER, Westheimer FH (1962). Photolysis of diazoacetylchymotrypsin. J. Biol. Chem..

[8] Kolb HC, Finn M, Sharpless GKB (2001). Click chemistry: diverse chemical function from a few good reactions. Angew. Chem. Int. Ed..

[9] Bock VD, Hiemstra H, van Maarseveen JH (2006). CuI-Catalyzed alkyne–azide “click” cycloadditions from a mechanistic and synthetic perspective. Eur. J. Org. Chem..

[10] Becer CR, Hoogenboom R, Schubert US (2009). Click chemistry beyond metal-catalyzed cycloaddition. Angew. Chem. Int. Ed..

[11] Meng G (2019). Modular click chemistry libraries for functional screens using a diazotizing reagent. Nature.

[12] Bryan MC (2018). Key green chemistry research areas from a pharmaceutical manufacturers’ perspective revisited. Green. Chem..

[13] Mitsunobu O, Wada M, Sano T (1972). Stereospecific and stereoselective reactions. I. Preparation of amines from alcohols. J. Am. Chem. Soc..

[14] Hughes DL (1992). The Mitsunobu reaction. Org. React..

[15] Loibner H, Zbiral E (1977). New preparative methods using triphenylphosphine-diethyl-azodicarboxylate-hydroxycompound. Helv. Chim. Acta.

[16] Mitsunobu, O. The use of diethyl azodicarboxylate and triphenylphosphine in synthesis and transformation of natural products. *Synthesis* **1981**, 1–28 (1981).

[17] Hughes DL (1996). Progress in Mitsunobu reaction: a review. Org. Prep. Proced. Int..

[18] Saito A, Saito K, Tanaka A, Oritani T (1997). An efficient method for converting alcohols to azides with 2,4,4,6-tetrabromo-2,5-cyclohexadienone/PPh_3_/Zn(N_3_)_2_·2Py. Tetrahedron Lett..

[19] Mizuno, M. & Shior, T. Efficient method for the one-pot azidation of alcohols using bis(p-nitrophenyl) phosphorazidate. *Chem. Commun*. **22**, 2165–2166 (1997).

[20] Fabiano, E., Golding, B. T. & Sadeghi, M. M. A simple conversion of alcohols into amines. *Synthesis* **1987**, 190–192 (1987).

[21] Bessodes M, Abushanab E, Antonakis K (1984). Enantiospecific synthesis of the immunopotentiators erythro-9 (2-hydroxy-3-nonyl) hypoxanthines and the threo-diastereomers. Tetrahedron Lett..

[22] Lee S-H, Yoon J, Chung S-H, Lee Y-S (2001). Efficient asymmetric synthesis of 2,3-diamino-3-phenylpropanoicacid derivatives. Tetrahedron.

[23] He L, Wanunu M, Byun H-S, Bittman R (1999). Regioselective and stereospecific azidation of 1,2- and 1,3-diols by azidotrimethylsilane via a Mitsunobu reaction. J. Org. Chem..

[24] Lal B, Pramanik BN, Manhas MS, Bose AK (1977). Diphenylphosphoryl azide a novel reagent for the stereospecific synthesis of azides from alcohols. Tetrahedron Lett..

[25] Viaud, M. C. & Rollin, P. Zinc azide mediated Mitsunobu substitution. An expedient method for the one-pot azidation of alcohols *Synthesis* **1990**, 130-132 (1990).

[26] Iranpoor N, Firouzabadi H, Akhlaghinia B, Nowrouzi N (2004). A novel and highly selective conversion of alcohols, thiols, and silyl ethers to azides using the triphenylphosphine/2,3-dichloro-5,6-dicyanobenzoquinone(DDQ)/*n*-Bu_4_NN_3_ system. Tetrahedron Lett..

[27] Kumar HMS, Reddy BVS, Anjaneyulu S, Yadav JS (1998). An expedient and highly selective conversion of alcohols to azides using a NaN_3_/BF_3_•Et_2_O System. Tetrahedron Lett..

[28] Jayanthi, A., Gumaste, V. K. & Deshmukh, A. R. A. S. A simple one-pot method for the preparation of allyl azides from allyl alcohols using triphosgene: synthesis of *N*1-cinnamyl azetidin-2-ones. *Synlett* **2004**, 979–982 (2004).

[29] Rad MNS, Behrouz S, Khalafi-Nezhad A (2007). A simple one-pot procedure for the direct conversion of alcohols into azides using TsIm. Tetrahedron Lett..

[30] Yu C, Liu B, Hu L (2000). A simple one-pot procedure for the direct conversion of alcohols to azides via phosphate activation. Org. Lett..

[31] Li Z, Qiao R, Yang Z, Zhang L, Zhang L (2006). One-step synthesis of novel tricyclic isomeric azidonucleosides. Tetrahedron. Asymmetry.

[32] Toyota M, Komori C, Ihara M (2000). A concise formal total synthesis of mappicine and nothapodytine B via an intramolecular hetero Diels−Alder reaction. J. Org. Chem..

[33] Reddy GVS, Rao GV, Subramanyam RVK, Iyengar DS (2000). A new novel and practical one pot methodology for conversion of alcohols to amines. Synth. Commun..

[34] Chen J, Lin J, Xiao J (2018). Dehydroxylation of alcohols for nucleophilic substitution. Chem. Commun..

[35] Reggelin, M. & Zur, C. Sulfoximines: structures, properties and synthetic applications. *Synthesis* **2000**, 1–64 (2000).

[36] Lücking U (2013). Sulfoximines: a neglected opportunity in medicinal chemistry. Angew. Chem. Int. Ed..

[37] Bizet V, Kowalczyk R, Bolm C (2014). Fluorinated sulfoximines: syntheses, properties and applications. Chem. Soc. Rev..

[38] Shen X, Hu J (2014). Fluorinated sulfoximines: preparation, reactions and applications. Eur. J. Org. Chem..

[39] Liu Q, Shen X, Ni C, Hu J (2017). Stereoselective carbonyl olefination with fluorosulfoximines: facile access to *Z* or *E* terminal monofluoroalkenes. Angew. Chem. Int. Ed..

[40] Guo J (2019). Rapid deoxyfluorination of alcohols with *N*-Tosyl-4-chlorobenzenesulfonimidoyl fluoride (SulfoxFluor) at room temperature. Chem. Eur. J..

[41] Tang H, Cheng J, Liang Y, Wang Y (2020). Discovery of a chiral fluorinated azetidin-2-one as a tubulin polymerisation inhibitor with potent antitumour efficacy. Eur. J. Med. Chem..

[42] Ouellette, R. J. & Rawn, J. D. *Organic Chemistry: Structure, Mechanism, and Synthesis* 2nd edn (Elsevier, 2018).

[43] Nielsen MK, Ugaz CR, Li W, Doyle AG (2015). PyFluor: a low-cost, stable, and selective deoxyfluorination reagent. J. Am. Chem. Soc..

[44] Nielsen MK, Ahneman DT, Riera O, Doyle AG (2018). Deoxyfluorination with sulfonyl fluorides: navigating reaction space with machine learning. J. Am. Chem. Soc..

[45] Bennua-Skalmowski B, Vorbrüggen H (1995). A facile conversion of primary or secondary alcohols with n-perfluorobutane-sulfonyl fluoride/1,8-diazabicyclo[5.4.0]undec-7-ene into their corresponding fluorides. Tetrahedron Lett..

[46] Vorbrüggen, H. The conversion of primary or secondary alcohols with nonaflyl fluoride into their corresponding inverted fluorides. *Synthesis* **2008**, 1165–1174 (2008).

[47] Yin J, Zarkowsky DS, Thomas DW, Zhao MM, Huffman MA (2004). Direct and convenient conversion of alcohols to fluorides. Org. Lett..

[48] Girard M, Murphy P, Tsou NN (2005). An exception to the normal Mitsunobu reaction with phenols; the formation of hydrazones from salicylaldehydes. Tetrahedron Lett..

[49] Long, A. A. W., Nayler, J. H. C., Smith, H., Taylor, T. & Ward, N. Derivatives of 6-aminopenicillanic acid. Part X1. a-amino-p-hydroxy-benzylpenicillin. *J. Chem. Soc. C* 1920–1922 (1971). 5103806

[50] Lewis CA, Miller SJ (2006). Site-selective derivatization and remodeling of erythromycin A by using simple peptide-based chiral catalysts. Angew. Chem. Int. Ed..

[51] Newman DJ, Cragg GM (2007). Natural products as sources of new drugs over the last 25 years. J. Nat. Prod..

[52] Butler MS (2008). Natural products to drugs: natural product-derived compounds in clinical trials. Nat. Prod. Rep..

[53] Reed SA, Mazzotti AR, White MC (2009). A catalytic, Brønsted base strategy for intermolecular allylic C−H amination. J. Am. Chem. Soc..

[54] Balthaser BR, Maloney MC, Beeler AB, Porco JA, Snyder JK (2011). Remodelling of the natural product fumagillol employing a reaction discovery approach. Nat. Chem..

[55] Jordan PA, Miller SJ (2012). An approach to the site-selective deoxygenation of hydroxy groups based on catalytic phosphoramidite transfer. Angew. Chem. Int. Ed..

[56] Pathak TP, Miller SJ (2012). Site-selective bromination of vancomycin. J. Am. Chem. Soc..

[57] Fowler BS, Laemmerhold KM, Miller SJ (2012). Catalytic site-selective thiocarbonylations and deoxygenations of vancomycin reveal hydroxyl-dependent conformational effects. J. Am. Chem. Soc..

[58] Voica A-F, Mendoza A, Gutekunst WR, Fraga JO, Baran PS (2012). Guided desaturation of unactivated aliphatics. Nat. Chem..

[59] Rostovtsev VV, Green LG, Fokin VV, Sharpless KB (2002). A stepwise Huisgen cycloaddition process: copper(I)-catalyzed regioselective “ligation” of azides and terminal alkynes. Angew. Chem. Int. Ed..

[60] Liu R (2022). Modified and scalable synthesis of *N*-tosyl-4-chlorobenzenesulfonimidoyl fluoride (SulfoxFluor): direct imidation of sulfinyl chlorides with chloramine-T trihydrate. Org. Process Res. Dev..

[61] Zhou, X. et al. Method for preparing a deoxyfluorination reagent. Chinese patent application. CN 113717087A (2020).

