# Supplementary material for: SulfoxFluor-enabled deoxyazidation of alcohols with NaN3
Source: Nat Commun. 2022 May 18;13:2752. doi: 10.1038/s41467-022-30132-x (PMC9117260; doi:10.1038/s41467-022-30132-x)
Supplement: Supplementary file 1 — Supplementary Information [file 41467_2022_30132_MOESM1_ESM.pdf]

Supplementary Information

SulfoxFluor-Enabled Deoxyazidation of Alcohols with NaN_3

Junkai Guo[‡], Xiu Wang[‡], Chuanfa Ni, Xiaolong Wan and Jinbo Hu*

Key Laboratory of Organofluorine Chemistry, Center for Excellence in Molecular Synthesis, Shanghai Institute of Organic Chemistry, University of Chinese Academy of Sciences, Chinese Academy of Sciences, 345 Ling-Ling Road, Shanghai 200032, China. *e-mail: jinbohu@sioc.ac.cn

Table of Contents

Supplementary Methods……………………………….…....……….S2

1. General Information……………………..……..…………….......………S2
2. Synthesis of Non-commercial Reagents……………….………………..…S2
3. Deoxyazidation of Alcohols with NaN_3 by SulfoxFluor………………...…S10
4. Synthetic Utility of Deoxyazidation of Alcohols…………………….….......S44
5. Survey of Reaction Conditions ………………………………...….….S46
6. Deoxyazidation of Alcohols with Bis(2,4-dichlorophenyl) chlorophosphate/NaN_3/DMAP: A Comparison………….……..…....…S49
7. Mechanistic Study…………………………………………….………..…S52

Supplementary Figures ………………..…………………...……S54

Supplementary Tables …………………….……………..…...…S124

Supplementary References ……………………………………..…S130

Supplementary Methods

1. General Information

Unless otherwise mentioned, solvents and reagents were purchased from commercial sources and used as received. Toluene, acetonitrile (CH_3CN), *N*,*N*-dimethylformamide (DMF), and dimethyl sulfoxide (DMSO) were dispensed from a dry solvent system. ^{1}H, ^{13}C and ^{19}F NMR spectra were recorded on a 500 MHz or 400 MHz NMR spectrometer. ^{1}H NMR chemical shifts were determined relative to internal $(CH_3)_4Si$ (TMS) at δ 0.0 or to the signal of the residual protonated solvent: $CDCl_3$ δ = 7.26 ppm, DMSO-d_6 δ = 2.50 ppm. ^{13}C NMR chemical shifts were determined relative to internal TMS at δ 0.0. ^{19}F NMR chemical shifts were determined relative to $CFCl_3$ at δ 0.0. Data for ^{1}H, ^{13}C and ^{19}F NMR are recorded as follows: chemical shift (δ, ppm), multiplicity (s = singlet, d = doublet, t = triplet, m = multiplet, q = quartet, br = broad). Mass spactra were obtained on a mass spectrometer. High-resolution mass data were recorded on a high-resolution mass spectrometer in the EI or ESI or DART mode.

Safety Cautions! We did NOT encounter any explosion during the handling of sodium azide (NaN_3) and all the organic azides listed in this document (on less than 10-mmol scale). However, for safety concerns, sodium azide (NaN_3) and all organic azide compounds should always be considered as explosive and toxic substances. When handling sodium azide or organic azide compounds, care should be taken to avoid strong mechanical shock or friction, and contact with metal apparatus such as syringe needle and metal spatula should be avoided.

2. Synthesis of Non-commercial Reagents

A list of non-commercial reagents is given in Supplementary Table 1. Alcohols **2d-2h**, **2m**, **2n, 2p, 2u, 2z-2ab, 2ae-2ah, 2aj, 2ak, 2an, 2ap, 2av 2ay-2ba** were commercially available. Alcohols **2j**,[1] **2k**,[2] **2o**,[3] **2q**,[4] **2r**,[5] **2s**,[6] **2v**,[6] **2w**,[6] **2ac**,[7] **2ad**,[8] **2al**,[9] **2am**,[9] **2ar**,[10] **2as**,[11] and **2at**[12] were synthesized according to previous reports. Procedures for the synthesis of alcohols **2i**, **2l**, **2t**, **2x**, **2y**, **2ai**, **2ao**, **2aq**, **2au**, **2aw** and **2ax** are described as follows. SulfoxFluor (CAS No.: 2143892-50-4) is commercially available from Daicel

Chiral Technologies (China) Co., Ltd. It can also be prepared according to reported modified procedures.[13]

Synthesis of Ethyl 4-((5-hydroxypentyl)oxy)benzoate (2i)

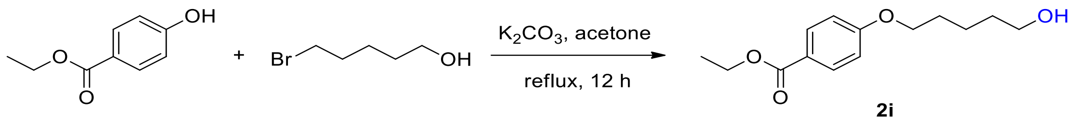

Ethyl 4-((5-hydroxypentyl)oxy)benzoate (**2i**) was prepared according to previous report.[14] Ethyl 4-hydroxybenzoate (1.66 g, 10 mmol, 1.0 equiv) was dissolved in acetone (20 mL) and 5-bromopentan-1-ol (1.38 g, 10 mmol, 1 equiv), potassium carbonate (1.38 g, 10 mmol, 1 equiv) were sequentially added. The reaction mixture was heated at 60 °C for 12 h. After cooling, the solid was filtered off and water was added and the mixture was extracted with EtOAc (2 × 50 mL). The combined organic extracts were dried (Na_2SO_4), concentrated under reduced pressure, and purified by column chromatography on silica gel (petroleum ether/EtOAc 4:1, v/v) to give **2i** as a white solid (1.12 g, 44% yield).

M.p.: 43-44 °C. **IR** (film): 3396, 2922, 2877, 2849, 1710, 1646, 1606, 1579, 1511, 1468, 1423, 1392, 1367, 1314, 1277, 1255, 1168, 1104, 1057, 1023, 846, 771, 698, 644 cm^{-1}. **^{1}H NMR** (400 MHz, $CDCl_3$) δ 7.98 – 7.94 (m, 2H), 6.89 – 6.85 (m, 2H), 4.32 (q, J = 7.1 Hz, 2H), 3.99 (t, J = 6.4 Hz, 2H), 3.66 (t, J = 6.3 Hz, 2H), 1.85 – 1.78 (m, 3H), 1.66 – 1.49 (m, 4H), 1.36 (t, J = 7.1 Hz, 3H). **^{13}C NMR** (100 MHz, $CDCl_3$) δ 166.56, 162.86, 131.59, 122.78, 114.07, 68.06, 62.72, 60.72, 32.45, 28.98, 22.42, 14.46. **HRMS (ESI)**: Calcd. For $C_{14}H_{21}O_4^+$: 253.1434; Found: 253.1436.

Synthesis of 3-(2-(Benzo[*d*]thiazol-2-yl)phenoxy)propan-1-ol (2l)

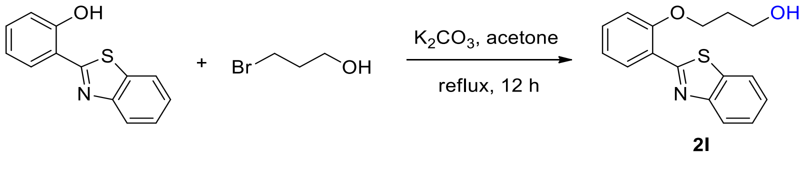

3-(2-(Benzo[d]thiazol-2-yl)phenoxy)propan-1-ol (**2l**) was prepared according to reference.[14] Starting from 2-(benzo[*d*]thiazol-2-yl)phenol (2.27 g, 10.0 mmol), The title

compound **2l** was obtained as as a white solid (1.93 g, 68% yield).

M.p.: 106-108 °C. **IR** (film): 3382, 3063, 2950, 2880, 1598, 1582, 1498, 1452, 1432, 1395, 1320, 1292, 1248, 1218, 1162, 1117, 1054, 1018, 987, 971, 954, 851, 827, 753, 729, 694, 661, 628, 612, 570, 457 cm^{-1}. **^{1}H NMR** (400 MHz, $CDCl_3$) δ 8.36 (dd, J = 7.8, 1.7 Hz, 1H), 8.11 (d, J = 8.2 Hz, 1H), 7.90 (d, J = 7.9 Hz, 1H), 7.51 – 7.35 (m, 3H), 7.13 – 7.09 (m, 1H), 7.05 (d, J = 8.3 Hz, 1H), 4.34 (t, J = 5.9 Hz, 2H), 4.01 (t, J = 5.8 Hz, 2H), 2.75 (s, 1H), 2.24 (p, J = 5.8 Hz, 2H). **^{13}C NMR** (100 MHz, DMSO-d_6) δ 162.21, 156.37, 151.49, 135.43, 132.22, 128.73, 126.12, 124.77, 122.38, 121.65, 121.11, 120.79, 112.89, 66.15, 57.57, 32.12. **HRMS (ESI)**: Calcd. For $C_{16}H_{16}O_2NS^+$: 286.0896; Found: 286.0897.

Synthesis of 1-([1,1'-Biphenyl]-4-yl)dodecan-2-ol (2t)

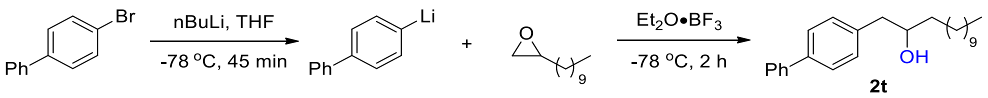

1-([1,1'-Biphenyl]-4-yl)dodecan-2-ol (**2t**) was prepared according to reference[4] with modification. A 2.5 M of BuLi (4.4 mL, 11 mmol, 1.1 equiv) in THF was added slowly to a solution of 4-bromo-1,1'-biphenyl (2.33 g, 10 mmol, 1.0 equiv) in 15 ml of distilled THF at -78 °C with stirring for 45 minutes under N_2. To the solution was added 1,2-epoxydodecane (1.95 g, 10.5 mmol, 1.05 equiv) at this temperature. The mixture was stirred for 2 h at -78 °C, then diluted with water and extracted with ether. The combined organic extracts were dried (Na_2SO_4), concentrated under reduced pressure, and purified by column chromatography on silica gel (petroleum ether/EtOAc 20:1, v/v) to give **2t** as a white solid (1.00 g, 30% yield).

M.p.: 91-92 °C. **IR** (film): 3378, 3326, 2947, 2918, 2849, 1492, 1466, 1432, 1408, 1337, 1130, 1095, 1066, 998, 942, 909, 867, 835, 819, 760, 731, 692 cm^{-1}. **^{1}H NMR** (400 MHz,$CDCl_3$) δ 7.61 – 7.55 (m, 4H), 7.44 (t, J = 7.6 Hz, 2H), 7.36 – 7.26 (m, 3H), 3.91 – 3.82 (m, 1H), 2.79 (ddd, J = 22.0, 13.6, 6.3 Hz, 2H), 1.58 – 1.46 (m, 4H), 1.42 – 1.28 (m, 15H), 0.89 (t, J = 6.8 Hz, 3H). **^{13}C NMR** (100 MHz, $CDCl_3$) δ 140.95, 139.41, 137.80, 129.87, 128.77, 127.28, 127.16, 127.02, 72.72, 43.68, 36.96, 31.94, 29.69, 29.65, 29.37, 25.81, 22.71, 14.14. **MS** (**EI**, m/z, %): 338 (M^+, 4), 168 (100); **HRMS (EI)**: Calcd. For

$C_{24}H_{34}O^+$: 338.2604; Found: 338.2604.

Synthesis of 1-(Thiophen-2-ylsulfonyl)pyrrolidin-3-ol (2x)

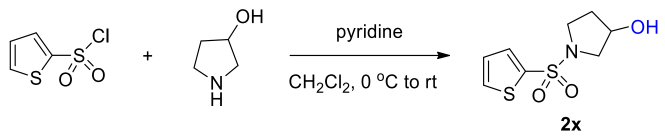

1-(Thiophen-2-ylsulfonyl)pyrrolidin-3-ol (**2x**) was prepared according to reference.[6] Pyrrolidin-3-ol (871.2 mg, 10.0 mmol, 1.0 equiv.) was dissolved in CH_2Cl_2 (10 mL) and pyridine (1.2 mL, 15.0 mmol, 1.5 equiv.) were added. The reaction mixture was cooled at 0 °C and a solution of thiophene-2-sulfonyl chloride (1.83 g, 10.0 mmol, 1.0 equiv.) in CH_2Cl_2 (10 mL) was added dropwise by syringe over 30 min. The reaction mixture was stirred at 0 °C for 2 h and then warmed to room temperature and stirred for 10 hours. Water was added and the solution was extracted with CH_2Cl_2 (20 mL × 2). The combined organic layer was washed with water (30 mL × 1) and brine (20 mL × 1), dried over Na_2SO_4. Then remove the solvent, after purification by silica-gel chromatography (eluant: petroleum ether/ EtOAc 1:1, v/v) give the title compound **2x** as a yellow liquid (1.02 g, 44% yield).

IR (film): 3519, 3097, 2951, 2881, 1505, 1461, 1441, 1404, 1344, 1226, 1154, 1096, 1074, 1027, 972, 915, 855, 797, 725, 667, 609, 579, 559, 439 cm^{-1}. **^{1}H NMR** (400 MHz, $CDCl_3$) δ 7.60 – 7.59 (m, 2H), 7.14 – 7.12 (m, 1H), 4.42 – 4.36 (m, 1H), 3.45 – 3.41 (m, 3H), 3.31 – 3.25 (m, 1H), 1.97 – 1.81 (m, 3H). **^{13}C NMR** (100 MHz, $CDCl_3$) δ 136.64, 132.38, 131.95, 127.64, 70.78, 56.35, 46.36, 34.30. **HRMS (ESI)**: Calcd. For $C_8H_{12}O_3NS_2^+$: 234.0253; Found: 234.0254.

Synthesis of 1-((3,5-Dimethylisoxazol-4-yl)sulfonyl)pyrrolidin-3-ol (2y)

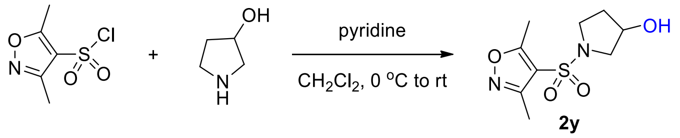

1-((3,5-Dimethylisoxazol-4-yl)sulfonyl)pyrrolidin-3-ol (**2y**) was prepared according to reference.[6] Starting from 3,5-dimethylisoxazole-4-sulfonyl chloride (1.96 g, 10.0

mmol), The title compound **2y** was obtained as as a white solid (600 mg, 23% yield).

M.p.: 65-68 °C. **IR** (film): 3525, 3446, 2985, 2951, 2890, 2251, 1589, 1501, 1480, 1440, 1408, 1372, 1341, 1263, 1229, 1202, 1177, 1125, 1075, 1033, 987, 974, 914, 798, 757, 733, 686, 641, 586, 570, 517, 441 cm^{-1}. **^{1}H NMR** (400 MHz, $CDCl_3$) δ 4.50 – 4.43 (m, 1H), 3.43 – 3.33 (m, 3H), 3.30 – 3.20 (m, 1H), 2.63 (s, 3H), 2.40 – 2.38 (m, 4H), 2.09 – 2.00 (m, 1H), 1.97 – 1.90 (m, 1H). **^{13}C NMR** (100 MHz, $CDCl_3$) δ 173.62, 158.14, 114.76, 70.53, 55.75, 45.55, 34.51, 13.07, 11.43. **HRMS (ESI)**: Calcd. For $C_9H_{15}O_4N_2S^+$: 247.0747; Found: 247.0745.

Synthesis of 1-((5-(Dimethylamino)naphthalen-1-yl)sulfonyl)pyrrolidin-3-ol (2ai)

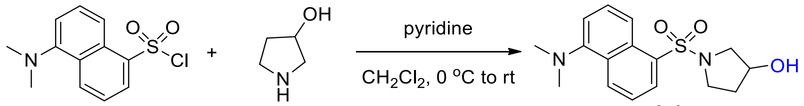

2ai

1-((5-(Dimethylamino)naphthalen-1-yl)sulfonyl)pyrrolidin-3-ol (**2ai**) was prepared according to reference.[6] Starting from dansyl chloride (2.70 g, 10.0 mmol), The title compound **2ai** was obtained as as a yellow solid (1.33 g, 42% yield).

M.p.: 88-91 °C. **IR** (film): 3506, 3079, 2944, 2833, 2788, 1611, 1587, 1572, 1503, 1478, 1455, 1406, 1319, 1230, 1202, 1185, 1143, 1094, 1076, 987, 944, 915, 838, 871, 791, 734, 682, 629, 597, 569, 541, 499, 462, 440 cm^{-1}. **^{1}H NMR** (400 MHz, $CDCl_3$) δ 8.54 (d, J = 8.5 Hz, 1H), 8.44 (d, J = 8.7 Hz, 1H), 8.22 (d, J = 7.3 Hz, 1H), 7.56 – 7.50 (m, 2H), 7.17 (d, J = 7.5 Hz, 1H), 4.45 – 4.39 (m, 1H), 3.56 – 3.44 (m, 3H), 3.39 – 3.37 (m, 1H), 2.88 (s, 6H), 2.05 – 1.86 (m, 3H). **^{13}C NMR** (100 MHz, $CDCl_3$) δ 151.80, 134.13, 130.61, 130.59, 130.19, 129.73, 128.19, 123.34, 119.77, 115.36, 71.13, 55.78, 45.68, 45.56, 34.64. **HRMS (ESI)**: Calcd. For $C_{16}H_{21}O_3N_2S^+$: 321.1267; Found: 321.1266.

Synthesis of 7-(3-Hydroxypropoxy)-3-(4-methoxyphenyl)-4*H*-chromen-4-one (2ao)

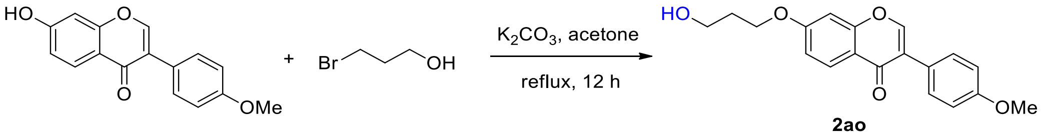

2ao

7-(3-Hydroxypropoxy)-3-(4-methoxyphenyl)-4*H*-chromen-4-one (**2ao**) was prepared

according to reference.[14] Starting from formononetin (2.64 g, 10.0 mmol), The title compound **2ao** was obtained as as a white solid (550 mg, 17% yield).

M.p.: 123-126 °C. **IR** (film): 3279, 3089, 2954, 2928, 2879, 2835, 1631, 1608, 1576, 1565, 1513, 1499, 1467, 1445, 1420, 1377, 1330, 1289, 1263, 1248, 1202, 1180, 1144, 1098, 992, 970, 939, 902, 886, 783, 695, 616, 540 cm^{-1}. **^{1}H NMR** (400 MHz, $CDCl_3$) δ 8.19 (d, J = 8.9 Hz, 1H), 7.91 (s, 1H), 7.50 (d, J = 8.6 Hz, 2H), 6.99 – 6.96 (m, 3H), 6.86 (d, J = 2.3 Hz, 1H), 4.21 (t, J = 6.1 Hz, 2H), 3.88 (dd, J = 10.9, 5.6 Hz, 2H), 3.84 (s, 3H), 2.10 (p, J = 6.0 Hz, 2H), 1.73 (t, J = 4.9 Hz, 1H). **^{13}C NMR** (100 MHz, $CDCl_3$) δ 176.03, 163.36, 159.72, 158.05, 152.23, 130.27, 127.92, 125.01, 124.35, 118.56, 114.93, 114.11, 100.80, 65.95, 59.77, 55.48, 31.93. **HRMS (ESI)**: Calcd. For $C_{19}H_{19}O_5^+$: 327.1227; Found: 327.1220.

Synthesis of (8*R*,9*S*,13*S*,14*S*)-3-(3-Hydroxypropoxy)-13-methyl-7,8,9,11,12,13,15,16-octa- hydro-6*H*-cyclopenta[*a*]phenanthren-17(14*H*)-one (2aq)

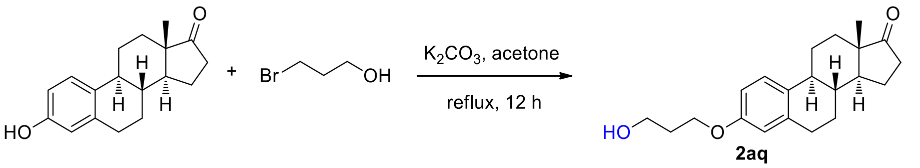

2aq

(8*R*,9*S*,13*S*,14*S*)-3-(3-Hydroxypropoxy)-13-methyl-7,8,9,11,12,13,15,16-octahydro-6*H*-cyclopenta[*a*]phenanthren-17(14*H*)-one (**2aq**) was prepared according to reference.[14] Starting from estrone (2.70 g, 10.0 mmol), The title compound **2aq** was obtained as as a white solid (1.24 g, 38% yield).

M.p.: 79-81 °C. **IR** (film): 3444, 2929, 2872, 2247, 1735, 1609, 1574, 1500, 1472, 1454, 1435, 1405, 1374, 1340, 1310, 1281, 1255, 1235, 1214, 1188, 1163, 1136, 1100, 1059, 1007, 961, 910, 872, 846, 818, 786, 732, 648, 581, 490, 446 cm^{-1}. **^{1}H NMR** (400 MHz, $CDCl_3$) δ 7.19 (d, J = 8.6 Hz, 1H), 6.72 (dd, J = 8.6, 2.6 Hz, 1H), 6.65 (d, J = 2.5 Hz, 1H), 4.10 (t, J = 5.9 Hz, 2H), 3.85 (t, J = 5.7 Hz, 2H), 2.96 – 2.83 (m, 2H), 2.53 – 2.46 (m, 1H), 2.43 – 2.33 (m, 1H), 2.28 – 2.21 (m, 1H), 2.18 – 2.10 (m, 1H), 2.09 – 1.93 (m, 6H), 1.67 – 1.37 (m, 6H), 0.90 (s, 3H). **^{13}C NMR** (100 MHz, $CDCl_3$) δ 221.15, 156.87, 137.89, 132.37, 126.46, 114.67, 112.20, 65.95, 60.73, 50.50, 48.12, 44.07, 38.46,

35.98, 32.13, 31.68, 29.75, 26.65, 26.03, 21.69, 13.96. **HRMS (ESI)**: Calcd. For $C_{21}H_{28}O_3Na^+$: 351.1931; Found: 351.1929.

Synthesis of 2-((1-Benzyl-1H-indazol-3-yl)oxy)ethanol (2au)

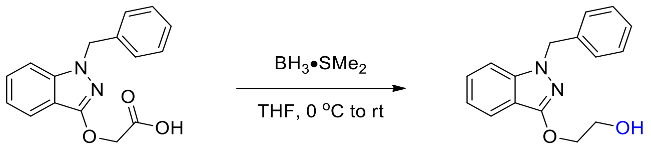

2-((1-Benzyl-1H-indazol-3-yl)oxy)ethanol (**2au**) was prepared according to reference.[12] A solution of BH_3•SMe_2 in THF (2 M, 2.07 mL, 4.2 mmol) was added slowly to an ice-cold solution of bendazac (1.13 g, 4.0 mmol) in THF (20 mL). The reaction was warmed up slowly from the ice-bath and stirred at room temperature overnight. Excess BH_3 was destroyed by the slow addition of MeOH (0.5 mL). The reaction mixture was evaporated to dryness under reduced pressure. The crude material was dissolved in CH_2Cl_2 (20 mL), washed with saturated $NaHCO_3$, water, 3 N HCl, water, and brine, dried over Na_2SO_4, filtered, concentrated under reduced pressure, and purified by column chromatography on silica gel (petroleum ether/EtOAc 1:1, v/v) to give **2au** as a white solid (765 mg, 71% yield).

M.p.: 76-78 °C. **IR** (film): 3373, 3093, 3064, 3031, 2932, 2877, 2247, 1618, 1581, 1530, 1496, 1454, 1442, 1350, 1311, 1256, 1188, 1146, 1104, 1077, 1047, 1005, 960, 908, 847, 823, 770, 733, 702, 651, 631, 589, 554, 482, 454, 428 cm^{-1}. **^{1}H NMR** (400 MHz, $CDCl_3$) δ 7.69 (d, J = 8.1 Hz, 1H), 7.37 – 7.27 (m, 4H), 7.25 – 7.19 (m, 3H), 7.07 (t, J = 7.5 Hz, 1H), 5.39 (s, 2H), 4.56 – 4.54 (m, 2H), 4.04 – 4.02 (m, 2H), 3.29 (s, 1H). **^{13}C NMR** (100 MHz, $CDCl_3$) δ 156.06, 141.77, 137.23, 128.76, 127.82, 127.74, 127.23, 120.23, 119.54, 112.93, 109.07, 71.53, 62.45, 52.49. **HRMS (ESI)**: Calcd. For $C_{16}H_{17}O_2N_2^+$: 269.1285; Found: 269.1283.

Synthesis of 3-((2,5,7,8-tetramethyl-2-(4,8,12-trimethyltridecyl)chroman-6-yl)oxy)propa- n-1-ol (2aw)

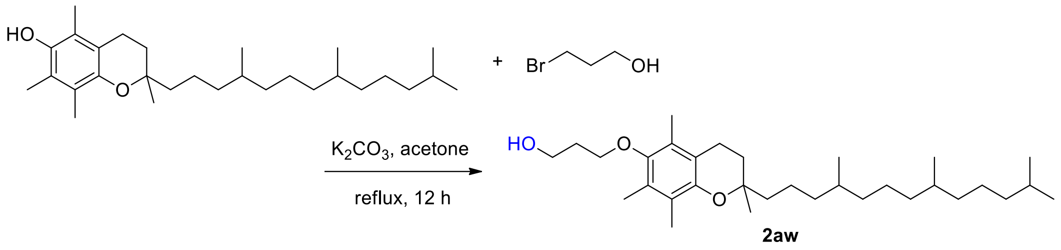

3-((2,5,7,8-tetramethyl-2-(4,8,12-trimethyltridecyl)chroman-6-yl)oxy)propan-1-ol (**2aw**) was prepared according to reference.[14] Starting from DL-α-tocopherol (4.30 g, 10.0 mmol), The title compound **2aw** was obtained as as a light yellow liquid (1.06 g, 22% yield).

IR (film): 3418, 2926, 2900, 1641, 1456, 1414, 1378, 1257, 1157, 1087, 1048, 945, 879 cm^{-1}. **^{1}H NMR** (400 MHz, $CDCl_3$) [mixture of rotamers] δ 4.00 – 3.93 (m, 2H), 3.84 (t, J = 5.8 Hz, 2H), 2.58 (t, J = 6.7 Hz, 2H), 2.39 (s, 1H), 2.18 (s, 3H), 2.14 (s, 3H), 2.09 (s, 3H), 2.06 – 2.02 (m, 2H), 1.85 – 1.72 m, 1H), 1.63 (s, 1H), 1.59 – 1.49 (m, 3H), 1.48 – 1.35 (m, 4H), 1.31 – 1.18 (m, 10H), 1.16 – 1.00 (m, 6H), 0.88 (s, 3H), 0.86 – 0.84 (m, 9H). **^{13}C NMR** (100 MHz, $CDCl_3$) [mixture of rotamers] δ 148.03 (s, 2C), 127.77, 125.78, 123.04, 117.66, 77.36, 74.89, 71.85, 61.77, 40.16 [40.11], 39.48, 37.68 – 37.40 (m, 4C), 32.89 [32.87], 32.79 –32.77 (m, 2C), 31.39 [31.34], 28.08, 24.93 [24.91], 24.54, 23.96, 22.83, 22.74, 21.14, 20.76, 19.86 – 19.71 (m, 2C), 12.78, 11.92, 11.89. **HRMS (ESI)**: Calcd. For $C_{32}H_{57}O_3^+$: 489.4302; Found: 489.4294.

Synthesis of (8*R*,9*S*,13*S*,14*S*,17*S*)-3-(3-Hydroxypropoxy)-13-methyl-7,8,9,11,12,13,14,15,16,17-decahydro-6*H*-cyclopenta[*a*]phenanthren-17-yl heptanoate (2ax)

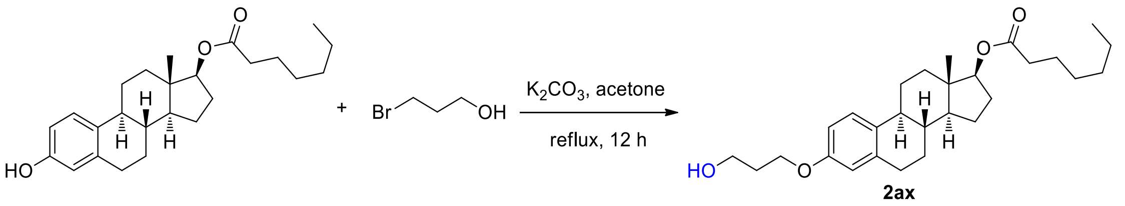

(8*R*,9*S*,13*S*,14*S*,17*S*)-3-(3-Hydroxypropoxy)-13-methyl-7,8,9,11,12,13,14,15,16, 17-decahydro-6*H*-cyclopenta[*a*]phenanthren-17-yl heptanoate (**2ax**) was prepared according to reference.[14] Starting from estradiol (3.85 g, 10.0 mmol), The title compound **2ax** was obtained as as a light yellow liquid (3.60 g, 81% yield).

IR (film): 3406, 2928, 2871, 1734, 1607, 1577, 1500, 1468, 1382, 1310, 1280, 1254, 1236, 1171, 1100, 1053, 1009, 961, 878, 814, 788, 572, 447 cm^{-1}. **^{1}H NMR** (400 MHz, $CDCl_3$) δ 7.19 (d, J = 8.4 Hz, 1H), 6.72 – 6.69 (m, 1H), 6.64 (s, 1H), 4.70 (t, J = 8.4 Hz, 1H), 4.10 (t, J = 5.9 Hz, 2H), 3.85 (t, J = 5.8 Hz, 2H), 2.91 – 2.79 (m, 2H), 2.33 – 2.17 (m, 5H), 2.11 – 2.00 (m, 3H), 1.94 – 1.83 (m, 2H), 1.78 – 1.70 (m, 1H), 1.69 – 1.59 (m, 2H), 1.58 – 1.52 (m, 1H), 1.50 – 1.24 (m, 12H), 0.91 – 0.88 (m, 3H), 0.83 (s, 3H). **^{13}C NMR** (100 MHz, $CDCl_3$) δ 174.13, 156.72, 138.04, 132.88, 126.46, 114.59, 112.09, 82.55, 65.94, 60.73, 49.88, 43.88, 43.06, 38.67, 37.02, 34.73, 32.13, 31.57, 29.86, 28.92, 27.70, 27.32, 26.31, 25.19, 23.37, 22.60, 14.14, 12.20. **HRMS (ESI)**: Calcd. For $C_{28}H_{43}O_4^+$: 443.3156; Found: 443.3151.

3. Deoxyazidation of Alcohols with NaN_3 by SulfoxFluor

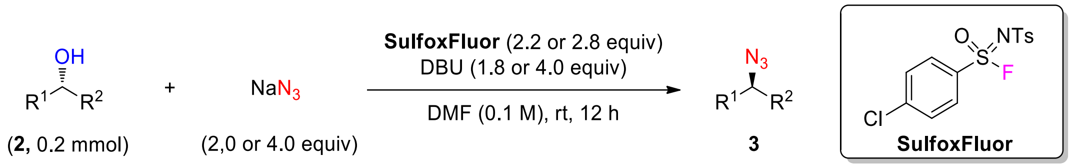

For Primary Alcohols (Method A):

Into a 25-mL Schlenk tube (glass) were sequentially added alcohol **2** (0.2 mmol), SulfoxFluor (152.9 mg, 0.44 mmol, 2.2 equiv), NaN_3 (52.0 mg, 0.8 mmol), DMF (2.0 mL), and DBU (54 μL, 0.36 mmol, 1.8 equiv) under N_2 atmosphere. The mixture was stirred at room temperature for 12 hours. Then water (2 ~ 5 mL) was added and the mixture was extracted with Et_2O (3 × 2 mL). The combined organic layers were dried over Na_2SO_4, filtered, concentrated under reduced pressure, and purified by chromatography on silica gel to afford the desired products.

For Secondary Alcohols (Method B):

Into a 25-mL Schlenk tube (glass) were sequentially added alcohol **2** (0.2 mmol), SulfoxFluor (194.6 mg, 0.56 mmol, 2.8 equiv), NaN_3 (26.0 mg, 0.4 mmol), DMF (2.0 mL), and DBU (120 μL, 0.80 mmol, 4.0 equiv) under N_2 atmosphere. The mixture was stirred at room temperature for 12 hours. Then water (2 ~ 5 mL) was added and

the mixture was extracted with Et_2O (3 × 2 mL). The combined organic layers were dried over Na_2SO_4, filtered, concentrated under reduced pressure, and purified by chromatography on silica gel to afford the desired products.

1-(2-Azidoethyl)naphthalene (3d)

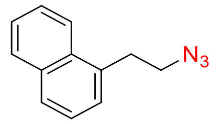

Following the general procedure for primary alcohols (Method A), prepared from 2-(naphthalen-1-yl)ethan-1-ol (**2d**) (34.4 mg, 0.2 mmol, 1.0 equiv), SulfoxFluor (152.9 mg, 0.44 mmol, 2.2 equiv), NaN_3 (52.0 mg, 0.8 mmol, 4.0 equiv), DBU (54 µL, 0.36 mmol, 1.8 equiv) in DMF for 12 hours; eluted with petroleum ether/EtOAc, 30:1 (v/v).

37.7 mg, 96% yield. Light yellow liquid. **IR** (film): 3063, 3047, 3006, 2929, 2874, 2096, 1597, 1512, 1463, 1450, 1396, 1349, 1298, 1265, 1218, 1166, 1126, 1111, 1080, 1067, 1024, 968, 949, 915, 901, 860, 797, 776, 734, 696, 672, 635, 591, 554, 513, 486, 437 cm^{-1}. **^{1}H NMR** (400 MHz, $CDCl_3$) δ 8.02 (d, J = 8.3 Hz, 1H), 7.92 – 7.90 (m, 1H), 7.81 (d, J = 8.1 Hz, 1H), 7.60 – 7.51 (m, 2H), 7.48 – 7.44 (m, 1H), 7.41 – 7.40 (m, 1H), 3.65 (t, J = 7.5 Hz, 2H), 3.39 (t, J = 7.5 Hz, 2H). **^{13}C NMR** (100 MHz, $CDCl_3$) δ 134.02, 133.96, 131.79, 129.10, 127.76, 127.08, 126.36, 125.81, 125.65, 123.23, 51.81, 32.56. **MS** (**EI**, m/z, %): 197 (M^+, 4.82), 141 (100.00); **HRMS (EI)**: Calcd. For $C_{12}H_{11}N_3^+$: 197.0947; Found: 197.0959. The NMR data are in agreement with the literature.[15]

4-(2-Azidoethyl)-1,1'-biphenyl (3e)

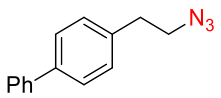

Following the general procedure for primary alcohols (Method A), prepared from 2-([1,1'-biphenyl]-4-yl)ethanol (**2e**) (39.6 mg, 0.2 mmol, 1.0 equiv), SulfoxFluor (152.9 mg, 0.44 mmol, 2.2 equiv), NaN_3 (52.0 mg, 0.8 mmol, 4.0 equiv), DBU (54 µL, 0.36 mmol, 1.8 equiv) in DMF for 12 hours; eluted with petroleum ether/EtOAc, 10:1

(v/v).

41.0 mg, 92% yield. Light yellow liquid. **IR** (film): 3084, 3056, 3028, 2927, 2869, 2097, 1601, 1564, 1519, 1486, 1450, 1410, 1347, 1300, 1283, 1262, 1246, 1208, 1157, 1115, 1076, 1040, 1008, 996, 965, 933, 911, 839, 761, 731, 697, 670, 583, 552, 507 cm^{-1}. **^{1}H NMR** (400 MHz, $CDCl_3$) δ 7.63 – 7.58 (m, 4H), 7.47 (t, *J* = 7.6 Hz, 2H), 7.39 – 7.36 (m, 1H), 7.32 (d, *J* = 8.1 Hz, 2H), 3.57 (t, *J* = 7.2 Hz, 2H), 2.96 (t, *J* = 7.2 Hz, 2H). **^{13}C NMR** (100 MHz, $CDCl_3$) δ 140.91, 139.87, 137.19, 129.28, 128.88, 127.48, 127.35, 127.14, 52.52, 35.08. **MS** (**EI**, m/z, %): 223 (M^+, 21.98), 167 (100.00); **HRMS (EI)**: Calcd. For $C_{14}H_{13}N_3^+$: 223.1104; Found: 223.1119.

1-Azidohexadecane (3f)

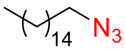

Following the general procedure for primary alcohols (Method A), prepared from 1-hexadecanol (**2f**) (48.4 mg, 0.2 mmol, 1.0 equiv), SulfoxFluor (152.9 mg, 0.44 mmol, 2.2 equiv), NaN_3 (52.0 mg, 0.8 mmol, 4.0 equiv), DBU (54 μL, 0.36 mmol, 1.8 equiv) in DMF for 12 hours; eluted with petroleum ether/EtOAc, 15:1 (v/v).

43.0 mg, 81% yield. Light yellow liquid. **IR** (film): 2923, 2853, 2095, 1466, 1377, 1349, 1285, 1258, 1173, 1132, 894, 721, 679, 662, 638, 557 cm^{-1}. **^{1}H NMR** (400 MHz, $CDCl_3$) δ 3.25 (t, *J* = 7.0 Hz, 2H), 1.59 (p, *J* = 6.9 Hz, 2H), 1.26 (s, 26H), 0.88 (t, *J* = 6.6 Hz, 3H). **^{13}C NMR** (100 MHz, $CDCl_3$) δ 51.64, 32.09, 29.85, 29.82, 29.79, 29.71, 29.64, 29.52, 29.32, 29.00, 26.88, 22.85, 14.26. **MS** (**EI**, m/z, %): 267 (M^+, 0.13), 70 (100.00); **HRMS (EI)**: Calcd. For $C_{16}H_{33}N_3^+$: 267.2669; Found: 267.2672. The NMR data are in agreement with the literature.[16]

***tert*-Butyl (3-azidopropyl)carbamate (3g)**

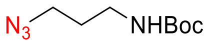

Following the general procedure for primary alcohols (Method A), prepared from 3-(Boc-amino)-1-propanol (**2g**) (35.0 mg, 0.2 mmol, 1.0 equiv), SulfoxFluor (152.9 mg, 0.44 mmol, 2.2 equiv), NaN_3 (52.0 mg, 0.8 mmol, 4.0 equiv), DBU (54 μL, 0.36

mmol, 1.8 equiv) in DMF for 12 hours; eluted with petroleum ether/EtOAc, 8:1 (v/v).

24.0 mg, 60% yield. Colorless liquid. **IR** (film): 3329, 3009, 2978, 2932, 2874, 2097, 1692, 1519, 1453, 1392, 1366, 1272, 1252, 1172, 1040, 1009, 972, 905, 865, 781, 753, 558 cm^{-1}. **^{1}H NMR** (400 MHz, $CDCl_3$) δ 4.68 (s, 1H), 3.35 (t, J = 6.6 Hz, 2H), 3.22 – 3.18 (m, 2H), 1.79 – 1.73 (m, 2H), 1.43 (s, 9H). **^{13}C NMR** (100 MHz, $CDCl_3$) δ 156.07, 79.54, 49.26, 38.16, 29.41, 28.51. **MS** (**EI**, m/z, %): 201 (M^+, 30.28), 57 (100.00); **HRMS (EI)**: Calcd. For $C_8H_{17}O_2N_4^+$: 201.1346; Found: 201.1352.The NMR data are in agreement with the literature.[17]

2-(2-Azidoethyl)isoindoline-1,3-dione (3h)

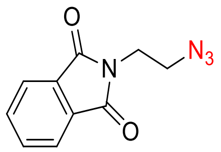

Following the general procedure for primary alcohols (Method A), prepared from *N*-hydroxyethylphthalimide (**2h**) (38.2 mg, 0.2 mmol, 1.0 equiv), SulfoxFluor (152.9 mg, 0.44 mmol, 2.2 equiv), NaN_3 (52.0 mg, 0.8 mmol, 4.0 equiv), DBU (54 µL, 0.36 mmol, 1.8 equiv) in DMF for 12 hours; eluted with petroleum ether/EtOAc, 10:1 (v/v).

31.7 mg, 73% yield. Light yellow liquid. **IR** (film): 3472, 3327, 3084, 3062, 3032, 2939, 2873, 2109, 1775, 1712, 1614, 1497, 1467, 1430, 1393, 1374, 1350, 1309, 1231, 1190, 1172, 1137, 1089, 1071, 1020, 987, 962, 886, 972, 817, 793, 718, 696, 659, 614, 603, 554, 529, 434 cm^{-1}. **^{1}H NMR** (400 MHz, $CDCl_3$) δ 7.88 – 7.84 (m, 2H), 7.75 – 7.71 (m, 2H), 3.89 (t, J = 6.1 Hz, 2H), 3.59 (t, J = 6.1 Hz, 2H). **^{13}C NMR** (100 MHz, $CDCl_3$) δ 168.13, 134.31, 131.98, 123.58, 49.09, 36.99. **MS** (**EI**, m/z, %): 216 (M^+, 0.03), 161 (100.00); **HRMS (EI)**: Calcd. For $C_{10}H_8N_4O_2^+$: 216.0642; Found: 216.0642. The NMR data are in agreement with the literature.[18]

Ethyl 4-((5-azidopentyl)oxy)benzoate (3i)

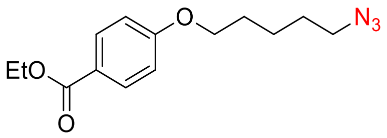

Following the general procedure for primary alcohols (Method A), prepared from ethyl 4-((5-hydroxypentyl)oxy)benzoate (**2i**) (50.4 mg, 0.2 mmol, 1.0 equiv), SulfoxFluor (152.9 mg, 0.44 mmol, 2.2 equiv), NaN_3 (52.0 mg, 0.8 mmol, 4.0 equiv), DBU (54 μL, 0.36 mmol, 1.8 equiv) in DMF for 12 hours; eluted with petroleum ether/EtOAc, 10:1 (v/v).

50.8 mg, 92% yield. Light yellow liquid. **IR** (film): 3077, 2985, 2941, 2870, 2096, 1712, 1606, 1580, 1511, 1469, 1421, 1391, 1366, 1316, 1281, 1253, 1168, 1103, 1072, 1050, 1021, 981, 905, 848, 817, 771, 735, 697, 647, 632, 612, 558, 514 cm^{-1}. **^{1}H NMR** (400 MHz, $CDCl_3$) δ 7.98 (d, J = 8.8 Hz, 2H), 6.89 (d, J = 8.8 Hz, 2H), 4.33 (q, J = 7.1 Hz, 2H), 4.00 (t, J = 6.3 Hz, 2H), 3.30 (t, J = 6.7 Hz, 2H), 1.82 (dt, J = 14.1, 6.4 Hz, 2H), 1.71 – 1.63 (m, 2H), 1.60 – 1.51 (m, 2H), 1.37 (t, J = 7.1 Hz, 3H). **^{13}C NMR** (100 MHz, $CDCl_3$) δ 166.47, 162.76, 131.61, 122.93, 114.06, 67.82, 60.70, 51.39, 28.77, 28.71, 23.43, 14.47. **HRMS (ESI)**: Calcd. For $C_{14}H_{20}O_3N_3^+$: 278.1499; Found: 278.1500.

1-(5-Azidopent-1-yn-1-yl)-4-methoxybenzene (3j)

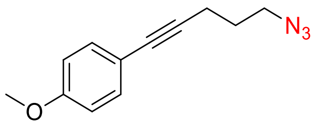

Following the general procedure for primary alcohols (Method A), prepared from 5-(4-methoxyphenyl)pent-4-yn-1-ol (**2j**) (38.0 mg, 0.2 mmol, 1.0 equiv), SulfoxFluor (152.9 mg, 0.44 mmol, 2.2 equiv), NaN_3 (52.0 mg, 0.8 mmol, 4.0 equiv), DBU (54 μL, 0.36 mmol, 1.8 equiv) in DMF for 12 hours; eluted with petroleum ether/EtOAc, 15:1 (v/v).

36.8 mg, 86% yield. Light yellow liquid. **IR** (film): 3039, 3002, 2959, 2934, 2871, 2837, 2098, 1607, 1568, 1509, 1463, 1442, 1411, 1364, 1345, 1289, 1246, 1173, 1106, 1074, 1033, 942, 911, 882, 832, 815, 800, 734, 649, 632, 551, 535 cm^{-1}. **^{1}H NMR** (400 MHz, $CDCl_3$) δ 7.34 (d, J = 8.8 Hz, 1H), 6.82 (d, J = 8.9 Hz, 1H), 3.79 (s, 2H), 3.47 (t, J = 6.7 Hz, 1H), 2.52 (t, J = 6.8 Hz, 1H), 1.86 (p, J = 6.8 Hz, 1H). **^{13}C NMR** (100 MHz, $CDCl_3$) δ 159.30, 133.00, 115.79, 113.95, 86.64, 81.48, 55.34, 50.42, 28.12, 16.83. **MS** (**EI**, m/z, %): 215 (M^+, 0.04), 159 (100.00); **HRMS (EI)**: Calcd.

For $C_{12}H_{13}N_3O^+$: 215.1053; Found: 215.1066. The NMR data are in agreement with the literature.[19]

3-(2-Azidoethyl)-1-methyl-1*H*-indole (3k)

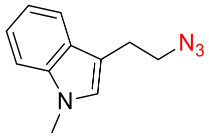

Following the general procedure for primary alcohols (Method A), prepared from 2-(1-methyl-1*H*-indol-3-yl)ethanol (**2k**) (35.0 mg, 0.2 mmol, 1.0 equiv), SulfoxFluor (152.9 mg, 0.44 mmol, 2.2 equiv), NaN_3 (52.0 mg, 0.8 mmol, 4.0 equiv), DBU (54 µL, 0.36 mmol, 1.8 equiv) in DMF for 12 hours; eluted with petroleum ether/EtOAc, 10:1 (v/v).

27.8 mg, 70% yield. Light yellow liquid. **IR** (film): 3054, 3032, 2929, 2877, 2829, 2096, 1615, 1585, 1553, 1473, 1425, 1378, 1346, 1324, 1297, 1251, 1210, 1182, 1155, 1132, 1124, 1068, 1013, 959, 924, 903, 803, 739, 696, 674, 639, 601, 563, 427 cm^{-1}. **^{1}H NMR** (400 MHz, $CDCl_3$) δ 7.61 (d, J = 7.9 Hz, 1H), 7.34 – 7.32 (m, 1H), 7.27 (t, J = 7.5 Hz, 1H), 7.16 (t, J = 7.4 Hz, 1H), 6.95 (s, 1H), 3.78 (s, 3H), 3.58 (t, J = 7.2 Hz, 2H), 3.08 (t, J = 7.2 Hz, 2H). **^{13}C NMR** (100 MHz, $CDCl_3$) δ 137.12, 127.67, 127.13, 121.86, 119.09, 118.71, 110.84, 109.48, 51.96, 32.77, 25.09. **MS** (**EI**, m/z, %): 200 (M^+, 27.44), 144 (100.00); **HRMS (EI)**: Calcd. For $C_{11}H_{12}N_4^+$: 200.1056; Found: 200.1061. The NMR data are in agreement with the literature.[20]

2-(2-(3-Azidopropoxy)phenyl)benzo[*d*]thiazole (3l)

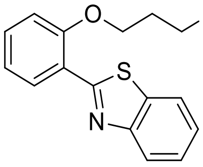

Following the general procedure for primary alcohols (Method A), prepared from 3-(2-(benzo[*d*]thiazol-2-yl)phenoxy)propan-1-ol (**2l**) (57.0 mg, 0.2 mmol, 1.0 equiv), SulfoxFluor (152.9 mg, 0.44 mmol, 2.2 equiv), NaN_3 (52.0 mg, 0.8 mmol, 4.0 equiv),

DBU (54 μL, 0.36 mmol, 1.8 equiv) in DMF for 12 hours; eluted with petroleum ether/EtOAc, 10:1 (v/v).

57.0 mg, 92% yield. Colorless liquid. **IR** (film): 3060, 2952, 2933, 2880, 2097, 1598, 1582, 1556, 1498, 1452, 1433, 1392, 1345, 1305, 1291, 1246, 1217, 1162, 1117, 1049, 971, 909, 852, 823, 755, 731, 695, 660, 627, 611, 568, 528, 459 cm^{-1}. **^{1}H NMR** (400 MHz, $CDCl_3$) δ 8.56 (dd, J = 7.9, 1.7 Hz, 1H), 8.11 (d, J = 8.2 Hz, 1H), 7.95 – 7.93 (m, 1H), 7.52 – 7.48 (m, 1H), 7.46 – 7.37 (m, 2H), 7.14 (t, J = 7.6 Hz, 1H), 7.02 (d, J = 8.3 Hz, 1H), 4.26 (t, J = 5.8 Hz, 2H), 3.68 (t, J = 6.7 Hz, 2H), 2.25 (p, J = 6.3 Hz, 2H). **^{13}C NMR** (100 MHz, $CDCl_3$) δ 162.85, 156.29, 152.17, 135.94, 131.90, 129.83, 126.09, 124.79, 122.91, 122.32, 121.42, 121.31, 112.27, 65.87, 48.53, 28.91. **HRMS (ESI)**: Calcd. For $C_{16}H_{15}ON_4S^+$: 311.0961; Found: 311.0962.

4-(3-Azidopropyl)pyridine (3m)

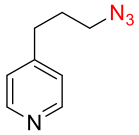

Following the general procedure for primary alcohols (Method A), prepared from 4-pyridinepropanol (**2m**) (27.4 mg, 0.2 mmol, 1.0 equiv), SulfoxFluor (152.9 mg, 0.44 mmol, 2.2 equiv), NaN_3 (52.0 mg, 0.8 mmol, 4.0 equiv), DBU (54 μL, 0.36 mmol, 1.8 equiv) in DMF for 12 hours; eluted with petroleum ether/EtOAc, 2:1 (v/v).

26.8 mg, 83% yield. Light yellow liquid. **IR** (film): 3069, 3026, 2929, 2867, 2098, 1644, 1602, 1558, 1496, 1453, 1415, 1350, 1292, 1259, 1220, 1158, 1070, 992, 959, 919, 893, 857, 834, 796, 755, 661, 635, 581, 557, 511, 494, 443 cm^{-1}. **^{1}H NMR** (400 MHz, $CDCl_3$) δ 8.50 (s, 2H), 7.11 – 7.10 (m, 2H), 3.29 (t, J = 6.6 Hz, 2H), 2.70 – 2.67 (m, 2H), 1.94 – 1.86 (m, 2H). **^{13}C NMR** (100 MHz, $CDCl_3$) δ 149.92, 149.89, 123.99, 50.53, 32.17, 29.46. **MS** (**EI**, m/z, %): 162 (M^+, 0.06), 133 (100.00); **HRMS (EI)**: Calcd. For $C_8H_{10}N_4^+$: 162.0900; Found: 162.0910.

5-(2-Azidoethyl)-4-methylthiazole (3n)

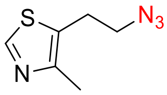

Following the general procedure for primary alcohols (Method A), prepared from 5-(2-hydroxyethyl)-4-methylthiazole (**2n**) (27.4 mg, 0.2 mmol, 1.0 equiv), SulfoxFluor (152.9 mg, 0.44 mmol, 2.2 equiv), NaN_3 (52.0 mg, 0.8 mmol, 4.0 equiv), DBU (54 µL, 0.36 mmol, 1.8 equiv) in DMF for 12 hours; eluted with petroleum ether/EtOAc, 8:1 (v/v).

25.1 mg, 75% yield. Light yellow liquid. **IR** (film): 3077, 2926, 2867, 2103, 1543, 1451, 1415, 1378, 1350, 1296, 1262, 1240, 1204, 1159, 1079, 1037, 1013, 941, 903, 843, 794, 700, 648, 553, 512, 447 cm^{-1}. **^{1}H NMR** (400 MHz, $CDCl_3$) δ 8.60 (s, 1H), 3.49 (t, J = 6.8 Hz, 2H), 3.02 (t, J = 6.9 Hz, 2H), 2.41 (s, 3H). **^{13}C NMR** (100 MHz, $CDCl_3$) δ 150.13, 150.10, 127.12, 52.12, 26.34, 15.01. **MS** (**EI**, m/z, %): 168 (M^+, 7.90), 112 (100.00); **HRMS (EI)**: Calcd. For $C_6H_8N_4S^+$: 168.0464; Found: 168.0475. The NMR data are in agreement with the literature.[21]

3-(2-Azidoethyl)benzo[*b*]thiophene (3o)

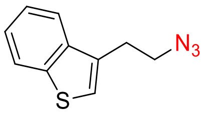

Following the general procedure for primary alcohols (Method A), prepared from 2-(benzo[*b*]thiophen-3-yl)ethanol (**2o**) (35.6 mg, 0.2 mmol, 1.0 equiv), SulfoxFluor (152.9 mg, 0.44 mmol, 2.2 equiv), NaN_3 (52.0 mg, 0.8 mmol, 4.0 equiv), DBU (54 µL, 0.36 mmol, 1.8 equiv) in DMF for 12 hours; eluted with petroleum ether/EtOAc, 15:1 (v/v).

39.2 mg, 97% yield. Light yellow liquid. **IR** (film): 3068, 3028, 2927, 2870, 2094, 1459, 1428, 1366, 1348, 1296, 1262, 1194, 1158, 1138, 1085, 1059, 1020, 977, 936, 910, 845, 823, 760, 732, 706, 640, 620, 581, 440, 421 cm^{-1}. **^{1}H NMR** (400 MHz, $CDCl_3$) δ 7.89 (d, J = 7.4 Hz, 1H), 7.76 – 7.74 (m, 1H), 7.45 – 7.37 (m, 2H), 7.24 (s, 1H), 3.64 (t, J = 7.2 Hz, 2H), 3.16 (t, J = 7.2 Hz, 2H). **^{13}C NMR** (100 MHz, $CDCl_3$) δ 140.57, 138.60, 132.46, 124.54, 124.23, 123.20, 123.14, 121.39, 50.79, 28.26. **MS** (**EI**, m/z, %): 203 (M^+, 17.11), 147 (100.00); **HRMS (EI)**: Calcd. For $C_{10}H_9N_3S^+$: 203.0512; Found: 203.0513.

Methyl 4-(azidomethyl)benzoate (3p)

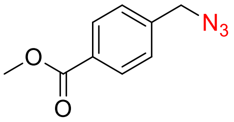

Following the general procedure for primary alcohols (Method A) with modification, prepared from methyl 4-(hydroxymethyl)benzoate (**2p**) (33.2 mg, 0.1 mmol, 1.0 equiv), SulfoxFluor (173.8 mg, 0.5 mmol, 2.5 equiv), NaN_3 (65.0 mg, 1.0 mmol, 5.0 equiv), DBU (54 μL, 0.18 mmol, 1.8 equiv) in DMF for 12 hours. eluted with petroleum ether/EtOAc, 40:1 (v/v).

19.0 mg, 50% yield. **IR** (film): 2961, 2922, 2848, 2101, 1723, 1582, 1514, 1435, 1415, 1343, 1280, 1196, 1108, 1020, 965, 860, 834, 797, 756, 712, 558 cm^{-1}. **^{1}H NMR** (400 MHz, $CDCl_3$) δ 8.06 (d, J = 8.2 Hz, 2H), 7.39 (d, J = 8.1 Hz, 2H), 4.42 (s, 2H), 3.92 (s, 3H). **^{13}C NMR** (100 MHz, $CDCl_3$) δ 166.77, 140.54, 130.26, 130.22, 128.06, 54.44, 52.34. **MS** (**EI**, m/z, %): 191 (M^+, 49.06), 149 (100.00); **HRMS (EI)**: Calcd. For $C_9H_9O_2N_3^+$: 191.0689; Found: 191.0687. The NMR data are in agreement with the literature.[22]

(*R*)-1-(2-Azidopropyl)naphthalene (3q)

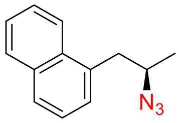

Following the general procedure for secondary alcohols (Method B), prepared from (*S*)-1-(naphthalen-1-yl)propan-2-ol (**2q**) (37.2 mg, 0.2 mmol, 1.0 equiv), SulfoxFluor (194.6 mg, 0.56 mmol, 2.8 equiv), NaN_3 (26.0 mg, 0.4 mmol, 2.0 equiv), DBU (120 μL, 0.80 mmol, 4.0 equiv) in DMF for 12 hours; eluted with petroleum ether/EtOAc, 10:1 (v/v).

40.2 mg, 95% yield. Light yellow liquid. $[\alpha]_D^{30.6}$ = –73.7° (c = 1.27, $CHCl_3$). **IR** (film): 3067, 3046, 3007, 2971, 2930, 2870, 2107, 1596, 1510, 1456, 1396, 1379, 1328, 1260, 1165, 1142, 1120, 1089, 1026, 967, 951, 904, 856, 870, 790, 776, 733, 668, 626, 593, 554, 514, 429 cm^{-1}. **^{1}H NMR** (400 MHz, $CDCl_3$) δ 8.01 (d, J = 8.3 Hz, 1H), 7.91 – 7.88 (m, 1H), 7.80 (d, J = 8.2 Hz, 1H), 7.59 – 7.50 (m, 2H), 7.47 – 7.43 (m, 1H), 7.39 – 7.38 (m, 1H), 3.94 – 3.86 (m, 1H), 3.27 (ddd, J = 74.5, 13.9, 7.0 Hz,

2H), 1.33 (d, *J* = 6.5 Hz, 3H). **^{13}C NMR** (100 MHz, $CDCl_3$) *δ* 134.06, 133.97, 132.02, 129.10, 127.89, 127.77, 126.26, 125.76, 125.57, 123.52, 58.36, 39.86, 19.63. **MS (EI**, m/z, %): 211 (M^+, 2.09), 141 (100.00); **HRMS (EI)**: Calcd. For $C_{13}H_{13}N_3^+$: 211.1104; Found: 211.1114.

HPLC Traces for Measuring Enantiomeric Excess: A racemic sample of compound **3q** was obtained through deoxy-azidation of racemic alcohol **2q** with SulfoxFluor. The racemic and optically active **3q** were analyzed with HPLC (Chiralcel AD-H, hexane, λ = 214 nm, 0.70 mL/min) to determine the retention time and enantiomeric excesses. For (*R*)-**3q**, *e.e.* = 98.6%. [*e.s.*% = 98.6/99.96*100% = 98.6%].

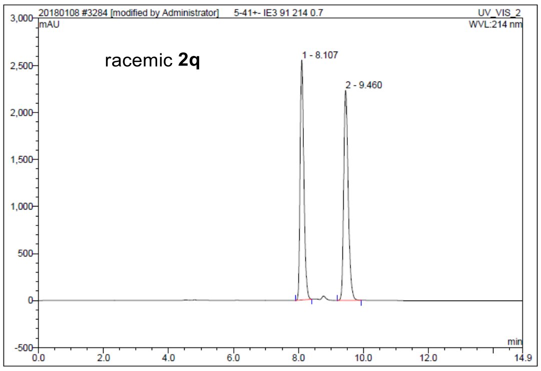

No.	Ret.Time min	Peak Name	Height mAU	Area mAU*min	Rel.Area %	Amount	Type
1	8.11	n.a.	2551.836	342.802	48.90	n.a.	BMB*
2	9.46	n.a.	2234.141	358.236	51.10	n.a.	BMB
Total:			4785.977	701.038	100.00	0.000	

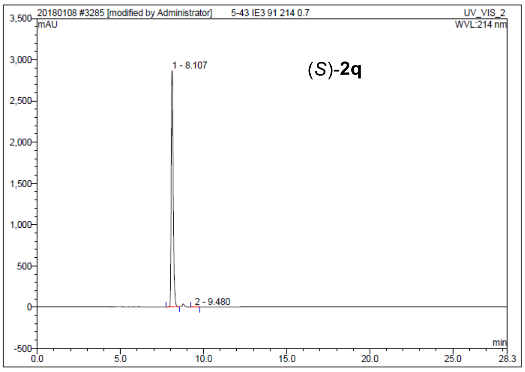

No.	Ret.Time min	Peak Name	Height mAU	Area mAU*min	Rel.Area %	Amount	Type
1	8.11	n.a.	2864.456	398.440	99.98	n.a.	BMB*
2	9.48	n.a.	1.138	0.070	0.02	n.a.	BMB*
Total:			2865.593	398.510	100.00	0.000	

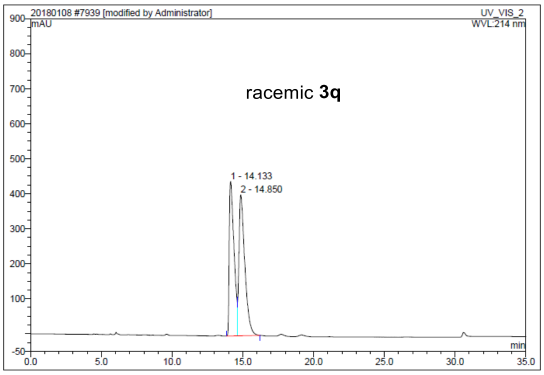

No.	Ret.Time min	Peak Name	Height mAU	Area mAU*min	Rel.Area %	Amount	Type
1	14.13	n.a.	441.475	172.761	47.04	n.a.	BM
2	14.85	n.a.	402.690	194.522	52.96	n.a.	MB
Total:			844.165	367.283	100.00	0.000	

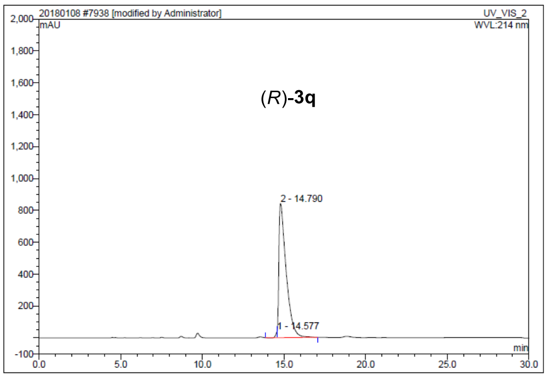

No.	Ret.Time min	Peak Name	Height mAU	Area mAU*min	Rel.Area %	Amount	Type
1	14.58	n.a.	39.553	3.055	0.70	n.a.	BM *
2	14.79	n.a.	841.412	436.314	99.30	n.a.	MB*
Total:			880.965	439.370	100.00	0.000	

1-(3-Azidobutyl)-4-(benzyloxy)benzene (3r)

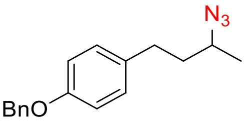

Following the general procedure for secondary alcohols (Method B), prepared from 4-(4-(benzyloxy)phenyl)butan-2-ol (**2r**) (51.2 mg, 0.2 mmol, 1.0 equiv), SulfoxFluor (194.6 mg, 0.56 mmol, 2.8 equiv), NaN_3 (26.0 mg, 0.4 mmol, 2.0 equiv), DBU (120 µL, 0.80 mmol, 4.0 equiv) in DMF for 12 hours; eluted with petroleum ether/EtOAc, 20:1 (v/v).

55.9 mg, 99% yield. Colorless liquid. **IR** (film): 3064, 3032, 2969, 2929, 2861, 2099, 1721, 1611, 1583, 1511, 1454, 1380, 1297, 1240, 1176, 1124, 1080, 1026, 909, 861, 827, 736, 696, 639, 611, 549, 515, 456 cm^{-1}. **^{1}H NMR** (400 MHz, $CDCl_3$) δ 7.47 – 7.45 (m, 2H), 7.41 (t, J = 7.3 Hz, 2H), 7.37 – 7.33 (m, 1H), 7.13 (d, J = 8.5 Hz, 2H), 6.94 (d, J = 8.6 Hz, 2H), 5.07 (s, 2H), 3.49 – 3.41 (m, 1H), 2.76 – 2.60 (m, 2H), 1.87 – 1.70 (m, 2H), 1.31 (d, J = 6.5 Hz, 3H). **^{13}C NMR** (100 MHz, $CDCl_3$) δ 157.28, 137.26, 133.65, 129.45, 128.67, 128.02, 127.57, 114.98, 70.16, 57.22, 38.19, 31.53, 19.60. **MS** (**EI**, m/z, %): 363 (M^+, 5.43), 91 (100.00); **HRMS** (**EI**): Calcd. For $C_{17}H_{19}N_3O^+$: 281.1523; Found: 281.1527.

1-(4-Azidopentyl)naphthalene (3s)

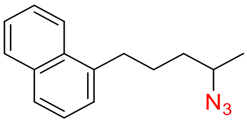

Following the general procedure for secondary alcohols (Method B), prepared from 5-(naphthalen-1-yl)pentan-2-ol (**2s**) (42.8 mg, 0.2 mmol, 1.0 equiv), SulfoxFluor (194.6 mg, 0.56 mmol, 2.8 equiv), NaN_3 (26.0 mg, 0.4 mmol, 2.0 equiv), DBU (120 µL, 0.80 mmol, 4.0 equiv) in DMF for 12 hours; eluted with petroleum ether/EtOAc, 15:1 (v/v).

44.1 mg, 92% yield. Light yellow liquid. **IR** (film): 3067, 3046, 2935, 2868, 2102, 1721, 1596, 1510, 1455, 1395, 1379, 1326, 1261, 1184, 1165, 1124, 1104, 1081, 1030, 1015, 968, 948, 906, 862, 798, 790, 778, 733, 692, 646, 557, 423 cm^{-1}. **^{1}H NMR** (400 MHz, $CDCl_3$) δ 8.05 (d, J = 8.3 Hz, 1H), 7.89 – 7.87 (m, 1H), 7.75 (d, J = 8.2 Hz, 1H), 7.57 – 7.48 (m, 2H), 7.44 – 7.40 (m, 1H), 7.35 – 7.33 (m, 1H), 3.49 (h, J = 6.5 Hz, 1H), 3.11 (t, J = 7.7 Hz, 2H), 1.97 – 1.77 (m, 2H), 1.72 – 1.58 (m, 2H), 1.27 (d, J = 6.5 Hz, 3H). **^{13}C NMR** (100 MHz, $CDCl_3$) δ 138.12, 134.04, 131.92, 128.94, 126.84,

126.07, 125.94, 125.63, 125.59, 123.79, 58.04, 36.31, 32.82, 27.32, 19.62. **MS** (**EI**, m/z, %): 239 (M^+, 11.59), 141 (100.00); **HRMS** (**EI**): Calcd. For $C_{15}H_{17}N_3^+$: 239.1417; Found: 239.1431.

4-(2-Azidododecyl)-1,1'-biphenyl (3t)

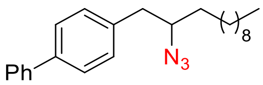

Following the general procedure for secondary alcohols (Method B), prepared from 1-([1,1'-biphenyl]-4-yl)dodecan-2-ol (**2t**) (67.6 mg, 0.2 mmol, 1.0 equiv), SulfoxFluor (194.6 mg, 0.56 mmol, 2.8 equiv), NaN_3 (26.0 mg, 0.4 mmol, 2.0 equiv), DBU (120 μL, 0.80 mmol, 4.0 equiv) in DMF for 12 hours; eluted with petroleum ether/EtOAc, 10:1 (v/v).

68.0 mg, 94% yield. Colorless liquid. **IR** (film): 3057, 3028, 2924, 2853, 2101, 1601, 1564, 1520, 1487, 1466, 1409, 1377, 1341, 1258, 1123, 1075, 1040, 1008, 996, 909, 843, 819, 761, 735, 697, 650, 607, 557, 507 cm^{-1}. **^{1}H NMR** (400 MHz, $CDCl_3$) δ 7.63 (d, J = 7.3 Hz, 2H), 7.59 (d, J = 8.0 Hz, 2H), 7.47 (t, J = 7.7 Hz, 2H), 7.37 (t, J = 7.4 Hz, 1H), 7.32 (d, J = 8.0 Hz, 2H), 3.57 (p, J = 6.9 Hz, 1H), 2.93 – 2.84 (m, 2H), 1.77 – 1.50 (m, 3H), 1.47 – 1.31 (m, 15H), 0.93 (t, J = 6.7 Hz, 3H). **^{13}C NMR** (100 MHz, $CDCl_3$) δ 140.96, 139.70, 137.13, 129.79, 128.86, 127.34, 127.30, 127.13, 64.27, 40.66, 34.20, 32.05, 29.73, 29.70, 29.65, 29.52, 29.47, 26.29, 22.83, 14.26. **MS** (**EI**, m/z, %): 363 (M^+, 5.43), 167 (100.00); **HRMS** (**EI**): Calcd. For $C_{24}H_{33}N_3^+$: 363.2669; Found: 363.2666.

***tert*-Butyl 3-azido-1-oxa-8-azaspiro[4.5]decane-8-carboxylate (3u)**

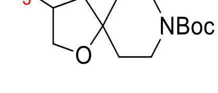

Following the general procedure for secondary alcohols (Method B), prepared from *tert*-butyl 3-hydroxy-1-oxa-8-azaspiro[4.5]decane-8-carboxylate (**2u**) (51.5 mg, 0.2 mmol, 1.0 equiv), SulfoxFluor (194.6 mg, 0.56 mmol, 2.8 equiv), NaN_3 (26.0 mg, 0.4 mmol, 2.0 equiv), DBU (120 μL, 0.80 mmol, 4.0 equiv) in DMF for 12 hours; eluted with petroleum ether/EtOAc, 10:1 (v/v).

48.5 mg, 86% yield. White solid. **M.p.**: 53-55 ºC. **IR** (film): 2975, 2940, 2869, 2104, 1693, 1478, 1467, 1422, 1392, 1366, 1321, 1245, 1175, 1149, 1109, 1069, 1022, 993, 967, 955, 911, 862, 825, 769, 734, 647, 555, 495 cm^{-1}. **^{1}H NMR** (400 MHz, $CDCl_3$) δ 4.17 – 4.13 (m, 1H), 3.96 – 3.92 (m, 1H), 3.83 – 3.80 (m, 1H), 3.68 – 3.54 (m, 2H), 3.31 – 3.22 (m, 2H), 2.03 – 1.97 (m, 1H), 1.87 – 1.83 (m, 1H), 1.79 – 1.75 (m, 1H), 1.63 – 1.48 (m, 3H), 1.43 (s, 9H). **^{13}C NMR** (100 MHz, $CDCl_3$) δ 154.84, 80.49, 79.56, 70.89, 61.51, 43.12, 41.02, 36.55, 36.18, 28.54. **HRMS (ESI)**: Calcd. For $C_{13}H_{22}O_3N_4Na^+$: 305.1584; Found: 305.1583.

(*R*)-3-azido-1-((4-bromophenyl)sulfonyl)pyrrolidine (3v)

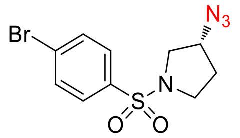

Following the general procedure for secondary alcohols (Method B), prepared from (*S*)-1-((4-bromophenyl)sulfonyl)pyrrolidin-3-ol (**2v**) (61.0 mg, 0.2 mmol, 1.0 equiv), SulfoxFluor (194.6 mg, 0.56 mmol, 2.8 equiv), NaN_3 (26.0 mg, 0.4 mmol, 2.0 equiv), DBU (120 μL, 0.80 mmol, 4.0 equiv) in DMF for 12 hours; eluted with petroleum ether/EtOAc, 4:1 (v/v).

63.1 mg, 96% yield. White solid. **M.p.**: 75-77 ºC. $[\alpha]_D^{25.8}$ = +9.57º (c = 1.20, $CHCl_3$). **IR** (film): 3089, 2954, 2885, 2101, 1574, 1471, 1442, 1389, 1348, 1275, 1224, 1166, 1092, 1068, 1036, 1008, 952, 914, 825, 787, 739, 705, 613, 578, 526, 491, 421 cm^{-1}. **^{1}H NMR** (400 MHz, $CDCl_3$) δ 7.70 – 7.66 (m, 4H), 4.11 – 4.07 (m, 1H), 3.49 – 3.40 (m, 2H), 3.28 – 3.21 (m, 2H), 2.08 – 1.98 (m, 1H), 1.94 – 1.88 (m, 1H). **^{13}C NMR** (100 MHz, $CDCl_3$) δ 135.78, 132.52, 128.98, 128.06, 59.99, 52.92, 46.09, 31.32. **HRMS (ESI)**: Calcd. For $C_{10}H_{12}O_2N_4BrS^+$: 330.9859; Found: 330.9858.

HPLC Traces for Measuring Enantiomeric Excess: A racemic sample of compound **3v** was obtained through deoxy-azidation of racemic alcohol **2v** with SulfoxFluor. The racemic and optically active **3v** were analyzed with HPLC (Chiralcel IE-3, hexane/IPA = 80/20 (v/v), λ = 214 nm, 0.70 mL/min) to determine the retention time and enantiomeric excesses. For (*R*)-**3v**, *e.e.* = 88.2%. [*e.s.*% =

88.2/87.9*100% > 99.9%]

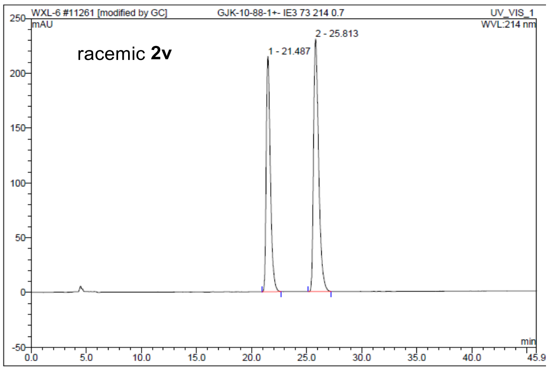

No.	Ret.Time min	Peak Name	Height mAU	Area mAU*min	Rel.Area %	Amount	Type
1	21.49	n.a.	214.799	94.086	43.08	n.a.	BMB
2	25.81	n.a.	230.558	124.317	56.92	n.a.	BMB
Total:			445.356	218.403	100.00	0.000	

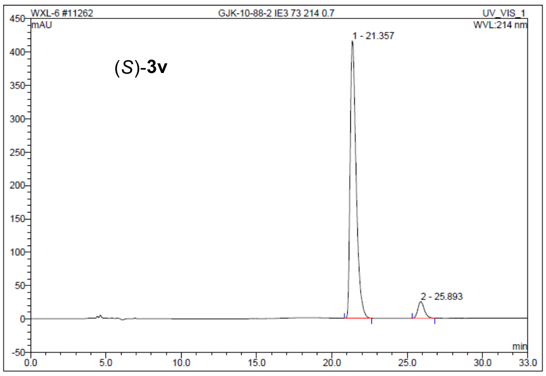

No.	Ret.Time min	Peak Name	Height mAU	Area mAU*min	Rel.Area %	Amount	Type
1	21.36	n.a.	416.157	192.437	93.93	n.a.	BMB
2	25.89	n.a.	25.032	12.425	6.07	n.a.	BMB
Total:			441.189	204.862	100.00	0.000	

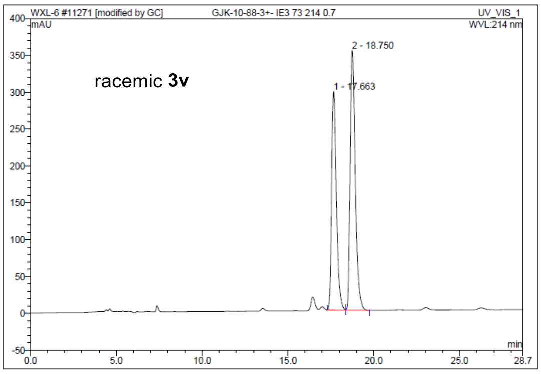

No.	Ret.Time min	Peak Name	Height mAU	Area mAU*min	Rel.Area %	Amount	Type
1	17.66	n.a.	296.864	96.077	43.82	n.a.	BM *
2	18.75	n.a.	352.874	123.180	56.18	n.a.	MB*
Total:			649.739	219.257	100.00	0.000	

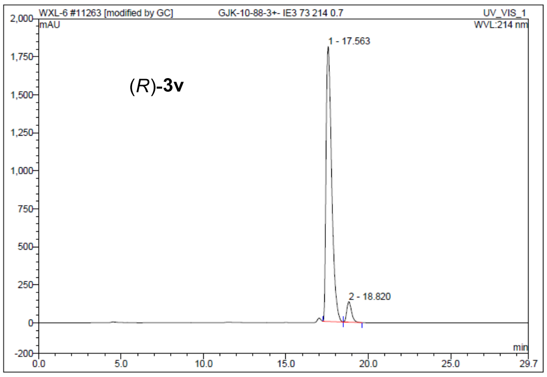

No.	Ret.Time min	Peak Name	Height mAU	Area mAU*min	Rel.Area %	Amount	Type
1	17.56	n.a.	1808.796	716.668	94.09	n.a.	BM *
2	18.82	n.a.	134.508	44.995	5.91	n.a.	MB*
Total:			1943.304	761.663	100.00	0.000	

4-(3-Azidopyrrolidine-1-carbonyl)benzaldehyde (3w)

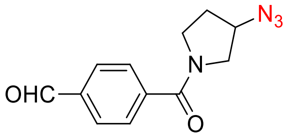

Following the general procedure for secondary alcohols (Method B), prepared from 4-(3-hydroxypyrrolidine-1-carbonyl)benzaldehyde (**2w**) (43.8 mg, 0.2 mmol, 1.0 equiv), SulfoxFluor (194.6 mg, 0.56 mmol, 2.8 equiv), NaN_3 (26.0 mg, 0.4 mmol, 2.0 equiv), DBU (120 μL, 0.80 mmol, 4.0 equiv) in DMF for 12 hours; eluted with petroleum ether/EtOAc, 2:1 (v/v).

40.0 mg, 82% yield. Colorless liquid. **IR** (film): 3053, 2953, 2886, 2738, 2101, 1704, 1633, 1569, 1508, 1428, 1320, 1385, 1302, 1265, 1207, 1169, 1147, 1104, 1058, 1017, 961, 910, 844, 829, 756, 733, 670, 646, 620, 594, 558, 504, 423 cm^{-1}. **^{1}H NMR**

(400 MHz, $CDCl_3$) [mixture of two rotamers] δ 10.04 (s, 1H), 7.92 (d, J = 8.0 Hz, 2H), 7.65 (dd, J = 12.4, 8.0 Hz, 2H), 4.34 – 4.13 (m, 1H), 3.84 – 3.74 (m, 2H), 3.62 – 3.56 (m, 1H), 3.48 – 3.36 (m, 1H), 2.27 – 2.01 (m, 2H). **^{13}C NMR** (100 MHz, $CDCl_3$) [mixture of two rotamers, minor rotamer denoted by *] δ 191.59, 168.73, 141.95*, 141.82, 137.36, 129.94*, 129.86, 127.90, 127.79*, 60.43*, 59.35, 54.12*, 51.57, 47.20, 44.33*, 32.09, 29.99*. **HRMS (ESI)**: Calcd. For $C_{12}H_{13}O_2N_4^+$: 245.1033; Found: 245.1032.

3-Azido-1-(thiophen-2-ylsulfonyl)pyrrolidine (3x)

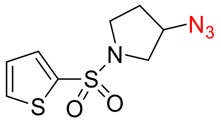

Following the general procedure for secondary alcohols (Method B), prepared from 1-(thiophen-2-ylsulfonyl)pyrrolidin-3-ol (**2x**) (46.8 mg, 0.2 mmol, 1.0 equiv), SulfoxFluor (194.6 mg, 0.56 mmol, 2.8 equiv), NaN_3 (26.0 mg, 0.4 mmol, 2.0 equiv),

DBU (120 μL, 0.80 mmol, 4.0 equiv) in DMF for 12 hours; eluted with petroleum ether/EtOAc, 4:1 (v/v).

46.5 mg, 90% yield. Colorless liquid. **IR** (film): 3098, 2954, 2883, 2101, 1506, 1464, 1442, 1404, 1351, 1267, 1225, 1155, 1096, 1023, 946, 914, 855, 787, 727, 667, 606, 578, 559, 519, 484, 423 cm^{-1}. **^{1}H NMR** (400 MHz, $CDCl_3$) δ 7.63 – 7.60 (m, 2H), 7.16 – 7.14 (m, 1H), 4.12 – 4.08 (m, 1H), 3.53 – 3.44 (m, 2H), 3.37 – 3.31 (m, 2H), 2.06 – 1.97 (m, 1H), 1.95 – 1.88 (m, 1H). **^{13}C NMR** (100 MHz, $CDCl_3$) δ 136.46, 132.45, 132.20, 127.75, 60.06, 53.02, 46.39, 31.36. **HRMS (ESI)**: Calcd. For $C_8H_{11}O_2N_4S_2^+$: 259.0318; Found: 259.0319.

4-((3-Azidopyrrolidin-1-yl)sulfonyl)-3,5-dimethylisoxazole (3y)

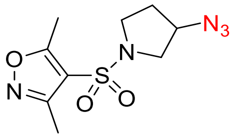

Following the general procedure for secondary alcohols (Method B), prepared from 1-((3,5-dimethylisoxazol-4-yl)sulfonyl)pyrrolidin-3-ol (**2y**) (49.2 mg, 0.2 mmol,

1.0 equiv), SulfoxFluor (194.6 mg, 0.56 mmol, 2.8 equiv), NaN_3 (26.0 mg, 0.4 mmol, 2.0 equiv), DBU (120 µL, 0.80 mmol, 4.0 equiv) in DMF for 12 hours; eluted with petroleum ether/EtOAc, 3:1 (v/v).

50.3 mg, 93% yield. White solid. **M.p.**: 56-59 ºC. **IR** (film): 2956, 2103, 1590, 1481, 1441, 1408, 1372, 1343, 1264, 1214, 1178, 1125, 1090, 1039, 983, 914, 883, 788, 757, 734, 686, 644, 584, 570, 517, 425 cm^{-1}. **^{1}H NMR** (400 MHz, $CDCl_3$) δ 4.22 – 4.18 (m, 1H), 3.51 – 3.43 (m, 2H), 3.33 – 3.26 (m, 2H), 2.64 (s, 3H), 2.41 (s, 3H), 2.21 – 2.11 (m, 1H), 2.08 – 2.01 (m, 1H). **^{13}C NMR** (100 MHz, $CDCl_3$) δ 173.69, 157.90, 114.70, 60.10, 52.60, 45.43, 31.43, 13.04, 11.37. **HRMS (DART)**: Calcd. For $C_9H_{14}O_3N_5S^+$: 272.0812; Found: 272.0813.

***tert*-Butyl 3-azidopyrrolidine-1-carboxylate (3z)**

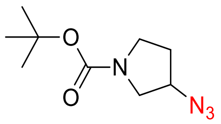

Following the general procedure for secondary alcohols (Method B), prepared from 1-boc-3-hydroxypyrrolidine (**2z**) (37.4 mg, 0.2 mmol, 1.0 equiv), SulfoxFluor (194.6 mg, 0.56 mmol, 2.8 equiv), NaN_3 (26.0 mg, 0.4 mmol, 2.0 equiv), DBU (120 µL, 0.80 mmol, 4.0 equiv) in DMF for 12 hours; eluted with petroleum ether/EtOAc, 10:1 (v/v).

38.6 mg, 91% yield. Colorless liquid. **IR** (film): 2977, 2933, 2885, 2103, 1697, 1603, 1543, 1479, 1455, 1405, 1366, 1350, 1320, 1262, 1167, 1115, 1056, 1031, 987, 961, 921, 878, 853, 772, 733, 647, 621, 551, 462, 424 cm^{-1}. **^{1}H NMR** (400 MHz, $CDCl_3$) [mixture of two rotamers] δ 4.13 – 4.09 (m, 1H), 3.51 – 3.33 (m, 4H), 2.09 – 1.93 (m, 2H), 1.44 (s, 9H). **^{13}C NMR** (100 MHz, $CDCl_3$) [mixture of two rotamers] δ 154.41 [154.35], 79.78, 60.51 [59.82], 51.07 [50.81], 44.06 [43.71], 31.44 [30.65], 28.56. **HRMS (ESI)**: Calcd. For $C_9H_{16}O_2N_4Na^+$: 235.1165; Found: 235.1165. The NMR data are in agreement with the literature.[23]

Benzyl 3-azidopyrrolidine-1-carboxylate (3aa)

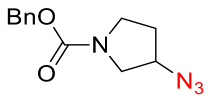

Following the general procedure for secondary alcohols (Method B), prepared from benzyl 3-hydroxypyrrolidine-1-carboxylate (**2aa**) (44.2 mg, 0.2 mmol, 1.0 equiv), SulfoxFluor (194.6 mg, 0.56 mmol, 2.8 equiv), NaN_3 (26.0 mg, 0.4 mmol, 2.0 equiv), DBU (120 μL, 0.80 mmol, 4.0 equiv) in DMF for 12 hours; eluted with petroleum ether/EtOAc, 4:1 (v/v).

42.0 mg, 85% yield. Light yellow liquid. **IR** (film): 3089, 3065, 3033, 2953, 2886, 2101, 1704, 1607, 1586, 1537, 1498, 1417, 1348, 1265, 1210, 1109, 1056, 1029, 993, 969, 914, 878, 811, 768, 750, 698, 621, 605, 554, 449 cm^{-1}. **^{1}H NMR** (400 MHz, $CDCl_3$) [mixture of two rotamers] δ 7.41 – 7.27 (m, 5H), 5.18 – 5.11 (m, 2H), 4.20 – 4.11 (m, 1H), 3.61 – 3.45 (m, 4H), 2.15 – 1.97 (m, 2H). **^{13}C NMR** (100 MHz, $CDCl_3$) [mixture of two rotamers] δ 154.80 [154.72], 136.78 [136.76], 128.58, 128.12, 128.02, 67.07, 60.46 [59.74], 51.36 [50.95], 44.29 [43.92], 31.40 [30.59]. **HRMS (ESI)**: Calcd. For $C_{12}H_{14}O_2N_4Na^+$: 269.1009; Found: 269.1008.

(2*S*,4*S*)-1-*tert*-Butyl 2-methyl 4-azidopyrrolidine-1,2-dicarboxylate (3ab)

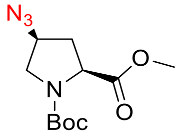

Following the general procedure for secondary alcohols (Method B), prepared from *N*-boc-trans-4-hydroxy-L-proline methyl ester (**2ab**) (49.0 mg, 0.2 mmol, 1.0 equiv), SulfoxFluor (194.6 mg, 0.56 mmol, 2.8 equiv), NaN_3 (26.0 mg, 0.4 mmol, 2.0 equiv), DBU (120 μL, 0.80 mmol, 4.0 equiv) in DMF for 12 hours; eluted with petroleum ether/EtOAc, 4:1 (v/v).

53.6 mg, 99% yield. Light yellow liquid. The *trans*-diastereomer was not detected by ^{1}H NMR. Based on the signal-to-noise ratio, we conclude that the diastereoselectivity is greater than 20:1.

IR (film): 2977, 2955, 2929, 2886, 2104, 1755, 1704, 1478, 1436, 1398, 1366, 1324, 1307, 1260, 1203, 1182, 1161, 1119, 1054, 1032, 992, 952, 915, 893, 865, 843,

770, 737, 624, 567 cm^{-1}. **^{1}H NMR** (400 MHz, $CDCl_3$) [mixture of two rotamers] δ 4.42 – 4.48 (m, 1H), 4.18 – 4.10 (m, 1H), 3.73 – 3.64 (m, 4H), 3.49 – 3.41 (m, 1H), 2.50 – 2.38 (m, 1H), 2.16 – 2.13 (m, 1H), 1.45 – 1.39 (m, 9H). **^{13}C NMR** (100 MHz, $CDCl_3$) [mixture of two rotamers, minor rotamer denoted by *] δ 172.33, 172.02*, 154.02*, 153.51, 80.64, 59.31*, 58.33, 57.81, 57.43*, 52.50*, 52.35, 51.36*, 50.89, 36.10, 35.18*, 28.44*, 28.32. **HRMS (ESI)**: Calcd. For $C_{11}H_{18}O_4N_4Na^+$: 293.1220; Found: 293.1218. The NMR data are in agreement with the literature.[24]

3-Azido-1-tosylpiperidine (3ac)

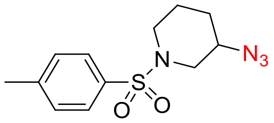

Following the general procedure for secondary alcohols (Method B), prepared from 1-tosylpiperidin-3-ol (**2ac**) (51.0 mg, 0.2 mmol, 1.0 equiv), SulfoxFluor (194.6 mg, 0.56 mmol, 2.8 equiv), NaN_3 (26.0 mg, 0.4 mmol, 2.0 equiv), DBU (120 µL, 0.80 mmol, 4.0 equiv) in DMF for 12 hours; eluted with petroleum ether/EtOAc, 5:1 (v/v).

41.0 mg, 73% yield. Light yellow liquid. **IR** (film): 3067, 3031, 2955, 2929, 2854, 2106, 1597, 1494, 1466, 1444, 1399, 1343, 1306, 1272, 1167, 1132, 1109, 1092, 1056, 1016, 954, 932, 894, 863, 817, 801, 746, 711, 676, 653, 612, 578, 549, 501, 477, 416 cm^{-1}. **^{1}H NMR** (400 MHz, $CDCl_3$) δ 7.63 (d, J = 8.2 Hz, 2H), 7.32 (d, J = 8.0 Hz, 2H), 3.64 – 3.53 (m, 2H), 3.47 – 3.42 (m, 1H), 2.50 – 2.36 (m, 5H), 1.97 – 1.92 (m, 1H), 1.87 – 1.79 (m, 1H), 1.70 – 1.59 (m, 1H), 1.35 – 1.23 (m, 1H). **^{13}C NMR** (100 MHz, $CDCl_3$) δ 143.92, 133.22, 129.88, 127.69, 56.37, 49.91, 45.97, 29.08, 22.88, 21.64. **HRMS (ESI)**: Calcd. For $C_{12}H_{17}O_2N_4S^+$: 281.1067; Found: 281.1066.

3-Azido-1-tosylazetidine (3ad)

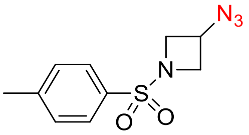

Following the general procedure for secondary alcohols (Method B), prepared from 1-tosylazetidin-3-ol (**2ad**) (45.4 mg, 0.2 mmol, 1.0 equiv), SulfoxFluor (194.6

mg, 0.56 mmol, 2.8 equiv), NaN_3 (26.0 mg, 0.4 mmol, 2.0 equiv), DBU (120 µL, 0.80 mmol, 4.0 equiv) in DMF for 12 hours; eluted with petroleum ether/EtOAc, 4:1 (v/v).

30.0 mg, 60% yield. White solid. **M.p.**: 99-102 ºC. **IR** (film): 3056, 3002, 2959, 2925, 2871, 2126, 1595, 1492, 1471, 1449, 1393, 1377, 1343, 1301, 1293, 1271, 1180, 1160, 1120, 1089, 1059, 1006, 979, 928, 910, 874, 815, 777, 735, 707, 674, 628, 598, 563, 551, 493, 445 cm^{-1}. **^{1}H NMR** (400 MHz, $CDCl_3$) δ 7.74 – 7.71 (m, 2H), 7.39 (d, J = 8.0 Hz, 2H), 4.13 – 4.03 (m, 3H), 3.67 – 3.58 (m, 2H), 2.46 (s, 3H). **^{13}C NMR** (100 MHz, $CDCl_3$) δ 144.77, 130.98, 130.10, 128.49, 56.80, 48.48, 21.76. **HRMS (ESI)**: Calcd. For $C_{10}H_{13}O_2N_4S^+$: 253.0754; Found: 253.0753.

2-(4-Azidopiperidin-1-yl)-5-bromopyrimidine (3ae)

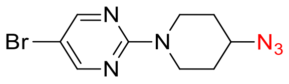

Following the general procedure for secondary alcohols (Method B), prepared from 1-(5-bromopyrimidin-2-yl)piperidin-4-ol (**2ae**) (51.4 mg, 0.2 mmol, 1.0 equiv), SulfoxFluor (194.6 mg, 0.56 mmol, 2.8 equiv), NaN_3 (26.0 mg, 0.4 mmol, 2.0 equiv), DBU (120 µL, 0.80 mmol, 4.0 equiv) in DMF for 12 hours; eluted with petroleum ether/EtOAc, 50:1 (v/v).

50.6 mg, 90% yield. Colorless liquid. **IR** (film): 3158, 3019, 2945, 2856, 2098, 1575, 1525, 1506, 1457, 1395, 1363, 1326, 1300, 1258, 1241, 1203, 1170, 1124, 1117, 1091, 1024, 994, 989, 949, 935, 879, 786, 762, 733, 644, 608, 559, 502, 466 cm^{-1}. **^{1}H NMR** (400 MHz, $CDCl_3$) δ 8.27 (s, 2H), 4.28 (dt, J = 13.4, 4.5 Hz, 2H), 3.70 – 3.64 (m, 1H), 3.41 – 3.43 (m, 2H), 1.97 – 1.90 (m, 2H), 1.61 (ddt, J = 13.6, 9.2, 4.6 Hz, 2H). **^{13}C NMR** (100 MHz, $CDCl_3$) δ 159.79, 158.04, 105.92, 57.93, 41.85, 30.52. **HRMS (ESI)**: Calcd. For $C_9H_{12}N_6Br^+$: 283.0301; Found: 283.0301.

(3*S*,3a*S*,6*S*,6a*R*)-6-Azidohexahydrofuro[3,2-*b*]furan-3-yl acetate (3af)

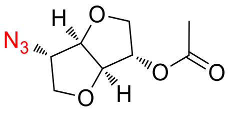

Following the general procedure for secondary alcohols (Method B), prepared

from isosorbide-2-acetate (**2af**) (37.6 mg, 0.2 mmol, 1.0 equiv), SulfoxFluor (194.6 mg, 0.56 mmol, 2.8 equiv), NaN_3 (26.0 mg, 0.4 mmol, 2.0 equiv), DBU (120 µl, 0.80 mmol, 4.0 equiv) in DMF for 12 hours; eluted with petroleum ether/EtOAc, 10:1 (v/v).

39.0 mg, 92% yield. Colorless liquid. The signal for the minor epimer is near the limit of quantitatification; we conclude that the epimer ratio is greater than 20:1.

IR (film): 2957, 2881, 2500, 2105, 1747, 1586, 1467, 1433, 1368, 1343, 1324, 1285, 1232, 1070, 989, 960, 938, 917, 859, 804, 774, 734, 689, 656, 620, 603, 557, 504, 438 cm^{-1}. **^{1}H NMR** (400 MHz, $CDCl_3$) δ 5.17 (d, J = 3.1 Hz, 1H), 4.62 (q, J = 3.8 Hz, 2H), 4.07 – 4.03 (m, 1H), 3.96 – 3.86 (m, 4H), 2.07 (s, 3H). **^{13}C NMR** (100 MHz, $CDCl_3$) δ 169.98, 86.15, 85.51, 77.62, 72.95, 71.68, 65.84, 20.98. **MS** (**EI**, m/z, %): 213 (M^+, 0.28), 115 (100.00); **HRMS (EI)**: Calcd. For $C_8H_{11}N_3O_4^+$: 213.0744; Found: 213.0743.

(3a*R*,4*R*,5*S*,6a*S*)-5-Azido-4-((*E*)-3,3-difluoro-4-phenoxybut-1-en-1-yl)hexahydro-2*H*-cyclopenta[*b*]furan-2-one (3ag)

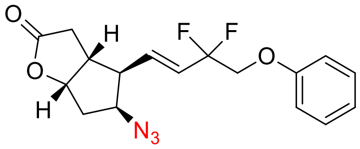

Following the general procedure for secondary alcohols (Method B), prepared from TF-HF (**2ag**) (64.9 mg, 0.2 mmol, 1.0 equiv), SulfoxFluor (194.6 mg, 0.56 mmol, 2.8 equiv), NaN_3 (26.0 mg, 0.4 mmol, 2.0 equiv), DBU (120 µl, 0.80 mmol, 4.0 equiv) in DMF for 12 hours; eluted with petroleum ether/EtOAc, 10:1 (v/v).

64.3 mg, 92% yield. Colorless liquid. The signal for the minor epimer was not detected by ^{19}F NMR and ^{1}H NMR.; we conclude that the epimer ratio is greater than 20:1.

IR (film): 3064, 3042, 2938, 2881, 2106, 1775, 1678, 1598, 1495, 1457, 1419, 1305, 1247, 1215, 1161, 1124, 1078, 1056, 1006, 976, 934, 908, 876, 850, 757, 734, 692, 649, 615, 548, 510 cm^{-1}. **^{1}H NMR** (400 MHz, $CDCl_3$) δ 7.33 – 7.29 (m, 2H), 7.02 (t, J = 7.4 Hz, 1H), 6.92 (d, J = 7.9 Hz, 2H), 6.31 – 6.21 (m, 1H), 5.89 (dt, J =

15.9, 11.0 Hz, 1H), 5.04 (td, *J* = 7.0, 3.6 Hz, 1H), 4.25 – 4.16 (m, 3H), 2.87 – 2.80 (m, 1H), 2.77 – 2.70 (m, 1H), 2.53 – 2.44 (m, 2H), 2.33 – 2.28 (m, 1H), 2.25 – 2.19 (m, 1H). **^{13}C NMR** (100 MHz, $CDCl_3$) δ 175.86, 157.90, 133.28 (t, *J* = 9.1 Hz), 129.76, 126.55 (t, *J* = 25.4 Hz), 122.06, 117.87 (t, *J* = 240.7 Hz), 114.84, 83.21, 69.42 (t, *J* = 35.2 Hz), 66.49, 52.15, 42.48, 39.11, 33.11. **^{19}F NMR** (376 MHz, $CDCl_3$) δ –103.95 (m, 2F). **MS** (**EI**, m/z, %): 349 (M^+, 1.60), 84 (100.00); **HRMS (EI)**: Calcd. For $C_{17}H_{17}N_3O_3F_2^+$: 349.1232; Found: 349.1247.

(3a*R*,4*S*,5*R*,6a*S*)-4-(Azidomethyl)-2-oxohexahydro-2*H*-cyclopenta[*b*]furan-5-yl benzoate (3ah)

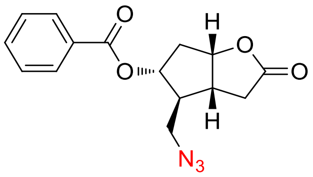

Following the general procedure for primary alcohols (Method A), prepared from (-)-corey lactone benzoate (**2ah**) (55.2 mg, 0.2 mmol, 1.0 equiv), SulfoxFluor (152.9 mg, 0.44 mmol, 2.2 equiv), NaN_3 (52.0 mg, 0.8 mmol, 4.0 equiv), DBU (54 µL, 0.36 mmol, 1.8 equiv) in DMF for 12 hours; eluted with petroleum ether/EtOAc, 8:1 (v/v).

48.8 mg, 81% yield. White solid. **M.p.**: 67-69 °C. **IR** (film): 3064, 3032, 2968, 2933, 2864, 2102, 1771, 1716, 1601, 1584, 1491, 1451, 1419, 1359, 1327, 1314, 1275, 1223, 1178, 1114, 1098, 1071, 1026, 1001, 980, 951, 912, 807, 732, 714, 687, 674, 648, 616, 555, 503, 469 cm^{-1}. **^{1}H NMR** (400 MHz, $CDCl_3$) δ 7.99 – 7.97 (m, 2H), 7.59 – 7.53 (m, 1H), 7.44 (t, *J* = 7.6 Hz, 2H), 5.29 (dt, *J* = 6.4, 3.9 Hz, 1H), 5.08 – 5.04 (m, 1H), 3.50 (qd, *J* = 12.4, 6.1 Hz, 2H), 2.95 – 2.88 (m, 1H), 2.85 – 2.79 (m, 1H), 2.57 – 2.46 (m, 2H), 2.35 – 2.31 (m, 2H). **^{13}C NMR** (100 MHz, $CDCl_3$) δ 176.21, 166.03, 133.50, 129.75, 129.47, 128.63, 84.21, 77.75, 52.48, 52.13, 41.20, 38.26, 35.82. **HRMS (DART)**: Calcd. For $C_{15}H_{16}O_4N_3^+$: 302.1135; Found: 302.1136.

5-((3-Azidopyrrolidin-1-yl)sulfonyl)-*N*,*N*-dimethylnaphthalen-1-amine (3ai)

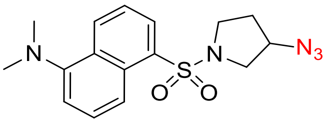

Following the general procedure for secondary alcohols (Method B), prepared from 1-((5-(dimethylamino)naphthalen-1-yl)sulfonyl)pyrrolidin-3-ol (**2ai**) (64.0 mg, 0.2 mmol, 1.0 equiv), SulfoxFluor (194.6 mg, 0.56 mmol, 2.8 equiv), NaN_3 (26.0 mg, 0.4 mmol, 2.0 equiv), DBU (120 µl, 0.80 mmol, 4.0 equiv) in DMF for 12 hours; eluted with petroleum ether/EtOAc, 4:1 (v/v).

65.0 mg, 94% yield. Yellow liquid. **IR** (film): 3080, 3054, 2985, 2944, 2872, 2833, 2789, 2101, 1611, 1587, 1573, 1503, 1479, 1455, 1394, 1325, 1406, 1266, 1230, 1202, 1144, 1094, 1076, 1061, 1046, 945, 913, 838, 792, 733, 683, 629, 595, 568, 541, 500, 462, 423 cm^{-1}. **^{1}H NMR** (400 MHz, $CDCl_3$) δ 8.55 (d, J = 8.5 Hz, 1H), 8.43 (d, J = 8.7 Hz, 1H), 8.22 (d, J = 7.3 Hz, 1H), 7.54 (dt, J = 10.4, 8.0 Hz, 2H), 7.18 (d, J = 7.5 Hz, 1H), 4.13 – 4.09 (m, 1H), 3.60 – 3.56 (m, 1H), 3.54 – 3.49 (m, 1H), 3.44 – 3.37 (m, 2H), 2.87 (s, 6H), 2.13 – 2.04 (m, 1H), 1.97 – 1.90 (m, 1H). **^{13}C NMR** (100 MHz, $CDCl_3$) δ 151.81, 133.77, 130.76, 130.53, 130.15, 129.88, 128.23, 123.26, 119.56, 115.35, 60.16, 52.32, 45.74, 45.49, 31.53. **HRMS (ESI)**: Calcd. For $C_{16}H_{20}O_2N_5S^+$: 346.1332; Found: 346.1331.

1-(2-Azidoethyl)-2-methyl-5-nitro-1*H*-imidazole (3aj)

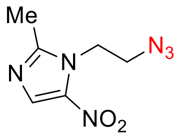

Following the general procedure for primary alcohols (Method A), prepared from metronidazole (**2aj**) (34.2 mg, 0.2 mmol, 1.0 equiv), SulfoxFluor (152.9 mg, 0.44 mmol, 2.2 equiv), NaN_3 (52.0 mg, 0.8 mmol, 4.0 equiv), DBU (54 µL, 0.36 mmol, 1.8 equiv) in DMF for 12 hours; eluted with petroleum ether/EtOAc, 2:1 (v/v).

32.0 mg, 82% yield. Yellow liquid. **IR** (film): 3131, 2930, 2868, 2103, 1634, 1531, 1467, 1427, 1383, 1363, 1293, 1263, 1188, 1148, 1091, 1038, 990, 961, 911, 867, 824, 733, 680, 649, 605, 556, 492, 418 cm^{-1}. **^{1}H NMR** (400 MHz, $CDCl_3$) δ 7.96 (s, 1H), 4.44 – 4.41 (m, 2H), 3.78 – 3.75 (m, 2H), 2.53 (s, 3H). **^{13}C NMR** (100 MHz, $CDCl_3$)

δ 151.42, 138.35, 133.55, 51.04, 45.62, 14.65. **HRMS (DART)**: Calcd. For $C_6H_9O_2N_6^+$: 197.0782; Found: 197.0783. The NMR data are in agreement with the literature.[25]

3-(Azidomethyl)heptan-4-ol (3ak)

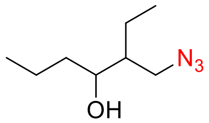

Following the general procedure for primary alcohols (Method A), prepared from 2-ethyl-1,3-hexanediol (**2ak**) (58.4 mg, 0.4 mmol, 1.0 equiv), SulfoxFluor (305.8 mg, 0.88 mmol, 2.2 equiv), NaN_3 (104.0 mg, 1.6 mmol, 4.0 equiv), DBU (108 µL, 0.72 mmol, 1.8 equiv) in DMF for 12 hours; eluted with petroleum ether/EtOAc, 15:1 (v/v).

56.1 mg, 82% yield. Colorless liquid. **IR** (film): 3413, 2962, 2934, 2875, 2098, 1460, 1381, 1346, 1273, 1132, 1117, 1071, 1015, 1005, 960, 910, 848, 812, 776, 735, 648, 556 cm^{-1}. **^{1}H NMR** (400 MHz, $CDCl_3$) [mixture of two diastereoisomers] δ 3.73 – 3.61 (m, 1H), 3.51 – 3.35 (m, 2H), 1.84 (s, 1H), 1.56 – 1.24 (m, 7H), 0.96 – 0.91 (m, 6H). **^{13}C NMR** (100 MHz, $CDCl_3$) [mixture of two diastereoisomers, minor diastereoisomer denoted by *] δ 72.20*, 72.03, 52.57*, 51.40, 45.18, 45.17*, 37.23, 36.23*, 21.73, 19.56, 19.25*, 14.14, 12.19*, 11.61. **HRMS (DART)**: Calcd. For $C_8H_{18}ON_3^+$: 172.1444; Found: 172.1445.

(3*R*,5*R*,8*R*,9*S*,10*S*,13*R*,14*S*,17*R*)-17-((*R*)-5-Azidopentan-2-yl)-10,13-dimethylhexadecahydro-1*H*-cyclopenta[*a*]phenanthren-3-ol (3al)

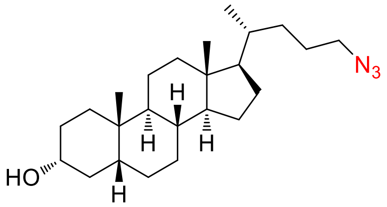

Following the general procedure for primary alcohols (Method A), prepared from (3*R*,5*R*,8*R*,9*S*,10*S*,13*R*,14*S*,17*R*)-17-((*R*)-5-hydroxypentan-2-yl)-10,13-dimethylhexadecahydro-1*H*-cyclopenta[a]phenanthren-3-ol (**2al**) (72.4 mg, 0.2 mmol, 1.0 equiv), SulfoxFluor (152.9 mg, 0.44 mmol, 2.2 equiv), NaN_3 (52.0 mg, 0.8 mmol, 4.0 equiv),

DBU (54 μL, 0.36 mmol, 1.8 equiv) in DMF for 12 hours; eluted with petroleum ether/EtOAc, 5:1 (v/v).

53.8 mg, 70% yield. White solid. **M.p.**: 98-104 ºC. **IR** (film): 3344, 2935, 2864, 2095, 1469, 1449, 1376, 1367, 1291, 1261, 1167, 1109, 1088, 1067, 1034, 1013, 964, 945, 908, 853, 789, 734, 649, 615, 558, 512, 481 cm^{-1}. **^{1}H NMR** (400 MHz, $CDCl_3$) δ 3.65 – 3.57 (m, 1H), 3.27 – 3.16 m, 2H), 1.97 – 1.93 (m, 1H), 1.88 – 1.70 (m, 4H), 1.69 – 1.60 (m, 3H), 1.58 – 1.46 (m, 3H), 1.44 – 1.33 (m, 7H), 1.30 – 1.18 (m, 4H), 1.03 – 1.03 (m, 6H), 1.00 – 0.91 (m, 7H), 0.63 (s, 3H). **^{13}C NMR** (100 MHz, $CDCl_3$) δ 71.95, 56.62, 56.18, 52.08, 42.83, 42.22, 40.56, 40.30, 36.56, 35.96, 35.59, 35.48, 34.69, 33.02, 30.66, 28.41, 27.32, 26.55, 25.65, 24.33, 23.50, 20.94, 18.71, 12.16. **HRMS (DART)**: Calcd. For $C_{24}H_{45}ON_4^+$: 405.3588; Found: 405.3590.

8-Azido-3-phenethyl-1-phenyloctan-3-ol (3am)

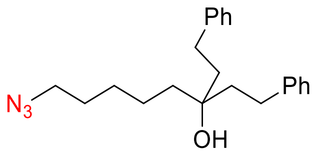

Following the general procedure for primary alcohols (Method A), prepared from 6-phenethyl-8-phenyloctane-1,6-diol (**2am**) (65.2 mg, 0.2 mmol, 1.0 equiv), SulfoxFluor (152.9 mg, 0.44 mmol, 2.2 equiv), NaN_3 (52.0 mg, 0.8 mmol, 4.0 equiv), DBU (54 μL, 0.36 mmol, 1.8 equiv) in DMF for 12 hours; eluted with petroleum ether/EtOAc, 5:1 (v/v).

52.5 mg, 75% yield. Colorless liquid. **IR** (film): 3578, 3461, 3084, 3062, 3026, 2939, 2862, 2096, 1602, 1496, 1454, 1378, 1349, 1259, 1178, 1156, 1109, 1048, 1030, 966, 909, 841, 734, 699, 648, 556, 517, 493 cm^{-1}. **^{1}H NMR** (400 MHz, $CDCl_3$) δ 7.35 – 7.31 (m, 4H), 7.24 – 7.20 (m, 6H), 3.30 (t, J = 6.9 Hz, 2H), 2.72 – 2.68 (m, 4H), 1.87 – 1.83 (m, 4H), 1.69 – 1.59 (m, 4H), 1.48 – 1.33 (m, 5H). **^{13}C NMR** (100 MHz, $CDCl_3$) δ 142.43, 128.59, 128.42, 125.99, 74.32, 51.51, 41.33, 39.17, 30.15, 28.98, 27.44, 23.31. **HRMS (ESI)**: Calcd. For $C_{22}H_{29}ON_3Na^+$: 374.2203; Found: 374.2202.

(3a*S*,4*R*,5*S*,6*S*,8*R*,9*R*,9a*R*,12*R*)-5-Hydroxy-4,6,9,12-tetramethyl-1-oxo-6-vinylde-

cahydro-3a,9-propanocyclopenta[8]annulen-8-yl 2-azidoacetate (3an)

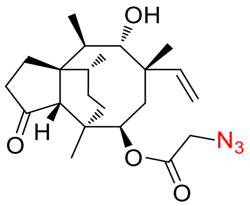

Following the general procedure for primary alcohols (Method A), prepared from pleuromutilin (**2an**) (84.1 mg, 0.2 mmol, 1.0 equiv, 90% wt), SulfoxFluor (152.9 mg, 0.44 mmol, 2.2 equiv), NaN_3 (52.0 mg, 0.8 mmol, 4.0 equiv), DBU (54 μL, 0.36 mmol, 1.8 equiv) in DMF for 12 hours; eluted with petroleum ether/EtOAc, 1:1 (v/v).

64.2 mg, 80% yield. Light yellow solid. **M.p.**: 128-132 °C. **IR** (film): 3064, 3032, 2968, 2933, 2864, 2102, 1771, 1716, 1601, 1584, 1491, 1451, 1419, 1359, 1327, 1314, 1275, 1223, 1178, 1114, 1098, 1071, 1226, 1001, 980, 951, 912, 807, 733, 714, 687, 674, 748, 616, 555, 503, 469 cm^{-1}. **^{1}H NMR** (400 MHz, $CDCl_3$) δ 6.46 (dd, J = 17.4, 11.0 Hz, 1H), 5.83 (d, J = 8.5 Hz, 1H), 5.34 (dd, J = 11.0, 1.2 Hz, 1H), 5.19 (dd, J = 17.4, 1.3 Hz, 1H), 3.75 (s, 2H), 3.36 – 3.32 (m, 1H), 2.32 (p, J = 7.2 Hz, 1H), 2.26 – 2.13 (m, 2H), 2.10 – 1.94 (m, 2H), 1.76 (dq, J = 14.5, 2.7 Hz, 1H), 1.71 – 1.62 (m, 2H), 1.59 – 1.42 (m, 6H), 1.41 – 1.34 (m, 1H), 1.33 – 1.27 (m, 1H), 1.15 – 1.06 (m, 4H), 0.86 (d, J = 7.0 Hz, 3H), 0.70 (d, J = 7.0 Hz, 3H). **^{13}C NMR** (100 MHz, $CDCl_3$) δ 216.87, 167.25, 138.86, 117.52, 74.64, 70.25, 58.17, 51.17, 45.51, 44.91, 44.05, 41.91, 36.71, 36.14, 34.49, 30.46, 26.88, 26.45, 24.91, 16.77, 14.91, 11.59. **HRMS (ESI)**: Calcd. For $C_{22}H_{33}O_4N_3Na^+$: 426.2363; Found: 426.2361.

7-(3-Azidopropoxy)-3-(4-methoxyphenyl)-4*H*-chromen-4-one (3ao)

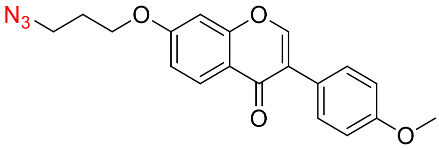

Following the general procedure for primary alcohols (Method A), prepared from 7-(3-hydroxypropoxy)-3-(4-methoxyphenyl)-4*H*-chromen-4-one (**2ao**) (65.2 mg, 0.2 mmol, 1.0 equiv), SulfoxFluor (152.9 mg, 0.44 mmol, 2.2 equiv), NaN_3 (52.0 mg, 0.8 mmol, 4.0 equiv), DBU (54 μL, 0.36 mmol, 1.8 equiv) in DMF for 12 hours; eluted with petroleum ether/EtOAc, 2:1 (v/v).

64.0 mg, 91% yield. White solid. **M.p.**: 111-114 °C. **IR** (film): 3084, 3058, 3006, 2959, 2929, 2881, 2842, 2091, 1621, 1574, 1564, 1513, 1497, 1471, 1444, 1377, 1324, 1289, 1252, 1202, 1180, 1143, 1115, 1097, 1051, 1023, 907, 884, 852, 830, 812, 783, 735, 698, 642, 616, 629, 538, 493, 465 cm^{-1}. **^{1}H NMR** (400 MHz, $CDCl_3$) δ 8.19 (d, J = 8.9 Hz, 1H), 7.89 (s, 1H), 7.49 (d, J = 8.8 Hz, 2H), 6.98 – 6.94 (m, 3H), 6.83 (d, J = 2.3 Hz, 1H), 4.12 (t, J = 5.9 Hz, 2H), 3.82 (s, 3H), 3.53 (t, J = 6.5 Hz, 2H), 2.09 (p, J = 6.2 Hz, 2H). **^{13}C NMR** (100 MHz, $CDCl_3$) δ 175.82, 162.99, 159.62, 157.89, 152.14, 130.16, 127.87, 124.88, 124.25, 118.60, 114.74, 114.00, 100.76, 65.24, 55.38, 48.09, 28.60. **HRMS (ESI)**: Calcd. For $C_{19}H_{18}O_4N_3^+$: 352.1292; Found: 352.1291.

1-(5-Azidohexyl)-3,7-dimethyl-1H-purine-2,6(3*H*,7*H*)-dione (3ap)

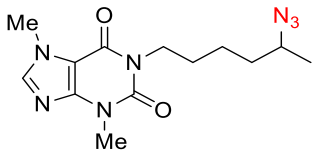

Following the general procedure for secondary alcohols (Method B), prepared from (+/–)-lisofylline (**2ap**) (44.8 mg, 0.16 mmol, 1.0 equiv), SulfoxFluor (155.7 mg, 0.448 mmol, 2.8 equiv), NaN_3 (20.8 mg, 0.32 mmol, 2.0 equiv), DBU (96 μL, 0.64 mmol, 4.0 equiv) in DMF for 12 hours; eluted with petroleum ether/EtOAc, 1:2 (v/v).

37.0 mg, 76% yield. Colorless liquid. **IR** (film): 3114, 2943, 2864, 2101, 1705, 1659, 1605, 1550, 1487, 1457, 1434, 1414, 1380, 1358, 1325, 1286, 1235, 1187, 1153, 1087, 1058, 1033, 1011, 985, 917, 857, 810, 764, 733, 648, 612, 558, 516, 459 cm^{-1}. **^{1}H NMR** (400 MHz, $CDCl_3$) δ 7.49 (s, 1H), 4.00 – 3.97 (m, 5H), 3.55 (s, 3H), 3.46 – 3.37 (m, 1H), 1.69 – 1.34 (m, 6H), 1.23 (d, J = 6.5 Hz, 3H). **^{13}C NMR** (100 MHz, $CDCl_3$) δ 155.36, 151.56, 148.85, 141.53, 107.75, 57.93, 41.18, 35.92, 33.68, 29.78, 27.81, 23.56, 19.53. **HRMS (DART)**: Calcd. For $C_{13}H_{20}O_2N_7^+$: 306.1673; Found: 306.1674.

(8*R*,9*S*,13*S*,14*S*)-3-(3-Azidopropoxy)-13-methyl-7,8,9,11,12,13,15,16-octahydro-6*H*-cyclopenta[*a*]phenanthren-17(14*H*)-one (3aq)

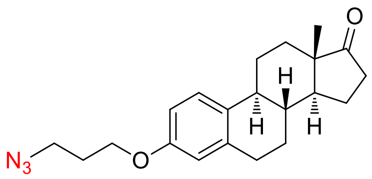

Following the general procedure for primary alcohols (Method A), prepared from (8*R*,9*S*,13*S*,14*S*)-3-(3-hydroxypropoxy)-13-methyl-7,8,9,11,12,13,15,16-octahydro-6*H*-cyclopenta[*a*]phenanthren-17(14*H*)-one (**2aq**) (65.6 mg, 0.2 mmol, 1.0 equiv), SulfoxFluor (152.9 mg, 0.44 mmol, 2.2 equiv), NaN_3 (52.0 mg, 0.8 mmol, 4.0 equiv), DBU (54 μL, 0.36 mmol, 1.8 equiv) in DMF for 12 hours; eluted with petroleum ether/EtOAc, 8:1 (v/v).

60.1 mg, 85% yield. White solid. **M.p.**: 76-77 ºC. **IR** (film): 2931, 2872, 2250, 2098, 1736, 1609, 1574, 1499, 1454, 1435, 1406, 1374, 1342, 1281, 1255, 1235, 1214, 1188, 1164, 1136, 1101, 1083, 1056, 1007, 964, 910, 873, 845, 819, 787, 734, 648, 581, 491, 445 cm^{-1}. **^{1}H NMR** (400 MHz, $CDCl_3$) δ 7.20 (d, *J* = 8.6 Hz, 1H), 6.72 (dd, *J* = 8.6, 2.7 Hz, 1H), 6.65 (d, *J* = 2.5 Hz, 1H), 4.03 (t, *J* = 5.9 Hz, 2H), 3.51 (t, *J* = 6.6 Hz, 2H), 2.96 – 2.85 (m, 2H), 2.54 – 2.47 (m, 1H), 2.44 – 2.34 (m, 1H), 2.27 – 2.22 (m, 1H), 2.19 – 2.12 (m, 1H), 2.10 – 1.98 (m, 4H), 1.97 – 1.91 (m, 1H), 1.69 – 1.56 (m, 2H), 1.55 – 1.53 (m, 1H), 1.50 (s, 1H), 1.48 – 1.38 (m, 2H), 0.91 (s, 3H). **^{13}C NMR** (100 MHz, $CDCl_3$) δ 220.91, 156.72, 137.86, 132.36, 126.42, 114.58, 112.16, 64.48, 50.44, 48.33, 48.04, 44.02, 38.40, 35.91, 31.63, 29.70, 28.89, 26.59, 25.98, 21.64, 13.90. **HRMS (DART)**: Calcd. For $C_{21}H_{28}O_2N_3^+$: 354.2176; Found: 356.2177.

2-(3-Azidopropyl)-4,5-diphenyloxazole (3ar)

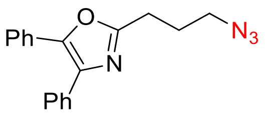

Following the general procedure for primary alcohols (Method A), prepared from 3-(4,5-diphenyloxazol-2-yl)propan-1-ol (**2ar**) (55.8 mg, 0.2 mmol, 1.0 equiv), SulfoxFluor (152.9 mg, 0.44 mmol, 2.2 equiv), NaN_3 (52.0 mg, 0.8 mmol, 4.0 equiv), DBU (54 μL, 0.36 mmol, 1.8 equiv) in DMF for 12 hours; eluted with petroleum ether/EtOAc, 10:1 (v/v).

52.5 mg, 86% yield. Colorless liquid. **IR** (film): 3061, 3041, 2932, 2872, 2099,

1605, 1581, 1570, 1502, 1445, 1350, 1287, 1262, 1220, 1178, 1162, 1143, 1059, 1026, 1001, 963, 977, 909, 845, 764, 733, 694, 674, 649, 586, 557, 525, 467 cm^{-1}. **^{1}H NMR** (400 MHz, $CDCl_3$) δ 7.67 – 7.64 (m, 2H), 7.61 – 7.58 (m, 2H), 7.41 – 7.30 (m, 6H), 3.48 (t, J = 6.7 Hz, 2H), 2.97 (t, J = 7.4 Hz, 2H), 2.16 (p, J = 7.0 Hz, 2H). **^{13}C NMR** (101 MHz, $CDCl_3$) δ 162.26, 145.49, 135.24, 132.54, 129.06, 128.75, 128.67, 128.56, 128.18, 127.99, 126.56, 50.69, 26.45, 25.39. **HRMS (DART)**: Calcd. For $C_{18}H_{17}ON_4^+$: 305.1397; Found: 305.1397. The NMR data are in agreement with the literature.[26]

(2*R*,3*R*,4*R*,5*R*)-2-(Azidomethyl)-5-(6-(*N*-benzoylbenzamido)-9*H*-purin-9-yl)tetrahydrofuran-3,4-diyl dibenzoate (3as)

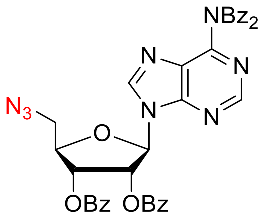

Following the general procedure for primary alcohols (Method A), prepared from N^6,N^6,$O^{2'}$,$O^{3'}$-tetrabenzoyladenosine (**2as**) (136.6 mg, 0.2 mmol, 1.0 equiv), SulfoxFluor (152.9 mg, 0.44 mmol, 2.2 equiv), NaN_3 (52.0 mg, 0.8 mmol, 4.0 equiv), DBU (54 μL, 0.36 mmol, 1.8 equiv) in DMF for 12 hours; eluted with petroleum ether/EtOAc, 2:1 (v/v).

45.0 mg, 32% yield. Colorless liquid. **IR** (film): 3097, 3065, 3034, 2927, 2855, 2253, 2107, 1730, 1600, 1580, 1493, 1450, 1421, 1410, 1339, 1274, 1236, 1123, 1093, 1071, 1026, 1002, 990, 908, 799, 870, 832, 787, 771, 731, 648, 616, 552 cm^{-1}. **^{1}H NMR** (400 MHz, $CDCl_3$) δ 8.70 (s, 1H), 8.43 (s, 1H), 7.98 – 7.92 (m, 4H), 7.87 – 7.85 (m, 4H), 7.60 – 7.54 (m, 2H), 7.49 (t, J = 7.4 Hz, 2H), 7.38 (p, J = 7.5 Hz, 8H), 6.52 (d, J = 5.3 Hz, 1H), 6.27 (t, J = 5.5 Hz, 1H), 6.04 – 6.01 (m, 1H), 4.62 (q, J = 4.1 Hz, 1H), 3.92 – 3.83 (m, 2H). **^{13}C NMR** (100 MHz, $CDCl_3$) δ 172.32, 165.44, 165.11, 152.87, 152.63, 152.30, 143.50, 134.05, 134.00, 133.95, 133.16, 129.98, 129.90, 129.59, 128.89, 128.70, 128.68, 128.67, 128.39, 127.87, 86.99, 82.06, 74.10, 71.87, 52.01. **HRMS (ESI)**: Calcd. For $C_{38}H_{29}O_7N_8^+$: 709.2154; Found: 709.2153.

(3-(2-Azidoethyl)-5-methoxy-2-methyl-1*H*-indol-1-yl)(4-chlorophenyl)methanone (3at)

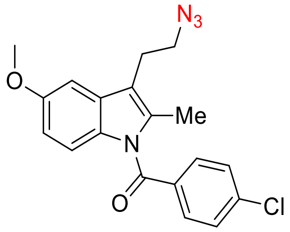

Following the general procedure for primary alcohols (Method A), prepared from (4-chlorophenyl)(3-(2-hydroxyethyl)-5-methoxy-2-methyl-1*H*-indol-1-yl)methanone (**2at**) (68.6 mg, 0.2 mmol, 1.0 equiv), SulfoxFluor (152.9 mg, 0.44 mmol, 2.2 equiv), NaN_3 (52.0 mg, 0.8 mmol, 4.0 equiv), DBU (54 μL, 0.36 mmol, 1.8 equiv) in DMF for 12 hours; eluted with petroleum ether/EtOAc, 10:1 (v/v).

62.9 mg, 85% yield. Yellow liquid. **IR** (film): 3006, 2929, 2881, 2834, 2099, 1682, 1591, 1477, 1456, 1436, 1400, 1361, 1316, 1261, 1224, 1179, 1150, 1089, 1053, 1035, 1015, 955, 910, 879, 840, 804, 755, 734, 690, 649, 614, 552, 482, 432 cm^{-1}. **^{1}H NMR** (400 MHz, $CDCl_3$) δ 7.67 – 7.64 (m, 2H), 7.48 – 7.435 (m, 2H), 6.92 – 6.88 (m, 2H), 6.68 (dd, J = 9.0, 2.5 Hz, 1H), 3.85 (s, 3H), 3.51 (t, J = 7.0 Hz, 2H), 2.96 (t, J = 7.1 Hz, 2H), 2.38 (s, 3H). **^{13}C NMR** (100 MHz, $CDCl_3$) δ 168.37, 156.10, 139.30, 135.58, 134.06, 131.24, 131.10, 130.63, 129.21, 115.62, 115.21, 111.41, 101.11, 55.85, 50.91, 24.30, 13.31. **HRMS (ESI)**: Calcd. For $C_{19}H_{18}O_2N_4Cl^+$: 369.1113; Found: 369.1113. The NMR data are in agreement with the literature.[27]

3-(2-Azidoethoxy)-1-benzyl-1*H*-indazole (3au)

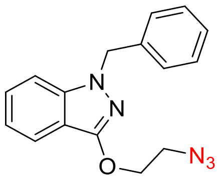

Following the general procedure for primary alcohols (Method A), prepared from 2-((1-benzyl-1*H*-indazol-3-yl)oxy)ethanol (**2au**) (53.6 mg, 0.2 mmol, 1.0 equiv), SulfoxFluor (152.9 mg, 0.44 mmol, 2.2 equiv), NaN_3 (52.0 mg, 0.8 mmol, 4.0 equiv), DBU (54 μL, 0.36 mmol, 1.8 equiv) in DMF for 12 hours; eluted with petroleum

ether/EtOAc, 4:1 (v/v).

48.9 mg, 83% yield. Light yellow liquid. **IR** (film): 3093, 3064, 3031, 2936, 2105, 1618, 1581, 1530, 1496, 1454, 1445, 1434, 1365, 1343, 1301, 1257, 1223, 1188, 1145, 1104, 1077, 1051, 1029, 1004, 959, 908, 841, 821, 769, 740, 702, 651, 588, 554, 454, 428 cm^{-1}. **^{1}H NMR** (400 MHz, $CDCl_3$) δ 7.75 – 7.72 (m, 1H), 7.38 – 7.27 (m, 4H), 7.23 – 7.19 (m, 3H), 7.11 – 7.07 (m, 1H), 5.43 (s, 2H), 4.63 – 4.61 (m, 2H), 3.70 – 3.67 (m, 2H). **^{13}C NMR** (100 MHz, $CDCl_3$) δ 155.36, 141.73, 137.44, 128.71, 127.66, 127.64, 127.08, 120.20, 119.53, 112.92, 108.96, 67.96, 52.45, 50.29. **HRMS (DART)**: Calcd. For $C_{16}H_{16}ON_5^+$: 294.1349; Found: 294.1350.

(2*R*,3*R*,4*S*,5*R*,6*R*)-2-Azido-3,4,5-tris(benzyloxy)-6-((benzyloxy)methyl)tetrahydro-2H-pyran (3av)

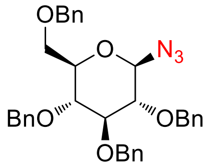

Following the general procedure for secondary alcohols (Method B), prepared from 2,3,4,6-tetra-*O*-benzyl-D-glucopyranose (**2av**) (108.2 mg, 0.2 mmol, 1.0 equiv), SulfoxFluor (194.6 mg, 0.56 mmol, 2.8 equiv), NaN_3 (26.0 mg, 0.4 mmol, 2.0 equiv), DBU (120 μL, 0.80 mmol, 4.0 equiv) in DMF for 12 hours; eluted with petroleum ether/EtOAc, 10:1 (v/v).

101.3 mg, 90% yield. Light yellow liquid. The signal for the minor epimer is near the limit of quantitatification; we conclude that the epimer ratio is greater than 20:1. **IR** (film): 3088, 3063, 3030, 2917, 2868, 2112, 1496, 1454, 1363, 1252, 1209, 1155, 1101, 1028, 910, 820, 735, 697, 648, 611, 568, 463 cm^{-1}. **^{1}H NMR** (400 MHz, $CDCl_3$) δ 7.42 – 7.29 (m, 20H), 4.98 (d, J = 11.6 Hz, 1H), 4.91 – 4.84 (m, 2H), 4.76 (s, 2H), 4.66 (d, J = 3.6 Hz, 1H), 4.63 (s, 1H), 4.48 (q, J = 11.8 Hz, 2H), 3.97 (d, J = 2.7 Hz, 1H), 3.83 – 3.79 (m, 1H), 3.69 – 3.57 (m, 4H). **^{13}C NMR** (101 MHz, $CDCl_3$) δ 138.51, 138.20, 138.13, 137.80, 128.56, 128.55, 128.49, 128.36, 128.33, 128.20, 128.04, 127.97, 127.92, 127.84, 127.74, 127.65, 90.65, 82.59, 78.85, 75.64, 75.60, 74.71,

73.72, 73.31, 72.93, 68.55. **HRMS (ESI)**: Calcd. For $C_{34}H_{35}O_5N_3Na^+$: 588.2469; Found: 588.2460. The NMR data are in agreement with the literature.[28]

6-(3-azidopropoxy)-2,5,7,8-tetramethyl-2-(4,8,12-trimethyltridecyl)chroman (3aw)

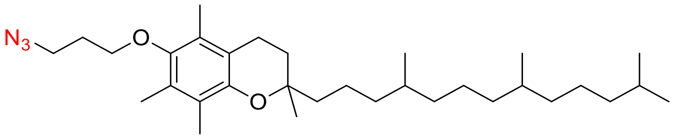

Following the general procedure for primary alcohols (Method A), prepared from 3-((2,5,7,8- tetramethyl-2-(4,8,12-trimethyltridecyl)chroman-6-yl)oxy)propan-1-ol (**2aw**) (97.7 mg, 0.2 mmol, 1.0 equiv), SulfoxFluor (152.9 mg, 0.44 mmol, 2.2 equiv), NaN_3 (52.0 mg, 0.8 mmol, 4.0 equiv), DBU (54 µL, 0.36 mmol, 1.8 equiv) in DMF for 12 hours; eluted with petroleum ether/EtOAc, 10:1 (v/v).

76.3 mg, 74% yield. Light yellow liquid. **IR** (film): 2953, 2873, 2851, 2093, 1575, 1455, 1416, 1378, 1343, 1296, 1257, 1199, 1158, 1089, 1060, 1015, 958, 909, 860, 816, 735, 669, 649, 614, 581, 557, 482 cm^{-1}. **^{1}H NMR** (400 MHz, $CDCl_3$) [mixture of rotamers] δ 3.74 (t, J = 6.0 Hz, 2H), 3.62 (t, J = 6.7 Hz, 2H), 2.59 (t, J = 6.7 Hz, 2H), 2.18 (s, 3H), 2.14 (s, 3H), 2.10 – 2.03 (m, 5H), 1.87 – 1.73 (m, 2H), 1.65 – 1.51 (m, 3H), 1.50 – 1.37 (m, 4H), 1.35 – 1.21 (m, 11H), 1.19 – 1.04 (m, 6H), 0.90 (s, 3H), 0.88 – 0.86 (m, 9H). **^{13}C NMR** (100 MHz, $CDCl_3$) [mixture of rotamers] δ 148.06, 148.01, 127.82, 125.82, 123.06, 117.70, 74.93, 69.27, 48.64, 40.21 [40.16], 39.52, 37.72 – 37.43 (m, 4C), 32.94 [32.92], 32.83 [32.81], 31.43 [31.38], 29.80, 28.12, 24.96 [24.95], 24.58, 24.01, 22.86, 22.77, 21.17, 20.80, 19.89 – 19.74 (m, 2C), 12.81, 11.94, 11.92. **MS** (**EI**, m/z, %): 513 (M^+, 3.10), 84 (100.00); **HRMS (DART)**: Calcd. For $C_{32}H_{59}O_2N_4^+$: 531.4633; Found: 531.4634.

(8*R*,9*R*,13*S*,14*S*,17*S*)-3-(3-Azidopropoxy)-13-methyl-7,8,9,11,12,13,14,15,16,17-deca- hydro-6*H*-cyclopenta[a]phenanthren-17-yl heptanoate (3ax)

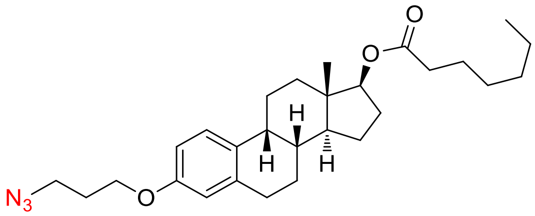

Following the general procedure for primary alcohols (Method A), prepared from (8*R*,9*R*,13*S*,14*S*,17*S*)-3-(3-hydroxypropoxy)-13-methyl-7,8,9,11,12,13,14,15,16,17-decahydro-6*H*-cyclopenta[*a*]phenanthren-17-yl heptanoate (**2ax**) (88.4 mg, 0.2 mmol, 1.0 equiv), SulfoxFluor (152.9 mg, 0.44 mmol, 2.2 equiv), NaN_3 (52.0 mg, 0.8 mmol, 4.0 equiv), DBU (54 μL, 0.36 mmol, 1.8 equiv) in DMF for 12 hours; eluted with petroleum ether/EtOAc, 10:1 (v/v).

47.6 mg, 51% yield. Light yellow liquid. **IR** (film): 3045, 2927, 2870, 2097, 1732, 1609, 1574, 1500, 1470, 1434, 1382, 1244, 1281, 1254, 1236, 1170, 1132, 1117, 1102, 1055, 1037, 1009, 974, 911, 872, 844, 817, 788, 735, 709, 647, 573, 557, 493, 446 cm^{-1}. **^{1}H NMR** (400 MHz, $CDCl_3$) δ 7.20 (d, J = 8.6 Hz, 1H), 6.70 (dd, J = 8.6, 2.5 Hz, 1H), 6.63 (d, J = 2.4 Hz, 1H), 4.70 (t, J = 8.4 Hz, 1H), 4.02 (t, J = 5.9 Hz, 2H), 3.51 (t, J = 6.6 Hz, 2H), 2.92 – 2.80 (m, 2H), 2.33 – 2.26 (m, 3H), 2.25 – 2.16 (m, 2H), 2.03 (p, J = 6.3 Hz, 2H), 1.91 – 1.83 (m, 2H), 1.78 – 1.71 m, 1H), 1.67 – 1.58 (m, 3H), 1.56 – 1.37 (m, 5H), 1.36 – 1.26 (m, 8H), 0.91 – 0.88 (m, 3H), 0.83 (s, 3H). **^{13}C NMR** (100 MHz, $CDCl_3$) δ 174.10, 156.68, 138.12, 132.97, 126.53, 114.60, 112.15, 82.55, 64.55, 49.93, 48.44, 43.94, 43.11, 38.71, 37.06, 34.76, 31.61, 29.90, 28.99, 28.96, 27.74, 27.35, 26.35, 25.23, 23.41, 22.64, 14.17, 12.23. **MS (EI**, m/z, %): 223 (M^+, 21.98), 113 (100.00); **HRMS (DART)**: Calcd. For $C_{28}H_{42}O_3N_3^+$: 468.3221; Found: 468.3221.

10-(3-(4-(2-Azidoethyl)piperazin-1-yl)propyl)-2-chloro-10*H*-phenothiazine (3ay)

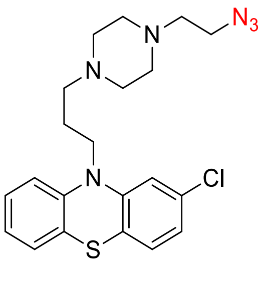

Following the general procedure for primary alcohols (Method A), prepared from perphenazine (**2ay**) (80.8 mg, 0.2 mmol, 1.0 equiv), SulfoxFluor (152.9 mg, 0.44 mmol, 2.2 equiv), NaN_3 (52.0 mg, 0.8 mmol, 4.0 equiv), DBU (54 µL, 0.36 mmol, 1.8 equiv) in DMF for 12 hours; eluted with petroleum ether/EtOAc, 1:3 (v/v).

35.2 mg, 41% yield. Light yellow liquid. **IR** (film): 3060, 2940, 2875, 2812, 2782, 2687, 2100, 1678, 1643, 1592, 1566, 1486, 1459, 1408, 1374, 1347, 1280, 1246, 1208, 1159, 1128, 1141, 1096, 1040, 1013, 976, 924, 849, 802, 749, 733, 681, 643, 584, 554, 483, 447 cm^{-1}. **^{1}H NMR** (400 MHz, $CDCl_3$) δ 7.16 – 7.10 (m, 2H), 7.00 (d, J = 8.1 Hz, 1H), 6.94 – 6.84 (m, 4H), 3.88 (t, J = 6.8 Hz, 2H), 3.33 (t, J = 6.1 Hz, 2H), 2.66 – 2.38 (m, 12H), 1.97 –1.90 (m, 2H). **^{13}C NMR** (100 MHz, $CDCl_3$) δ 146.57, 144.57, 133.32, 127.99, 127.62, 127.53, 124.92, 123.65, 123.02, 122.37, 115.96, 115.93, 57.14, 55.49, 53.17, 53.05, 48.28, 45.41, 24.18. **HRMS (ESI)**: Calcd. For $C_{21}H_{26}N_6ClS^+$: 429.1623; Found: 429.1621.

(*Z*)-(1-(4-(2-Azidoethoxy)phenyl)-4-chlorobut-1-ene-1,2-diyl)dibenzene (3az)

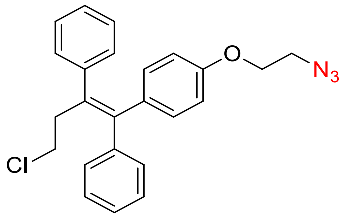

Following the general procedure for primary alcohols (Method A), prepared from ospemifene (**2az**) (75.8 mg, 0.2 mmol, 1.0 equiv), SulfoxFluor (152.9 mg, 0.44 mmol, 2.2 equiv), NaN_3 (52.0 mg, 0.8 mmol, 4.0 equiv), DBU (54 µL, 0.36 mmol, 1.8 equiv) in DMF for 12 hours; eluted with petroleum ether/EtOAc, 50:1 (v/v).

71.1 mg, 88% yield. White solid. **M.p.**: 87-89 °C. **IR** (film): 3078, 3056, 3020, 2960, 2931, 2870, 2249, 2109, 1605, 1575, 1507, 1492, 1460, 1442, 1412, 1386, 1349, 1286, 1241, 1175, 1063, 1113, 1029, 1013, 1002, 838, 807, 782, 765, 732, 702, 650, 617, 601, 559, 525, 517, 478 cm^{-1}. **^{1}H NMR** (400 MHz, $CDCl_3$) δ 7.42 – 7.38 (m, 2H), 7.33 – 7.30 (m, 3H), 7.25 – 7.21 (m, 2H), 7.19 – 7.15 (m, 3H), 6.85 – 6.82 (m, 2H), 6.61 – 6.58 (m, 2H), 4.03 – 4.00 (m, 2H), 3.51 (t, J = 5.0 Hz, 2H), 3.44 (t, J = 7.4 Hz, 2H), 2.96 (t, J = 7.4 Hz, 2H). **^{13}C NMR** (100 MHz, $CDCl_3$) δ 156.51, 142.89, 141.71,

140.99, 135.58, 135.52, 131.87, 129.63, 129.49, 128.47, 128.34, 127.09, 126.75, 113.62, 66.74, 50.24, 42.94, 38.67. **HRMS (DART)**: Calcd. For $C_{24}H_{23}ON_3Cl^+$: 404.1524; Found: 404.1526. The NMR data are in agreement with the literature.[29]

4-(4-(4-(4-(((3*R*,5*R*)-5-((1*H*-1,2,4-Triazol-1-yl)methyl)-5-(2,4-difluorophenyl)tetrahydrofuran-3-yl)methoxy)phenyl)piperazin-1-yl)phenyl)-1-((2*R*,3*S*)-2-azidopentan-3-yl) -1*H*-1,2,4-triazol-5(4*H*)-one (3ba)

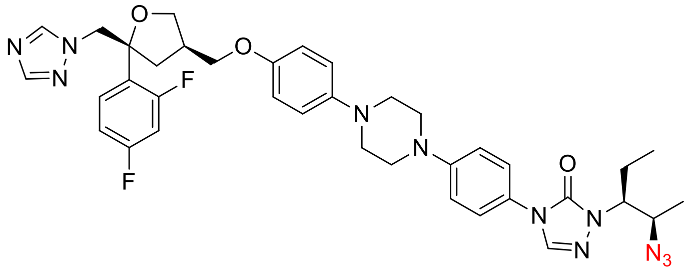

Following the general procedure for secondary alcohols (Method B), prepared from posaconazole (**2ba**) (140.2 mg, 0.2 mmol, 1.0 equiv), SulfoxFluor (194.6 mg, 0.56 mmol, 2.8 equiv), NaN_3 (26.0 mg, 0.4 mmol, 2.0 equiv), DBU (120 μL, 0.80 mmol, 4.0 equiv) in DMF for 12 hours; eluted with petroleum ether/EtOAc, 1:1 (v/v).

68.3 mg, 47% yield. White solid. **M.p.**: 161-164 °C. The signal for the minor epimer was not detected by ^{19}F NMR and ^{1}H NMR.; we conclude that the epimer ratio is greater than 20:1. **IR** (film): 3136, 3069, 2946, 2971, 2911, 2878, 2828, 2243, 2105, 1705, 1615, 1554, 1511, 1451, 1421, 1384, 1327, 1296, 1272, 1229, 1186, 1137, 1099, 1075, 1056, 1039, 964, 946, 910, 852, 825, 785, 733, 679, 663, 647, 583, 538, 514, 472 cm^{-1}. **^{1}H NMR** (400 MHz, $CDCl_3$) δ 8.10 (s, 1H), 7.78 (s, 1H), 7.66 (s, 1H), 7.43 – 7.34 (m, 3H), 7.01 (d, J = 9.1 Hz, 2H), 6.91 (d, J = 9.1 Hz, 2H), 6.87 – 6.65 (m, 4H), 4.65 – 4.61 (m, 1H), 4.52 – 4.48 (m, 1H), 4.13 – 4.01 (m, 2H), 3.77 (p, J = 6.3 Hz, 2H), 3.70 – 3.67 (m, 1H), 3.62– 3.58 (m, 1H), 3.36 – 3.33 (m, 4H), 3.22 – 3.19 (m, 4H), 2.65 – 2.50 (m, 2H), 2.09 – 2.03 (m, 1H), 1.94 (p, J = 7.3 Hz, 2H), 1.27 (d, J = 6.6 Hz, 3H), 0.87 (t, J = 7.3 Hz, 3H). **^{13}C NMR** (100 MHz, $CDCl_3$) δ 162.84 (dd, J = 249.7, 12.2 Hz, 1C), 159.06 (dd, J = 248.5, 12.1 Hz, 1C), 153.10, 152.90, 151.16, 150.75, 145.84, 144.64, 134.58, 128.68 (dd, J = 9.5, 5.5 Hz, 1C), 125.66, 125.47 (dd, J = 13.0, 3.7 Hz, 1C), 123.56, 118.56, 116.68, 115.24, 111.38 (dd, J = 20.6, 3.3 Hz,

1C), 104.69 (t, J = 26.1 Hz, 1C), 84.13 (d, J = 4.2 Hz), 70.84, 69.04, 60.72, 60.09, 56.02 (d, J = 3.9 Hz), 50.69, 49.25, 38.94, 37.54 (d, J = 3.4 Hz), 22.87, 16.29, 10.60. **^{19}F NMR** (376 MHz, $CDCl_3$) δ –109.44 (m, 1F), –110.94 (m, 1F). **HRMS (ESI)**: Calcd. For $C_{37}H_{42}O_3N_{11}F_2^+$: 726.3435; Found: 726.3434.

4. Synthetic Utility of Deoxyazidation of Alcohols

Synthesis of *tert*-butyl 4-(1-(1-((5-(dimethylamino)naphthalen-1-yl)sulfonyl)pyrrolidin-3-yl)-1*H*-1,2,3-triazol-4-yl)piperidine-1-carboxylate (9)

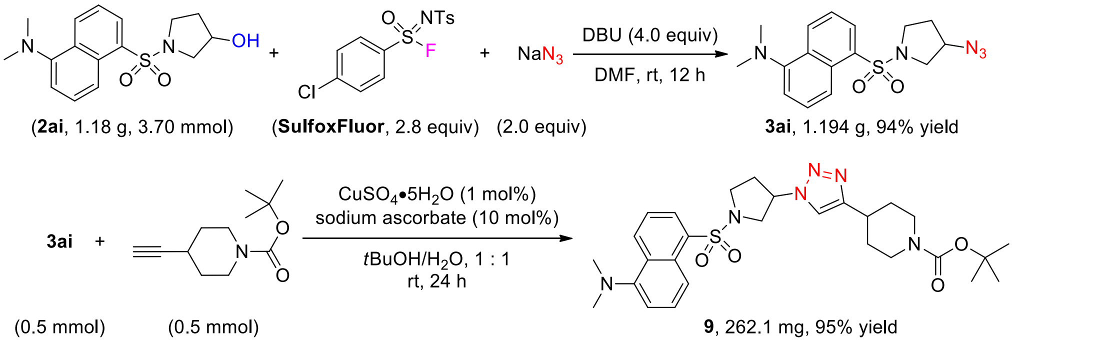

Following the general procedure for secondary alcohols (Method B), into a 250-mL Schlenk tube (glass) were sequentially added alcohol **2ai** (1.187 g, 3.7 mmol, 1.0 equiv), SulfoxFluor (3.6 g, 10.36 mmol, 2.8 equiv), NaN_3 (481.0 mg, 7.4 mmol, 2.0 equiv), DMF (40 mL), and DBU (2.22 mL, 14.8 mmol, 4.0 equiv) under N_2 atmosphere. The mixture was stirred at room temperature for 12 hours. After reaction completed, water (40 mL) was added and the mixture was extracted with Et_2O (3 × 20 mL). The combined organic layers were dried over Na_2SO_4, filtered, concentrated under reduced pressure, and purified by chromatography on silica gel eluted with PE/EA (8:1) to afford the azide **3ai** as a yellow liquid (1.194 g, 94% yield).

Azide **3ai** (172.5 mg, 0.5 mmol) and 1-boc-4-ethynylpiperidine (104.5 mg, 0.5 mmol) were suspended in a 1:1 mixture of water and tert-butyl alcohol (2 mL). Sodium ascorbate (0.05 mmol, 50 μL of freshly prepared 1M solution in water) was added, followed by copper(II) sulfate pentahydrate (1.25 mg, 0.005 mmol, in 17 μL of water). The heterogeneous mixture was stirred vigorously overnight, at which point it

cleared and TLC analysis indicated complete consumption of the reactant. The reaction mixture was diluted with water (5 mL), extracted with Et_2O (3 × 4 mL). Then the combined organic layers were dried over Na_2SO_4, filtered, concentrated under reduced pressure, and purified by chromatography on silica gel eluted with PE/EA (1:1) to afford the triazole **9** as a yellow liquid (262.1 mg, 95% yield).

Yellow liquid. **IR** (film): 3139, 3080, 2976, 2940, 2865, 2789, 2247, 1687, 1612, 1587, 1573, 1478, 1454, 1425, 1365, 1331, 1276, 1230, 1201, 1162, 1061, 1033, 985, 943, 913, 875, 838, 857, 793, 732, 683, 632, 596, 569, 541, 500, 482, 427 cm^{-1}. **^{1}H NMR** (400 MHz, $CDCl_3$) δ 8.56 (d, J = 8.5 Hz, 1H), 8.37 (d, J = 8.7 Hz, 1H), 8.24 (dd, J = 7.3, 1.1 Hz, 1H), 7.55 – 7.50 (m, 2H), 7.21 – 7.14 (m, 2H), 5.21 – 5.16 (m, 1H), 4.30 – 3.95 (m, 2H), 3.83 – 3.79 (m, 1H), 3.62 – 3.57 (m, 3H), 2.87 – 2.73 (m, 9H), 2.53 – 2.44 (m, 1H), 2.36 – 2.28 (m, 1H), 1.91 – 1.79 (m, 2H), 1.50 – 1.36 (m, 9H). **^{13}C NMR** (100 MHz, $CDCl_3$) δ 154.82, 152.30, 152.04, 133.16, 131.22, 130.42, 130.38, 130.10, 128.51, 123.29, 119.10, 117.77, 115.39, 79.54, 59.20, 52.81, 45.80, 45.47, 43.88, 33.57, 32.30, 31.69, 28.55. **HRMS (ESI)**: Calcd. For $C_{28}H_{39}O_4N_6S^+$: 555.2748; Found: 555.2749.

Synthesis of (3-(2-(4-(3-Bromophenyl)-1*H*-1,2,3-triazol-1-yl)ethyl)-5-methoxy-2-methyl-1*H*-indol-1-yl)(4-chlorophenyl)methanone (10)

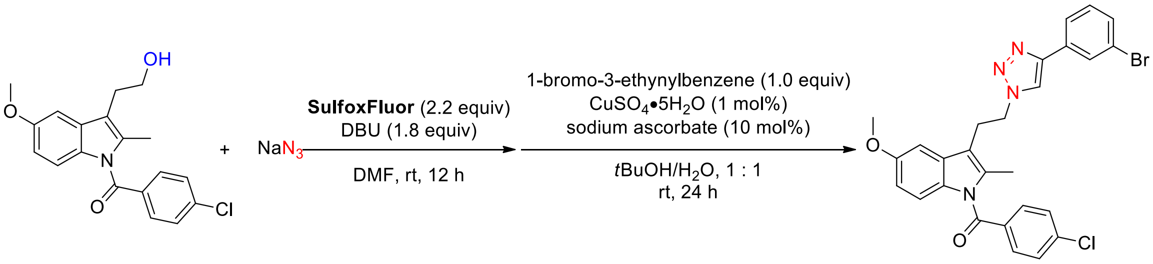

(**2at**, 171.5 mg, 0.5 mmol) (4.0 equiv) **10**, 226.6 mg, 83% yield

Following the general procedure for primary alcohols (Method A), into a 25-mL Schlenk tube (glass) were sequentially added alcohol **2at** (171.5 mg, 0.5 mmol, 1.0 equiv), SulfoxFluor (382.3 mg, 1.1 mmol, 2.2 equiv), NaN_3 (130.0 mg, 2.0 mmol, 4.0 equiv), DMF (5 mL), and DBU (135 μL, 0.9 mmol, 1.8 equiv) under N_2 atmosphere. The mixture was stirred at room temperature for 12 hours. After reaction completed,

water (5 mL) was added and the mixture was extracted with Et_2O (3 × 5 mL). The combined organic layers were dried over Na_2SO_4, filtered, concentrated under reduced pressure to afford crude azide. Then the crude azide and 1-bromo-3-ethynylbenzene (90.0 mg, 0.5 mmol, 1.0 equiv) were suspended in a 1:1 mixture of water and tert-butyl alcohol (2 mL). Sodium ascorbate (0.05 mmol, 50 μL of freshly prepared 1M solution in water) was added, followed by copper(II) sulfate pentahydrate (1.25 mg, 0.005 mmol, in 17 μL of water). The heterogeneous mixture was stirred vigorously overnight, at which point it cleared and TLC analysis indicated complete consumption of the reactant. The reaction mixture was diluted with water (5 mL), extracted with Et_2O (3 × 4 mL). Then the combined organic layers were dried over Na_2SO_4, filtered, concentrated under reduced pressure, and purified by chromatography on silica gel eluted with PE/EA (2:1) to afford the triazole **10** as a yellow solid (226.6 mg, 83% yield).

Yellow solid. **M.p.**: 121-123 °C. **IR** (film): 3131, 3086, 2998, 2959, 2929, 2833, 1731, 1679, 1604, 1567, 1477, 1456, 1434, 1400, 1358, 1325, 1241, 1287, 1223, 1179, 1156, 1136, 1089, 1070, 1050, 1015, 996, 981, 912, 835, 808, 785, 751, 737, 715, 686, 666, 612, 596, 550, 482, 435, 405 cm^{-1}. **^{1}H NMR** (400 MHz, $CDCl_3$) δ 7.87 – 7.86 (m, 1H), 7.64 (d, J = 7.8 Hz, 1H), 7.48 (d, J = 8.4 Hz, 2H), 7.44 – 7.41 (m, 2H), 7.29 (d, J = 8.4 Hz, 2H), 7.24 (t, J = 7.9 Hz, 1H), 7.06 (d, J = 9.0 Hz, 1H), 6.80 (d, J = 2.4 Hz, 1H), 6.70 (dd, J = 9.0, 2.4 Hz, 1H), 4.62 (t, J = 6.5 Hz, 2H), 3.80 (s, 3H), 3.25 (t, J = 6.5 Hz, 2H), 1.91 (s, 3H). **^{13}C NMR** (100 MHz, $CDCl_3$) δ 168.24, 156.22, 146.10, 139.31, 135.55, 133.73, 132.54, 131.11, 131.04, 130.97, 130.51, 130.01, 129.12, 128.53, 124.13, 123.03, 120.74, 115.29, 114.50, 111.77, 100.64, 55.78, 49.99, 25.67, 12.93. **HRMS (ESI)**: Calcd. For $C_{27}H_{23}O_2N_4BrCl^+$: 549.0687; Found: 549.0688.

5. Survey of Reaction Conditions

Deoxyazidation of Primary Alcohols with SulfoxFluor in DMF (Supplementary Table 2)

Method (Taking Supplementary Table 2, entry 1 as an example)

A 25-mL Schlenk tube was charged sequentially with 1-(4-fluorophenyl)propan-2-ol (**2a**) (14.0 mg, 0.10 mmol), **SulfoxFluor** (45.2 mg, 0.13 mmol), NaN_3 (26.0 mg, 0.40 mmol) in DMF (1.0 mL), and finally DBU (27 μL, 0.18 mmol) was added under N_2 atmosphere. After stirring at 600 rpm for 12 hours, 1-fluoronaphthalene (25 μL, 0.19 mmol) was added as an internal standard. The yields of 1-(2-azidoethyl)-4-fluorobenzene **3a**, 1-fluoro-4-(2-fluoroethyl)benzene **4**, and 1-fluoro-4-vinylbenzene **5** were determined by ^{19}F NMR spectroscopy analysis.

Summary of Results: The results showed that the highest yield (84%) for the azidation of primary alcohol **2a** could be obtained in 12 h by performing the reaction with 2.2 equiv of SulfoxFluor, 1.8 equiv of DBU and 4.0 equiv of NaN_3. And no fluorinaton and elimination side products were found under these reaction conditions. Moreover, DBU was the optimal base for this reaction.

Deoxyazidation of Secondary Alcohols with SulfoxFluor in DMF (Supplementary Table 3)

Method (Taking Supplementary Table 3, entry 1 as an example)

A 25-mL Schlenk tube was charged sequentially with 1-(4-fluorophenyl)propan-2-ol (**2b**) (15.2 mg, 0.10 mmol), **SulfoxFluor** (45.2 mg, 0.26 mmol), NaN_3 (26.0 mg, 0.40 mmol) in DMF (1.0 mL), and finally DBU (27 μL, 0.18 mmol) was added under N_2 atmosphere. After stirring at 600 rpm for 12 hours, 1-fluoronaphthalene (25 μL, 0.19 mmol) was added as an internal standard. The yield of 1-(2-azidopropyl)-4-fluorobenzene **3b**, 1-fluoro-4-(2-fluoro-propyl)benzene **6** and 1-fluoro-4-(prop-1-en-1-yl)benzene **7** was determined by ^{19}F NMR spectroscopy analysis.

Summary of Results: The results show that the highest yield (84%) for the azidation of secondary alcohol **2b** could be obtained in 12 h by performing the reaction with 2.8 equiv of SulfoxFluor, 4.0 equiv of DBU and 2.0 equiv of NaN_3. And no fluorinaton and elimination side products were found under these reaction conditions. PBSF was not suitable for this reaction because elimination occurred during azidation.

Screening the solvents for Deoxyazidation of Secondary Alcohols (Supplementary Table 4)

Method (Taking Supplementary Table 4, entry 1 as an example)

A 25-mL Schlenk tube was charged sequentially with 1-(4-fluorophenyl)propan-2-ol (**2b**) (15.2 mg, 0.10 mmol), **SulfoxFluor** (97.3 mg, 0.28 mmol), NaN_3 (13.0 mg, 0.20 mmol) in DMF (1.0 mL), and finally DBU (60 μL, 0.40 mmol) was added under N_2 atmosphere. After stirring at 600 rpm for 12 hours, 1-fluoronaphthalene (25 μL, 0.19 mmol) was added as an internal standard. The yields of 1-(2-azidopropyl)-4-fluorobenzene **3b**, 1-fluoro-4-(2-fluoro-propyl)benzene **6**, 1-fluoro-4-(prop-1-en-1-yl)benzene **7** and sulfonimidoyl ester **12** were determined by ^{19}F NMR spectroscopy analysis.

Summary of Results: The results show that DMF is the optimal solvent.

Comparison of Various Sulfonyl Fluorides and Sulfoximidoyl Fluorides in Deoxyazidation of Alcohols (Supplementary Table 5)

Method (Taking Supplementary Table 5, entry 1 as an example)

A 25-mL Schlenk tube was charged sequentially with 2,2,2-trifluoroethanol **2c** (10.0 mg, 7.2 μL, 0.10 mmol), **SulfoxFluor** (76.5 mg, 0.22 mmol), NaN_3 (26.0 mg, 0.40 mmol) in DMF (1.0 mL), and finally DBU (27 μL, 0.18 mmol) was added under N_2 atmosphere. After stirring at 600 rpm for 12 hours, $PhCF_3$ (25 μL, 0.20 mmol) was added as an internal standard. The yields of sulfonimidoyl ester **8a** and 2-azido-1,1,1-trifluoroethane **3c** were determined by ^{19}F NMR spectroscopy analysis by comparing integration of the ^{19}F NMR resonance of **3c** (–73.56 ppm) with that of $PhCF_3$ (–64.0 ppm).

Summary of Results: The results showed that **SulfoxFluor** was the optimal reagent in azidation of alcohols. Although PBSF gave an acceptable yield in azidation of 2,2,2-trifluoroethanol, elimination occurred during azidation of secondary alcohols as mentioned before. In the cases of other reagents, the reaction either stoped in the ester intermediate formation step or afforded the azidation product in a low yield.

6. Deoxyazidation of Alcohols with Bis(2,4-dichlorophenyl) chlorophosphate/NaN_3/DMAP: A Comparison

Preparation of Bis(2,4-dichlorophenyl) Phosphorochloridate (1j)

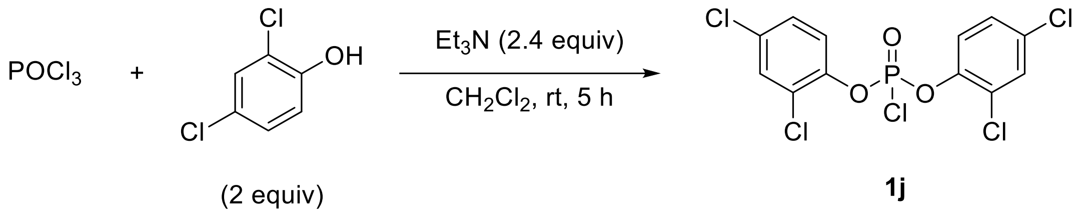

Prepared according to reported procedures.[30] To a 100-mL Schlenk tube equipped with a stir bar were added 2,4-dichlorophenol (21.2 mmol, 3.4563 g), Et_3N (50.9 mmol, 2.4 equiv, 3.5 mL) and CH_2Cl_2 (21 mL). Then $POCl_3$ (10.6 mmol, 1.6253 g) was added dropwise. The mixture was stirred for 5 h at room temperature. After completion, insoluble residues were filtered through Celite. The filtrate was diluted with CH_2Cl_2 and washed 3 times with water. Then the organic phase was dried over anhydrous Na_2SO_4. After the solution was filtered and the solvent was evaporated under vacuum, the desired bis(2,4-dichlorophenyl) phosphorochloridate (**1j**) was obtained and used without further purification.

White solid, yield: 76% (3.2598 g). **^{1}H NMR** (400 MHz, $CDCl_3$) δ 7.58-7.55 (m, 4H), 7.36-7.34 (m, 2H). **^{31}P NMR** (162 MHz, $CDCl_3$) δ -18.8. **^{13}C NMR** (101 MHz, $CDCl_3$) δ 145.0, 132.0, 130.8 (d, J = 3.7 Hz), 128.3 (t, J = 3.7 Hz), 126.7, 122.5 (d, J = 4.9 Hz). The NMR data are in agreement with the literature.[31]

Deoxyazidation of CF_3CH_2OH (2c) with Bis(2,4-dichlorophenyl) chlorophosphate/NaN_3/DMAP in DMF

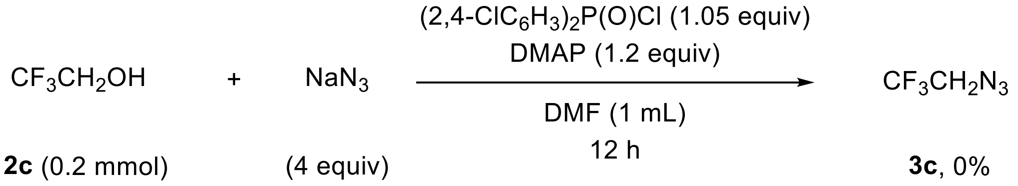

Method

The reaction was performed according to reported procedures.[32] To a solution of

CF_3CH_2OH (**2c**) (20.0 mg, 0.2 mmol) in anhydrous DMF (1.0 mL) were added at room temperature with stirring NaN_3 (51.3 mg, 0.8 mmol, 4.0 equiv) and 4-dimethylaminopyridine (DMAP) (29.3 mg, 0.24 mmol, 1.2 equiv) followed by bis(2,4-dichlorophenyl) phosphorochloridate (**1j**) (85.3 mg, 0.21 mmol, 1.05 equiv). After stirring at room temperature for 12 h, the reaction was monitored with ^{19}F NMR spectroscopy by adding $PhCF_3$ (22.5 mg) as the internal standard (*δ* -63.0 ppm). ^{19}F NMR analysis showed that no desired product $CF_3CF_2N_3$ (**3c**) was formed (**3c** is expected to appeared at *δ* -72.6 ppm). The remaining CF_3CH_2OH (**2c**) (*δ* -74.3 ppm) was 8% yield. In addition, three intermediates at *δ* -75.7, *δ* -75.9 and *δ* -76.0 were detected, which constituted 80% of all the CF_3CF_2O groups from CF_3CH_2OH.

Deoxyazidation of Secondary Alcohol 2r with Bis(2,4-dichlorophenyl) chlorophosphate/NaN3/DMAP in DMF

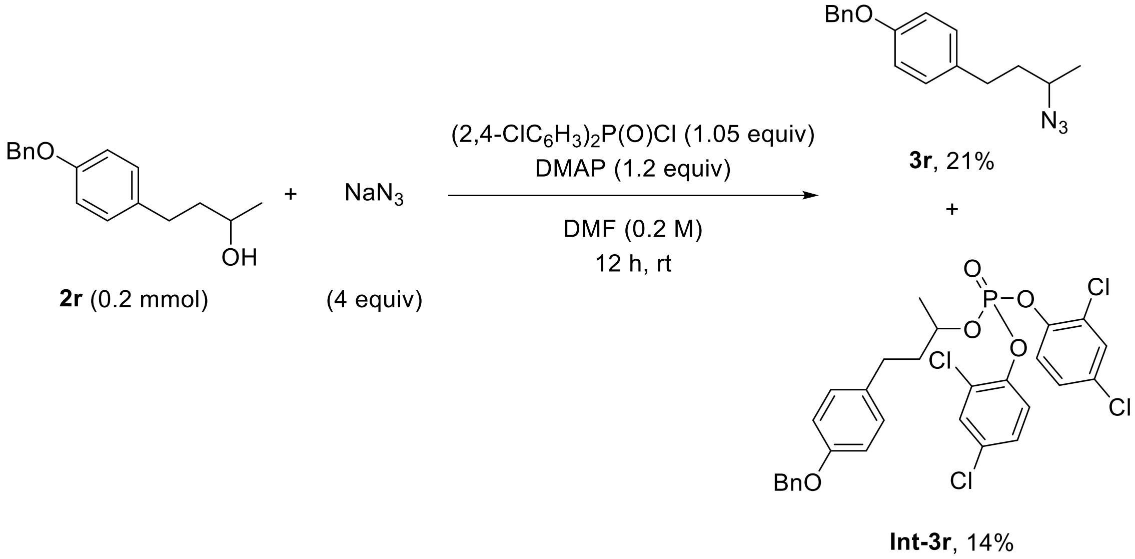

Method

The reaction was performed according to reported procedures.[32] To a solution of alcohol **2r** (51.3 mg, 0.2 mmol) in anhydrous DMF (1.0 mL) were added at room temperature with stirring NaN_3 (51.3 mg, 0.8 mmol, 4.0 equiv) and 4-dimethylaminopyridine (DMAP) (29.3 mg, 0.24 mmol, 1.2 equiv) followed by bis(2,4-dichlorophenyl) phosphorochloridate (**1j**) (85.3 mg, 0.21 mmol, 1.05 equiv). After stirring at room temperature for 12 h, the organic phase was extracted with

acetyl acetate and brine, and dried over anhydrous $NaSO_4$. After filtration, the solvent was removed in vacuo. The crude product was purified by flash column chromatography to afford the starting material **2r** (18.4 mg, 36% yield), desired azide product **3r** (12.0 mg, 21% yield) and the intermediate **Int-3r** (17.5 mg, 14% yield).

1-(3-Azidobutyl)-4-(benzyloxy)benzene (3r)

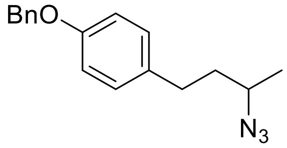

12.0 mg, 21% yield. White solid. **^{1}H NMR** (400 MHz, $CDCl_3$) *δ* 7.56-7.30 (m, 5H), 7.18-7.06 (m, 2H), 6.93 (d, *J* = 8.5 Hz, 2H), 5.06 (s, 2H), 3.52-3.31 (m, 1H), 2.76-2.58 (m, 2H), 1.87-1.68 (m, 2H), 1.30 (d, *J* = 6.5 Hz, 3H). **^{13}C NMR** (126 MHz, $CDCl_3$) *δ* 157.3, 137.3, 133.7, 129.5, 128.7, 128.0, 127.6, 115.0, 70.2, 57.2, 38.2, 31.6, 19.6. **MS** (ESI, *m*/*z*): 282.2 $[M+H]^+$.

4-(4-(Benzyloxy)phenyl)butan-2-yl bis(2,4-dichlorophenyl) phosphate (Int-3r)

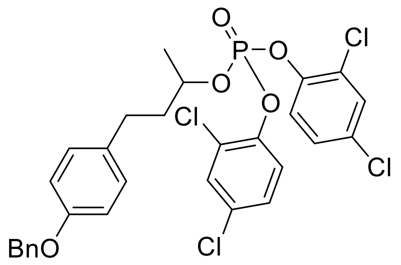

17.5 mg, 14% yield. Colorless oil. **^{1}H NMR** (400 MHz, $CDCl_3$) *δ* 7.51-29 (m, 9H), 7.22 (dt, *J* = 8.9, 3.0 Hz, 2H), 7.08-7.01 (m, 2H), 6.89 (d, *J* = 8.6 Hz, 2H), 5.04 (s, 2H), 4.88 (p, *J* = 6.3 Hz, 1H), 2.64 (qt, *J* = 13.8, 7.0 Hz, 2H), 2.03 (ddt, *J* = 13.4, 9.3, 6.5 Hz, 1H), 1.90 (q, *J* = 13.0, 10.5 Hz, 1H), 1.46 (d, *J* = 6.2 Hz, 3H). **^{31}P NMR** (162 MHz, $CDCl_3$) *δ* -13.53. **^{13}C NMR** (126 MHz, $CDCl_3$) δ 157.3, 145.4 (dd, *J* = 6.2, 3.0 Hz), 137.2, 133.3, 131.3 (d, *J* = 2.2 Hz), 130.6, 129.4, 128.7, 128.2 (m), 128.0, 127.6, 126.6 (d, *J* = 7.8 Hz), 122.3 (d, *J* = 2.5 Hz), 115.0, 79.5 (d, *J* = 7.2 Hz), 70.2, 39.3 (d, *J* = 6.6 Hz), 30.4, 21.6 (d, *J* = 3.0 Hz). **MS** (ESI, *m*/*z*): 625 $[M+H]^+$. **HRMS** (ESI) *m*/*z*: Calcd. for $C_{29}H_{25}Cl_4O_5P$ $[M+H]^+$: 625.0266; Found: 625.0266. **IR** (film):

3032, 2933, 1611, 1582, 1511, 1475, 1384, 1298, 1223, 1131, 1100, 1058, 1015, 953, 828, 737, 717, 695, 573 cm^{-1}.

7. Mechanistic Study

For details, see Supplementary Figure 138.

Method (Taking Supplementary Figure 138, Eq. (a) as an example)

A 25-mL Schlenk tube was charged sequentially with 1-(4-fluorophenyl)propan-2-ol (**2a**) (14.0 mg, 0.10 mmol), **SulfoxFluor** (76.5 mg, 0.22 mmol), NaN_3 (26.0 mg, 0.40 mmol) in DMF (1.0 mL), and finally DBU (27 μL, 0.18 mmol) was added under N_2 atmosphere. After stirring at 600 rpm for 12 hours, 1-fluoronaphthalene (25 μL, 0.19 mmol) was added as an internal standard. The yields of 1-(2-azidoethyl)-4-fluorobenzene **3a** and 1-fluoro-4-(2-fluoroethyl)benzene **4** were determined by ^{19}F NMR spectroscopy analysis.

In the case of Supplementary Figure 138, Eq. (b), the formation of the sulfonimidoyl azide intermediate was confirmed by MS analysis:

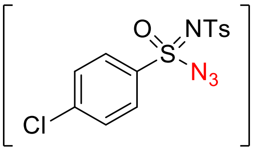

HRMS (DART): Calcd for $C_{13}H_{12}O_3N_4ClS_2$ (M^+): 371.0034; Found: 371.0033. However, all the attempts for the separation of the sulfonimidoyl azide intermediate failed due to its instability in our reaction system.

Summary of Results: The results showed that **SulfoxFluor**, DBU and NaN_3 were essential in the deoxy-azidation of alcohols. **SulfoxFluor** and DBU could react, but the intermediate they formed could not afford the azide **3a**. Based on the results, a proposed reaction pathway for the deoxyazidation of alcohols with **SulfoxFluor** is shown in the following scheme:

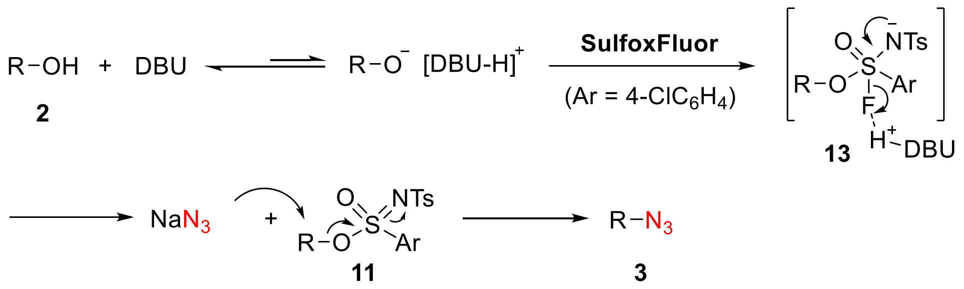

First, DBU deprotonates the alcohol to generate the alcoholate anion, which undergoes very fast nucleophilic addition to the SulfoxFluor to afford a pentacoordinated intermediate **13**. Then the stabilization effect of the protonated DBU on fluoride promotes the quick release of the fluorine substituent from intermediate **13** to afford the sulfonimidate ester intermediate **11**. Finally, the nucleophilic displacement of the sulfonimidate group by NaN_3 provided the corresponding azides.

For the NMR spectra of the reaction mixture in Supplementary Figure 138, entries 33 and 35, see Supplementary Figures 135-137.

Supplementary Figures

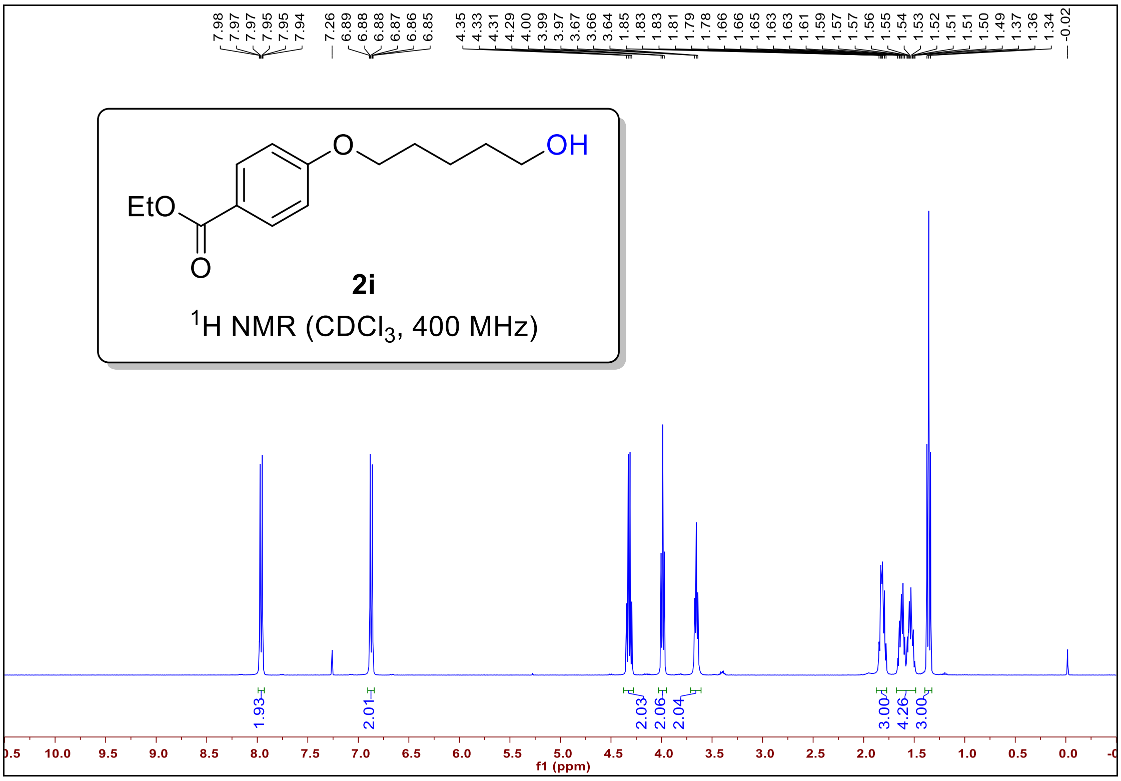

Supplementary Figure 1. ^{1}H NMR Spectrum of 2i

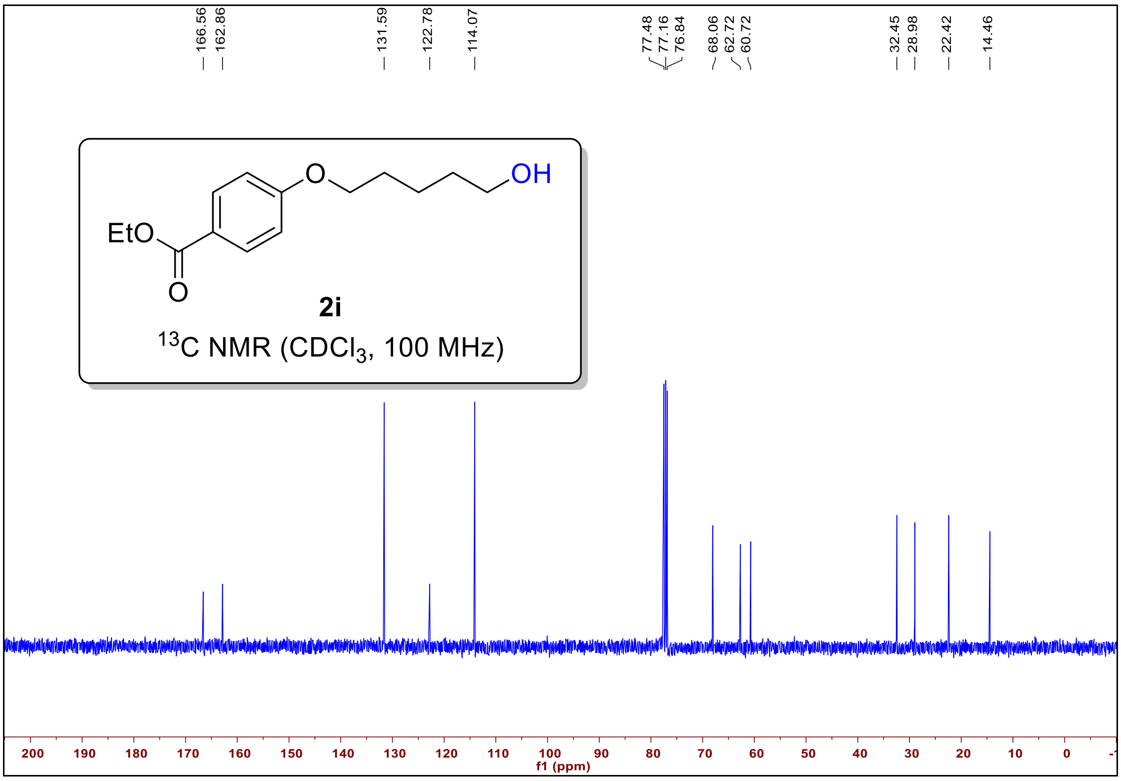

Supplementary Figure 2. ^{13}C NMR Spectrum of 2i

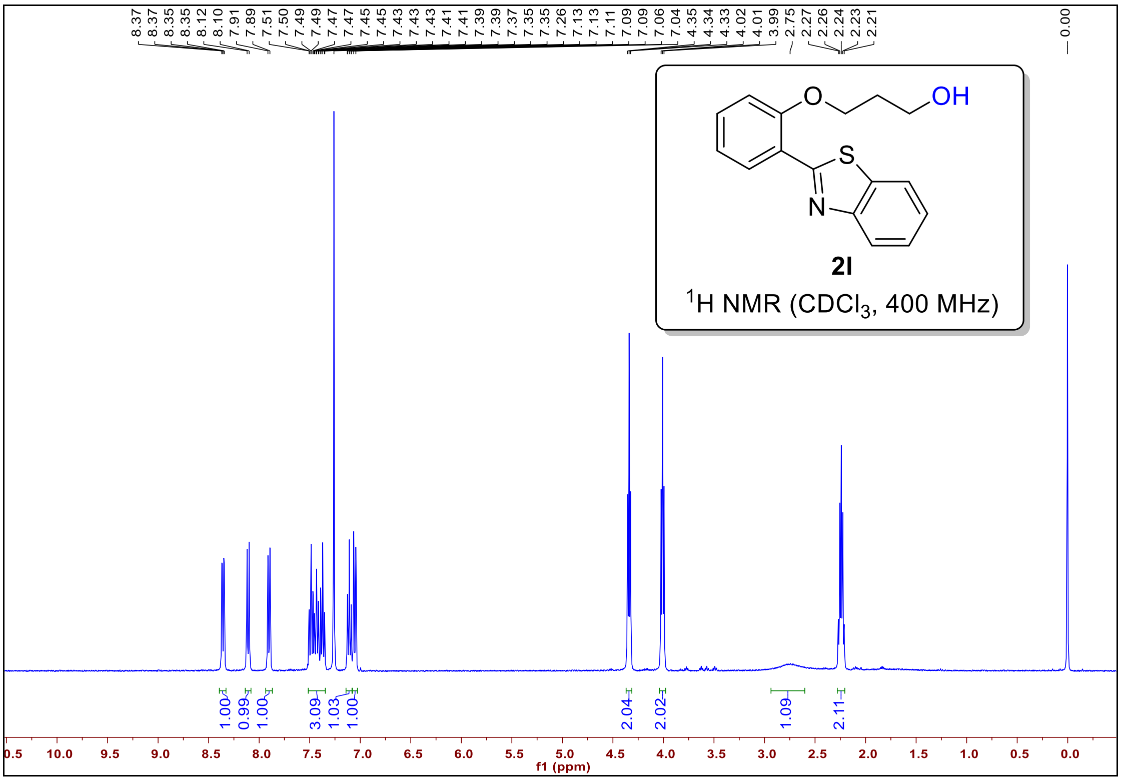

Supplementary Figure 3. ^{1}H NMR Spectrum of 2l

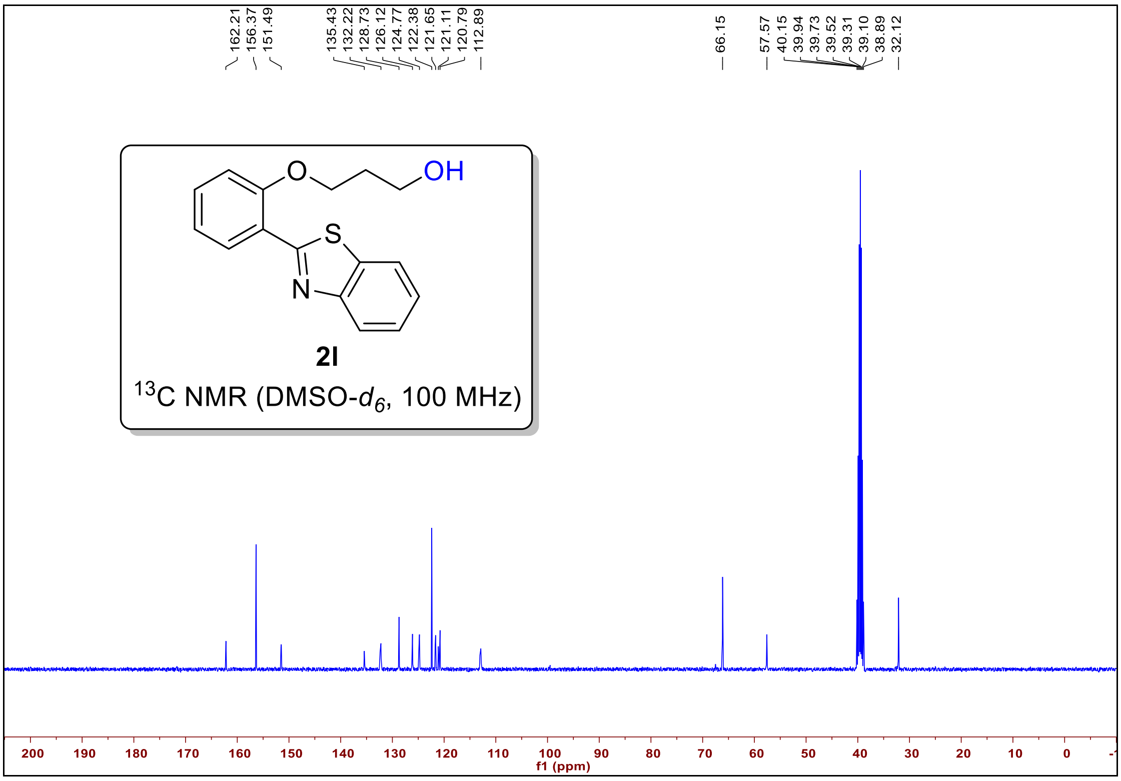

Supplementary Figure 4. ^{13}C NMR Spectrum of 2l

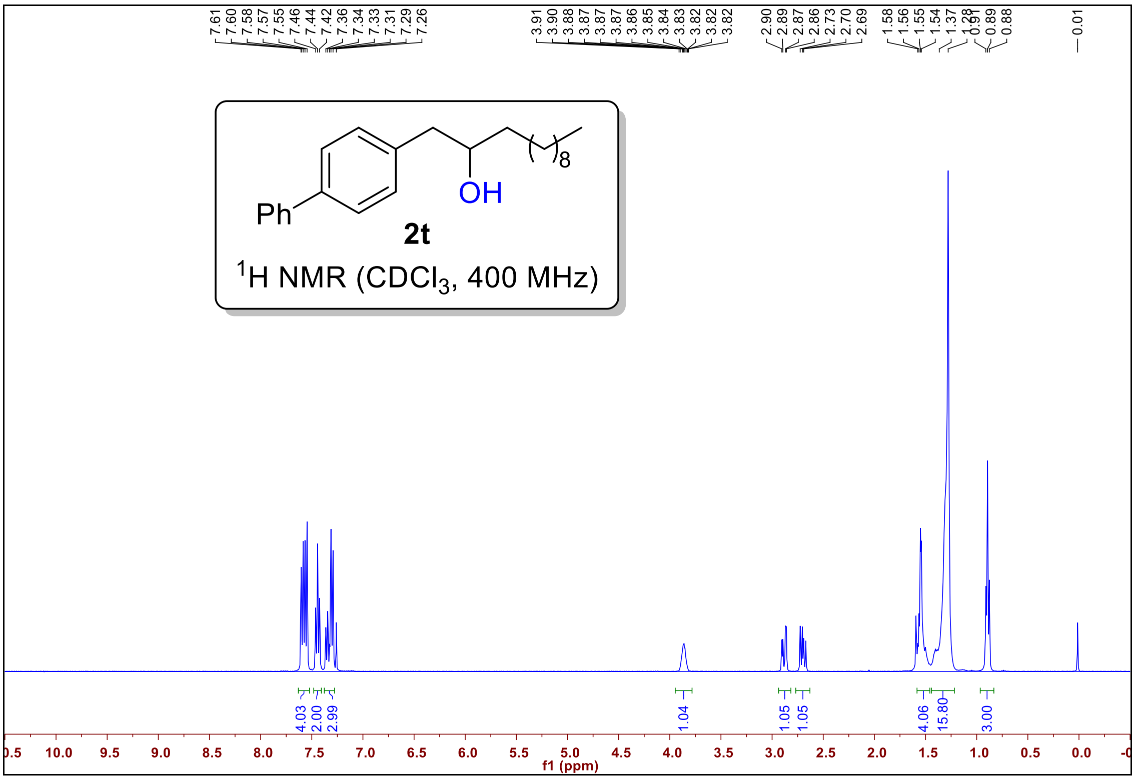

Supplementary Figure 5. ^{1}H NMR Spectrum of 2t

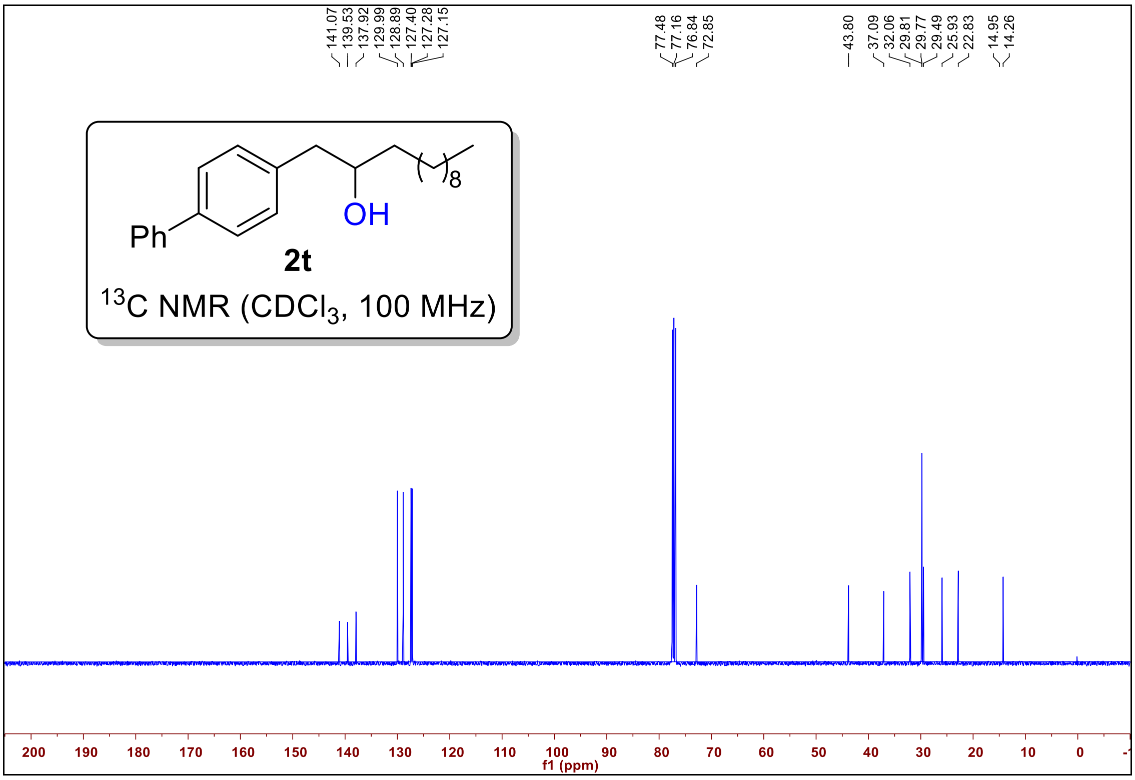

Supplementary Figure 6. ^{13}C NMR Spectrum of 2t

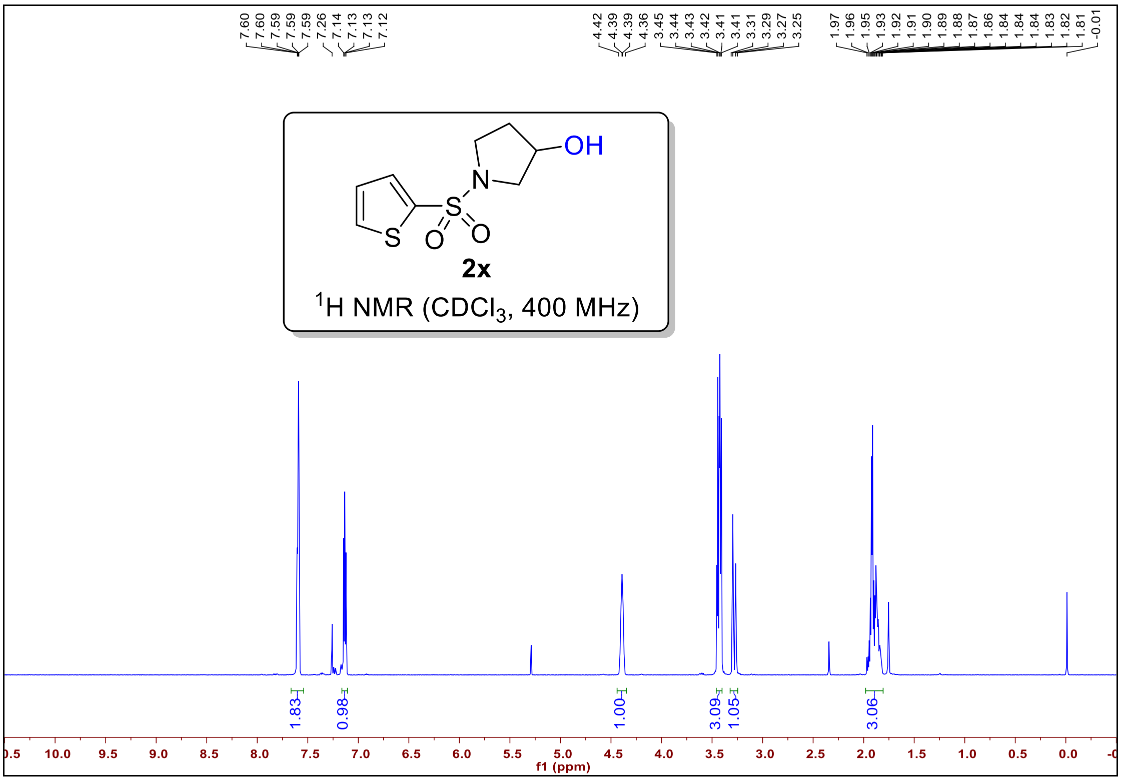

Supplementary Figure 7. ^{1}H NMR Spectrum of 2x

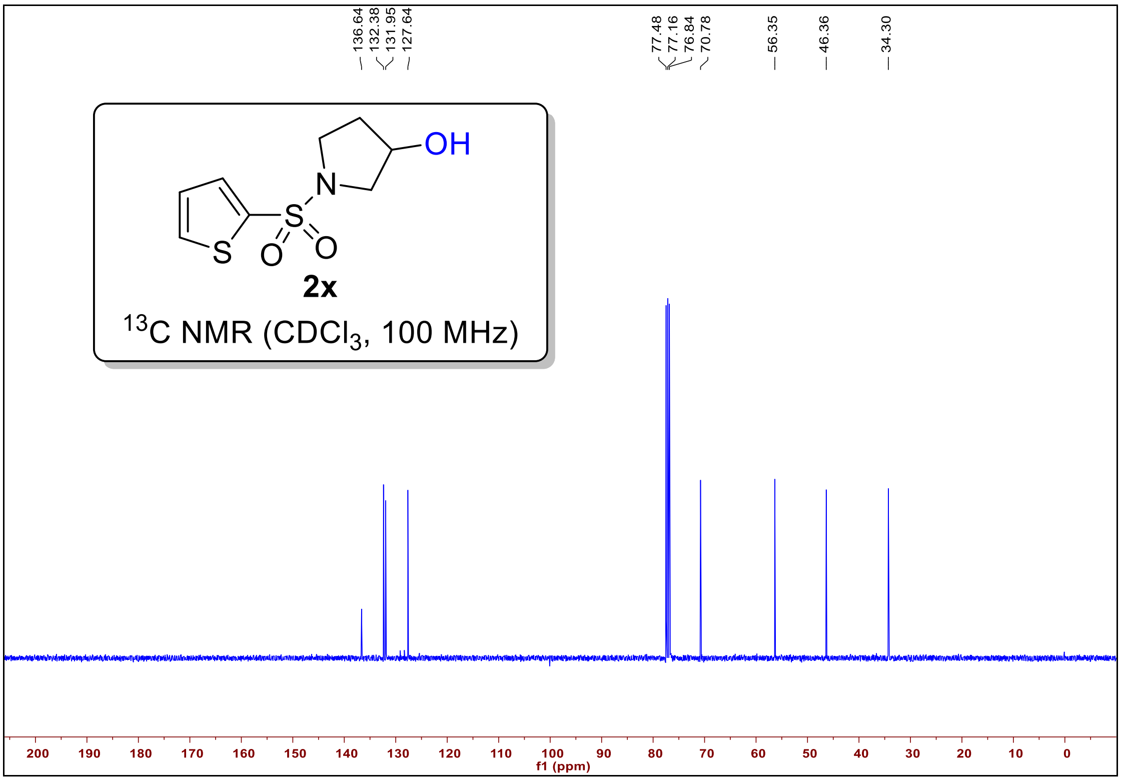

Supplementary Figure 8. ^{13}C NMR Spectrum of 2x

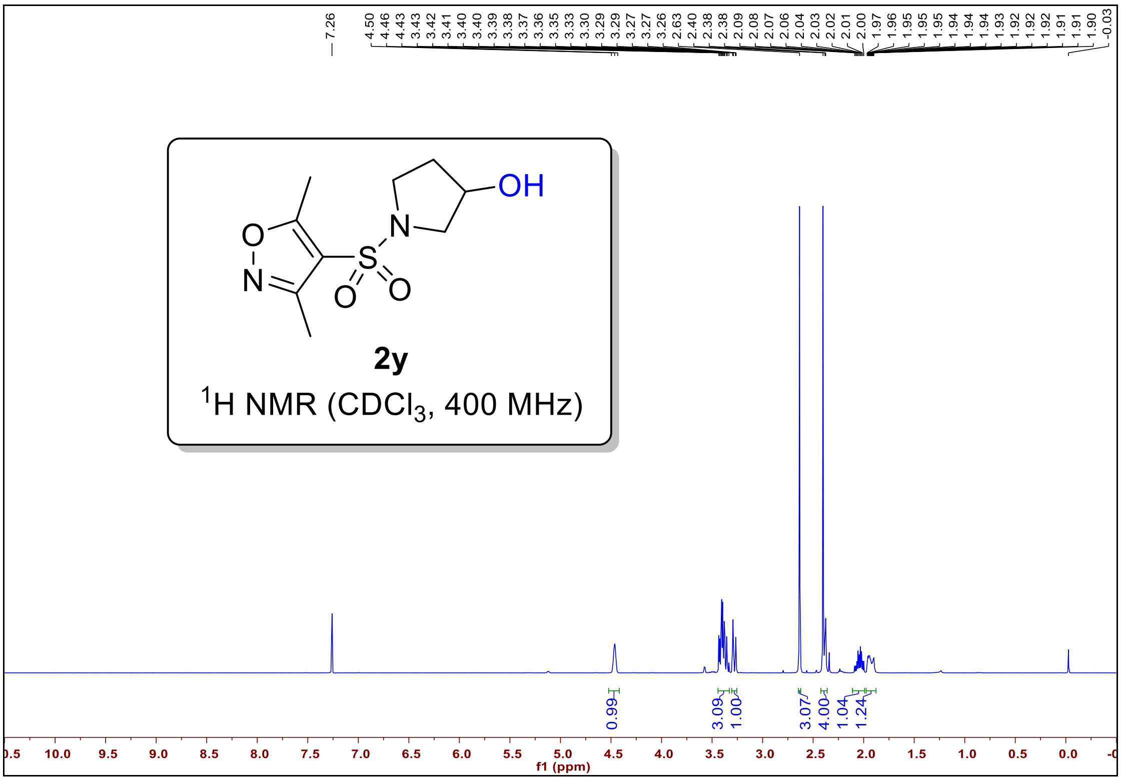

Supplementary Figure 9. ^{1}H NMR Spectrum of 2y

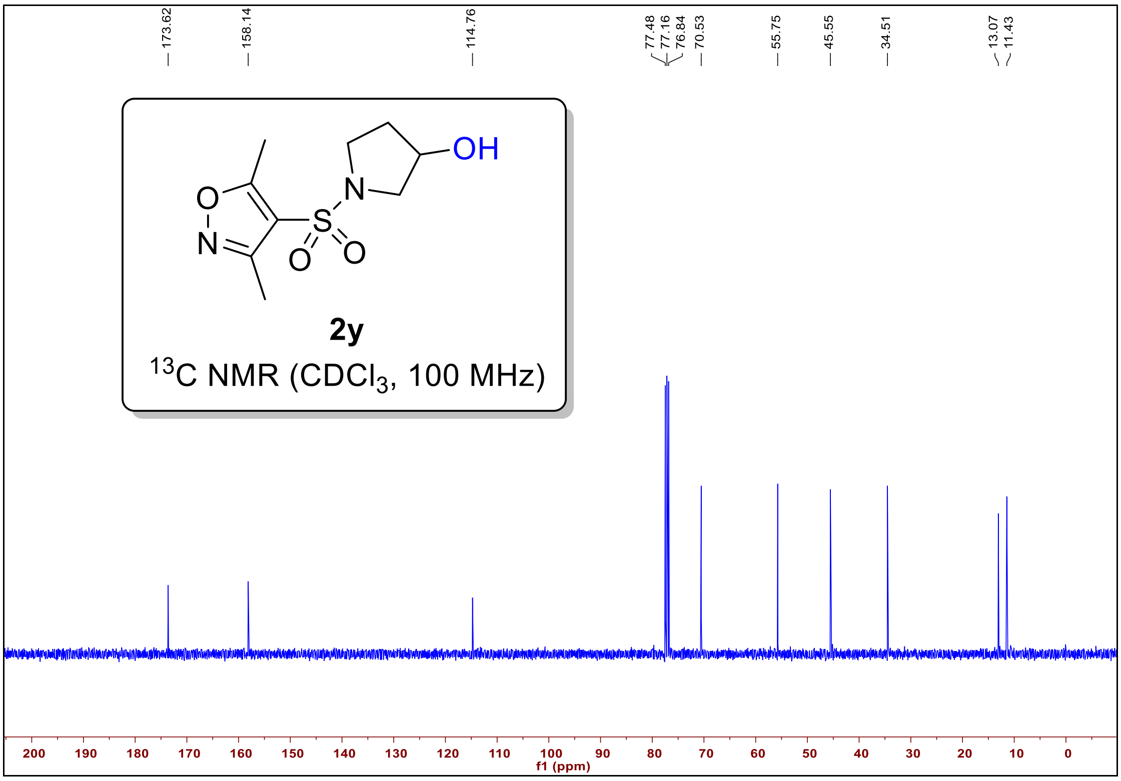

Supplementary Figure 10. ^{13}C NMR Spectrum of 2y

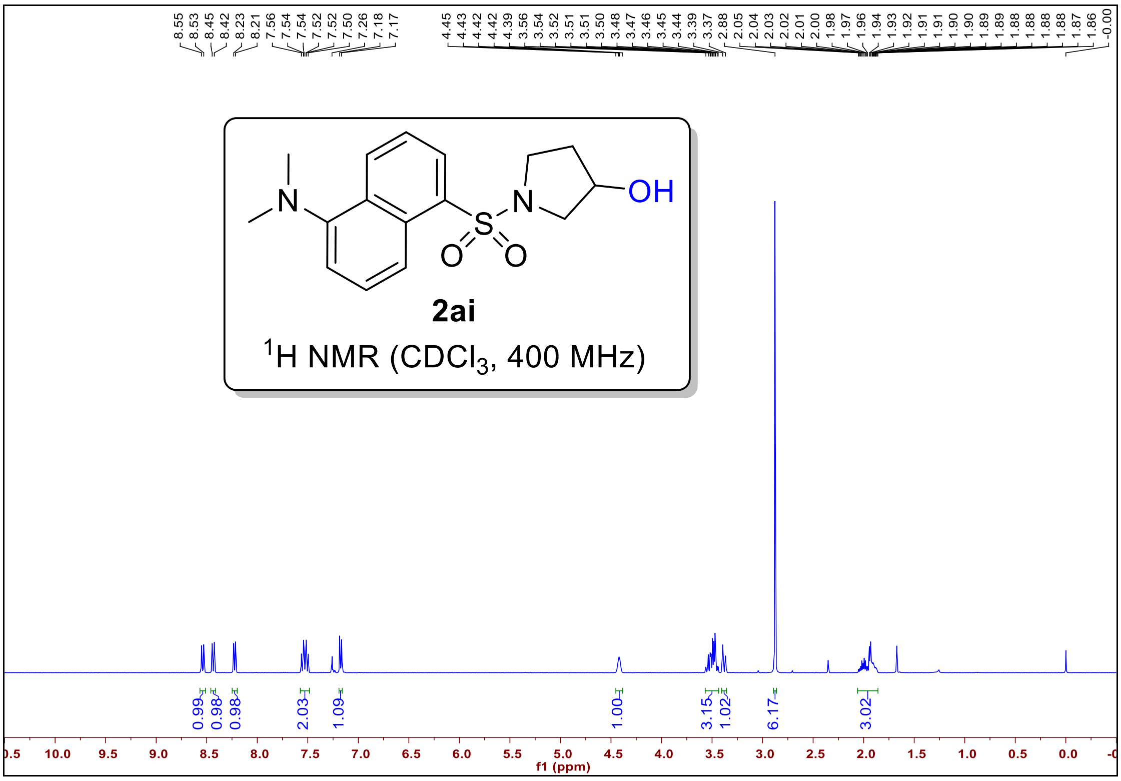

Supplementary Figure 11. ^{1}H NMR Spectrum of 2ai

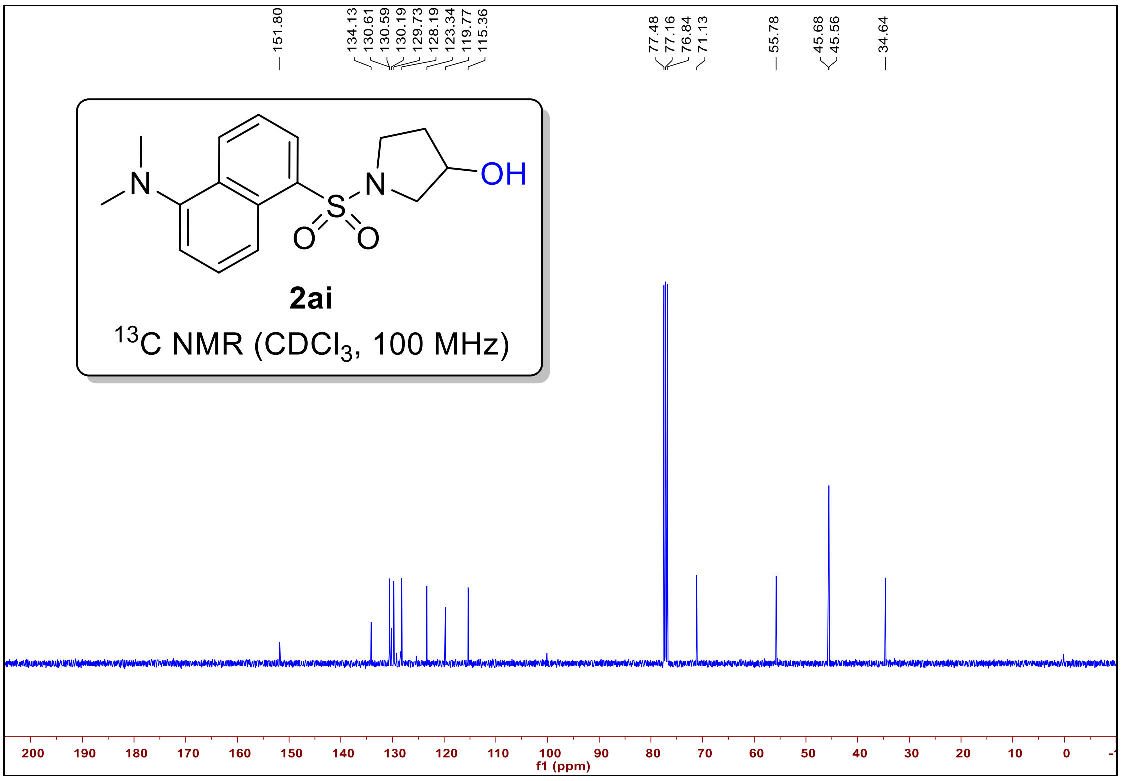

Supplementary Figure 12. ^{13}C NMR Spectrum of 2ai

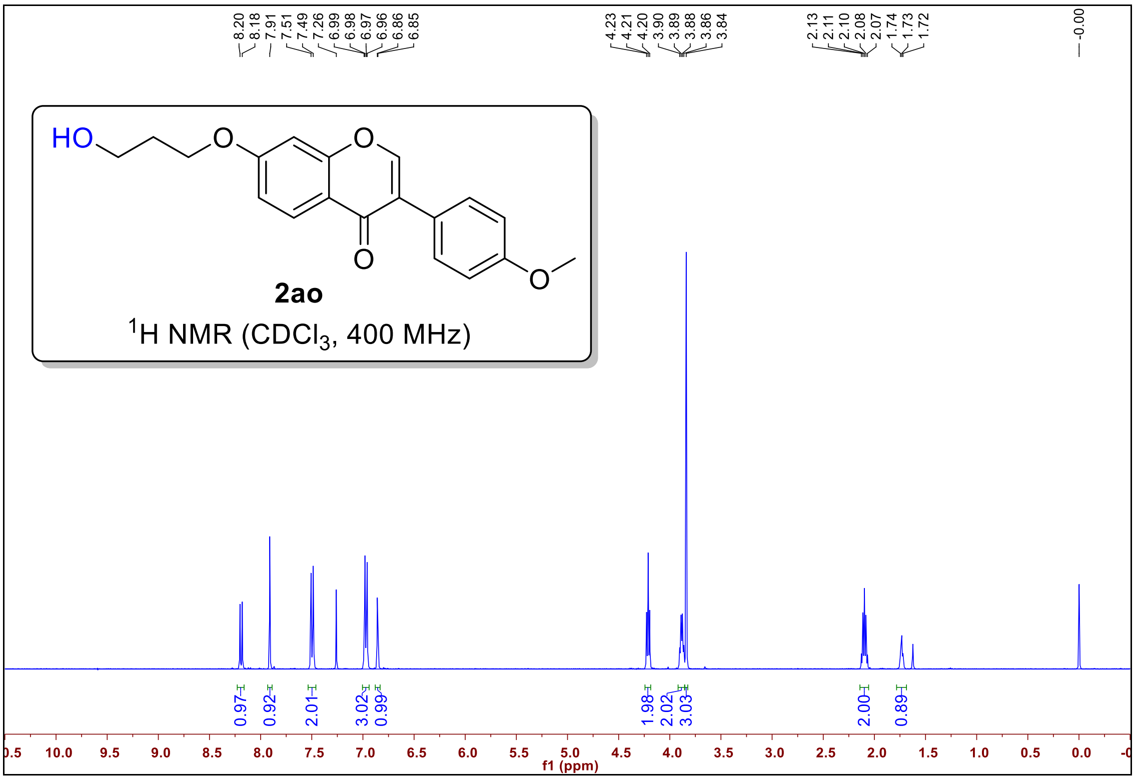

Supplementary Figure 13. ^{1}**H NMR Spectrum of 2ao**

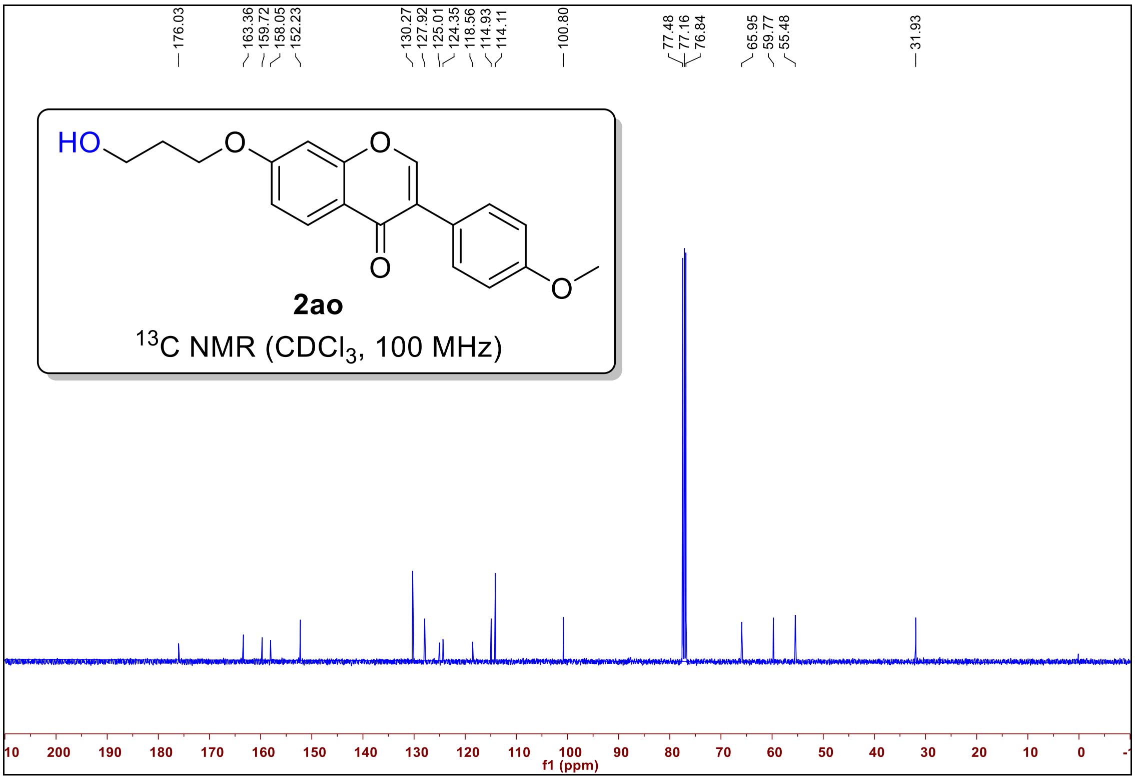

Supplementary Figure 14. ^{13}**C NMR Spectrum of 2ao**

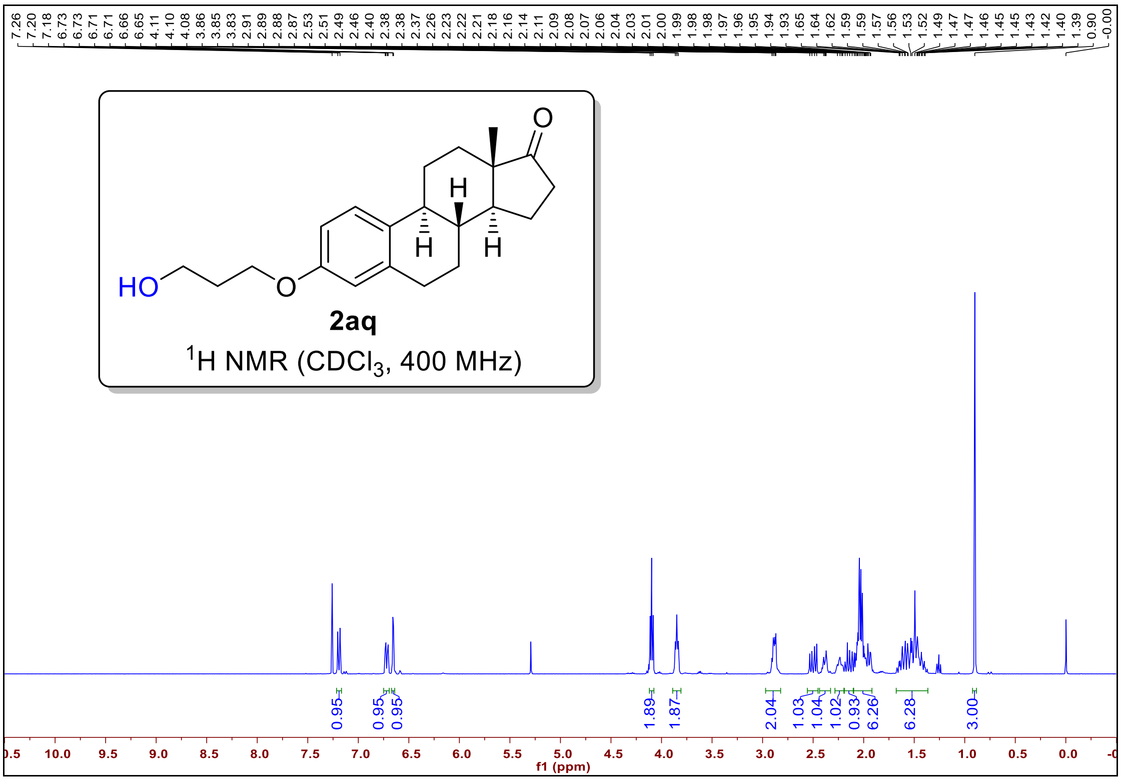

Supplementary Figure 15. ¹H NMR Spectrum of 2aq

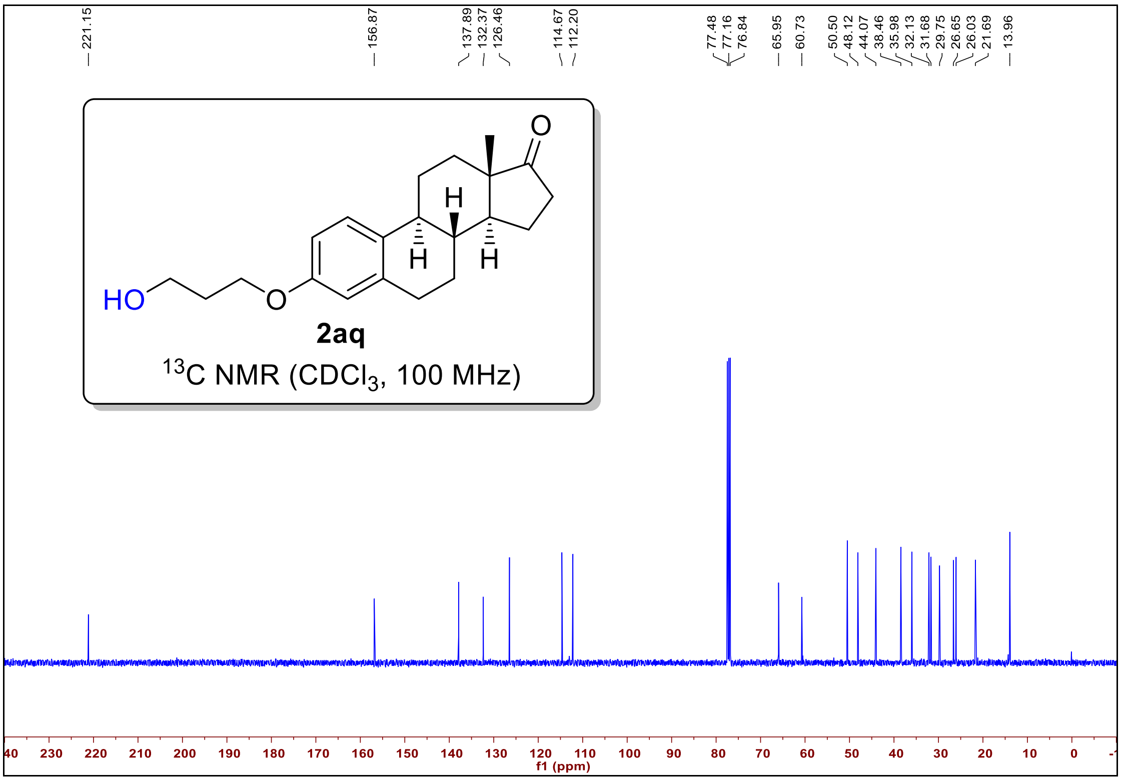

Supplementary Figure 16. ¹³C NMR Spectrum of 2aq

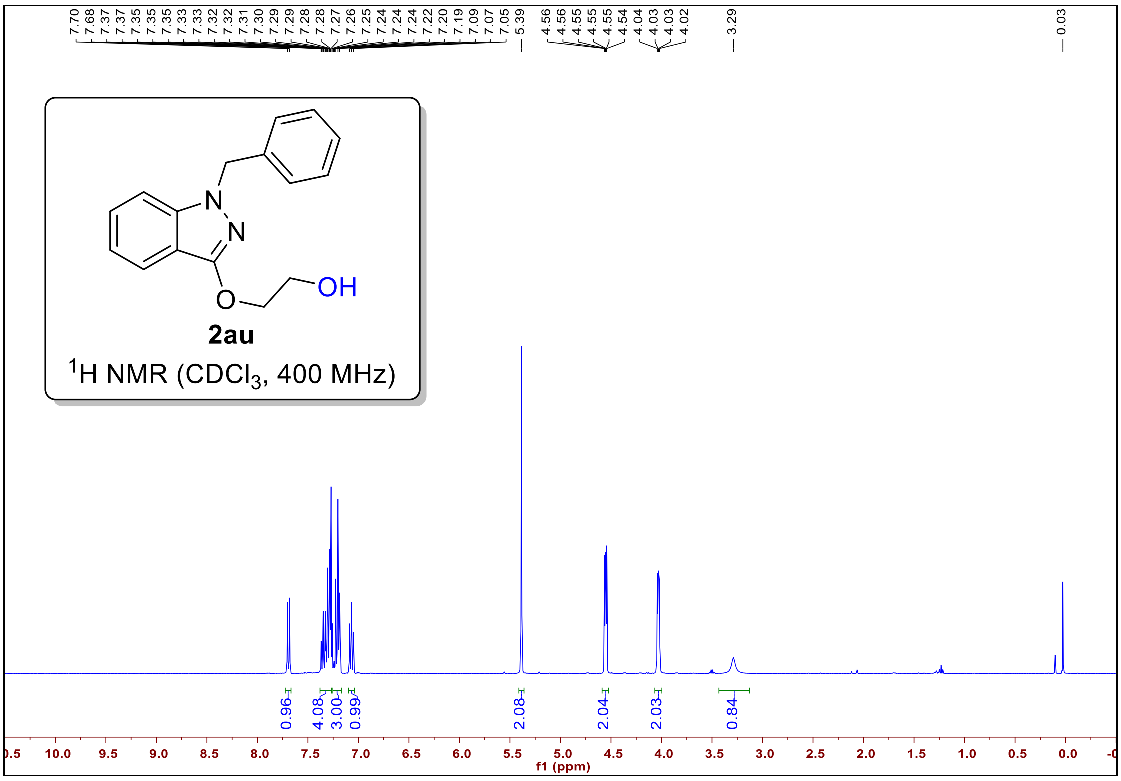

Supplementary Figure 17. ^{1}H NMR Spectrum of 2au

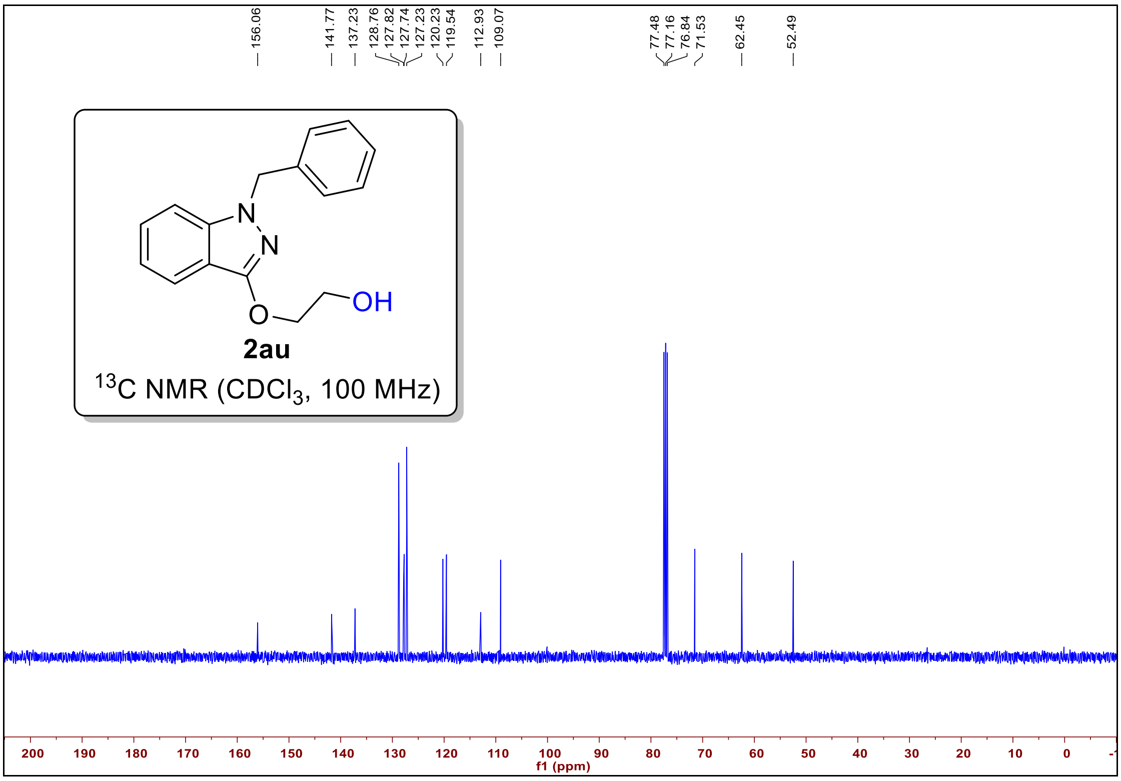

Supplementary Figure 18. ^{13}C NMR Spectrum of 2au

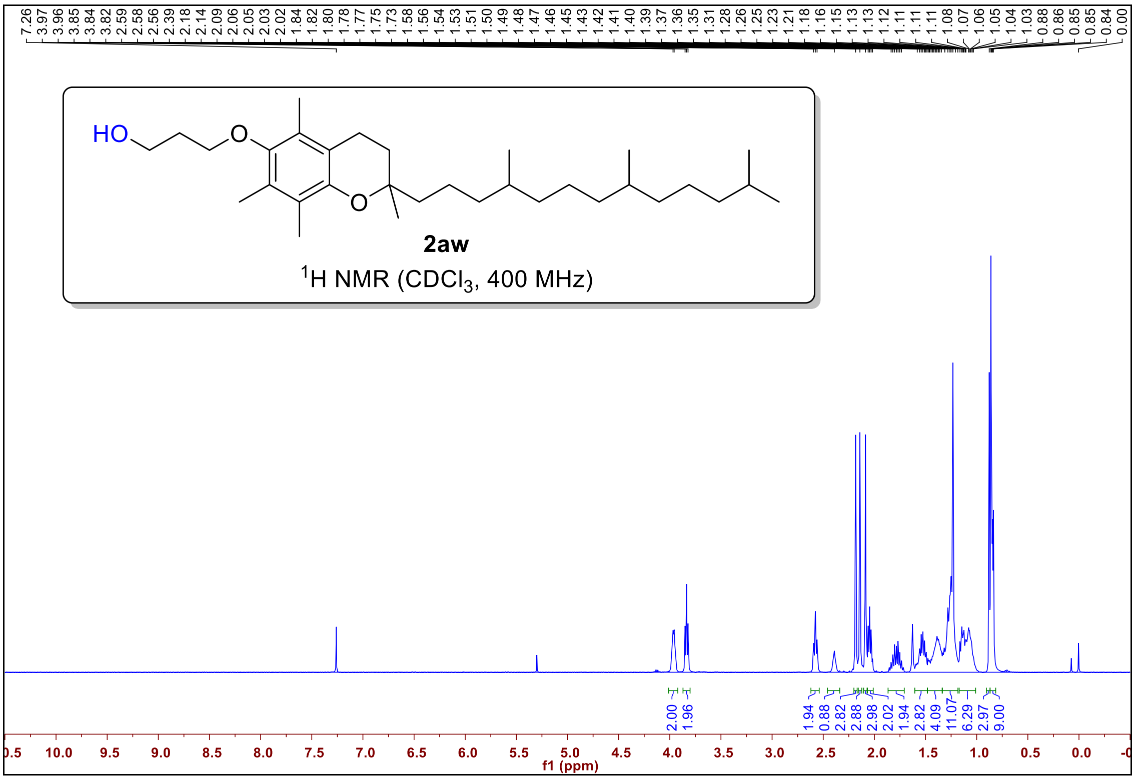

Supplementary Figure 19. ^{1}H NMR Spectrum of 2aw

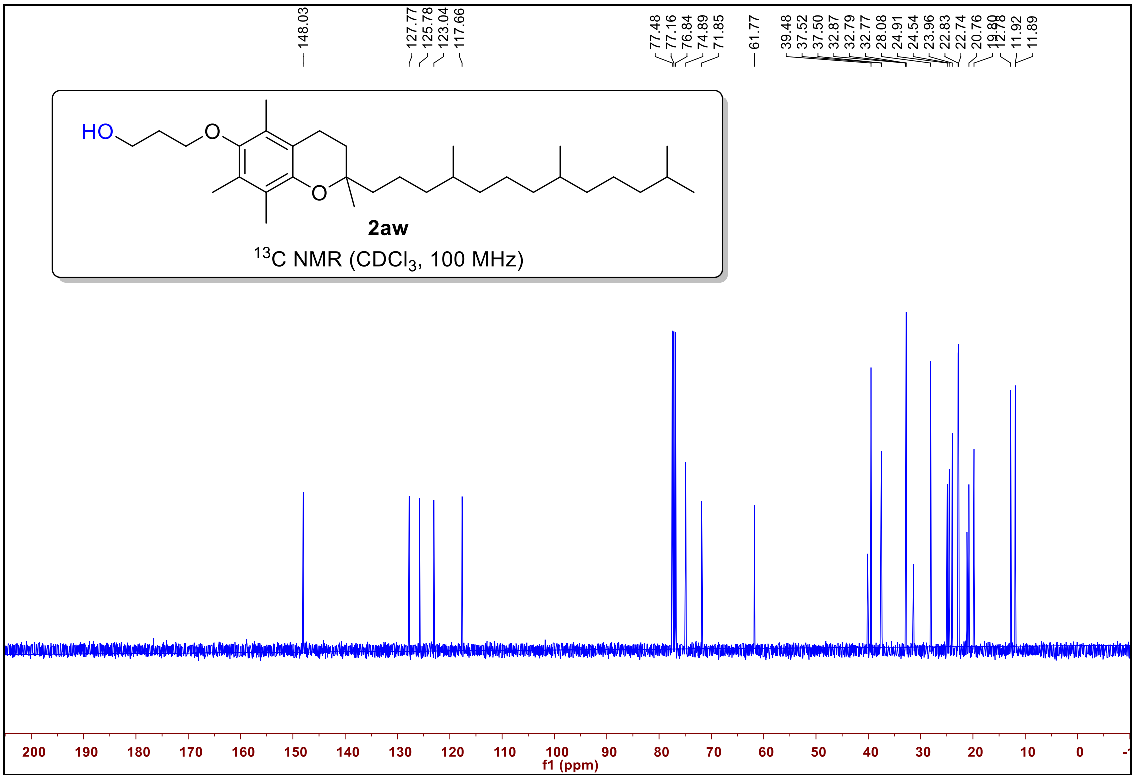

Supplementary Figure 20. ^{13}C NMR Spectrum of 2aw

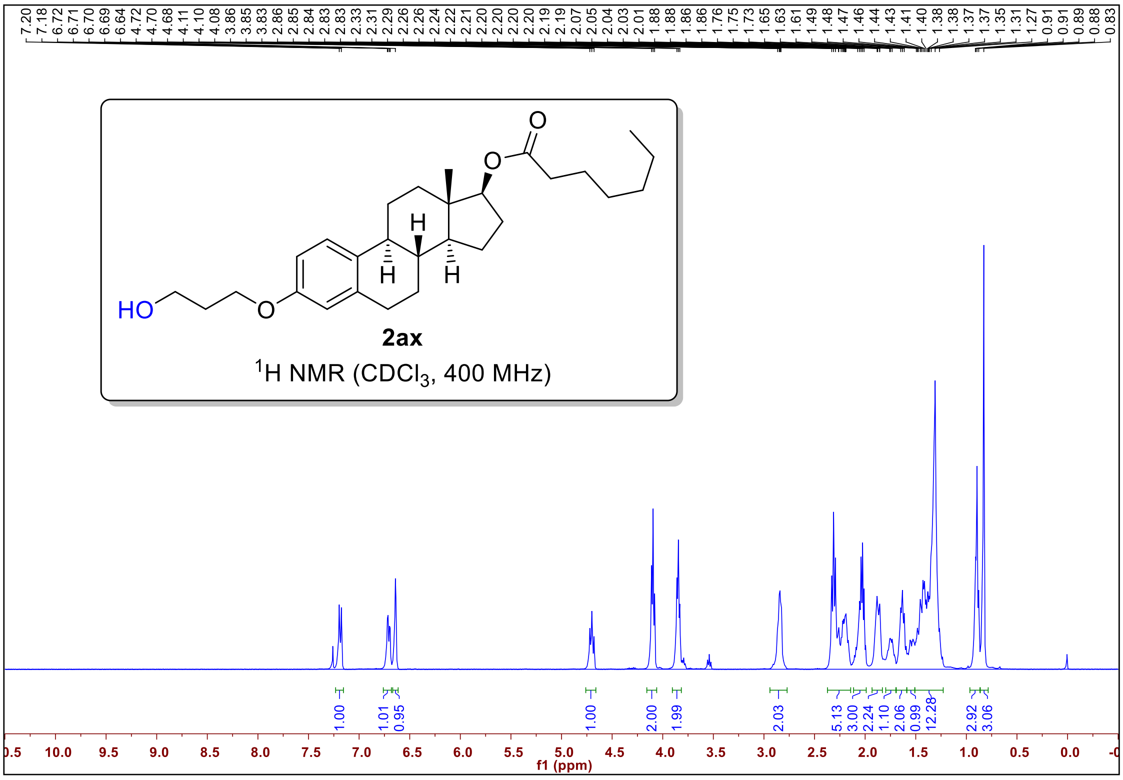

Supplementary Figure 21. ^{1}H NMR Spectrum of 2ax

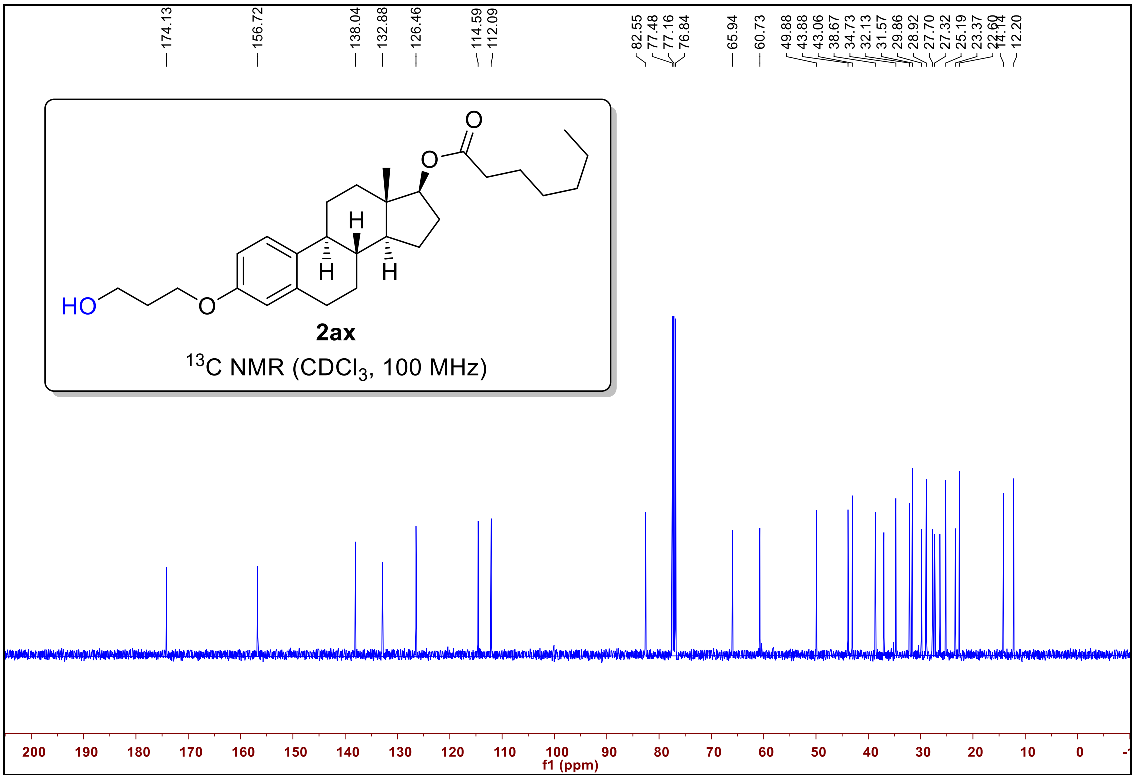

Supplementary Figure 22. ^{13}C NMR Spectrum of 2ax

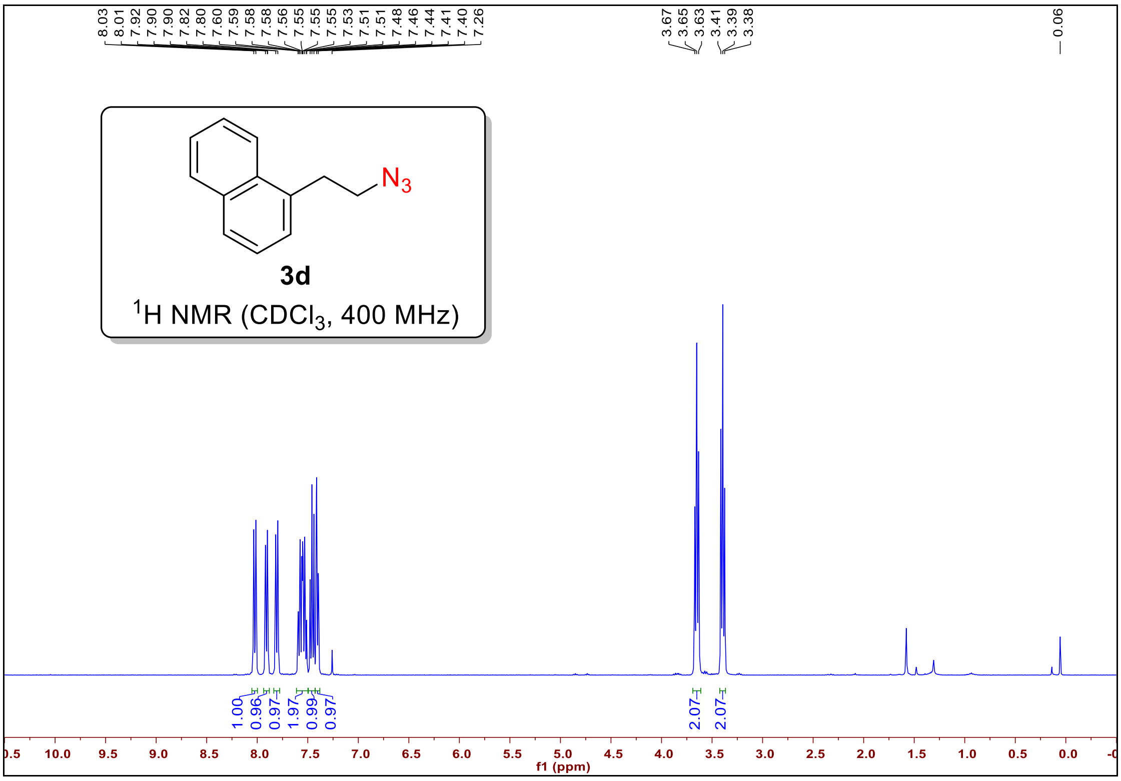

Supplementary Figure 23. ^{1}**H NMR Spectrum of 3d**

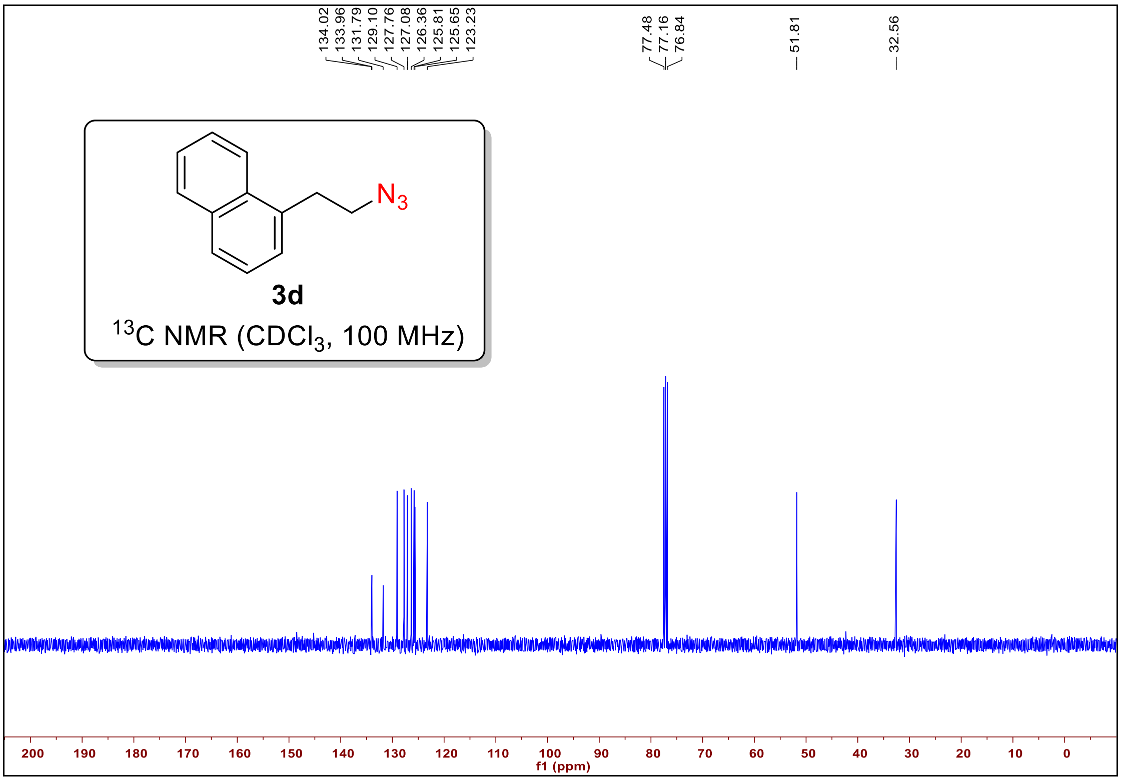

Supplementary Figure 24. ^{13}**C NMR Spectrum of 3d**

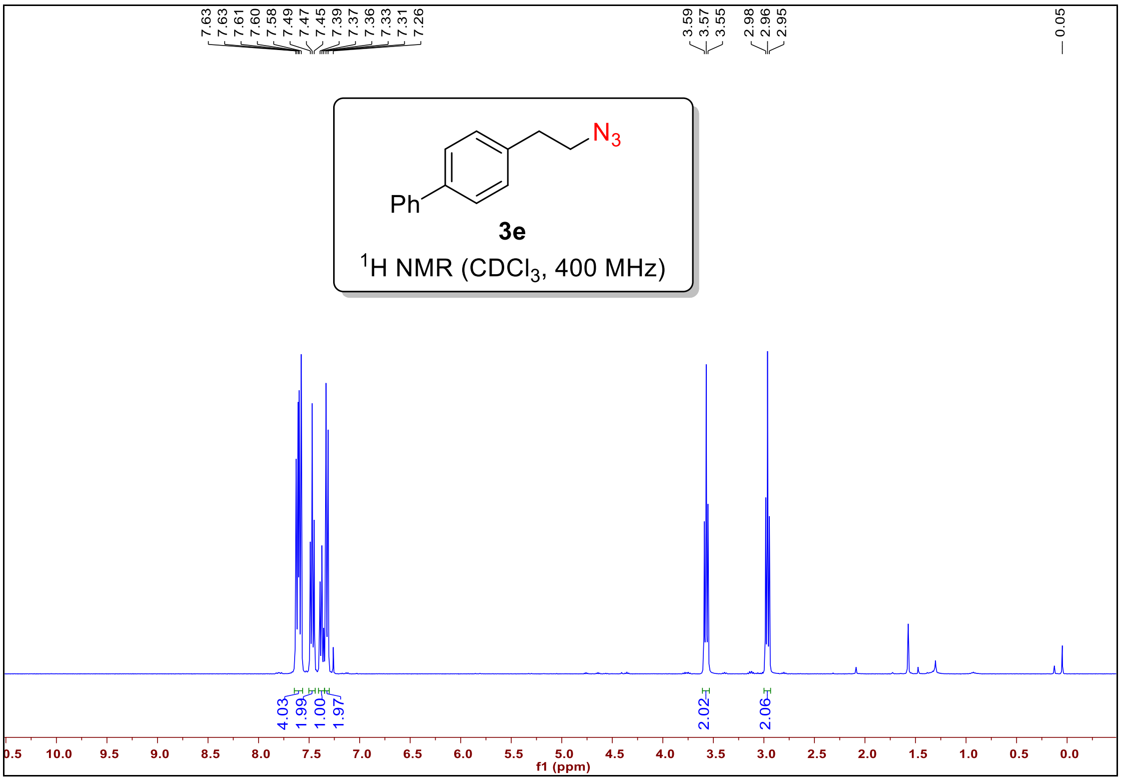

Supplementary Figure 25. ^{1}H NMR Spectrum of 3e

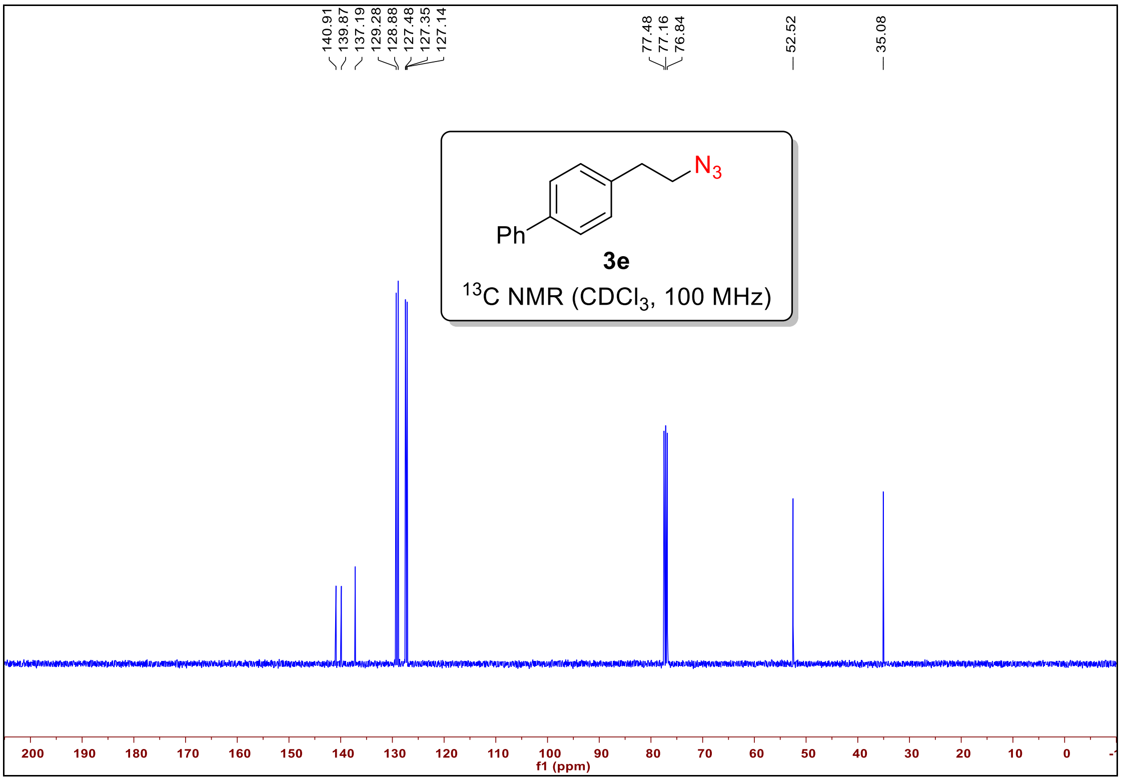

Supplementary Figure 26. ^{13}C NMR Spectrum of 3e

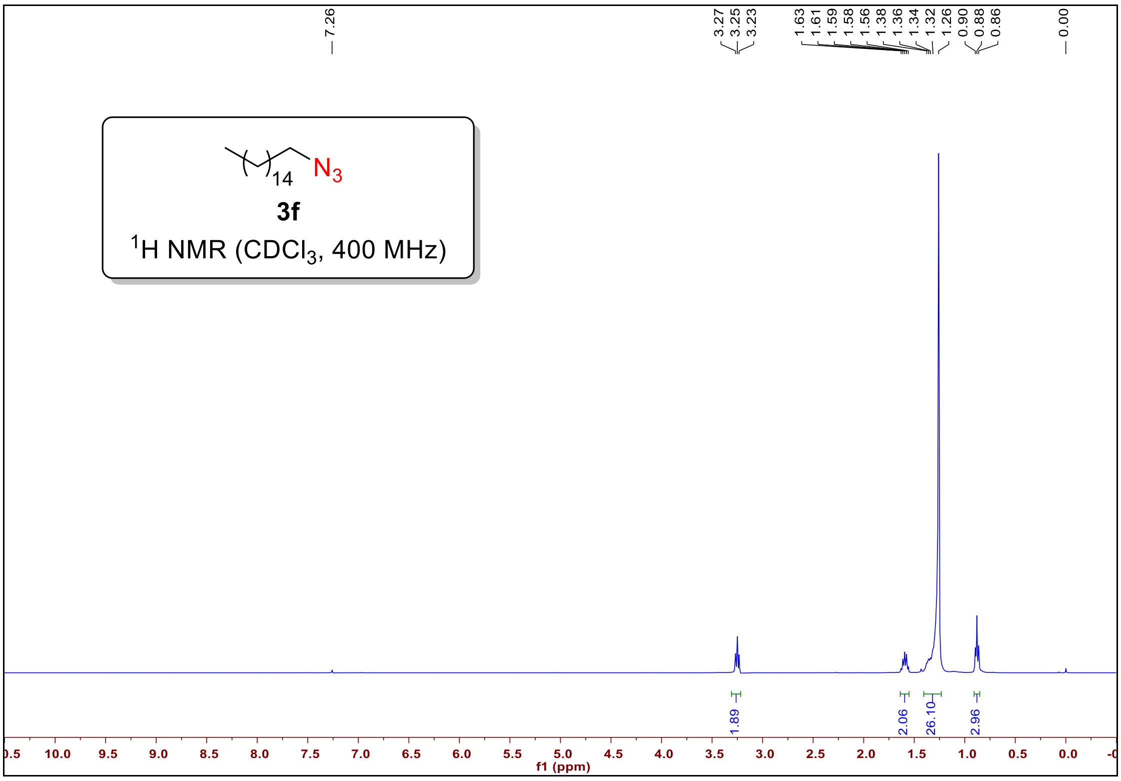

Supplementary Figure 27. ^{1}H NMR Spectrum of 3f

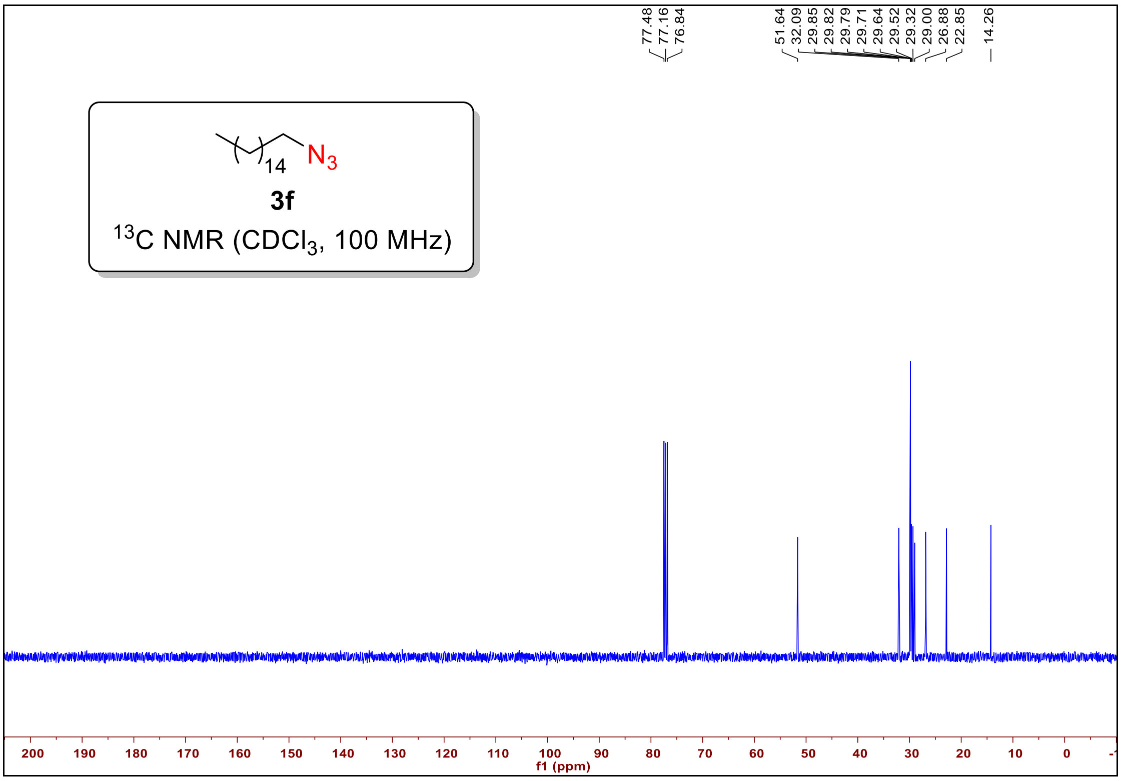

Supplementary Figure 28. ^{13}C NMR Spectrum of 3f

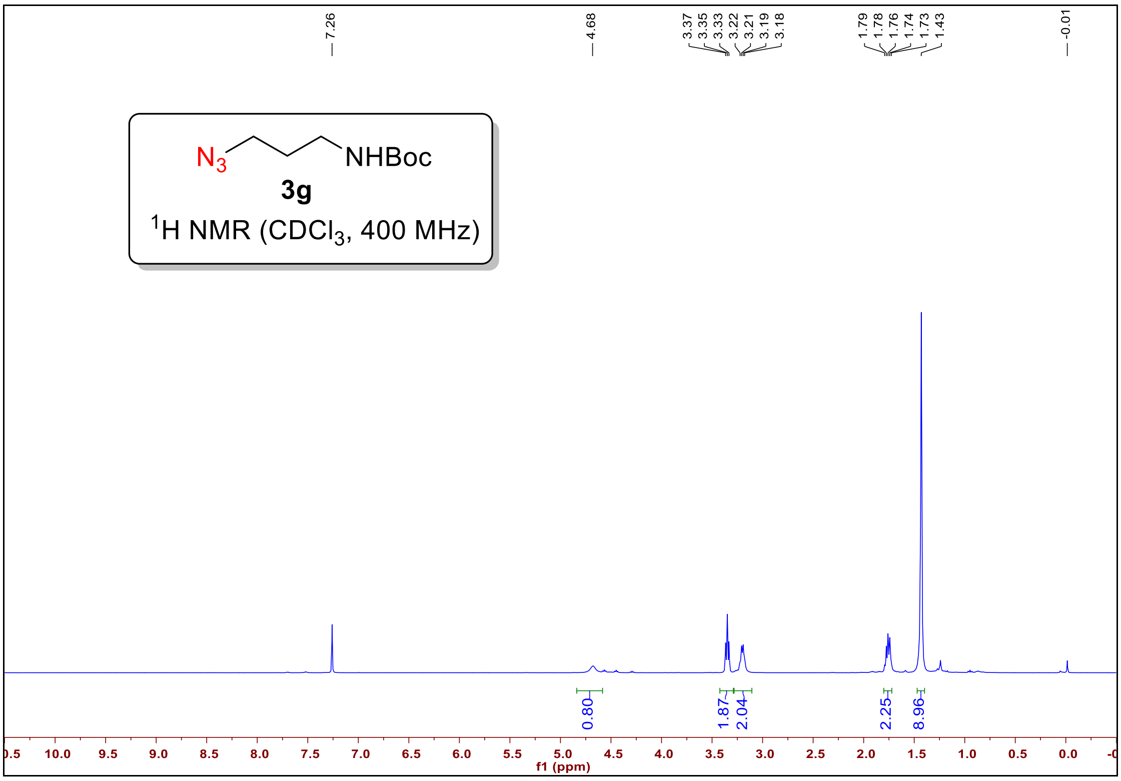

Supplementary Figure 29. **^{1}H NMR Spectrum of 3g**

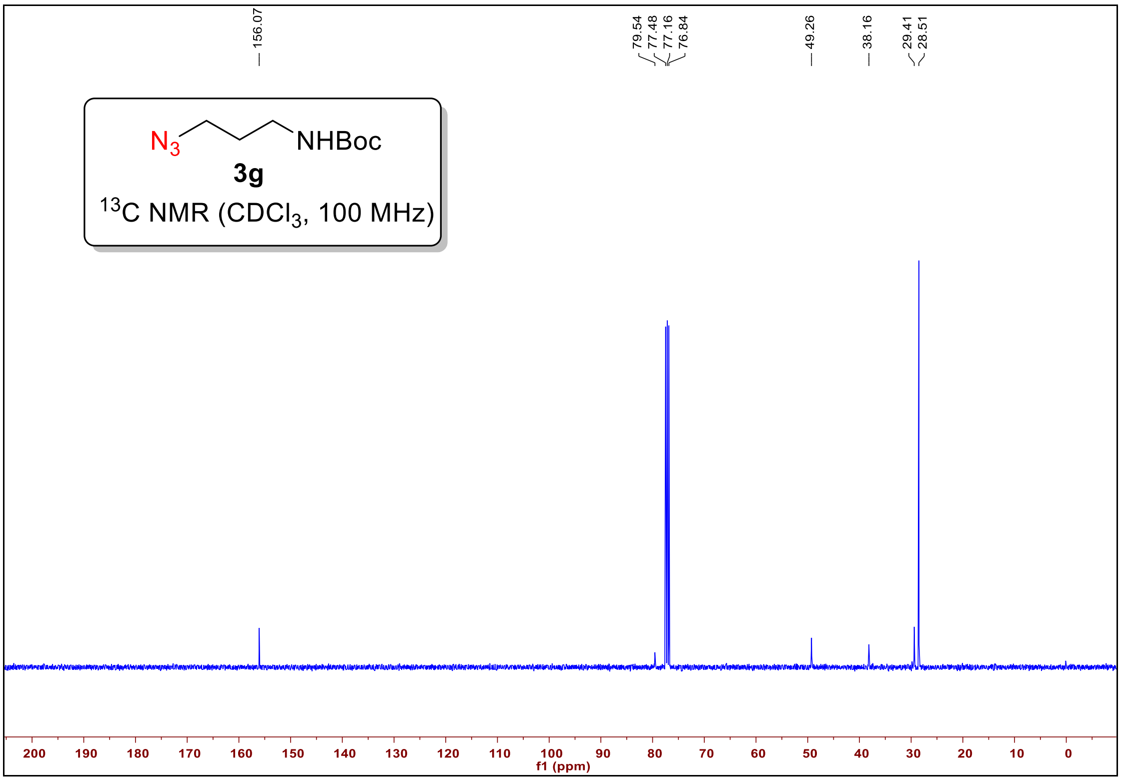

Supplementary Figure 30. **^{13}C NMR Spectrum of 3g**

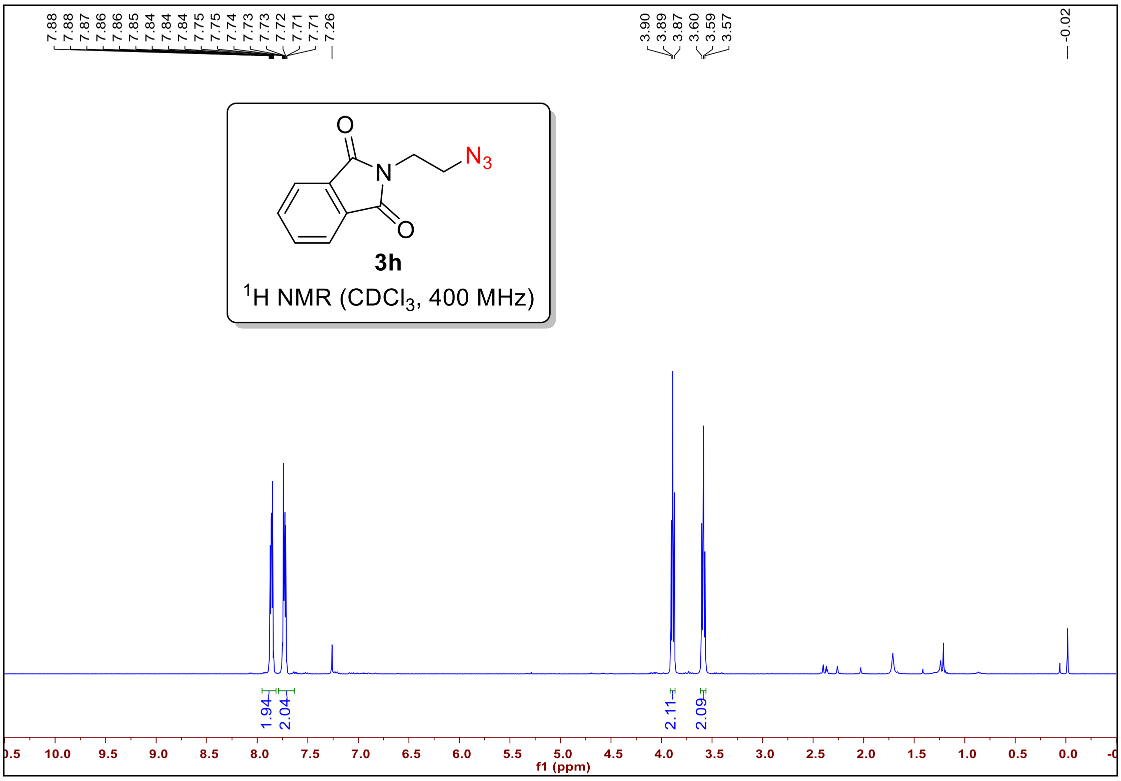

Supplementary Figure 31. ^{1}**H NMR Spectrum of 3h**

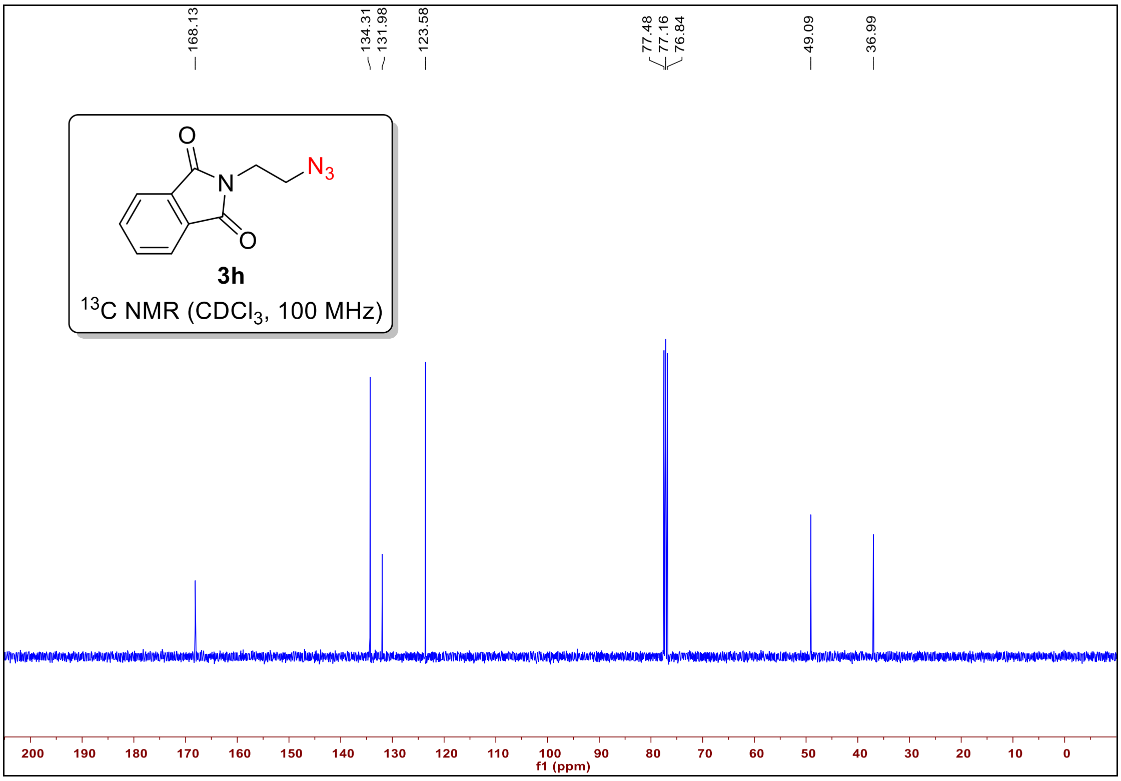

Supplementary Figure 32. ^{13}**C NMR Spectrum of 3h**

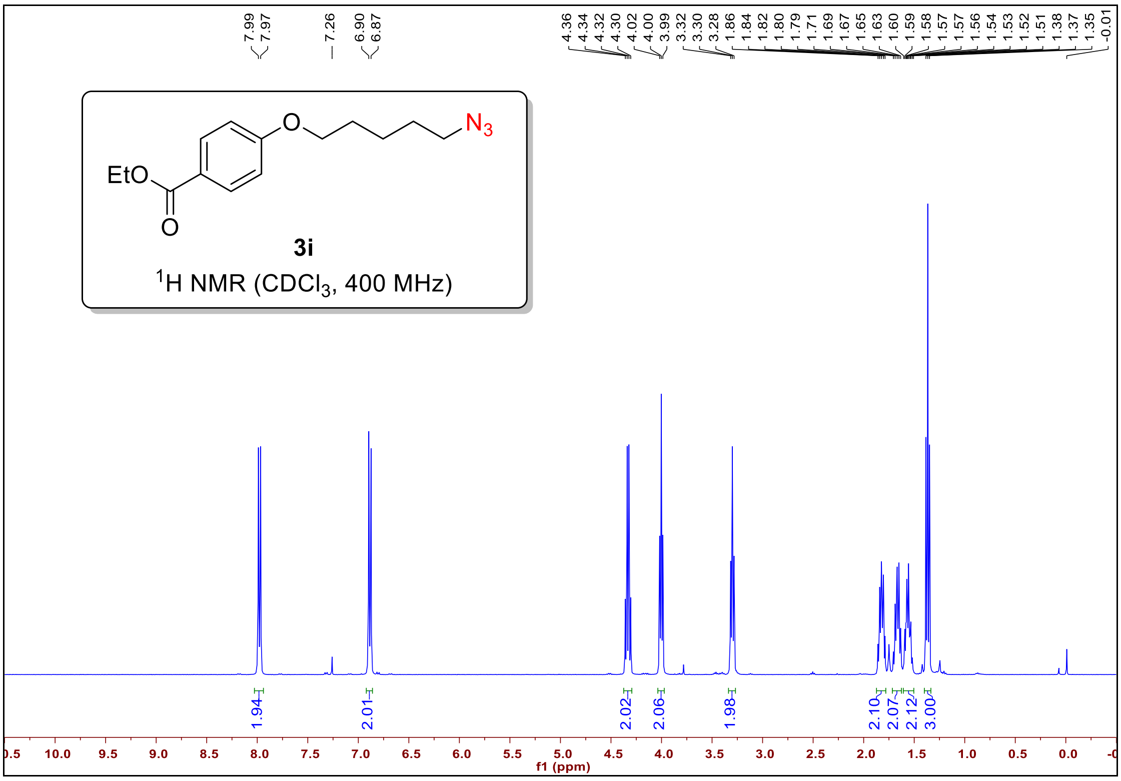

Supplementary Figure 33. ^{1}H NMR Spectrum of 3i

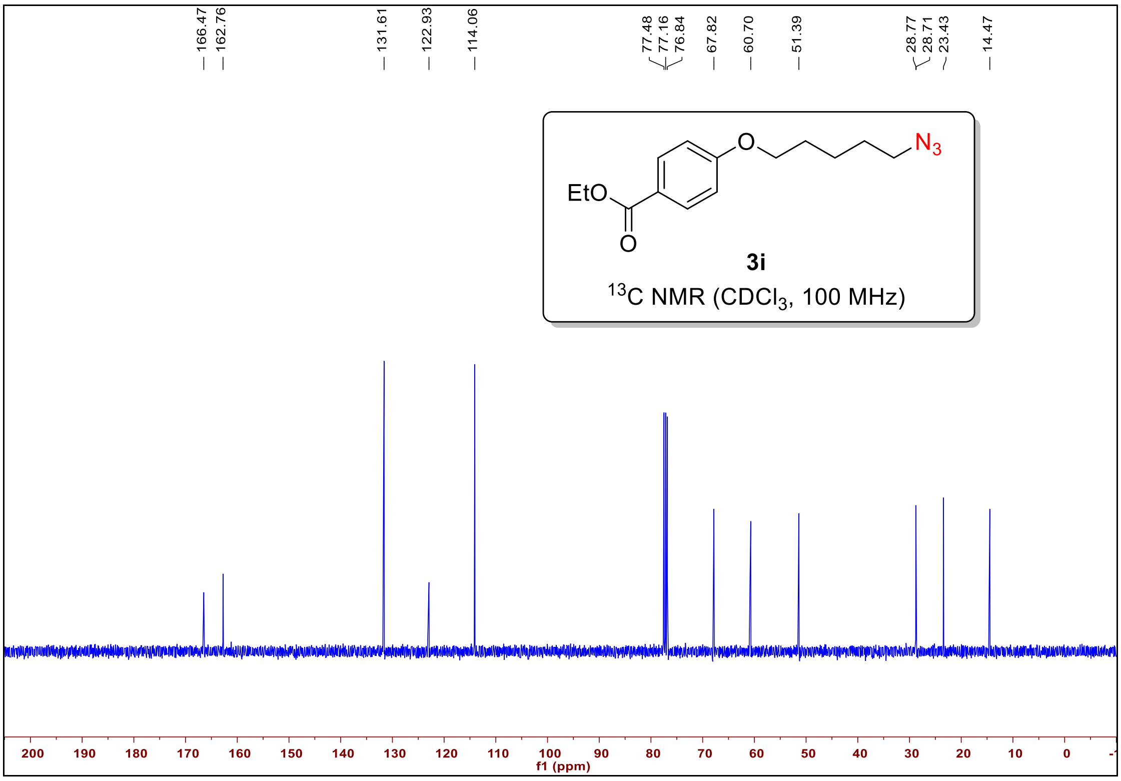

Supplementary Figure 34. ^{13}C NMR Spectrum of 3i

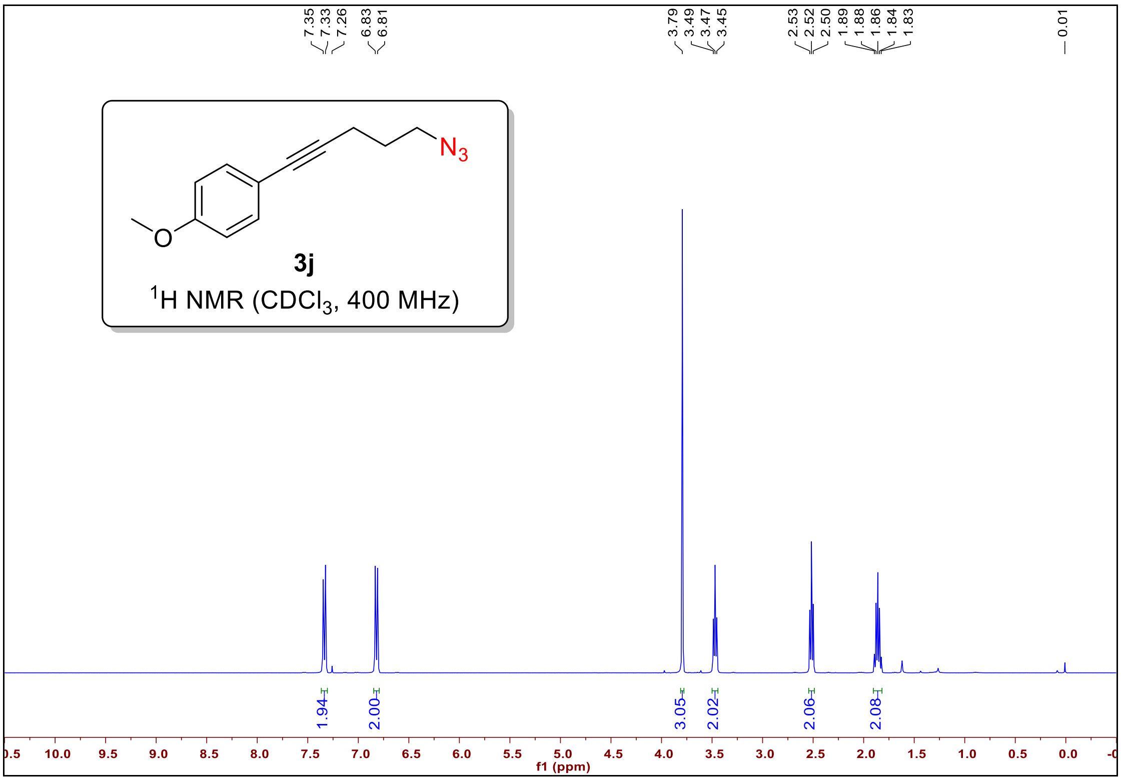

Supplementary Figure 35. ^{1}**H NMR Spectrum of 3j**

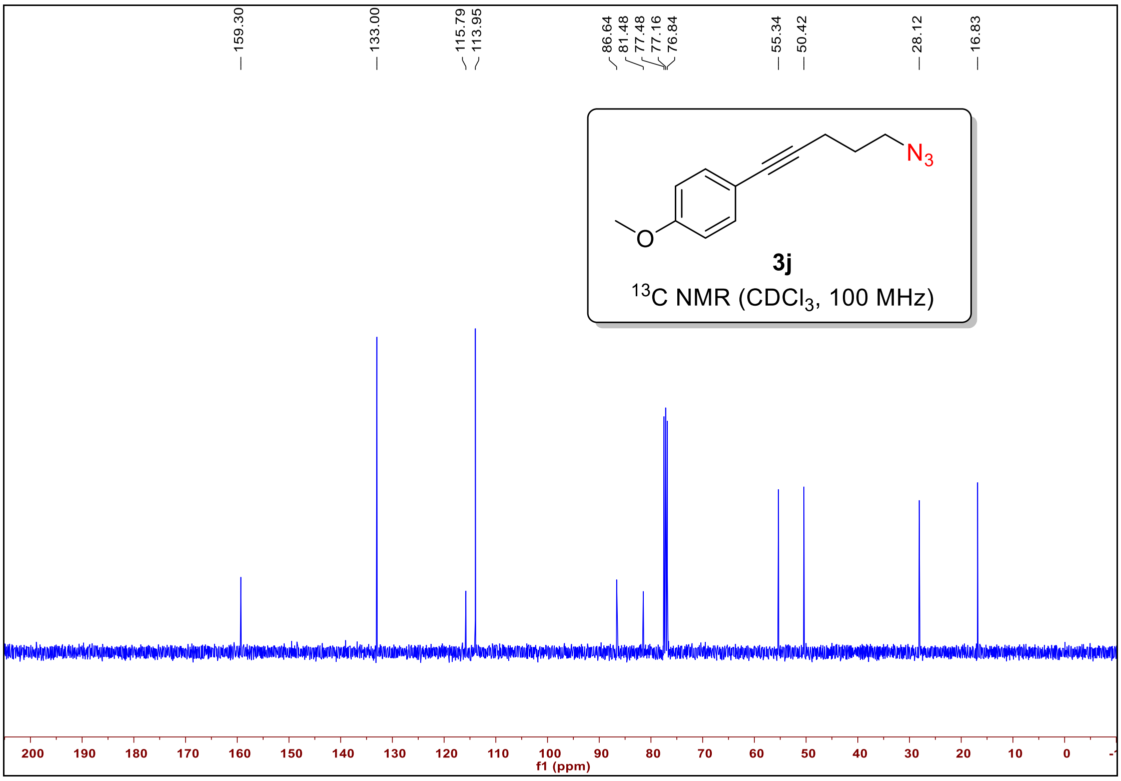

Supplementary Figure 36. ^{13}**C NMR Spectrum of 3j**

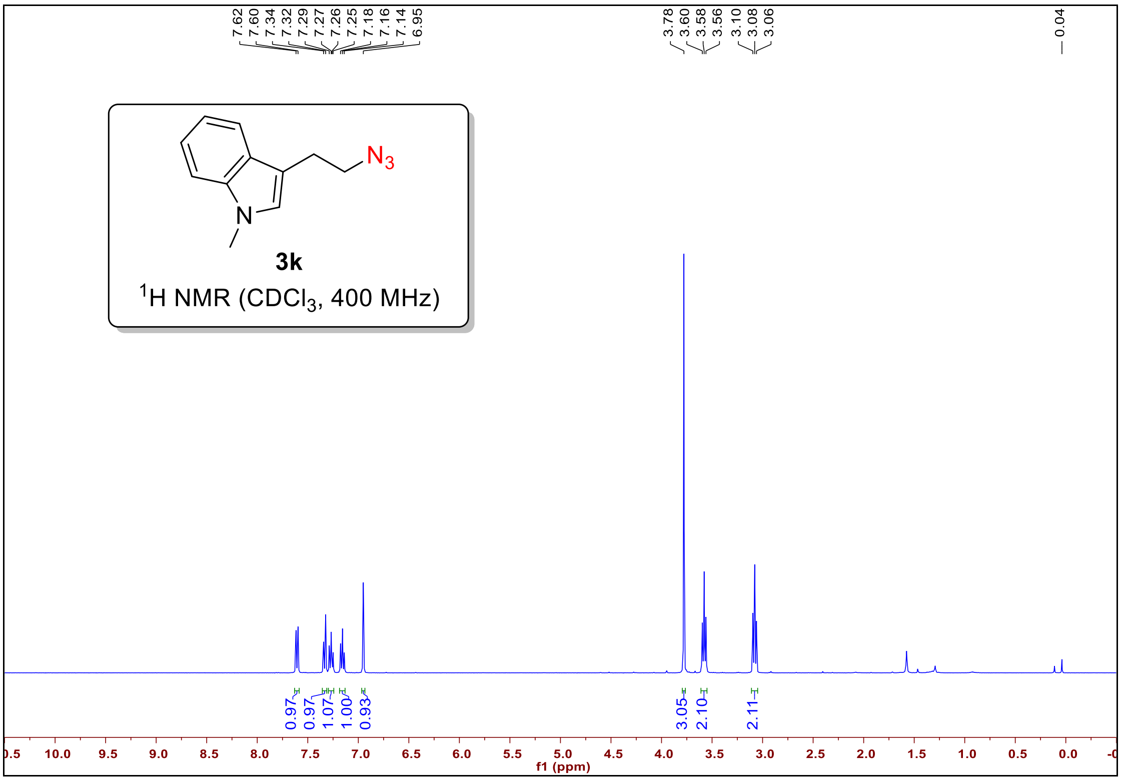

Supplementary Figure 37. ^{1}H NMR Spectrum of 3k

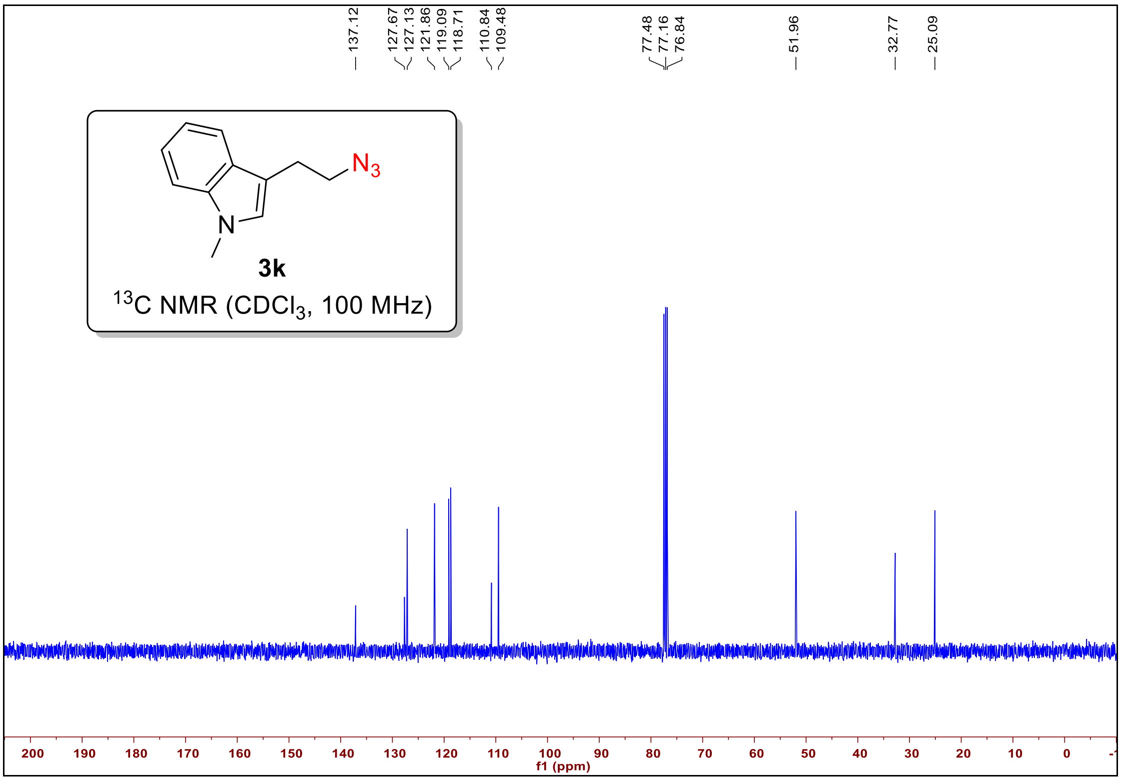

Supplementary Figure 38. ^{13}C NMR Spectrum of 3k

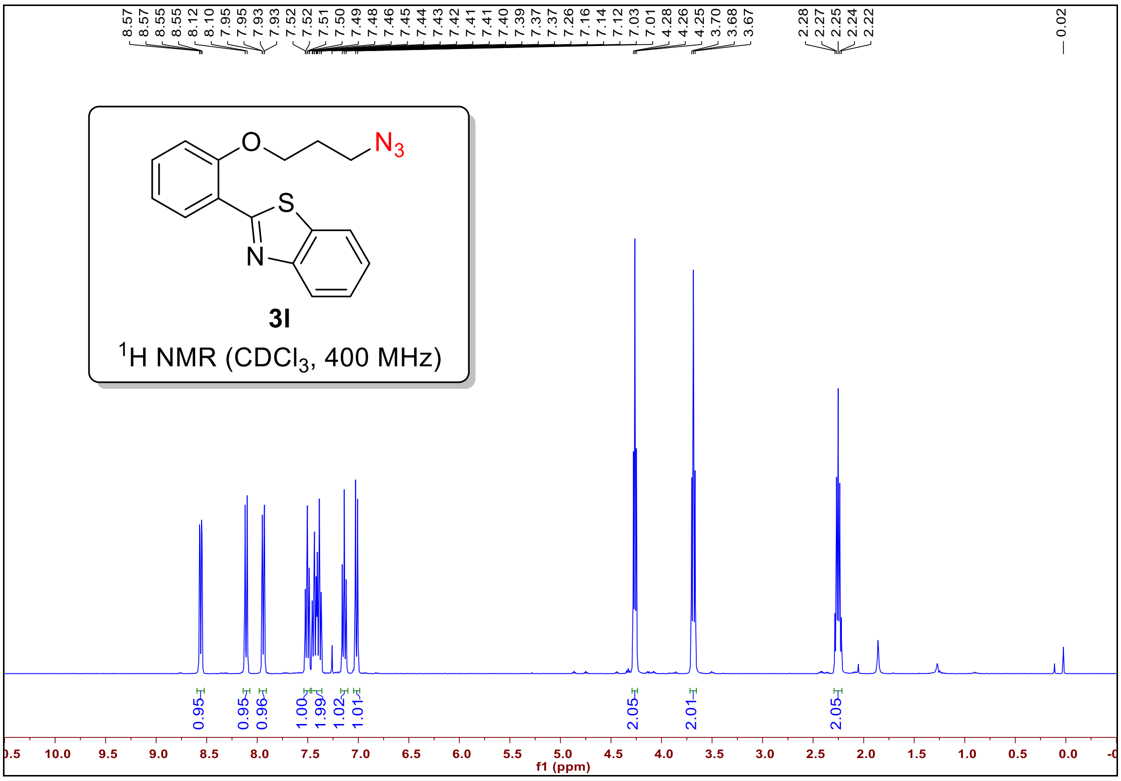

Supplementary Figure 39. ^{1}H NMR Spectrum of 3l

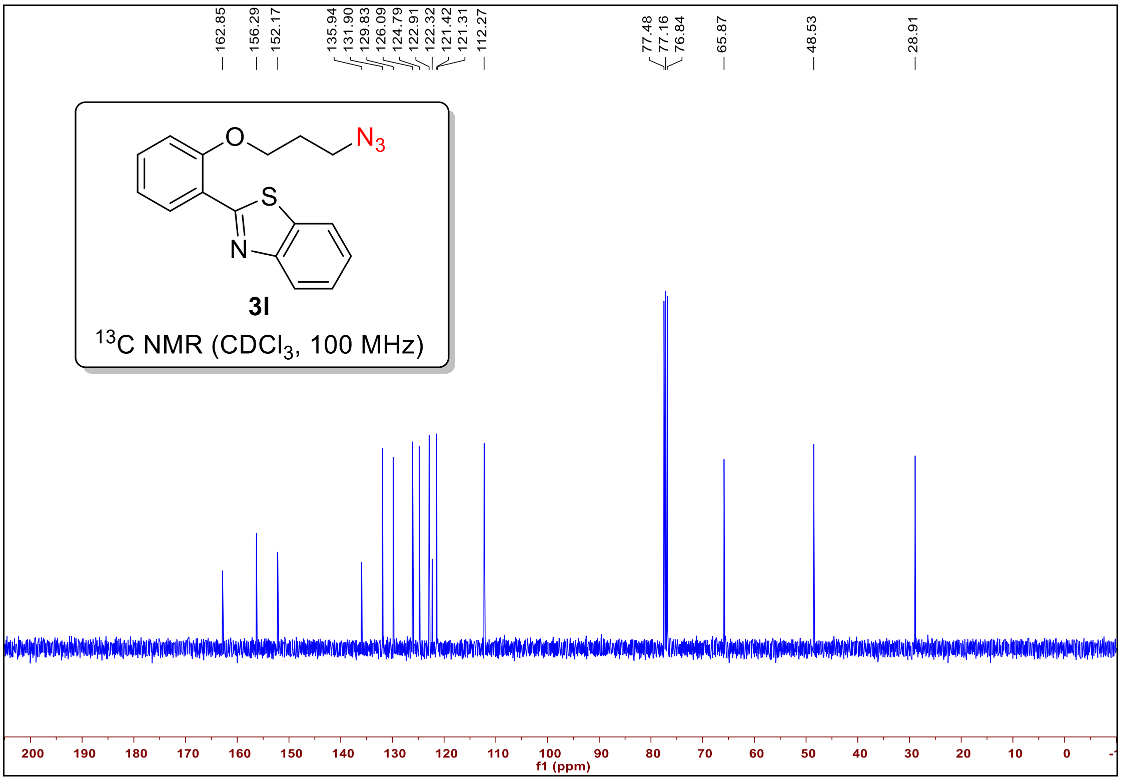

Supplementary Figure 40. ^{13}C NMR Spectrum of 3l

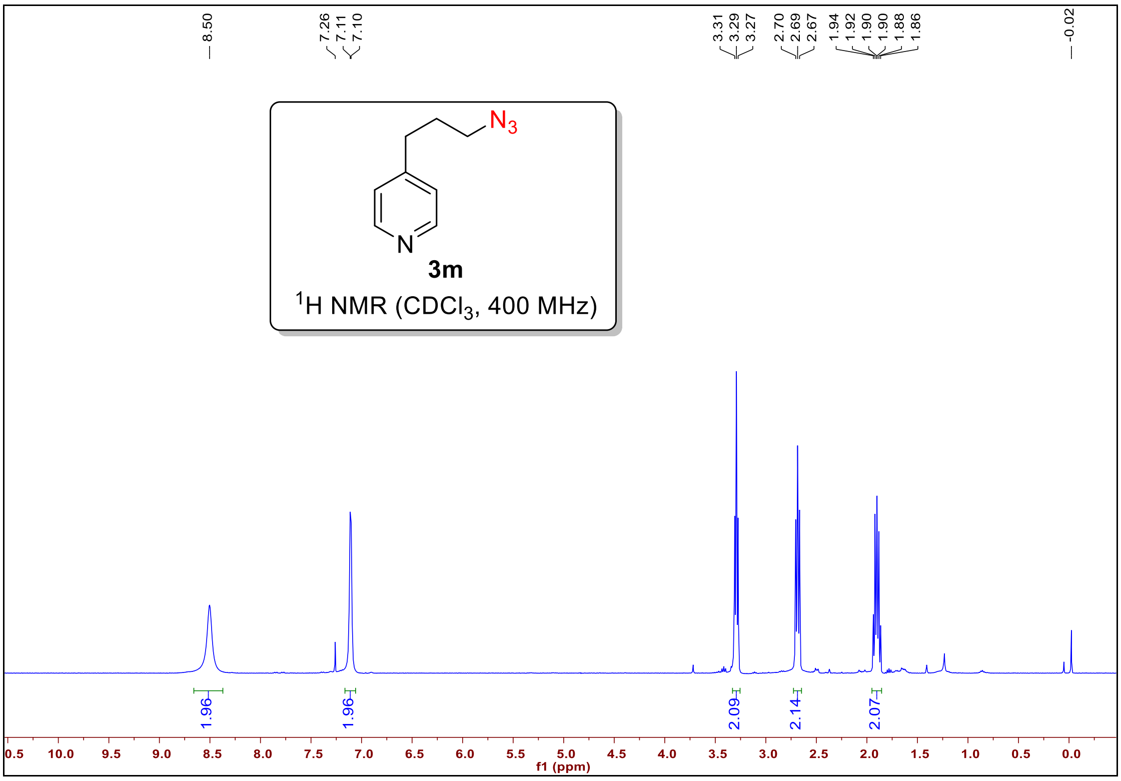

Supplementary Figure 41. ^{1}H NMR Spectrum of 3m

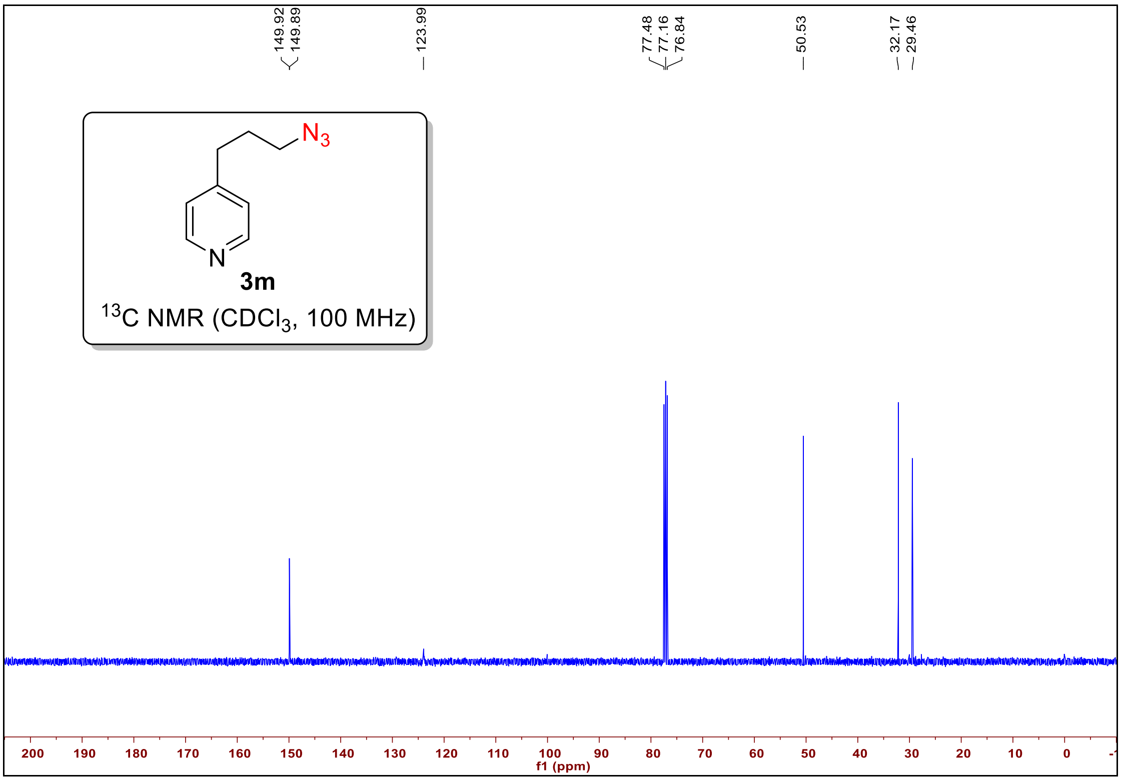

Supplementary Figure 42. ^{13}C NMR Spectrum of 3m

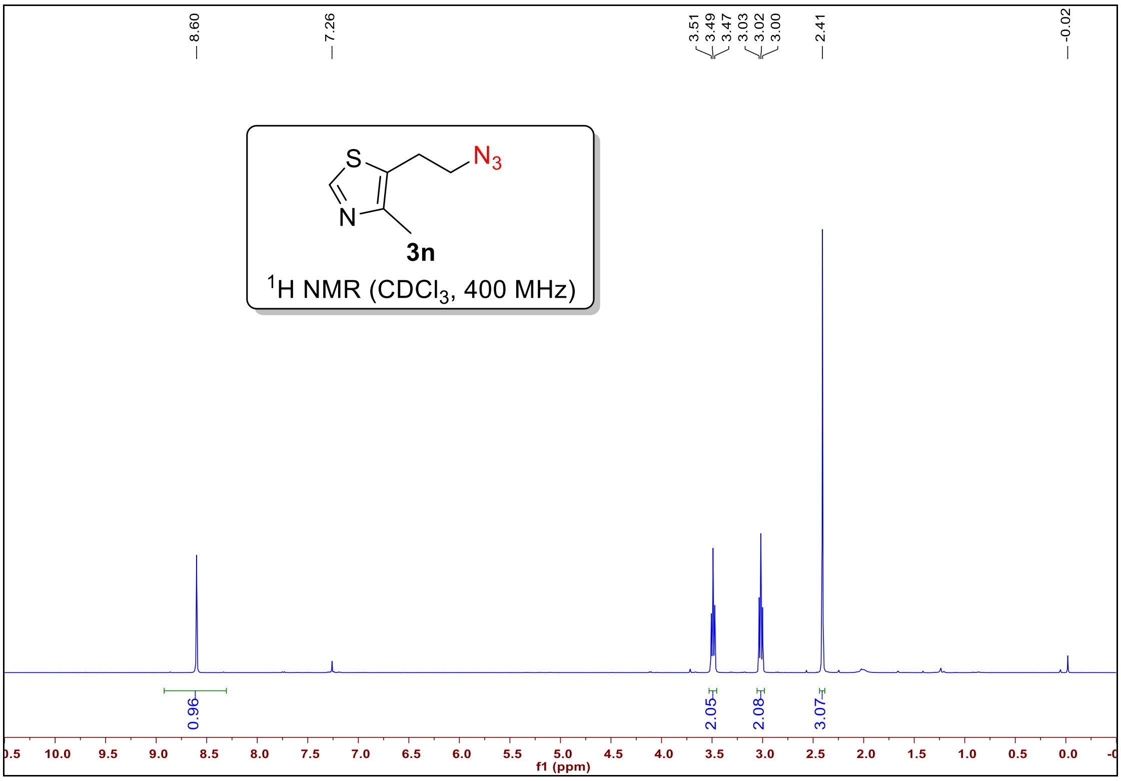

Supplementary Figure 43. ^{1}H NMR Spectrum of 3n

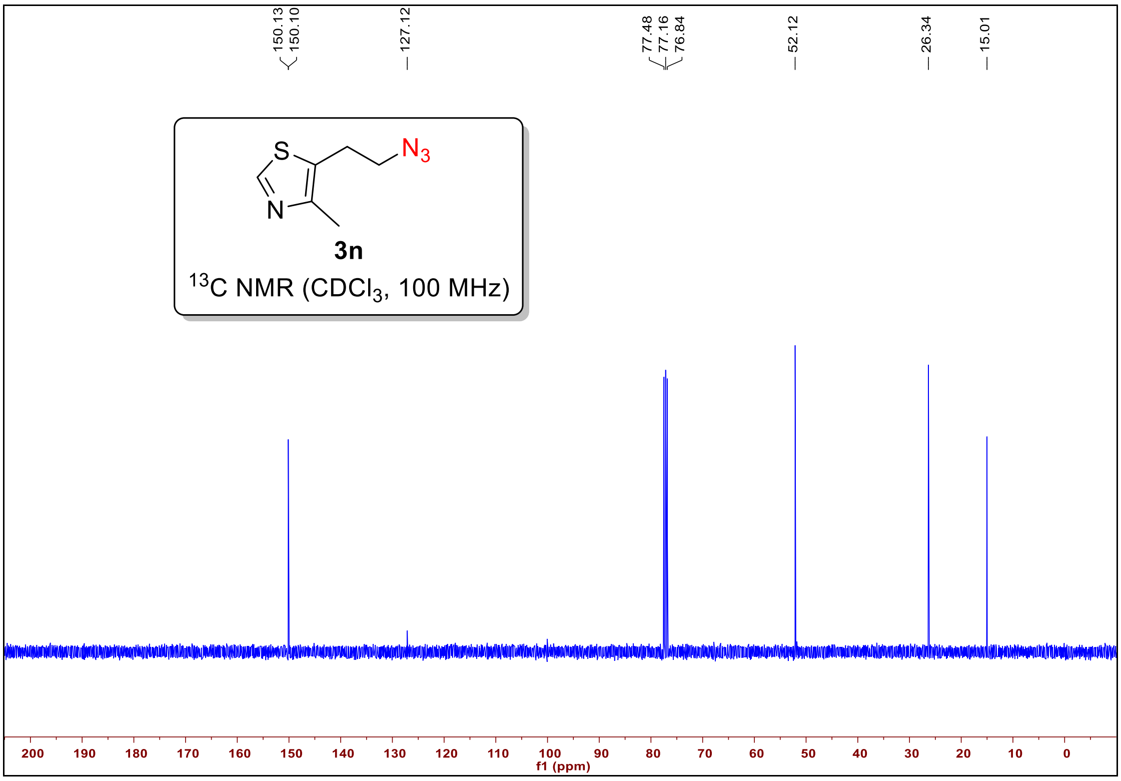

Supplementary Figure 44. ^{13}C NMR Spectrum of 3n

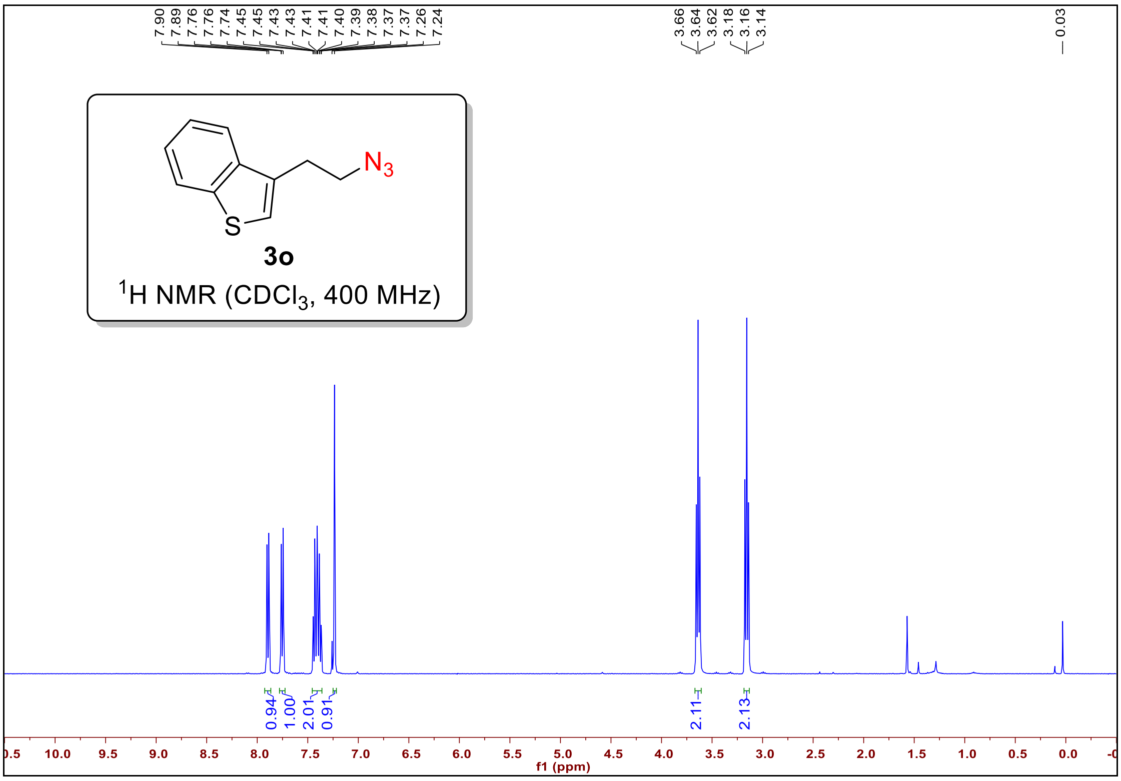

Supplementary Figure 45. ^{1}H NMR Spectrum of 3o

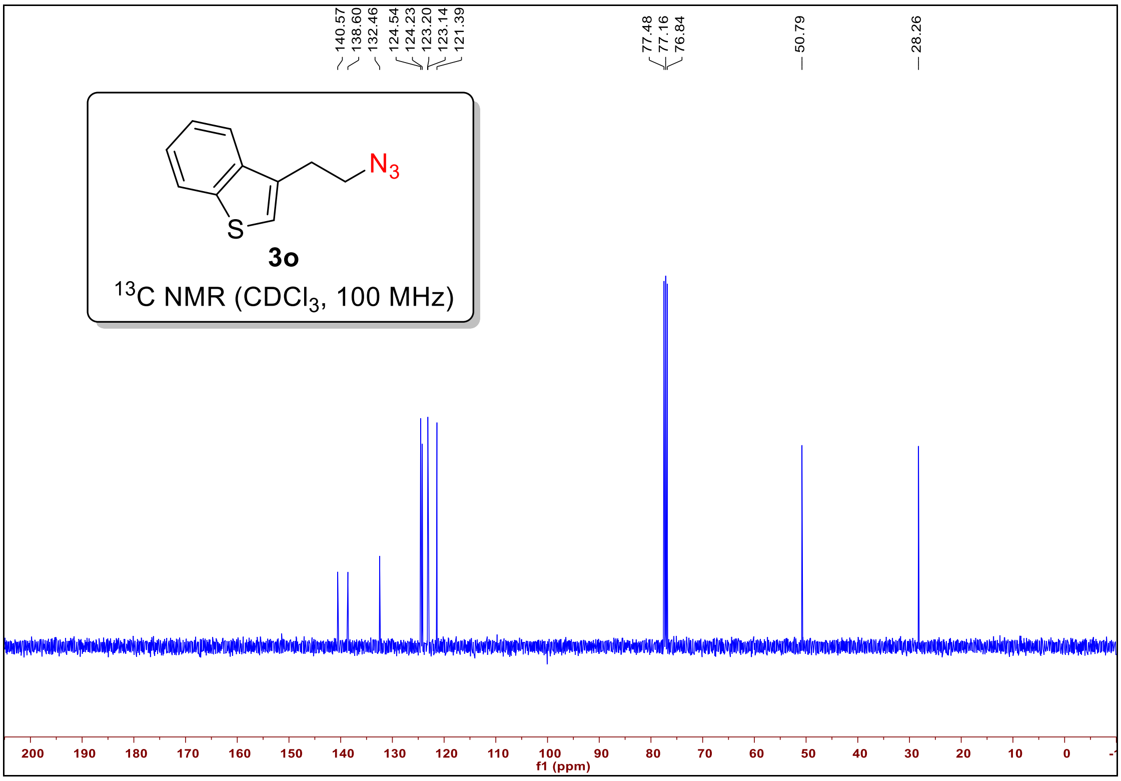

Supplementary Figure 46. ^{13}C NMR Spectrum of 3o

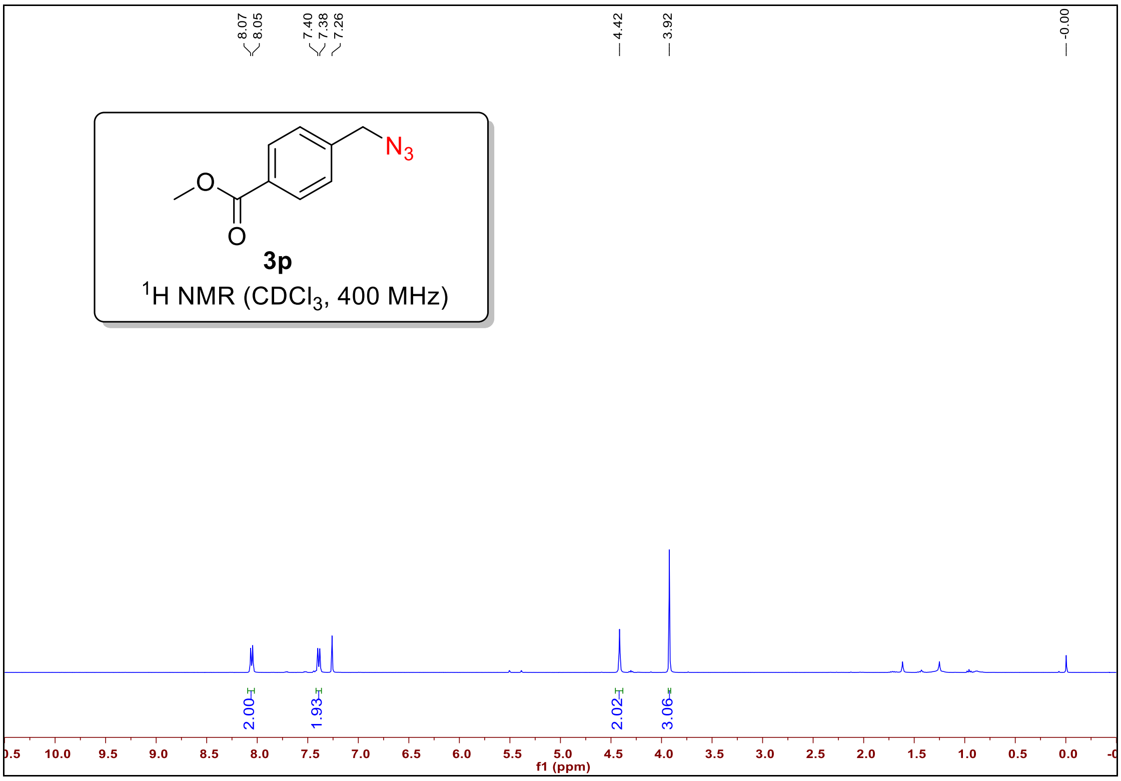

Supplementary Figure 47. ^{1}H NMR Spectrum of 3p

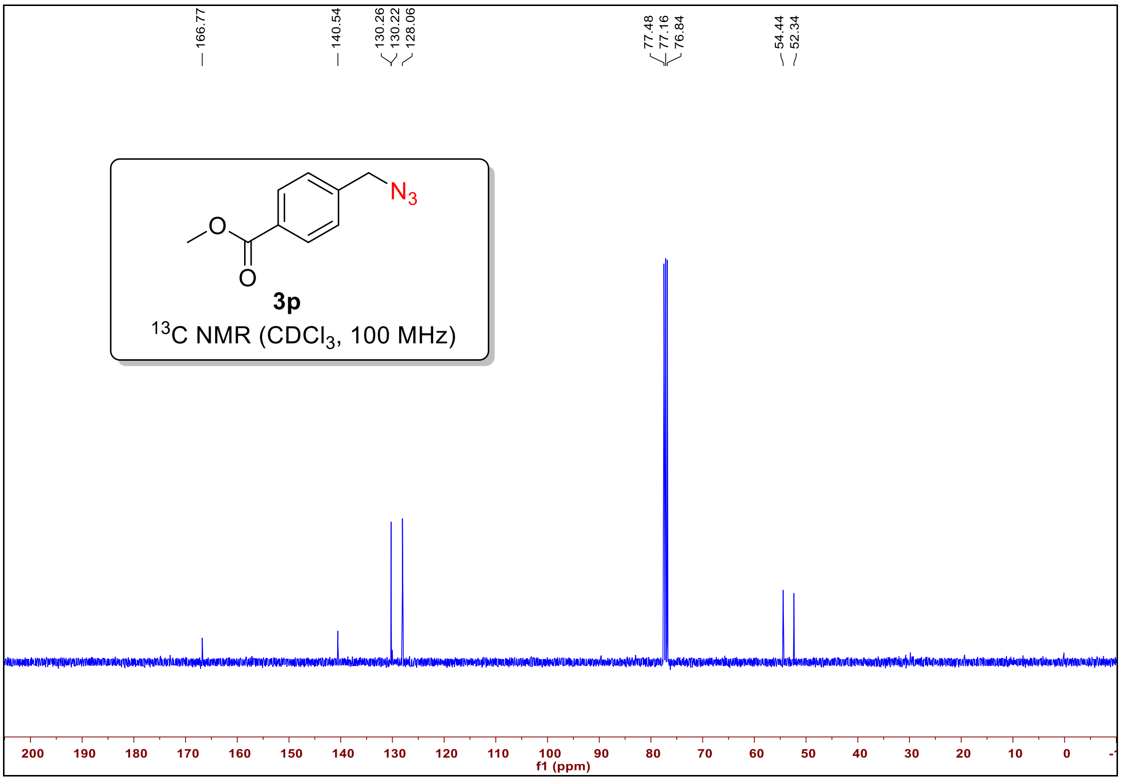

Supplementary Figure 48. ^{13}C NMR Spectrum of 3p

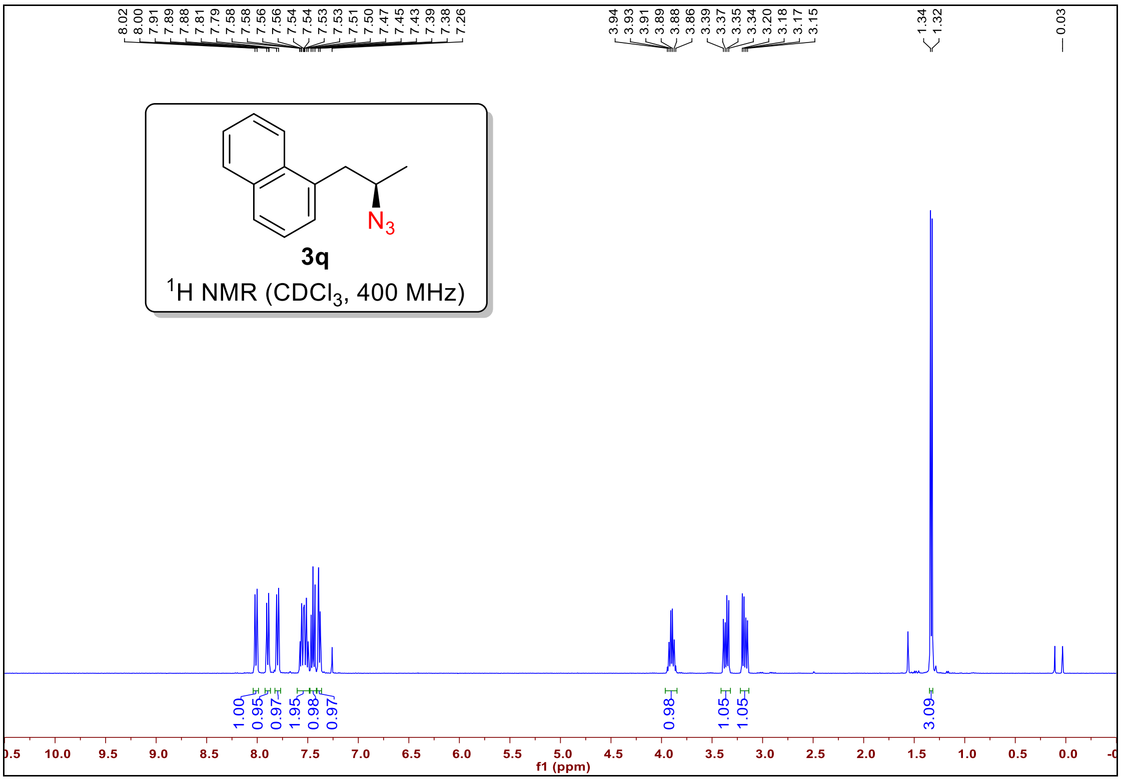

Supplementary Figure 49. ^{1}H NMR Spectrum of 3q

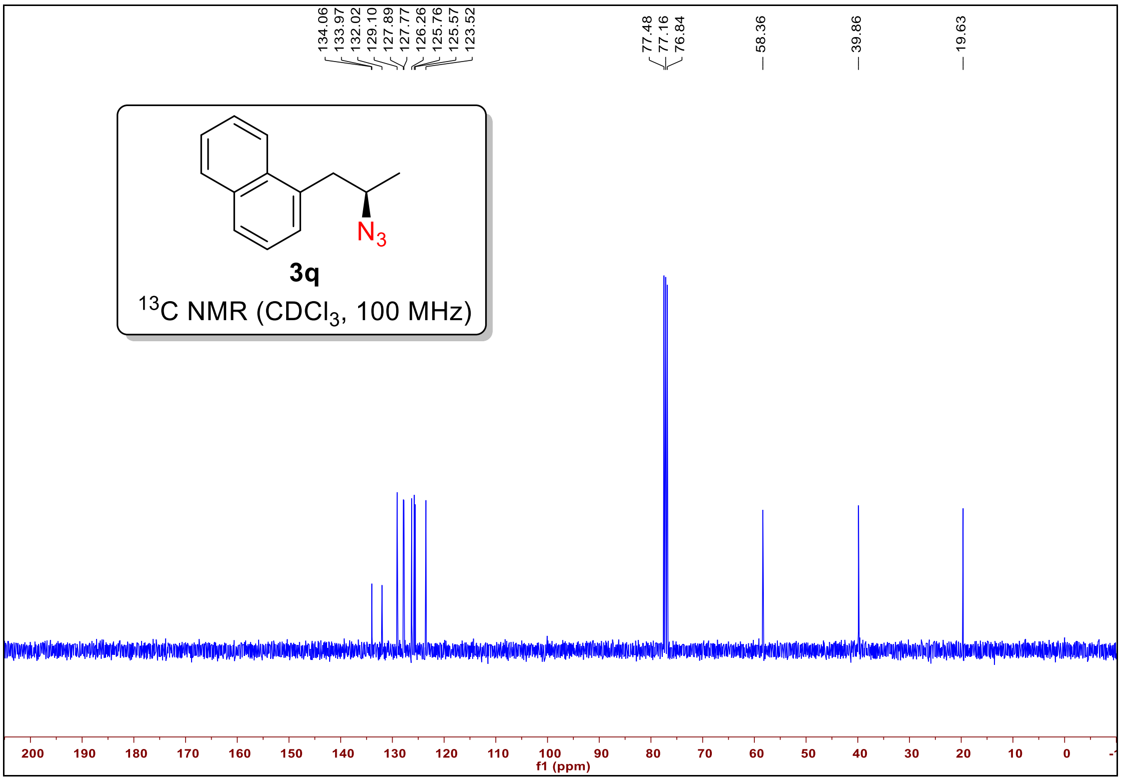

Supplementary Figure 50. ^{13}C NMR Spectrum of 3q

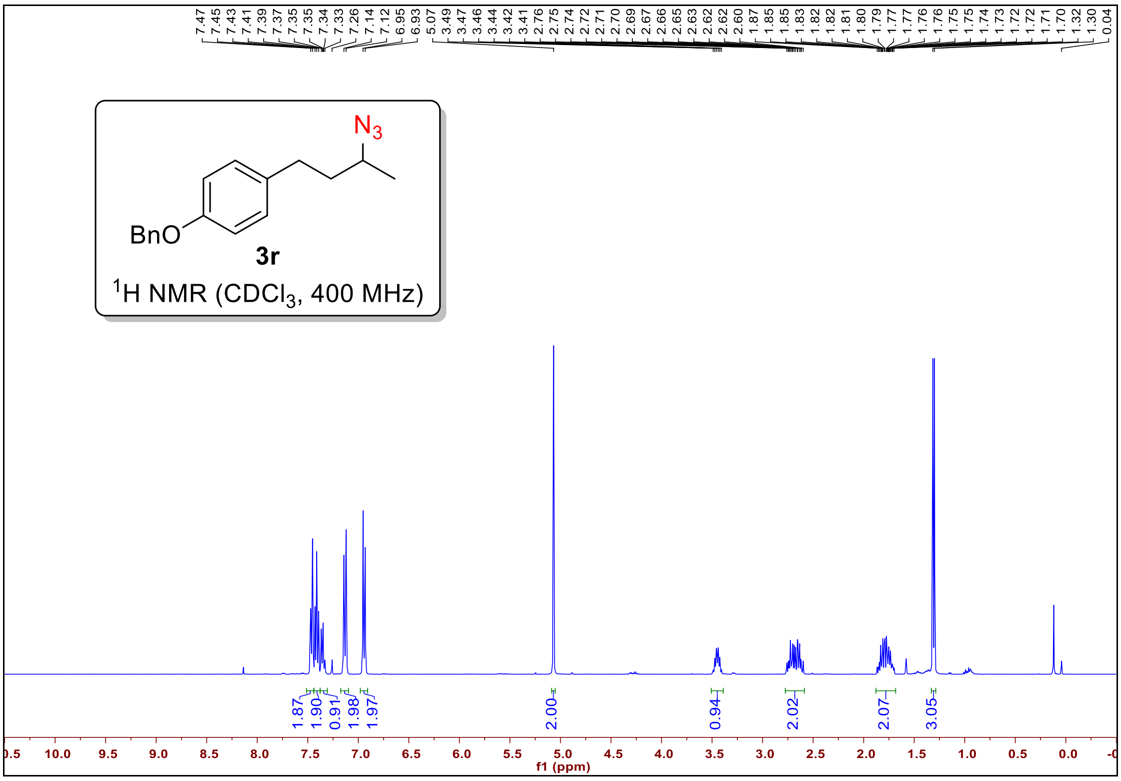

Supplementary Figure 51. ^{1}H NMR Spectrum of 3r

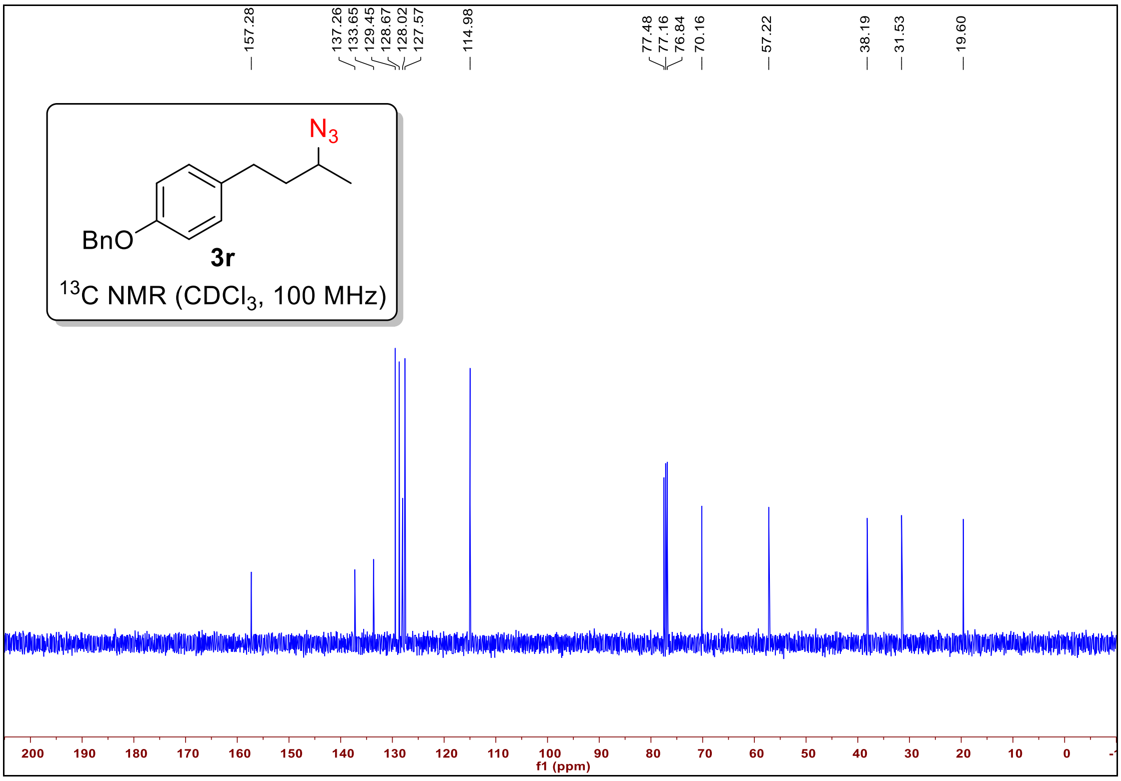

Supplementary Figure 52. ^{13}C NMR Spectrum of 3r

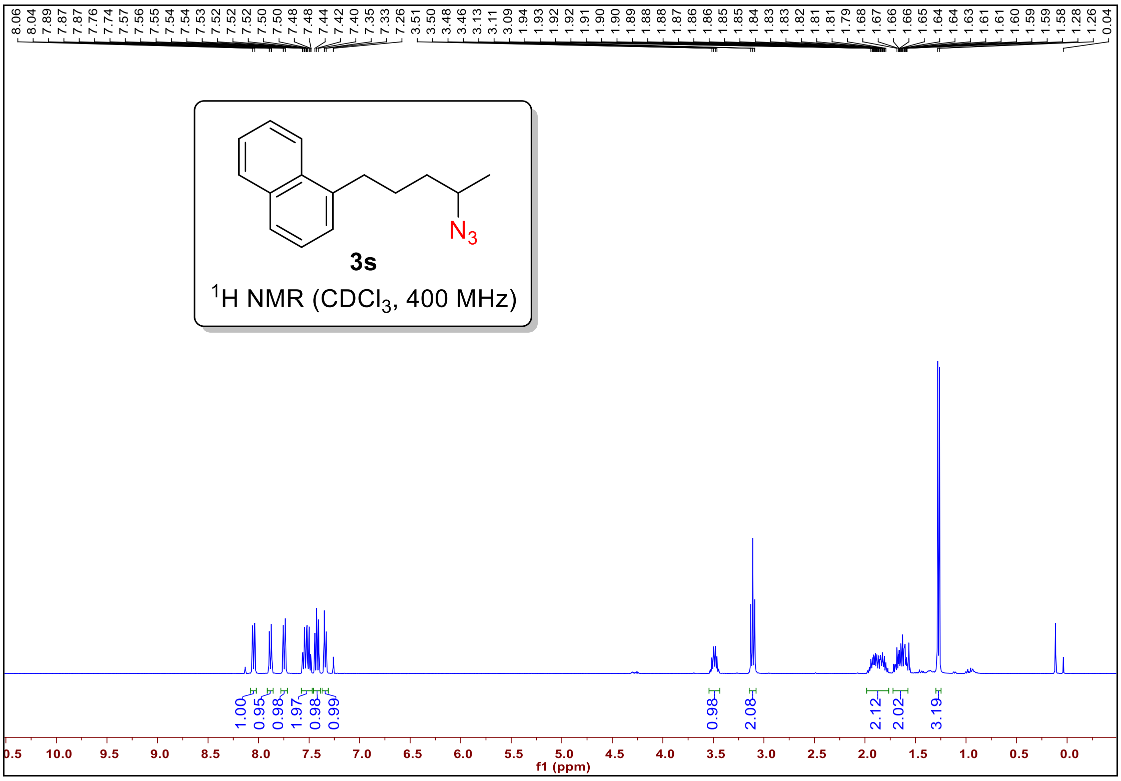

Supplementary Figure 53. ^{1}H NMR Spectrum of 3s

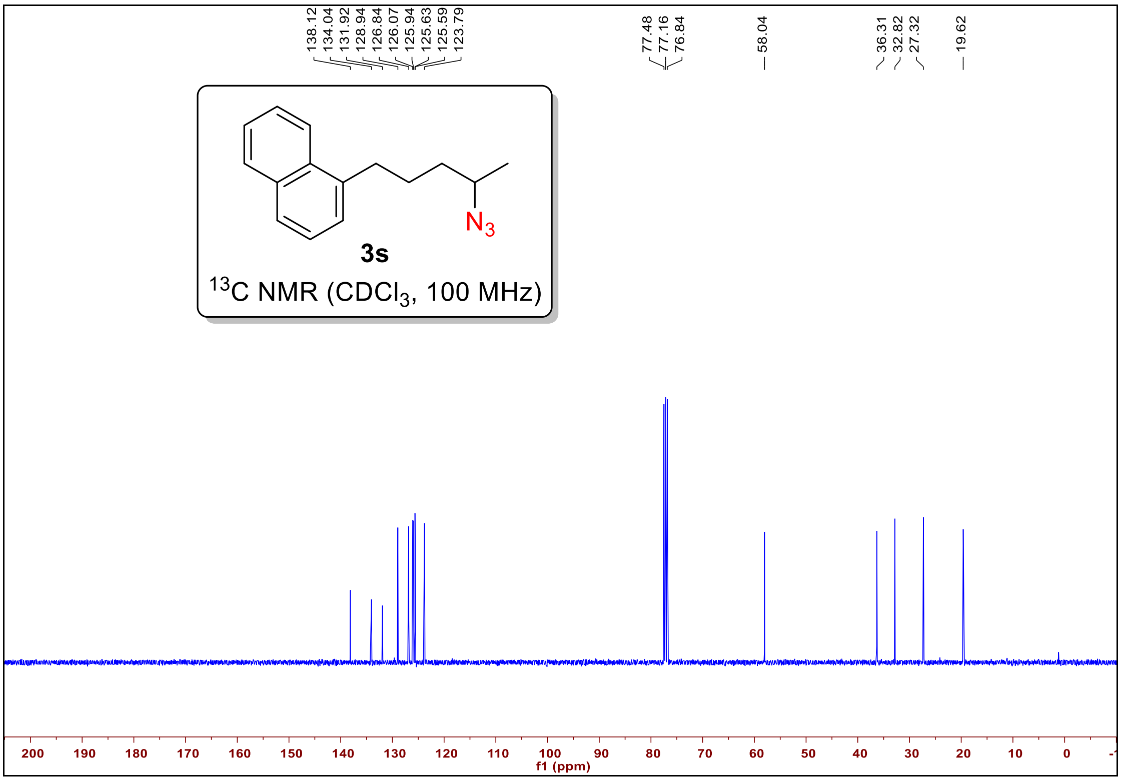

Supplementary Figure 54. ^{13}C NMR Spectrum of 3s

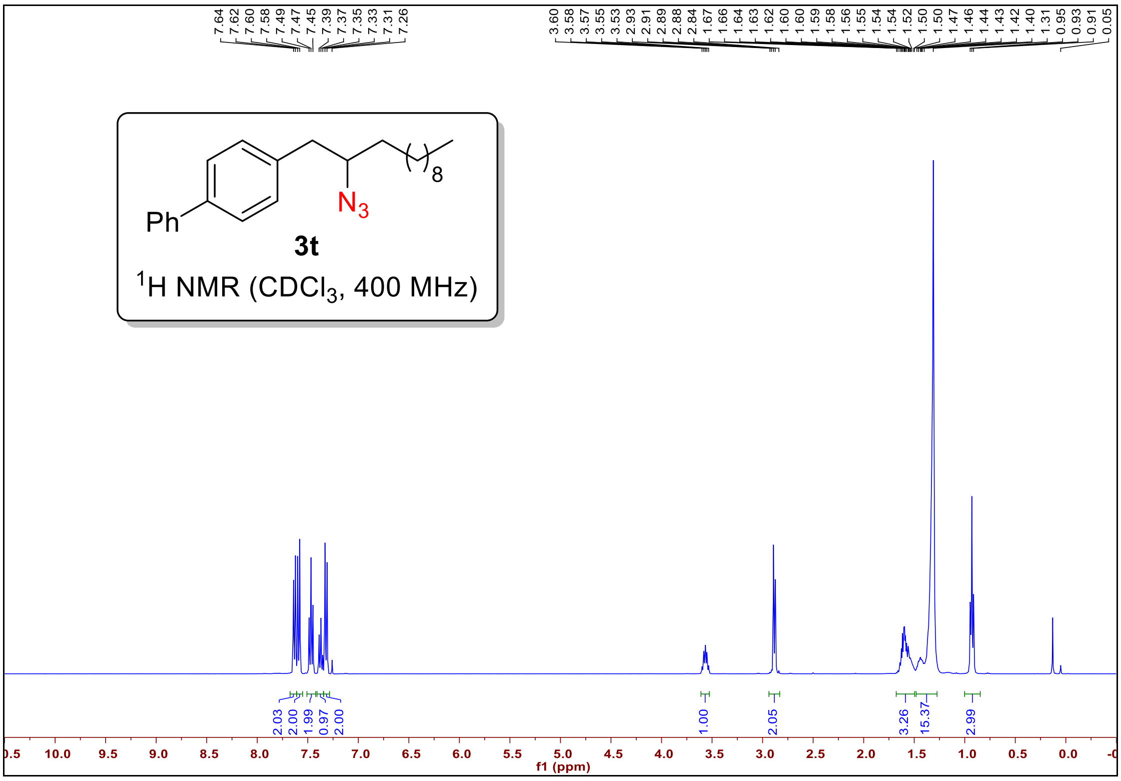

Supplementary Figure 55. ^{1}H NMR Spectrum of 3t

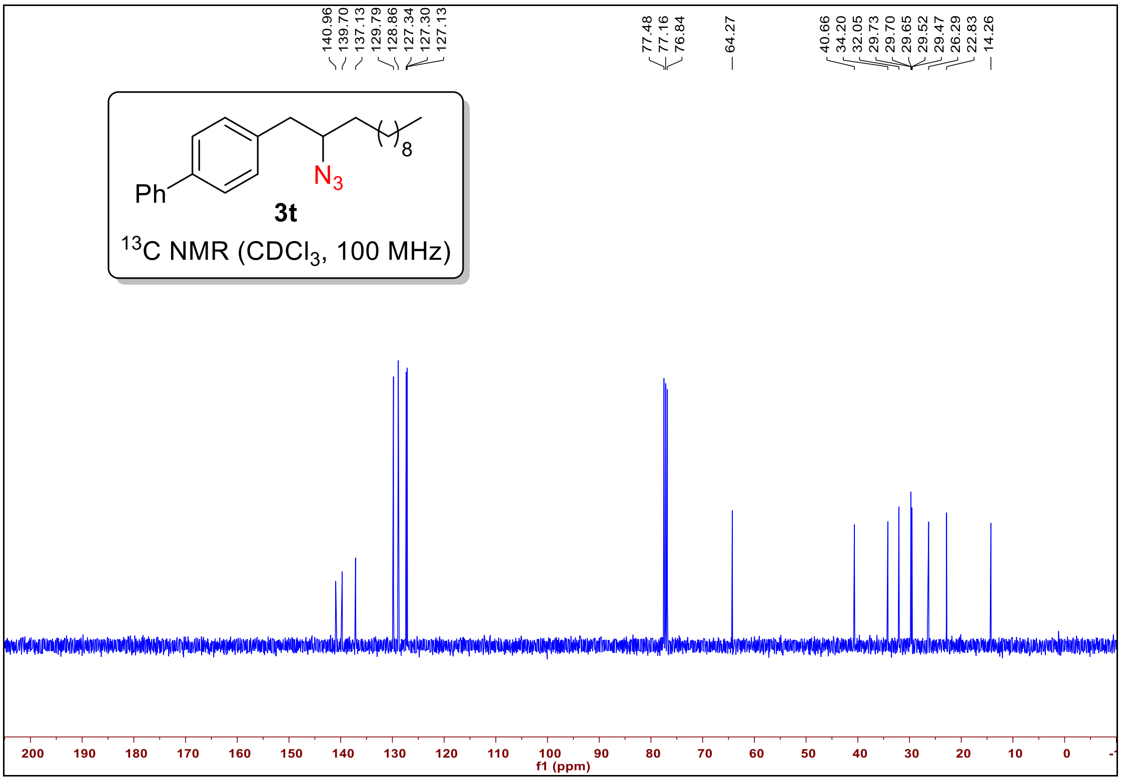

Supplementary Figure 56. ^{13}C NMR Spectrum of 3t

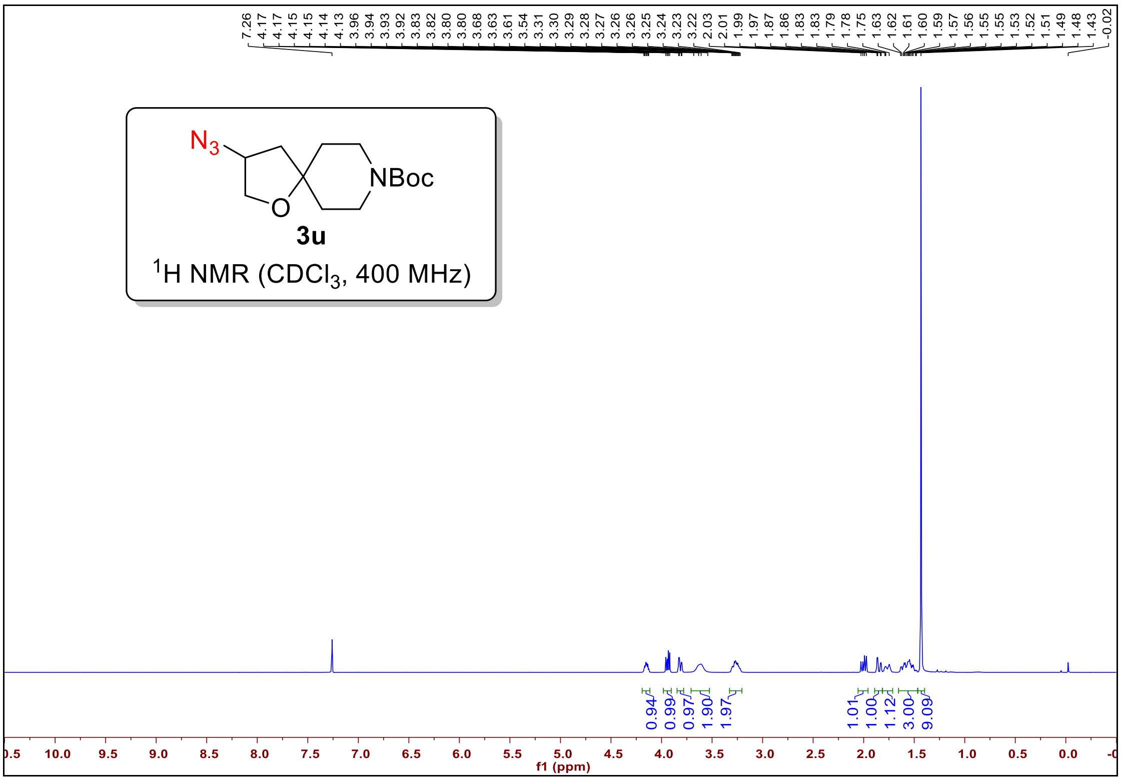

Supplementary Figure 57. ^{1}H NMR Spectrum of 3u

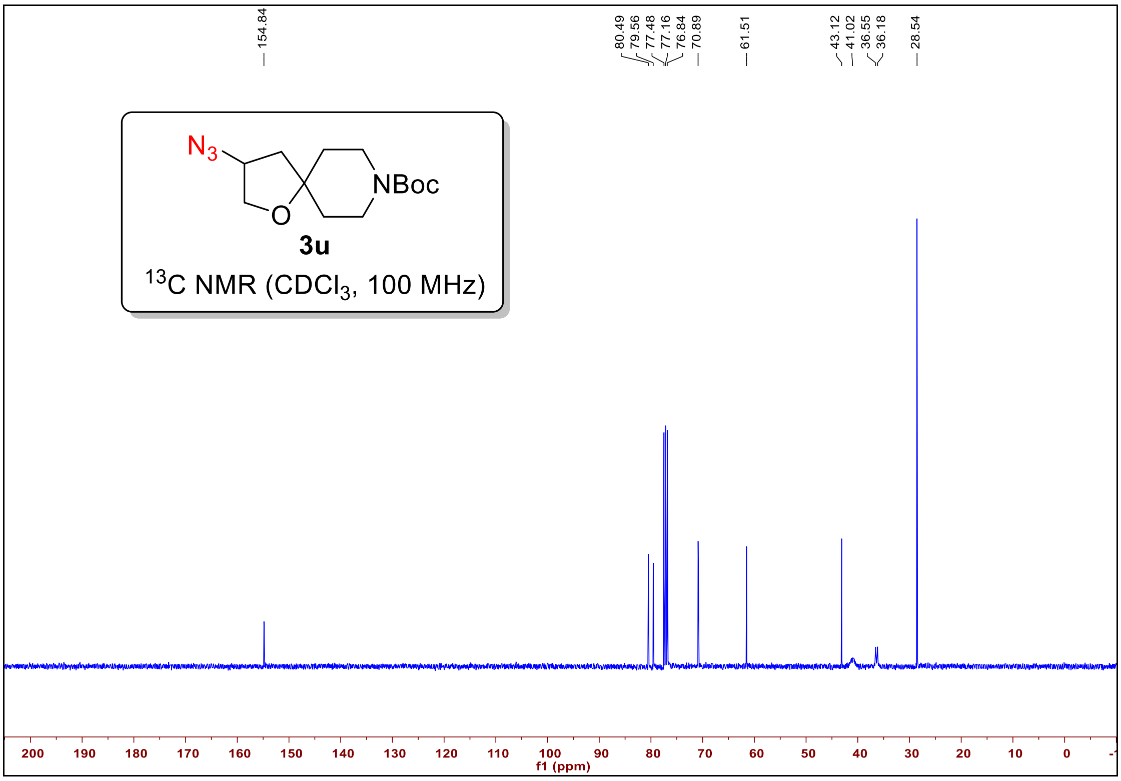

Supplementary Figure 58. ^{13}C NMR Spectrum of 3u

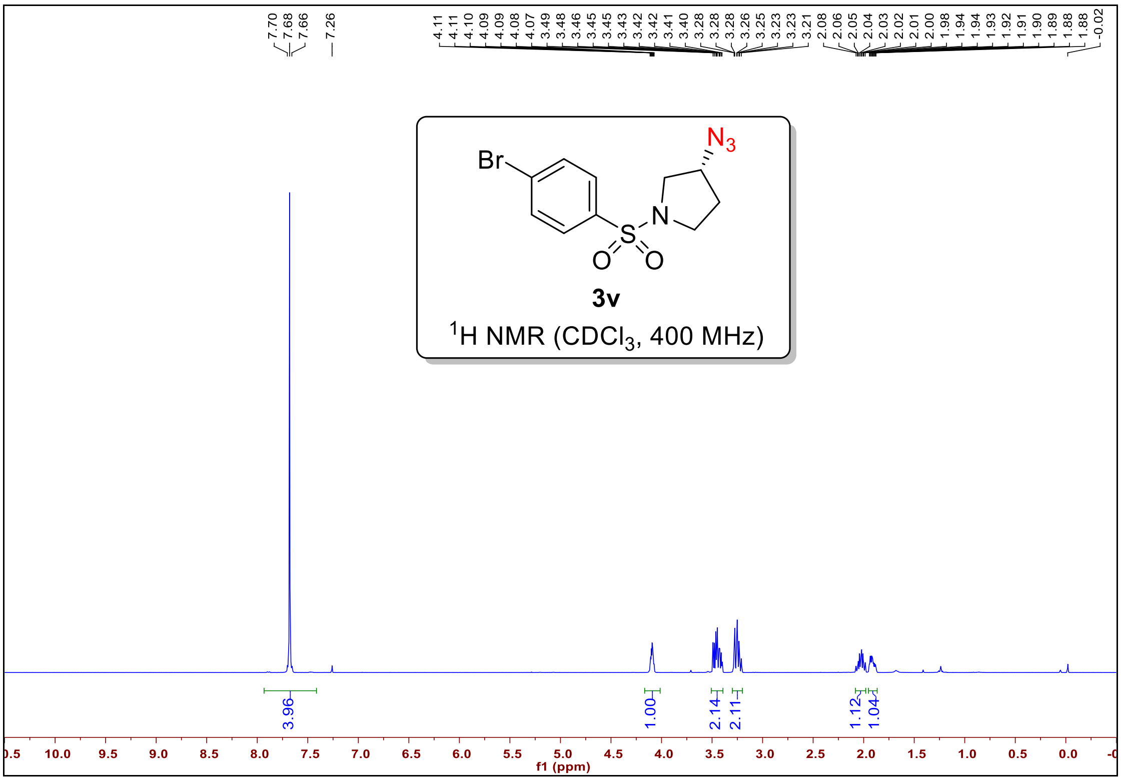

Supplementary Figure 59. ^{1}H NMR Spectrum of 3v

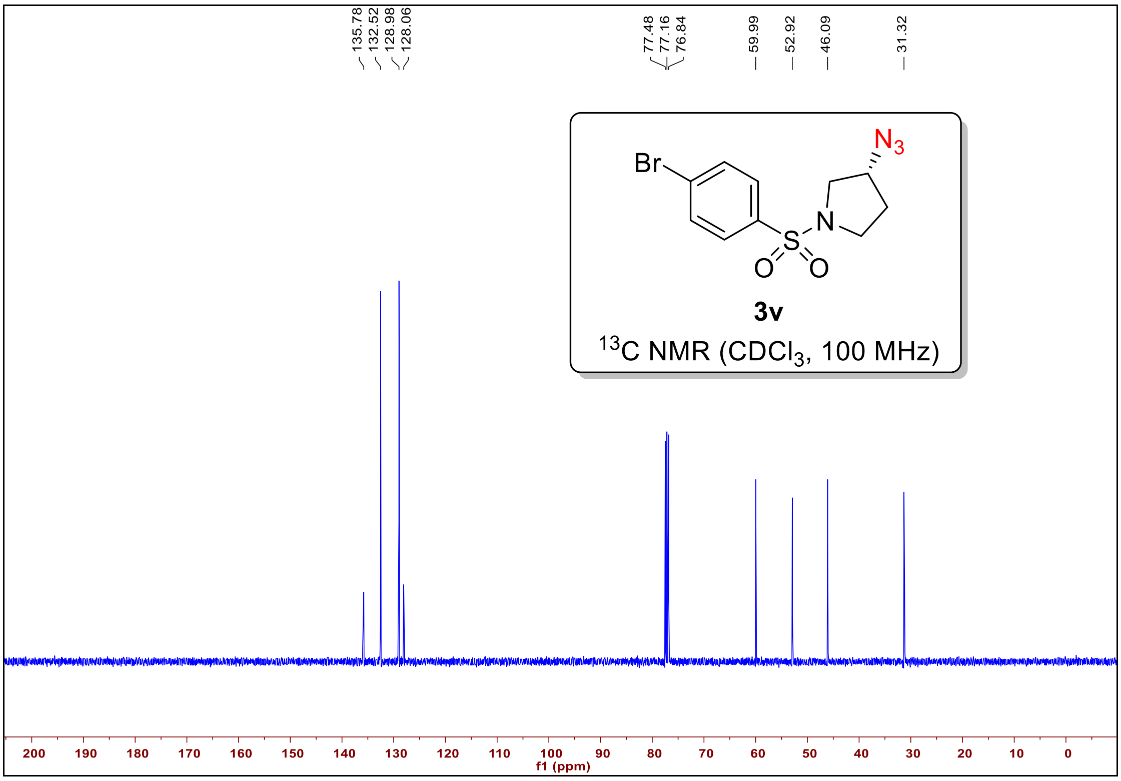

Supplementary Figure 60. ^{13}C NMR Spectrum of 3v

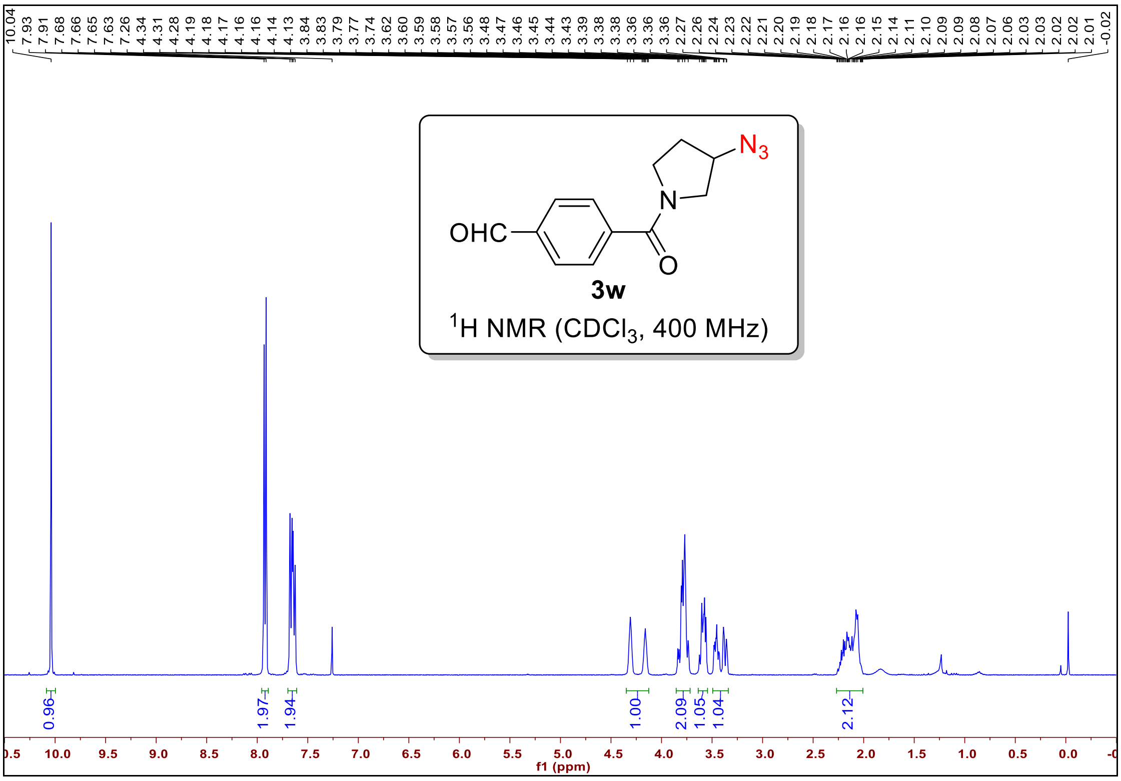

Supplementary Figure 61. ^{1}**H NMR Spectrum of 3w**

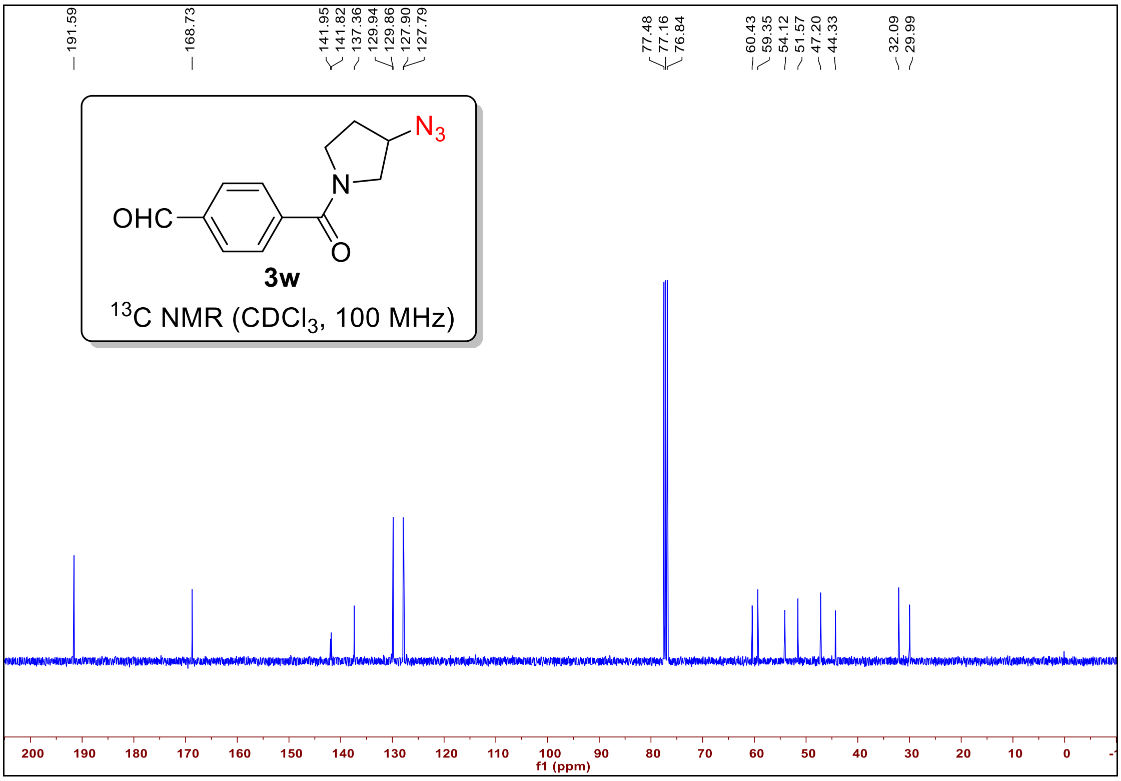

Supplementary Figure 62. ^{13}**C NMR Spectrum of 3w**

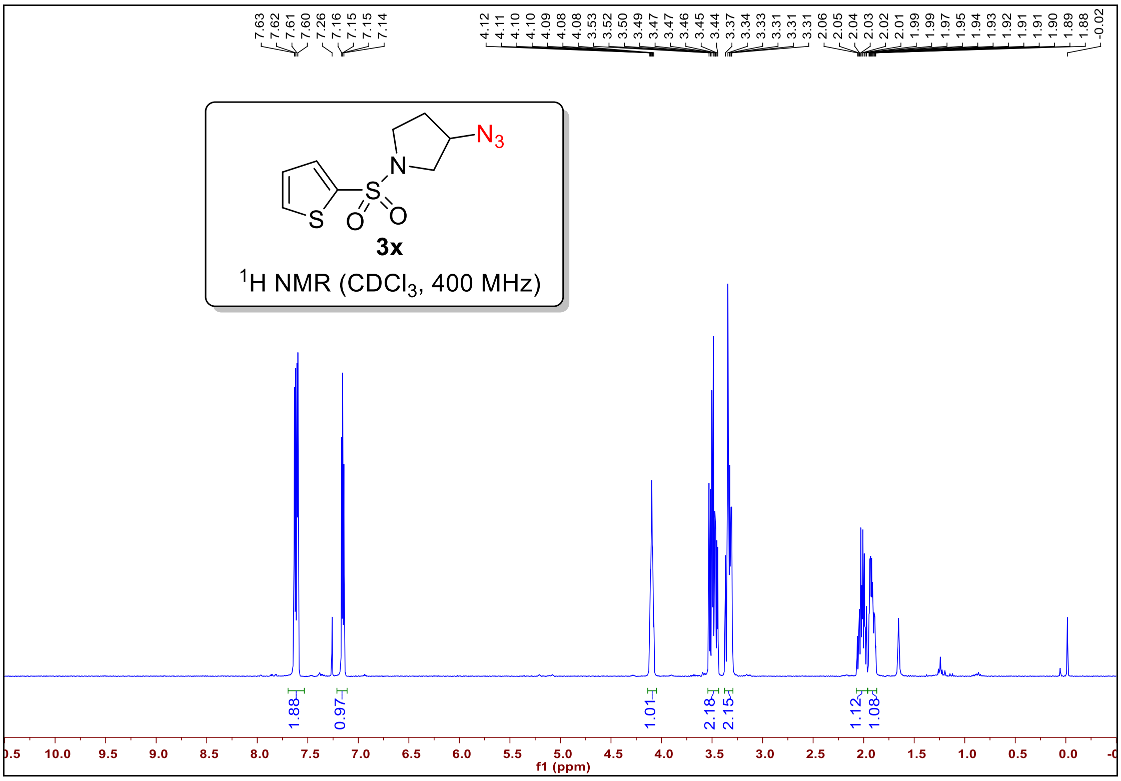

Supplementary Figure 63. ^{1}H NMR Spectrum of 3x

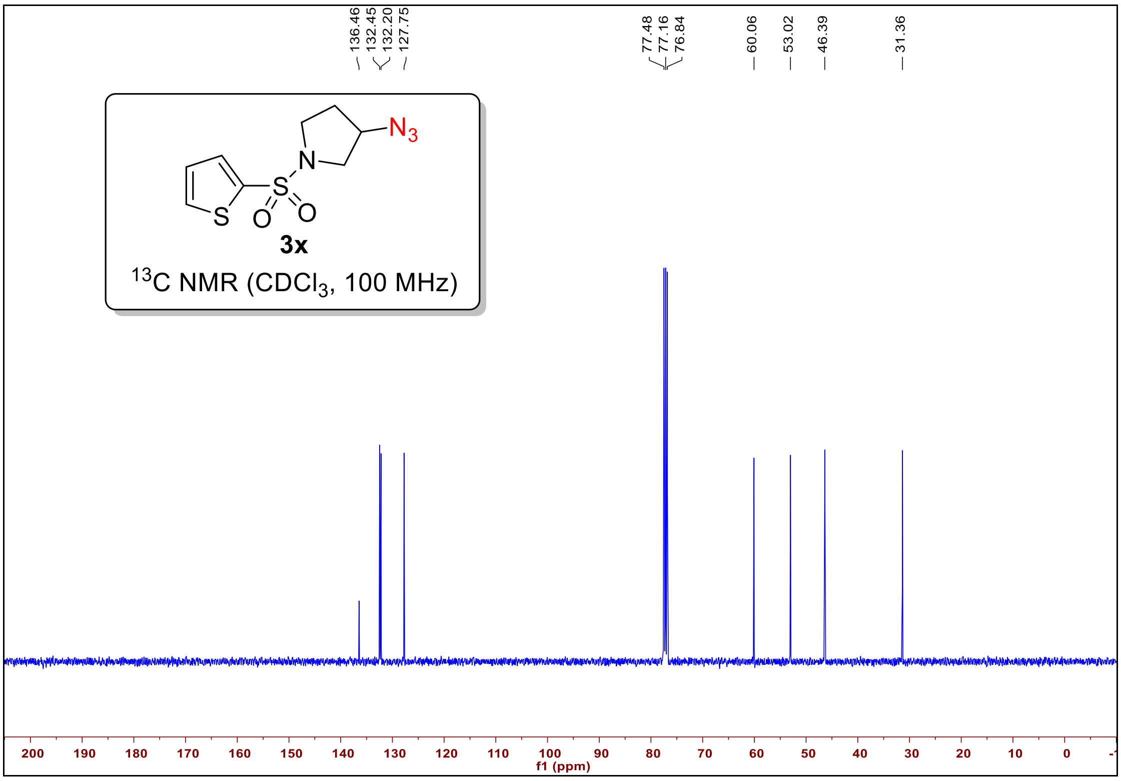

Supplementary Figure 64. ^{13}C NMR Spectrum of 3x

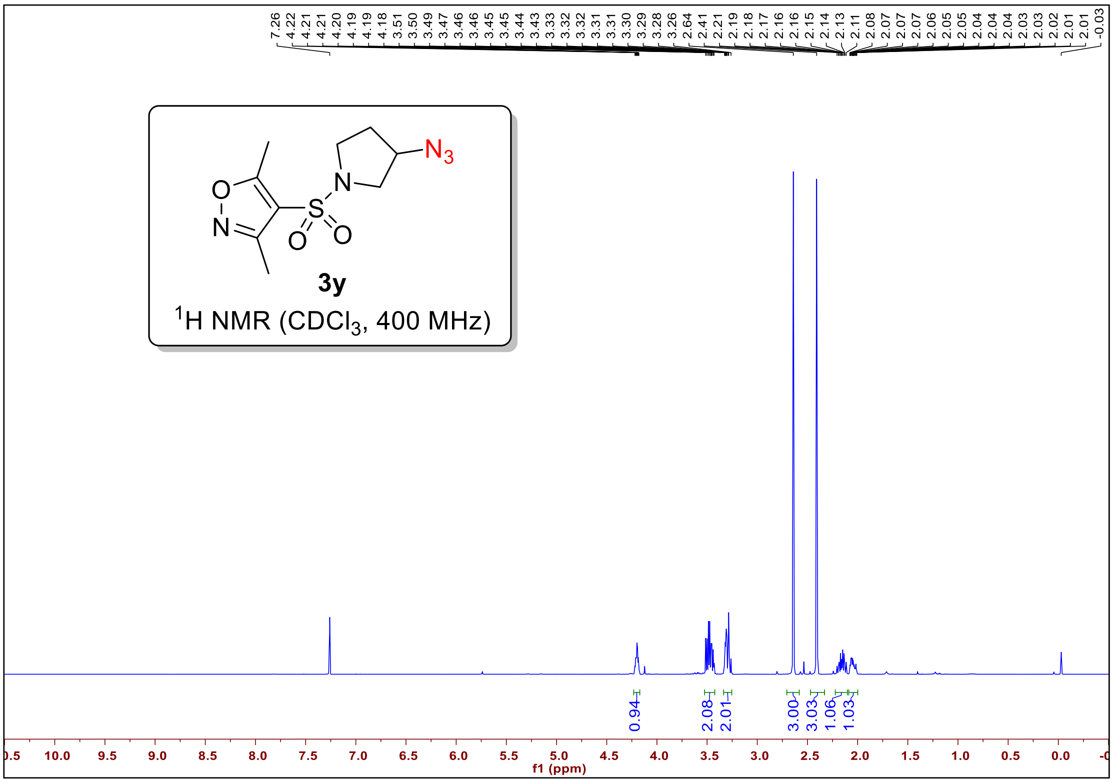

Supplementary Figure 65. ^{1}H NMR Spectrum of 3y

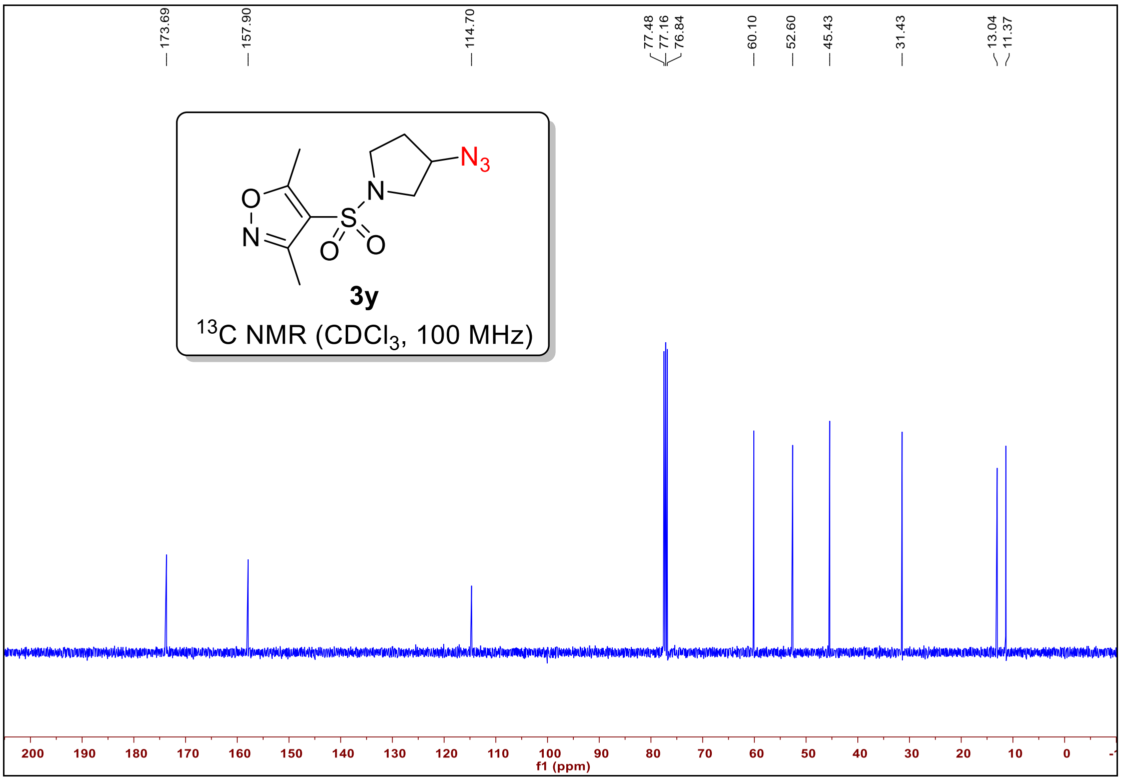

Supplementary Figure 66. ^{13}C NMR Spectrum of 3y

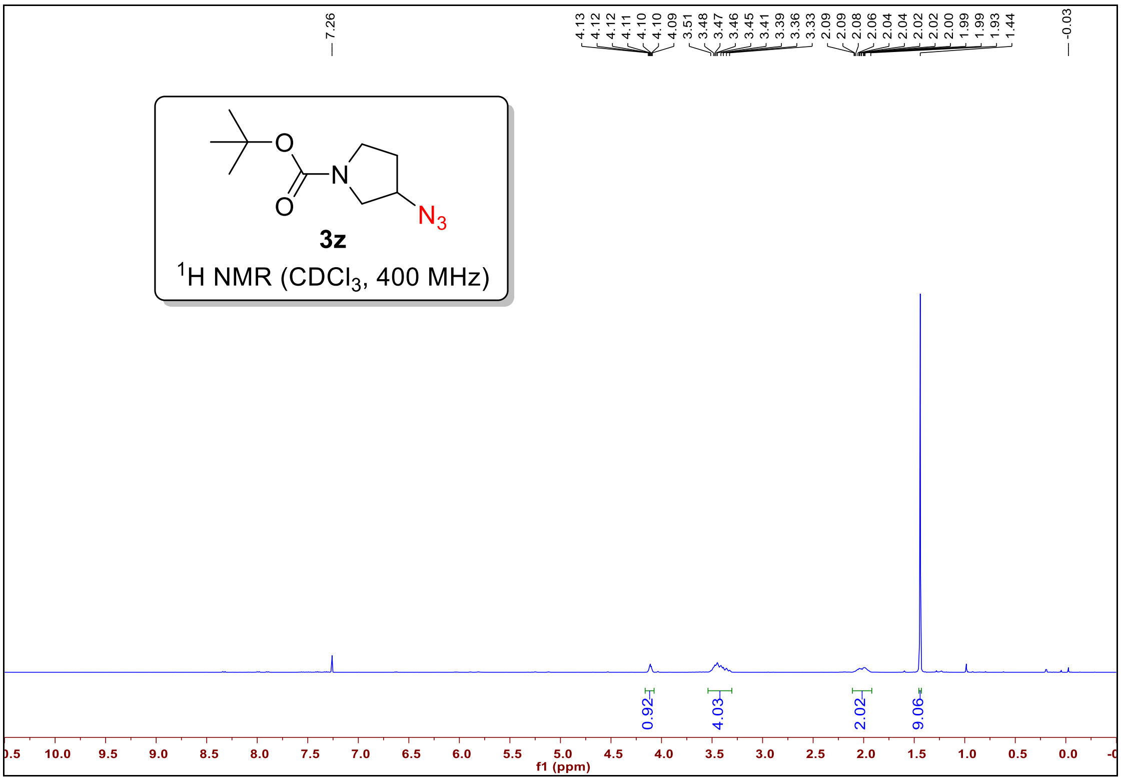

Supplementary Figure 67. ^{1}H NMR Spectrum of 3z

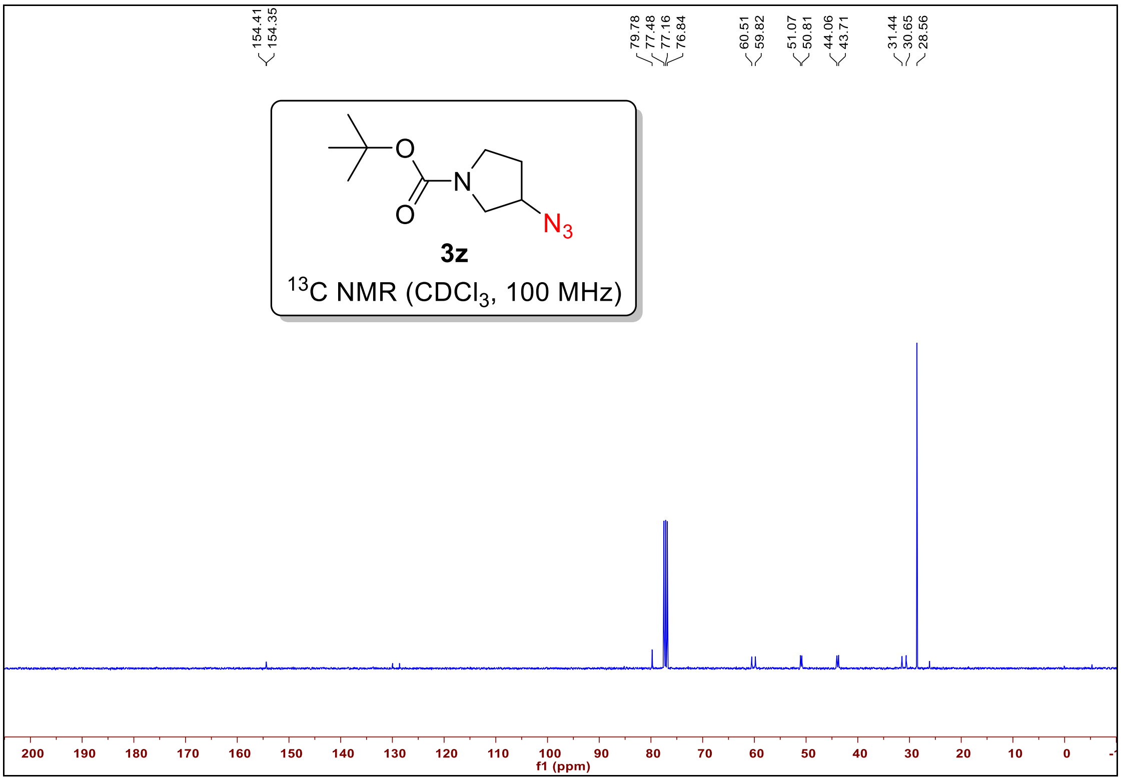

Supplementary Figure 68. ^{13}C NMR Spectrum of 3z

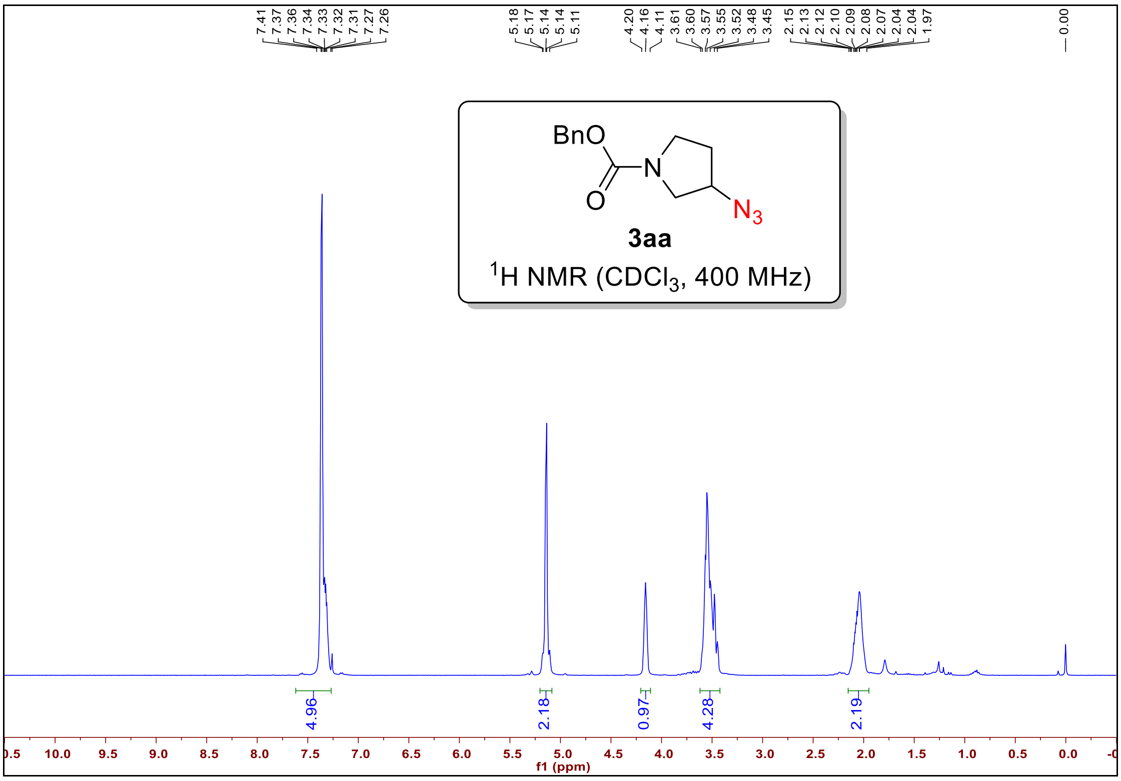

Supplementary Figure 69. ^{1}H NMR Spectrum of 3aa

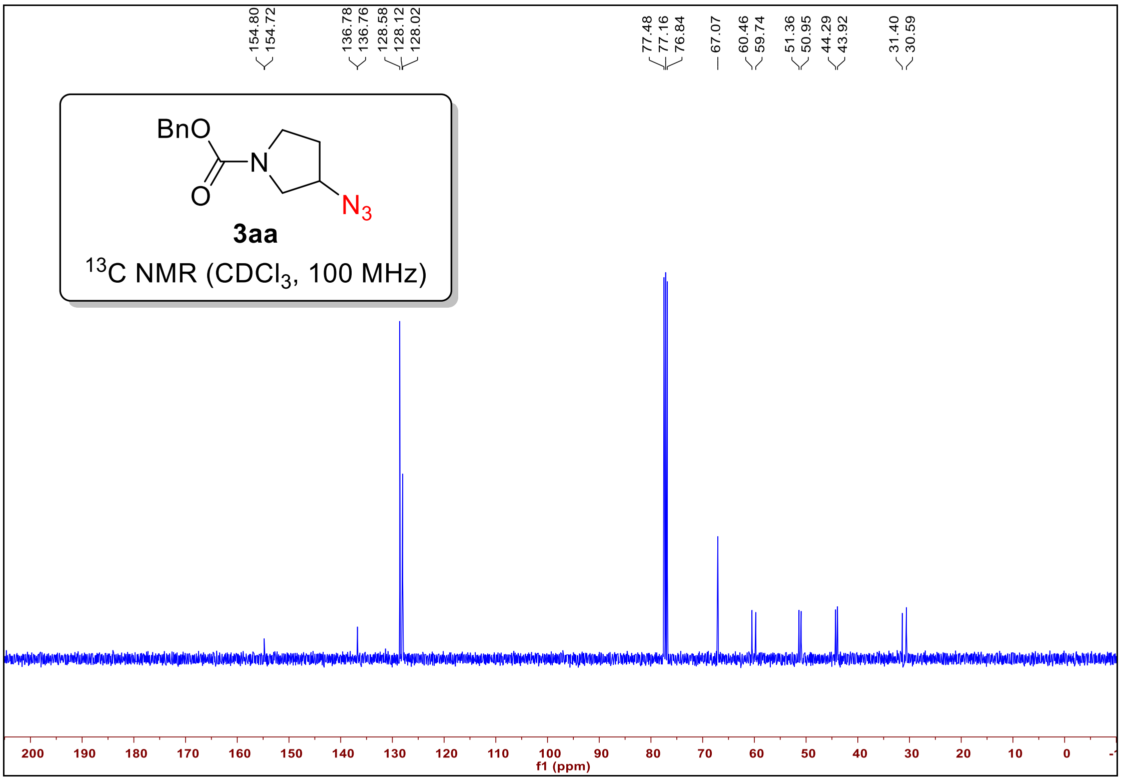

Supplementary Figure 70. ^{13}C NMR Spectrum of 3aa

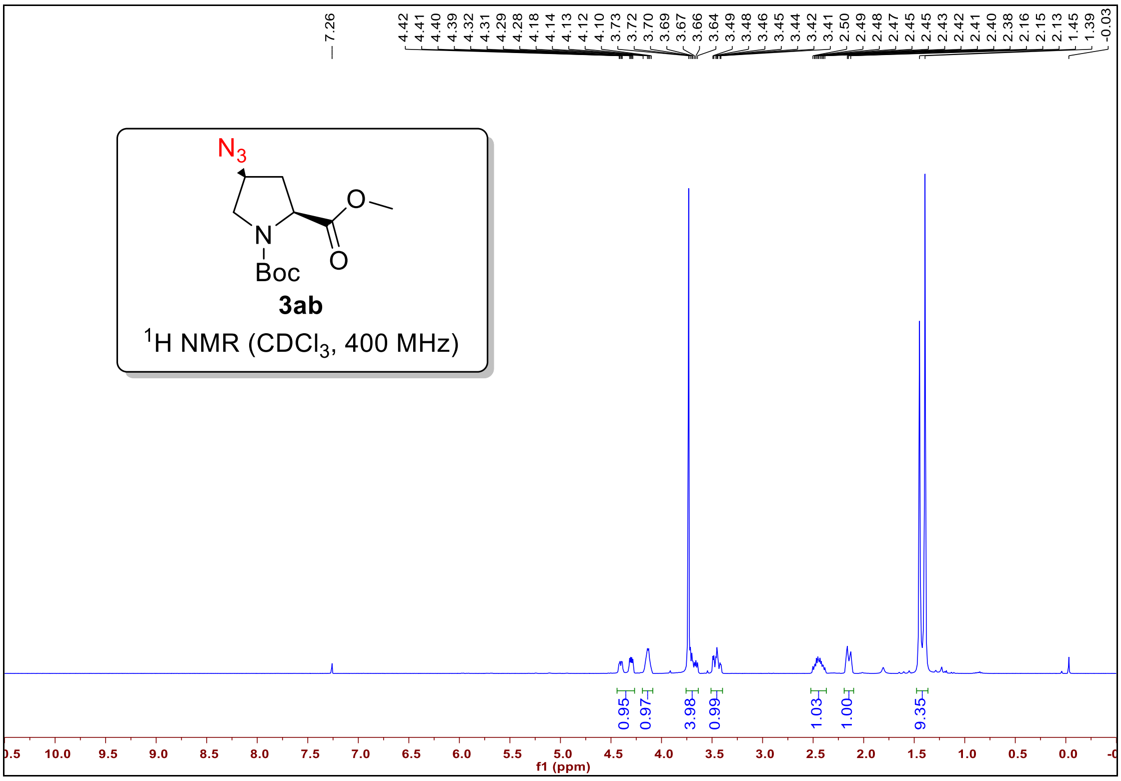

Supplementary Figure 71. ^{1}**H NMR Spectrum of 3ab**

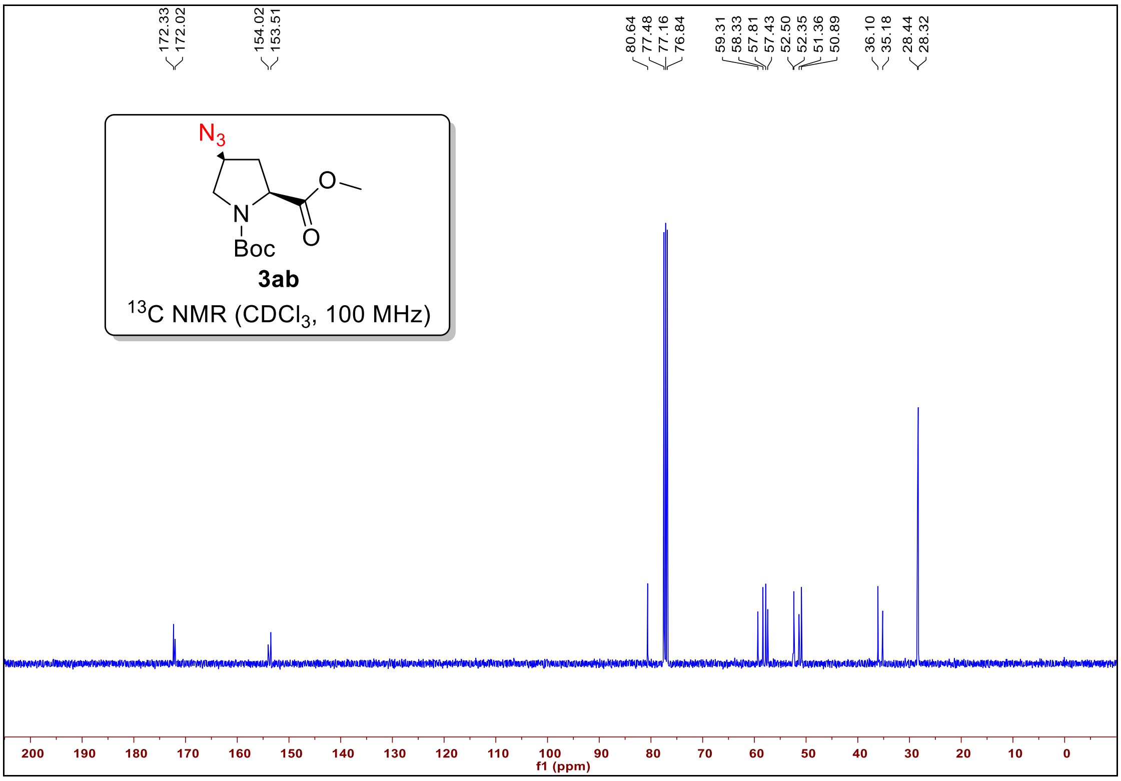

Supplementary Figure 72. ^{13}**C NMR Spectrum of 3ab**

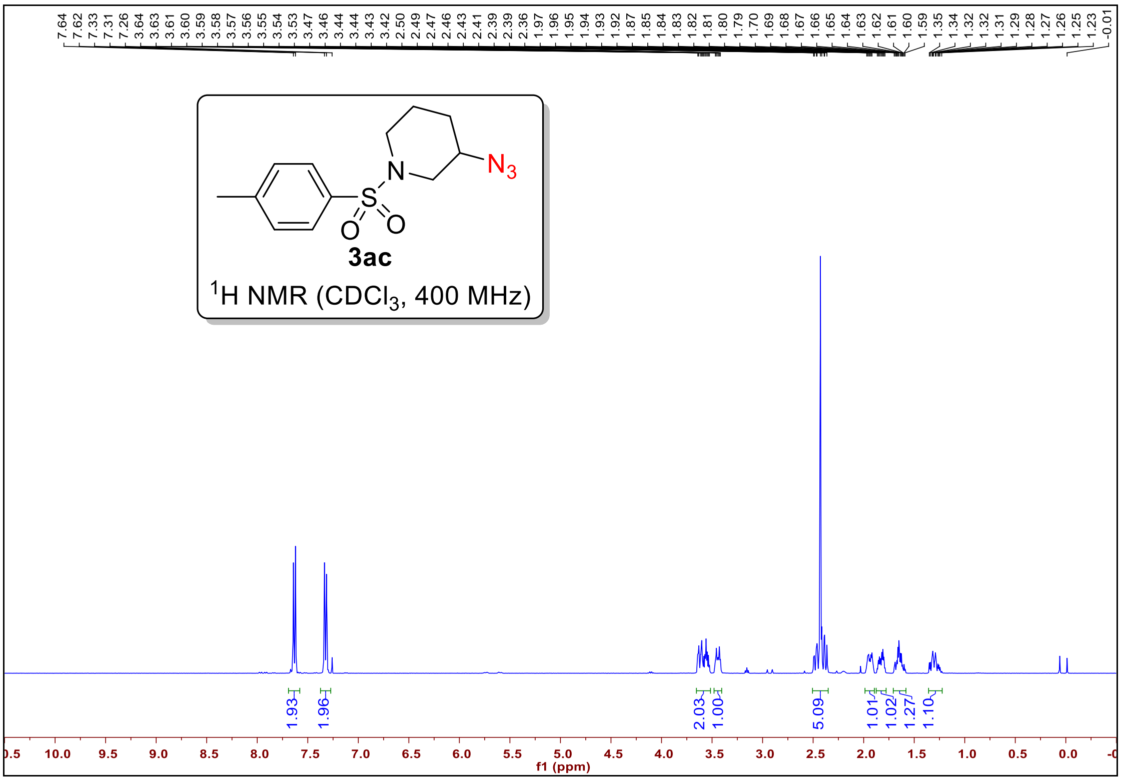

Supplementary Figure 73. ^{1}H NMR Spectrum of 3ac

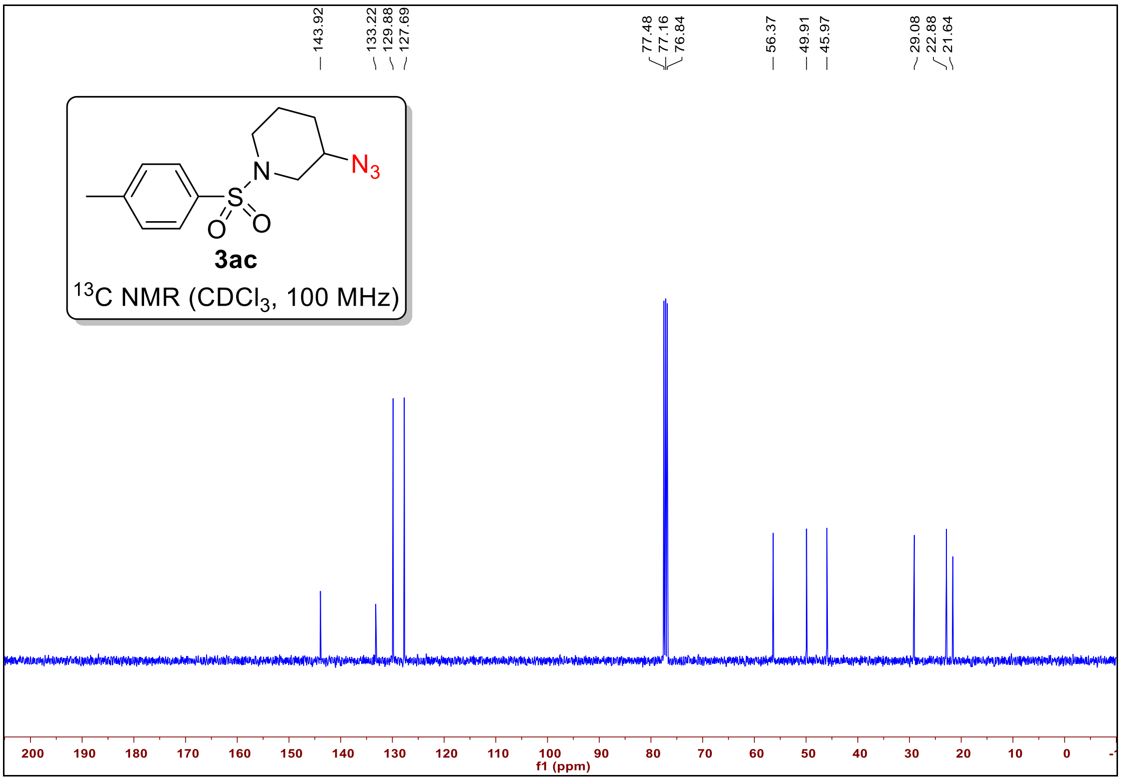

Supplementary Figure 74. ^{13}C NMR Spectrum of 3ac

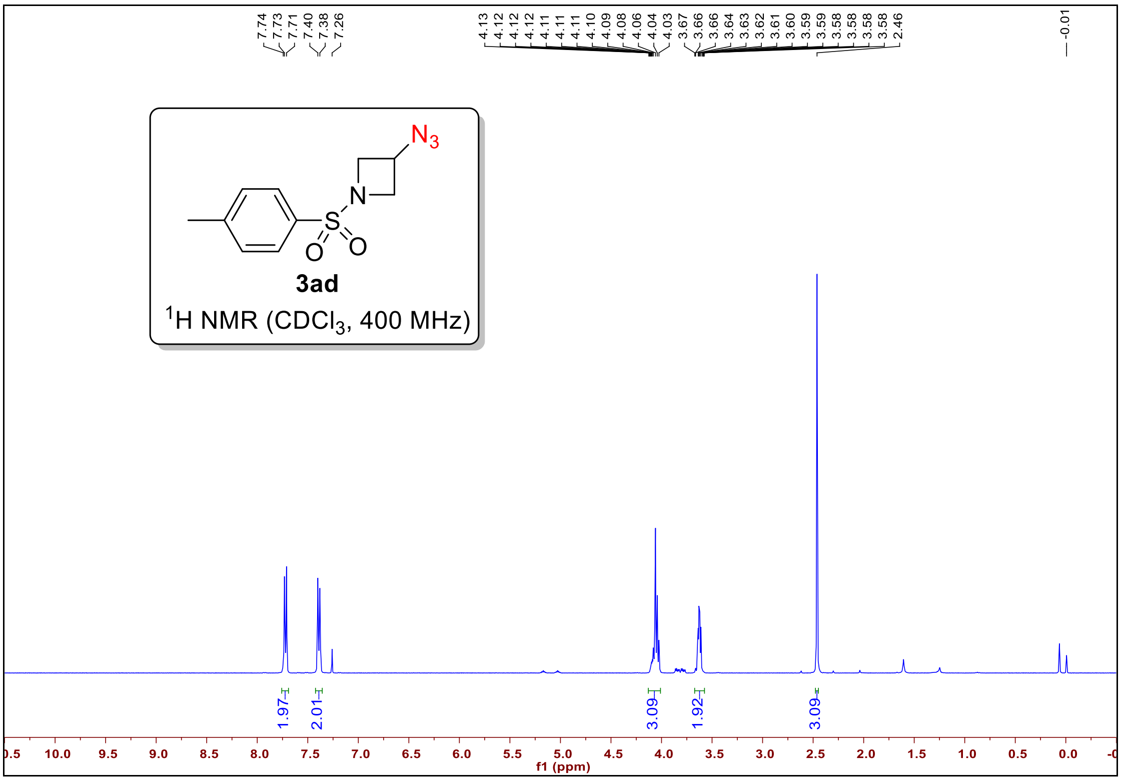

Supplementary Figure 75. ^{1}H NMR Spectrum of 3ad

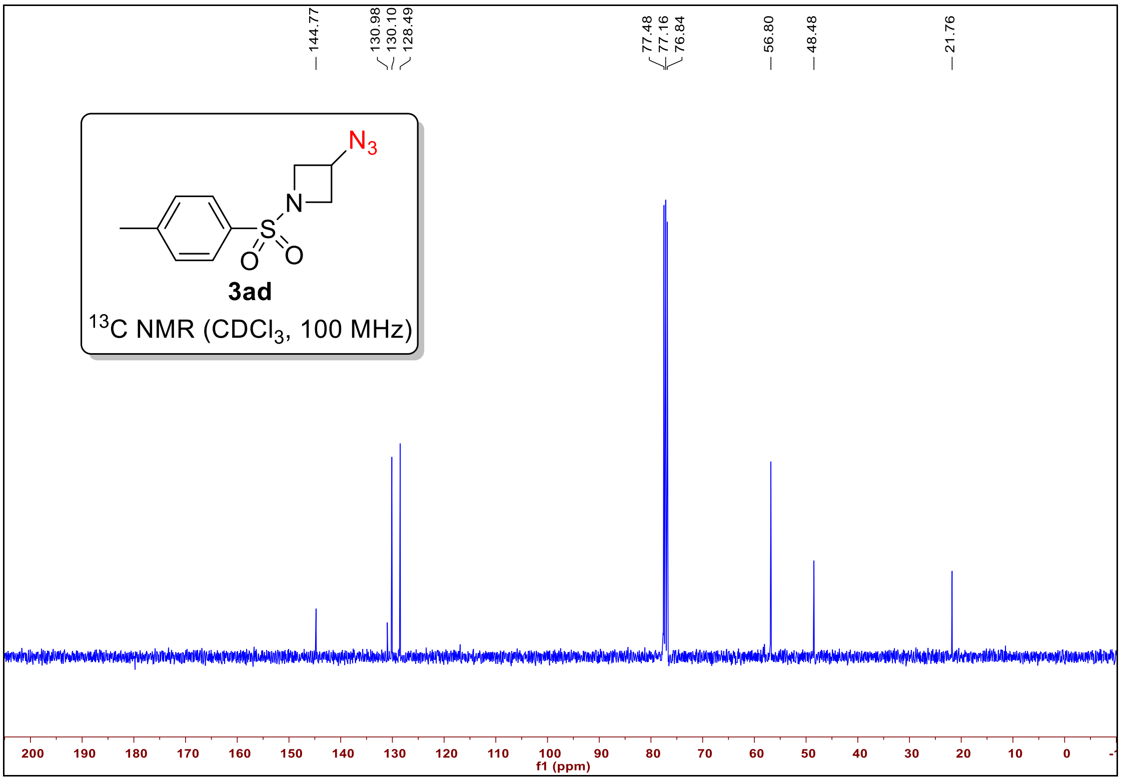

Supplementary Figure 76. ^{13}C NMR Spectrum of 3ad

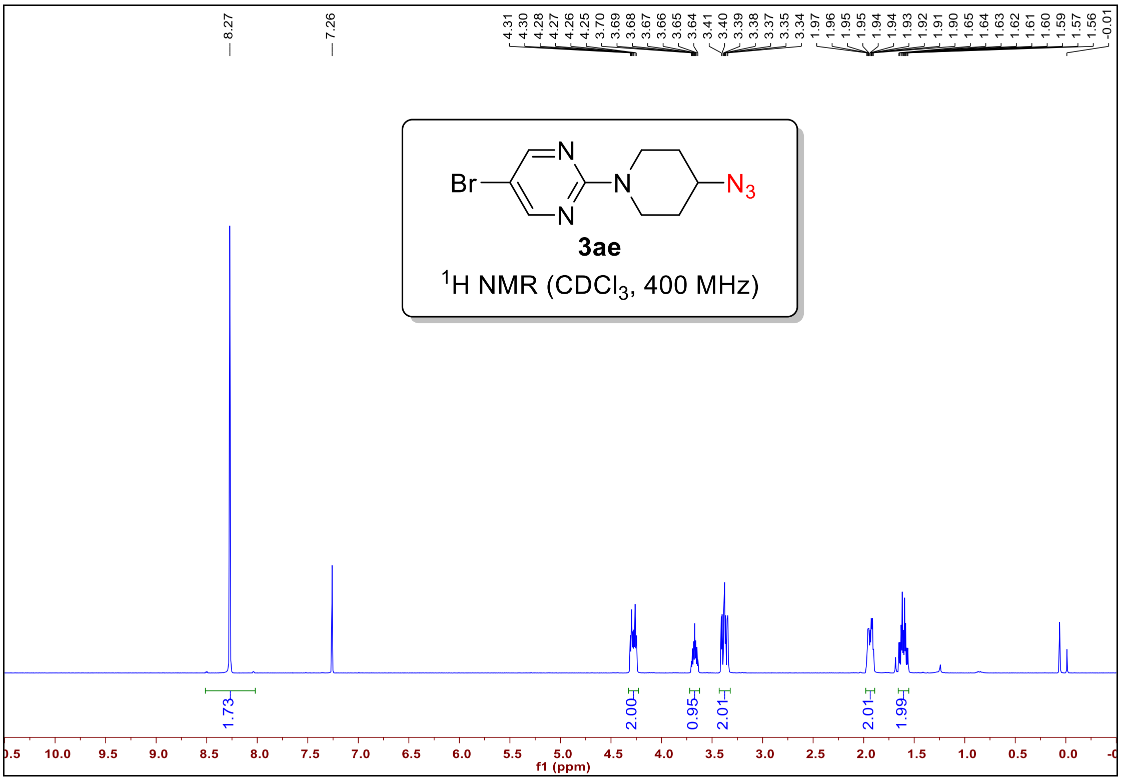

Supplementary Figure 77. ^{1}H NMR Spectrum of 3ae

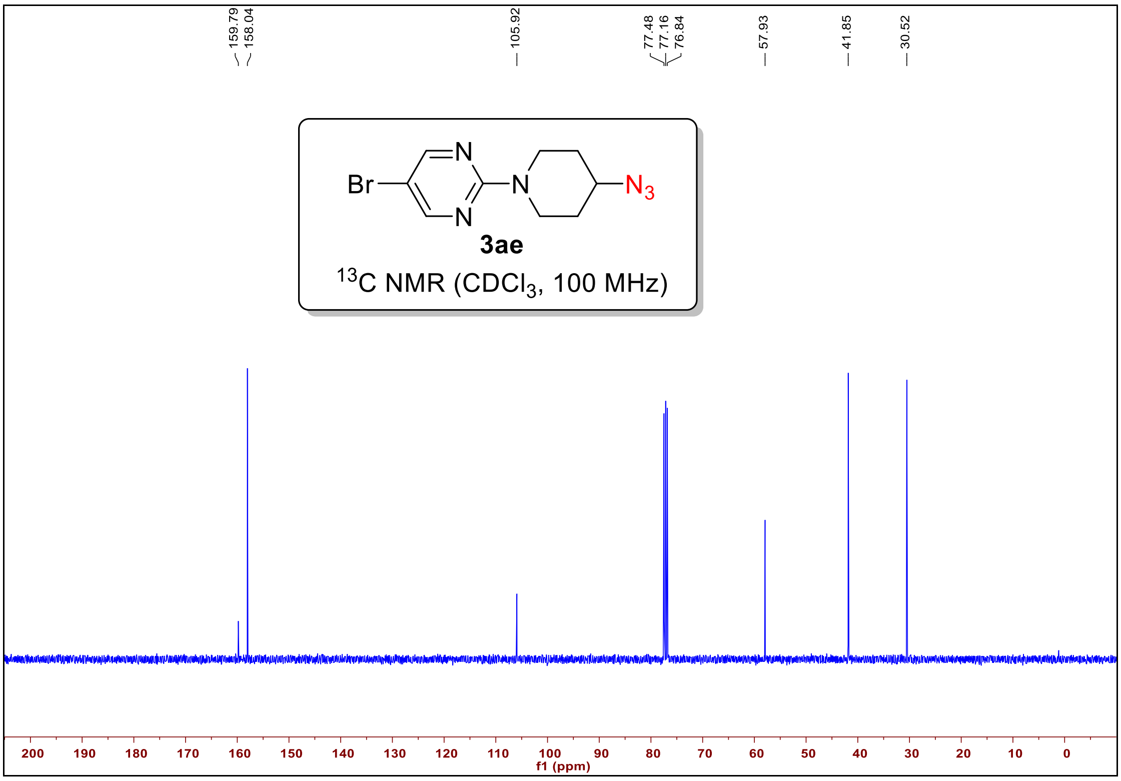

Supplementary Figure 78. ^{13}C NMR Spectrum of 3ae

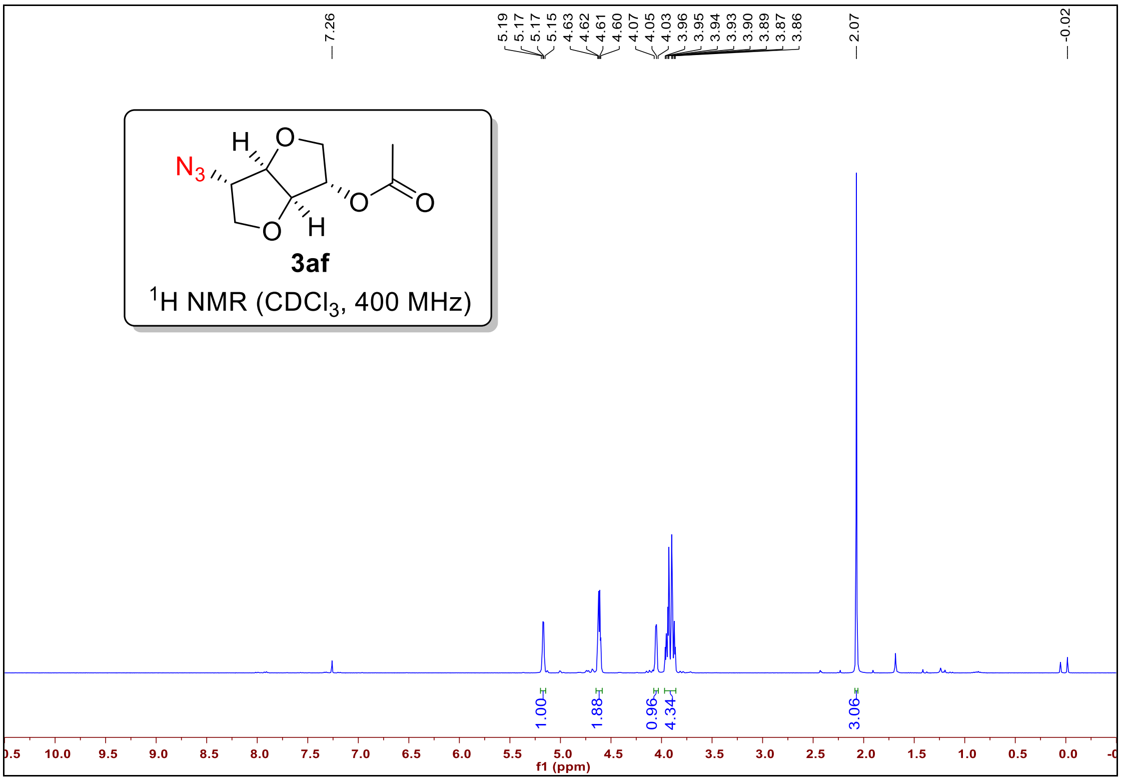

Supplementary Figure 79. ^{1}H NMR Spectrum of 3af

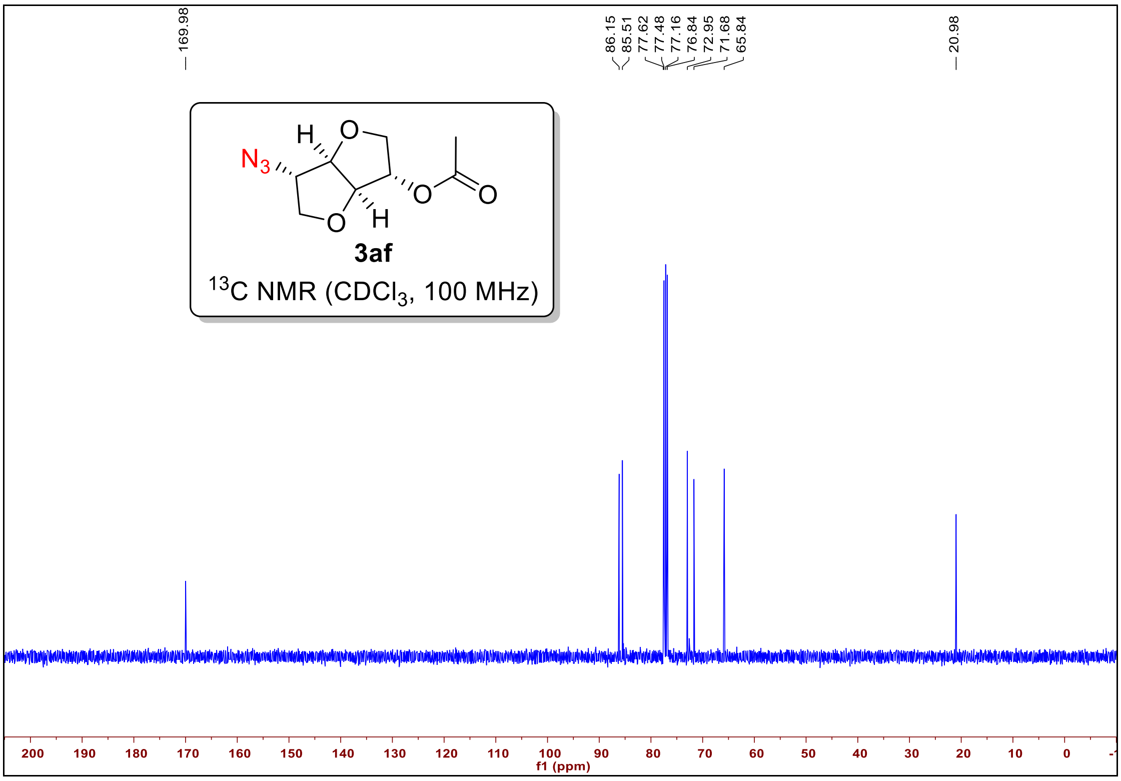

Supplementary Figure 80. ^{13}C NMR Spectrum of 3af

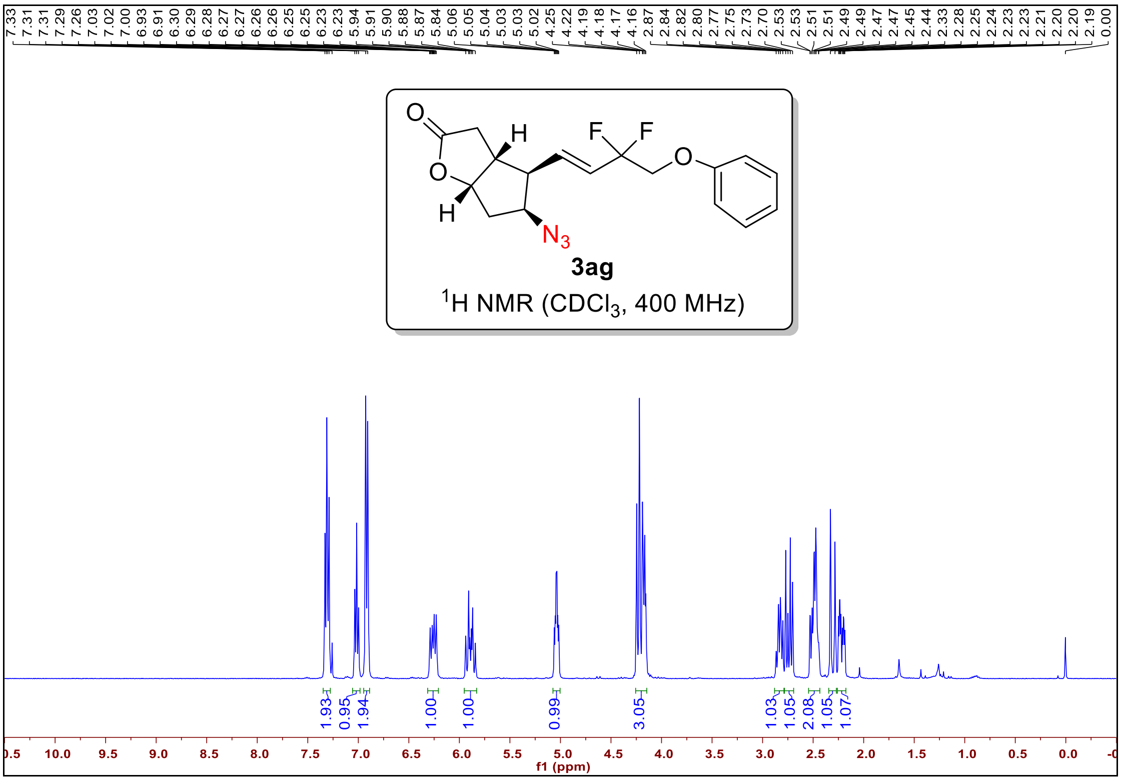

Supplementary Figure 81. ^{1}H NMR Spectrum of 3ag

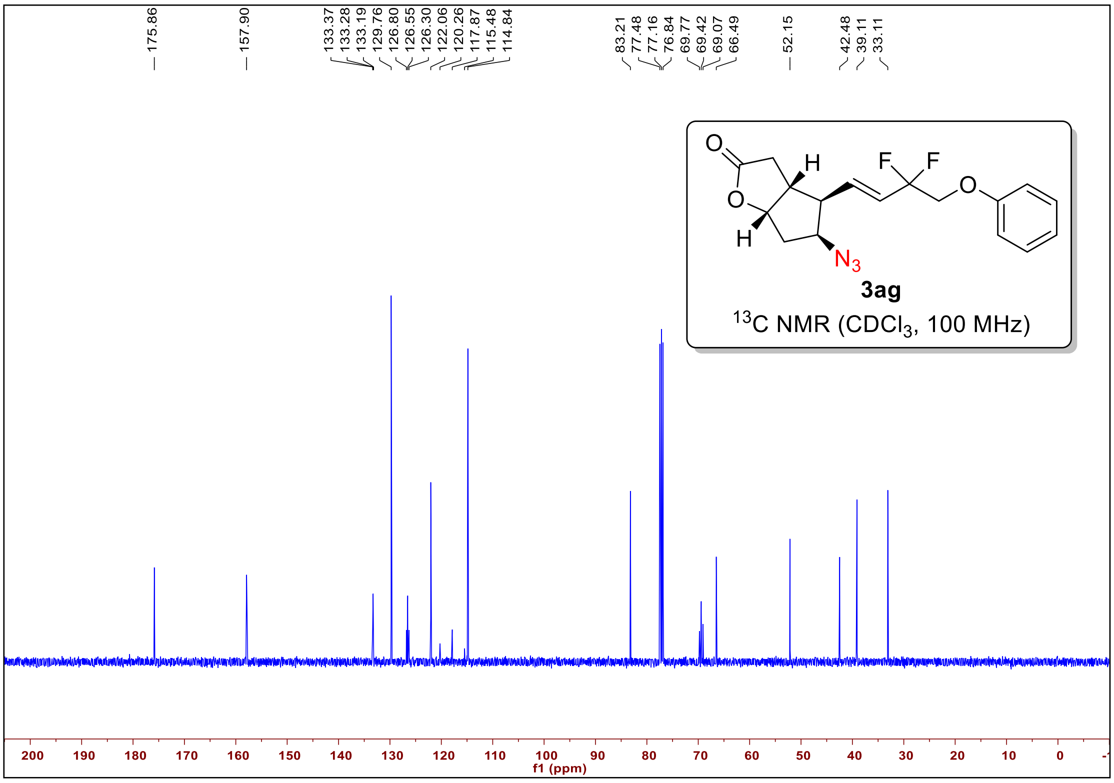

Supplementary Figure 82. ^{13}C NMR Spectrum of 3ag

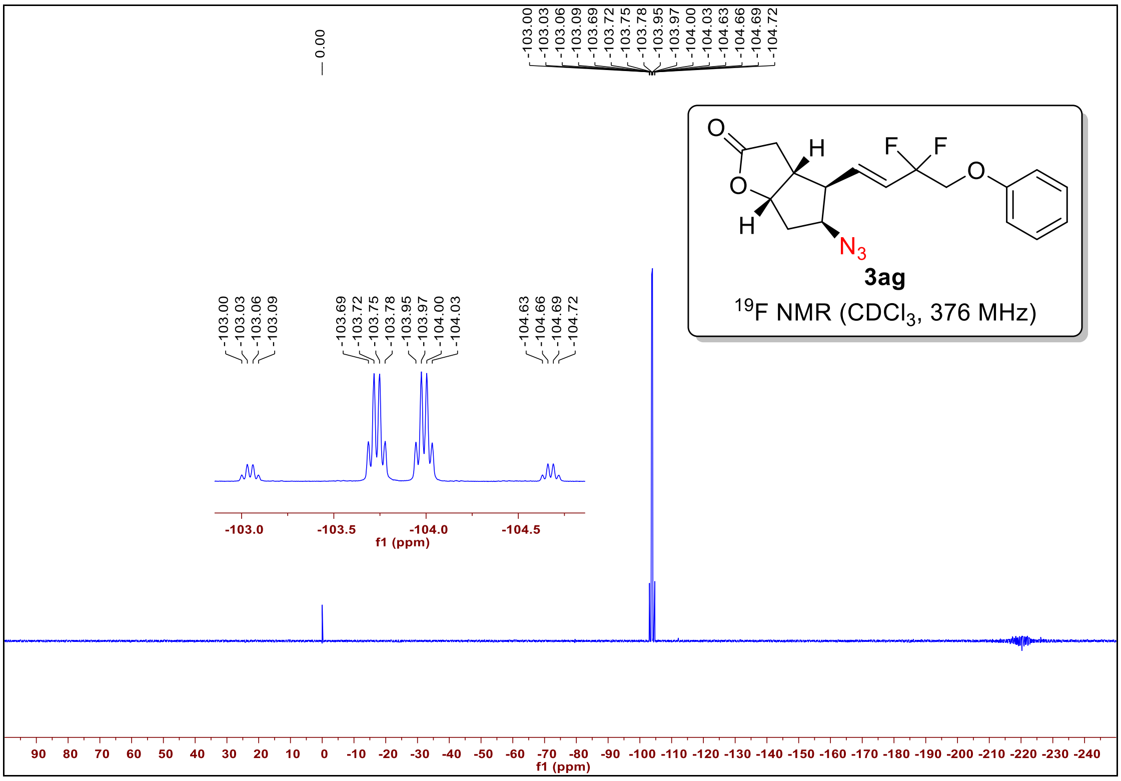

Supplementary Figure 83. ^{19}F NMR Spectrum of 3ag

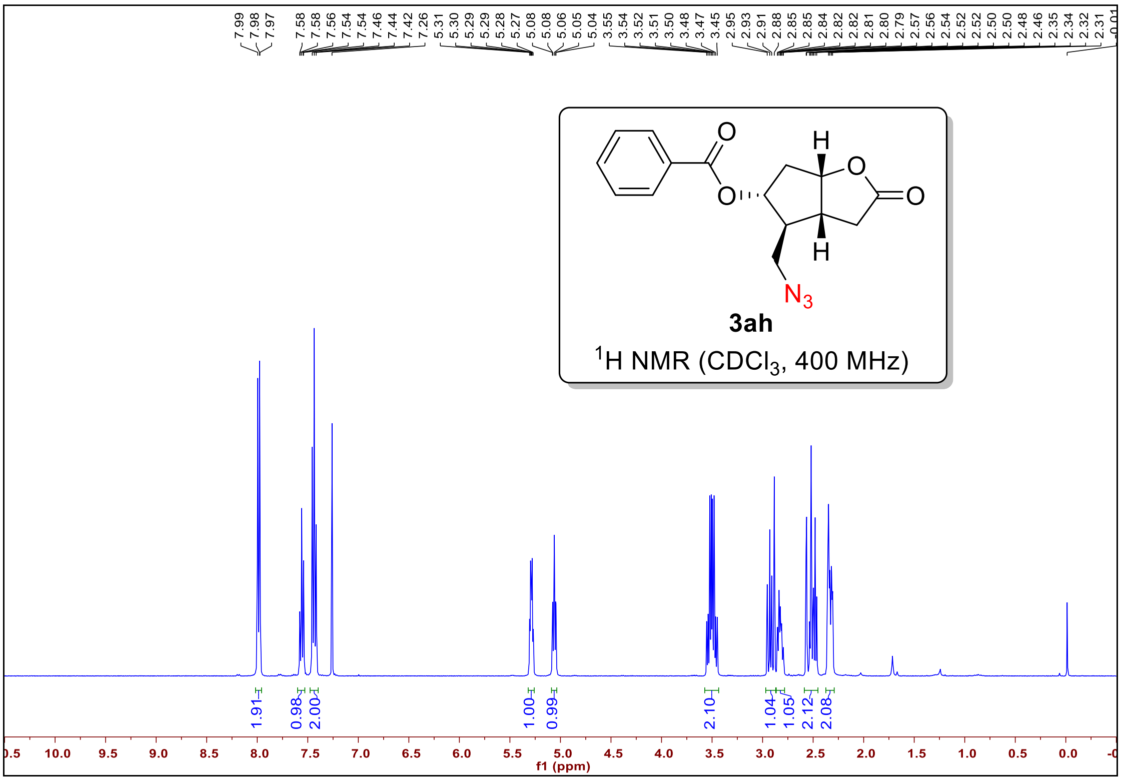

Supplementary Figure 84. ^{1}H NMR Spectrum of 3ah

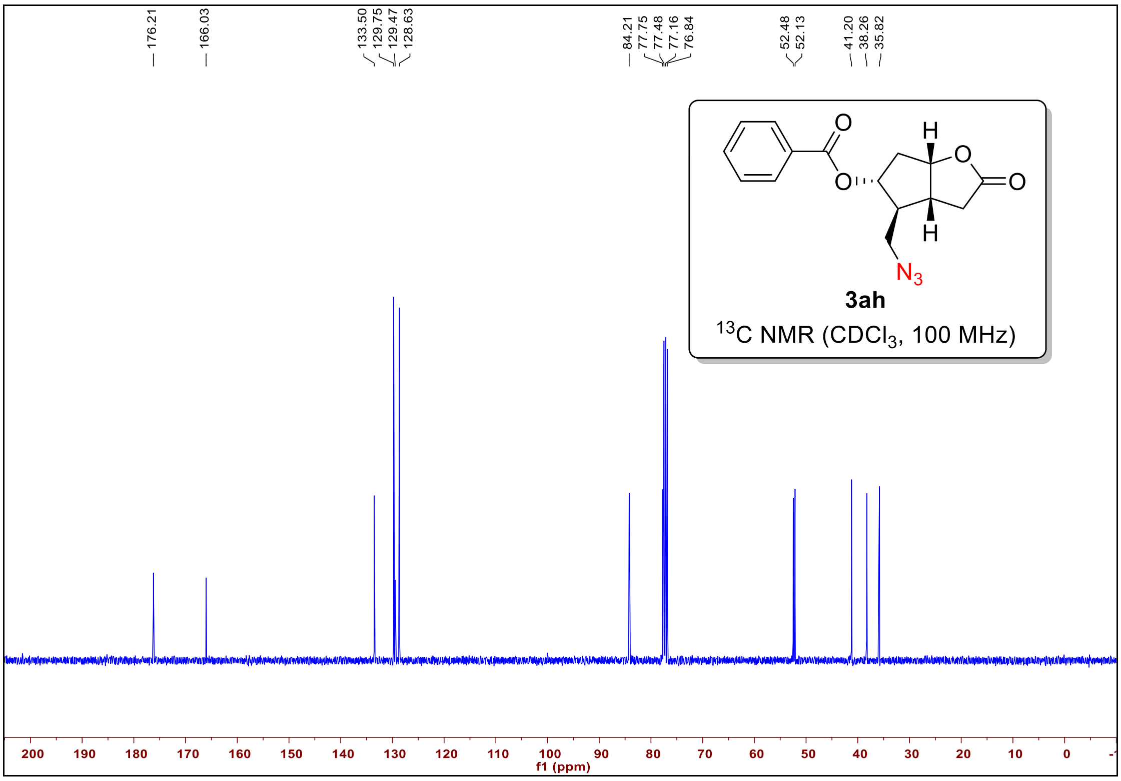

Supplementary Figure 85. ^{13}C NMR Spectrum of 3ah

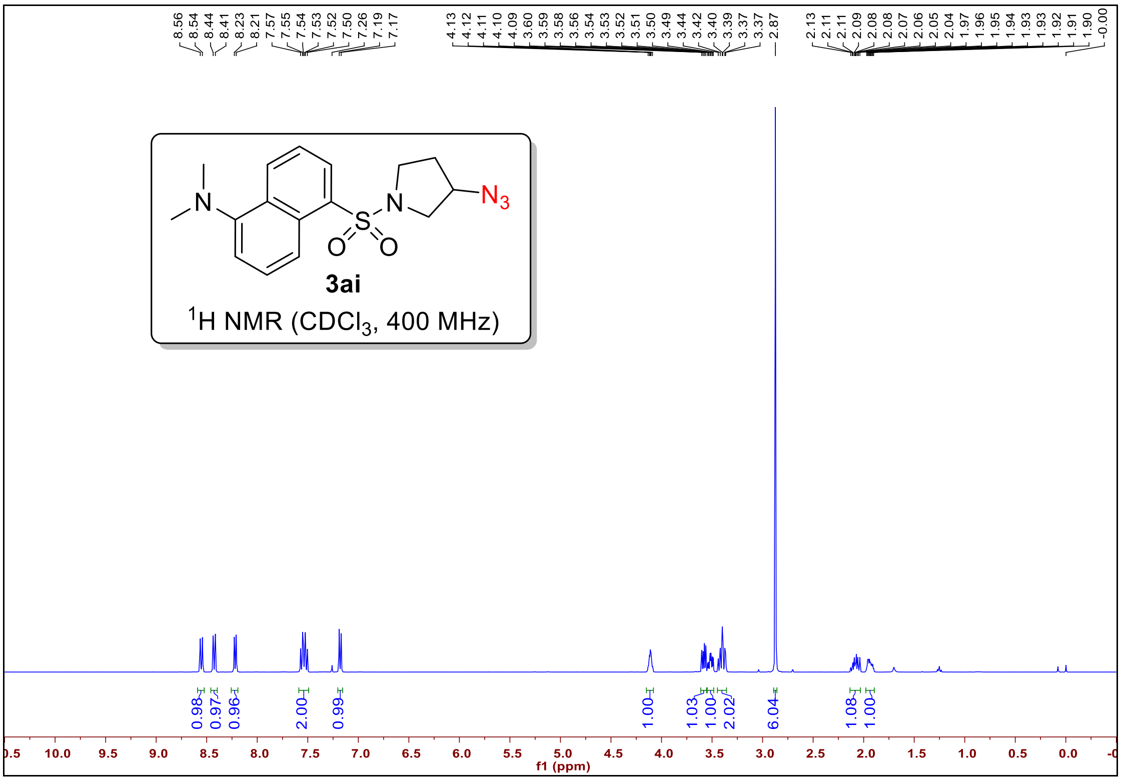

Supplementary Figure 86. ^{1}H NMR Spectrum of 3ai

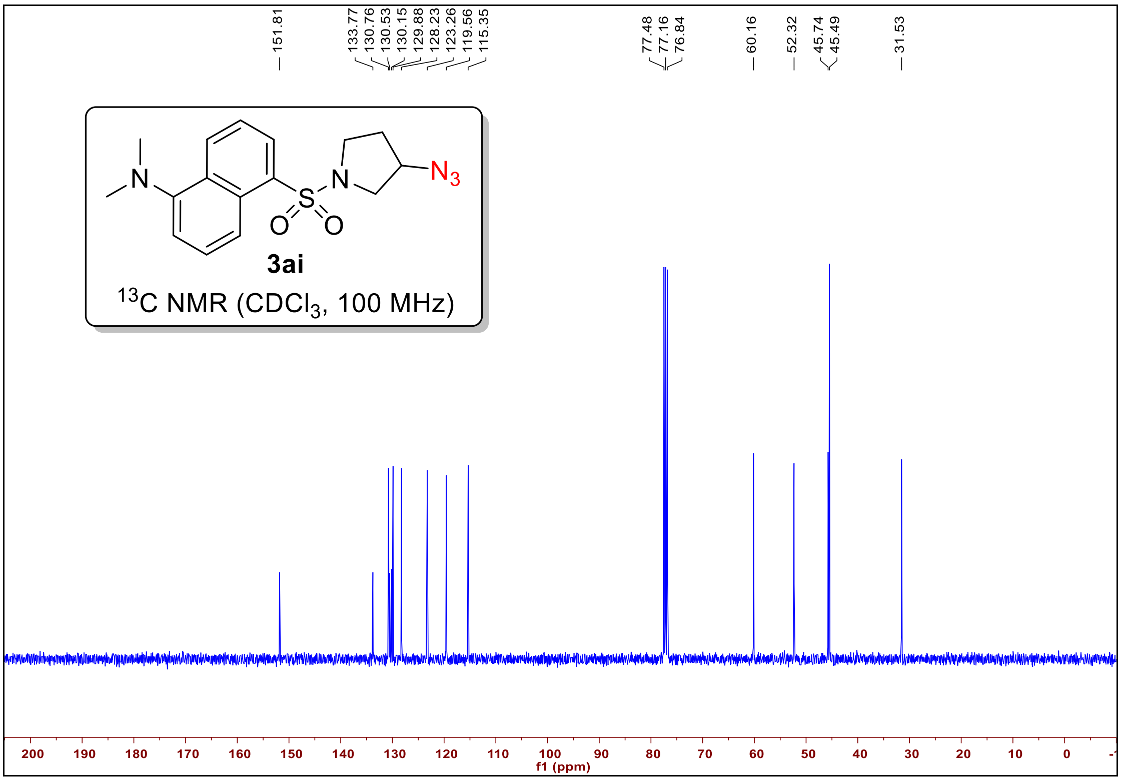

Supplementary Figure 87. ^{13}**C NMR Spectrum of 3ai**

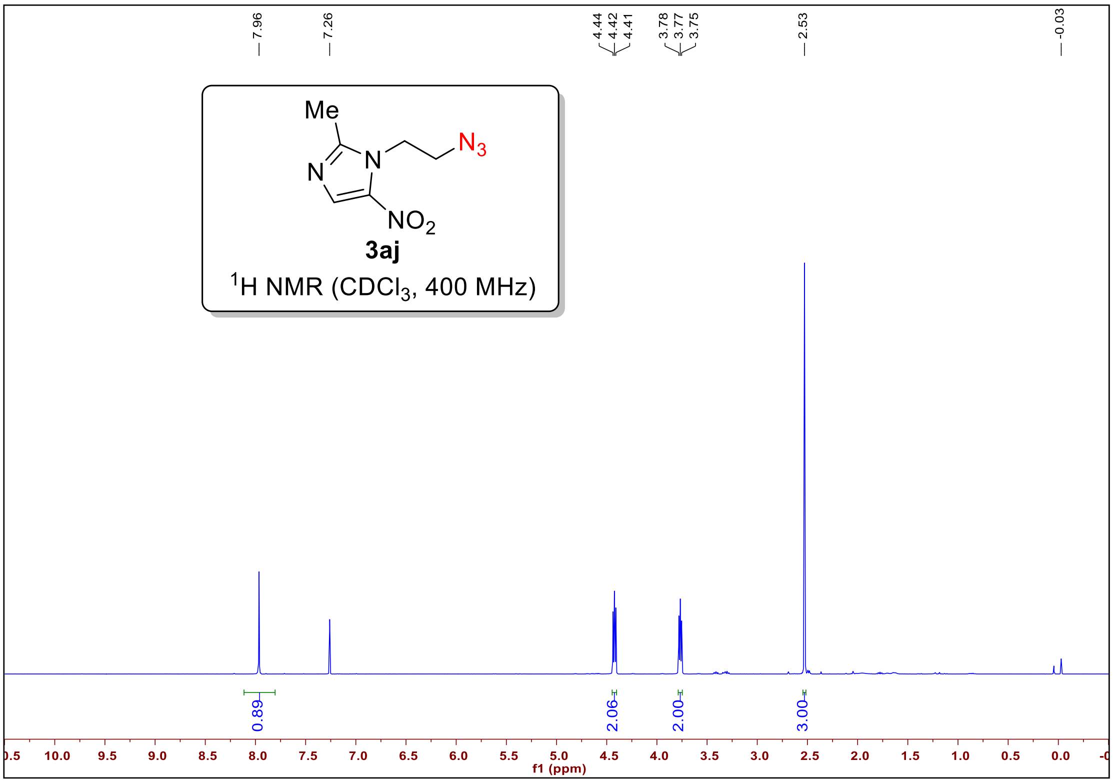

Supplementary Figure 88. ^{1}**H NMR Spectrum of 3aj**

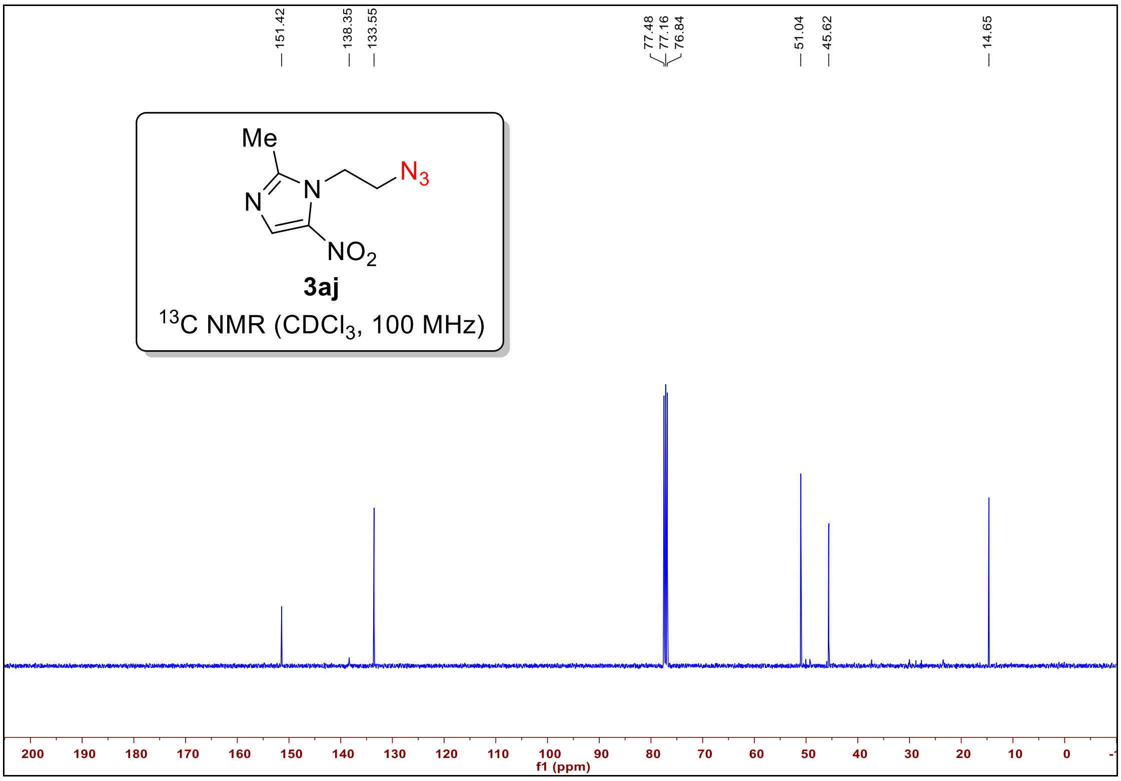

Supplementary Figure 89. ^{13}**C NMR Spectrum of 3aj**

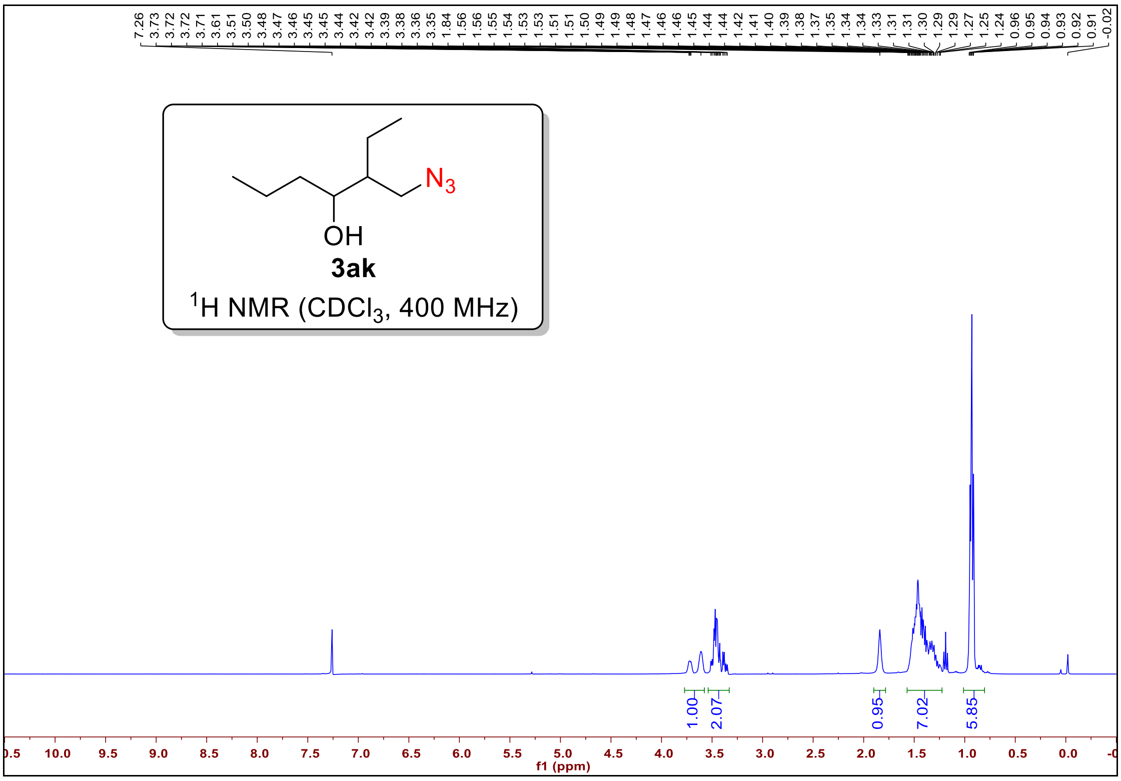

Supplementary Figure 90. ^{1}**H NMR Spectrum of 3ak**

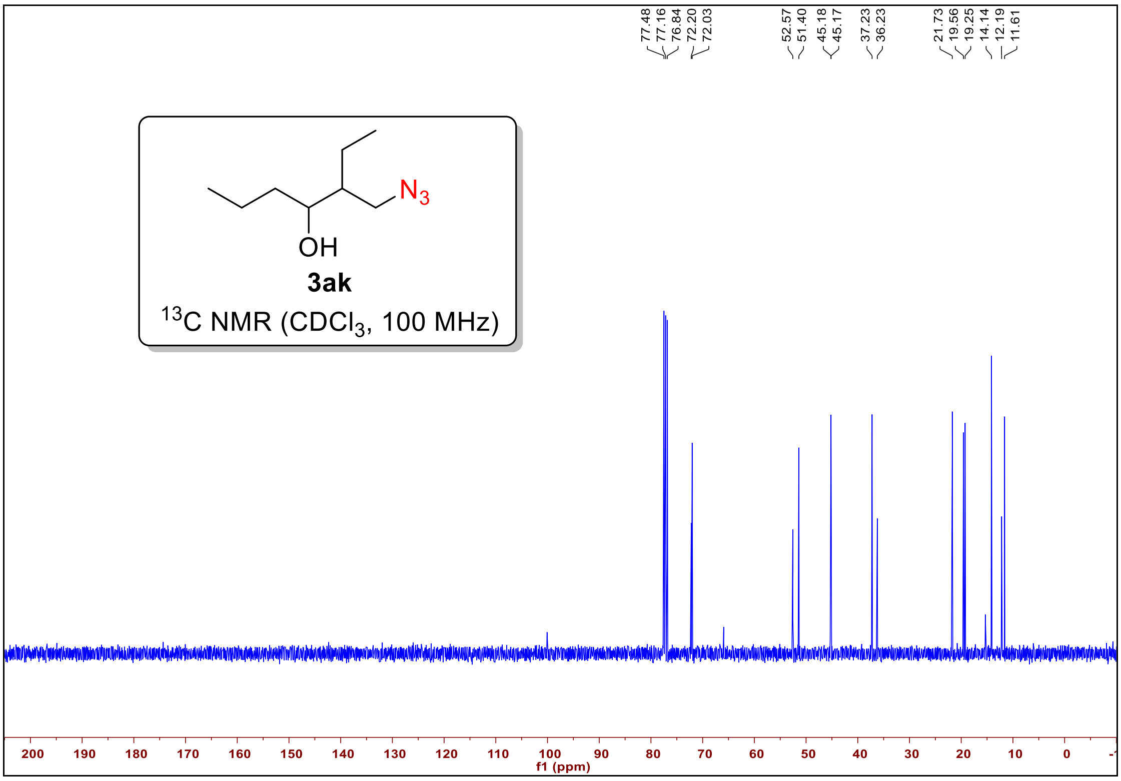

Supplementary Figure 91. ^{13}C **NMR Spectrum of 3ak**

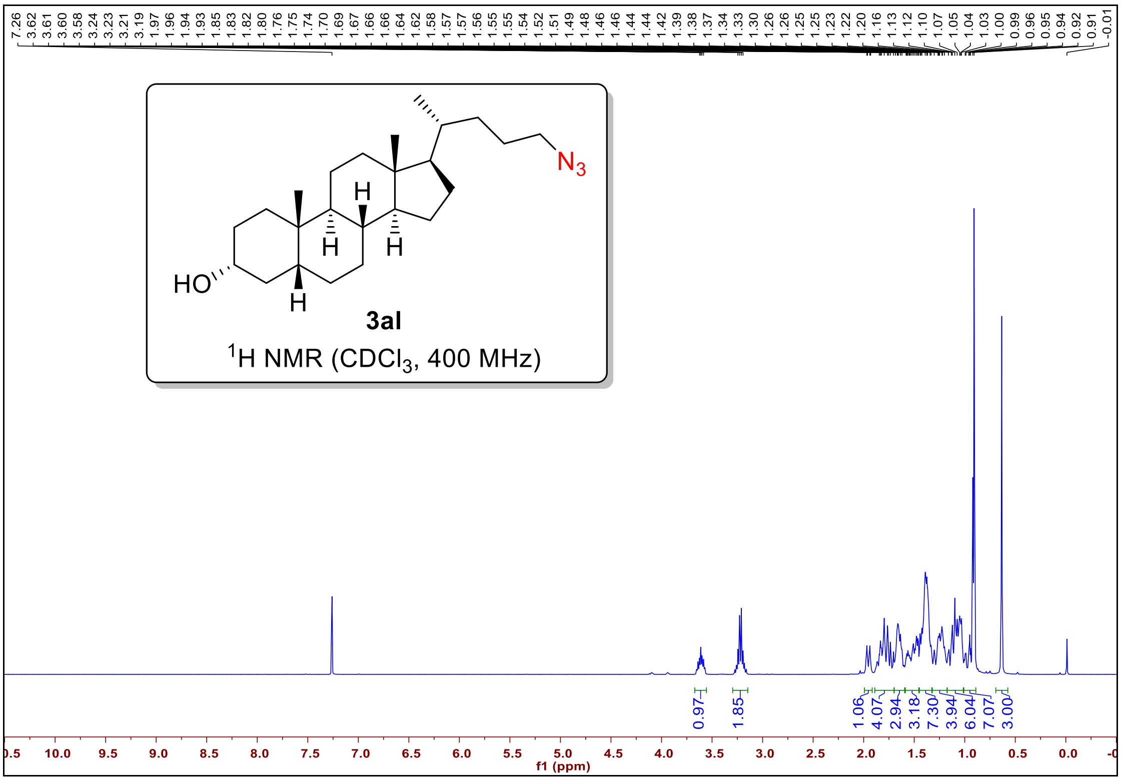

Supplementary Figure 92. ^{1}H **NMR Spectrum of 3al**

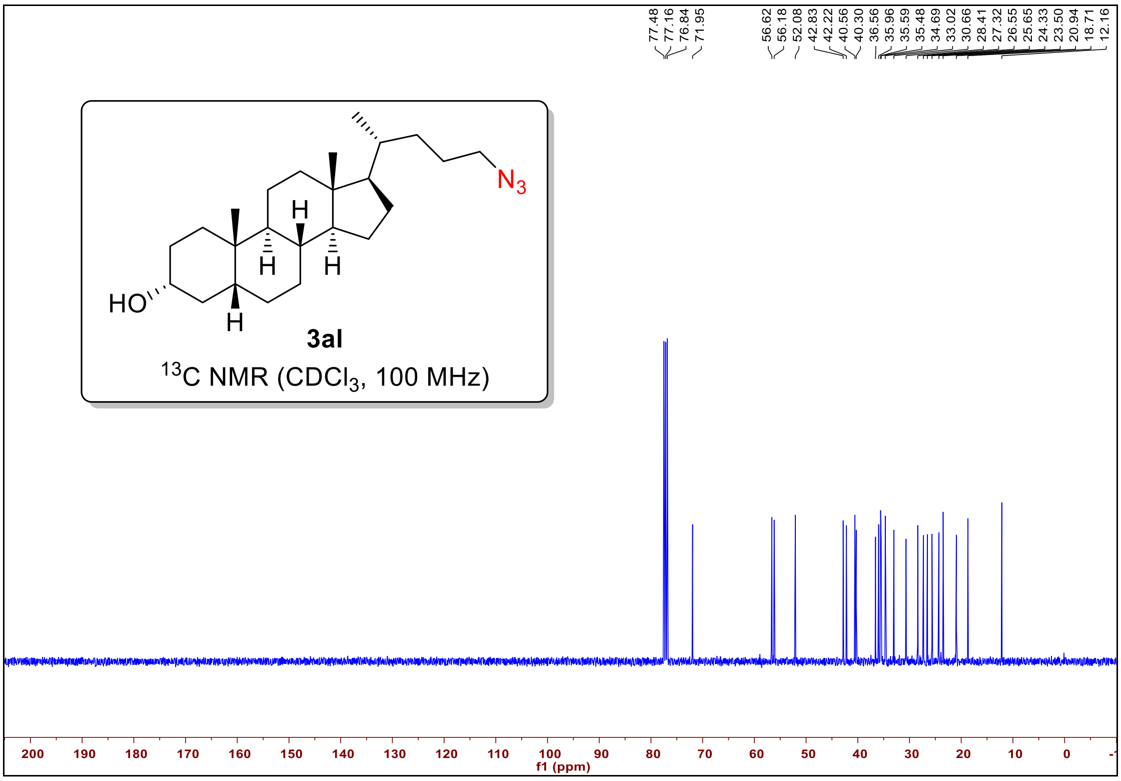

Supplementary Figure 93. ^{13}C NMR Spectrum of 3al

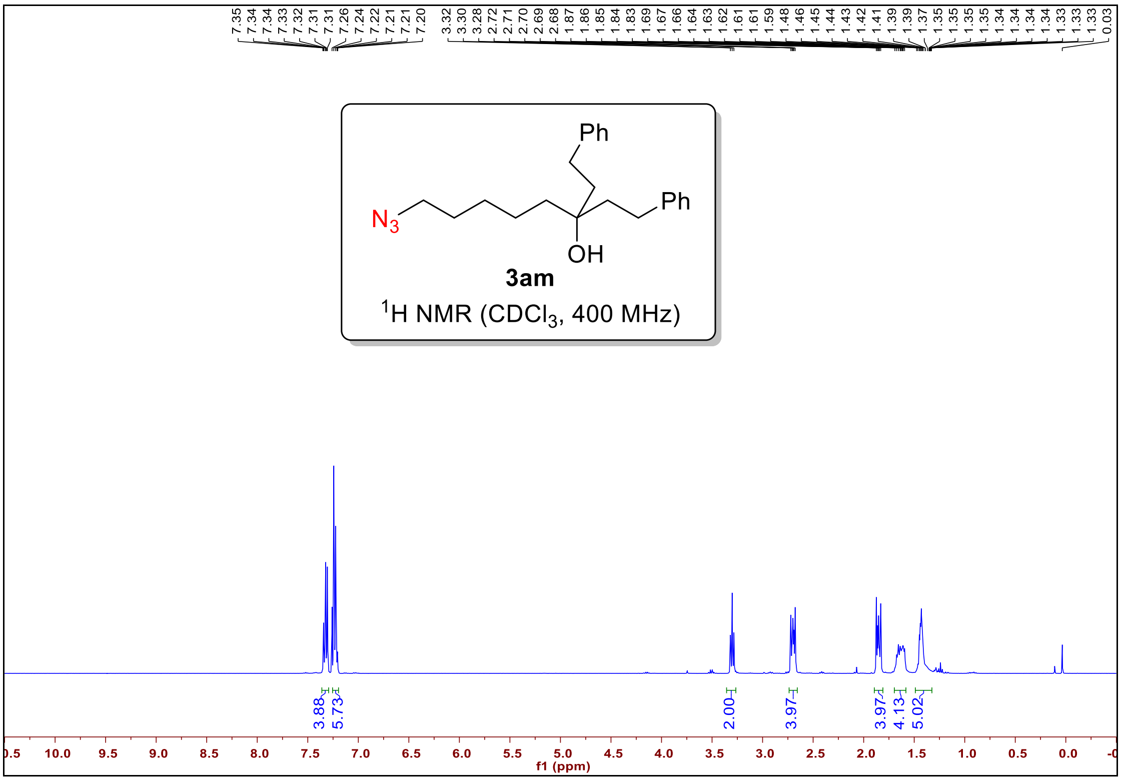

Supplementary Figure 94. ^{1}H NMR Spectrum of 3am

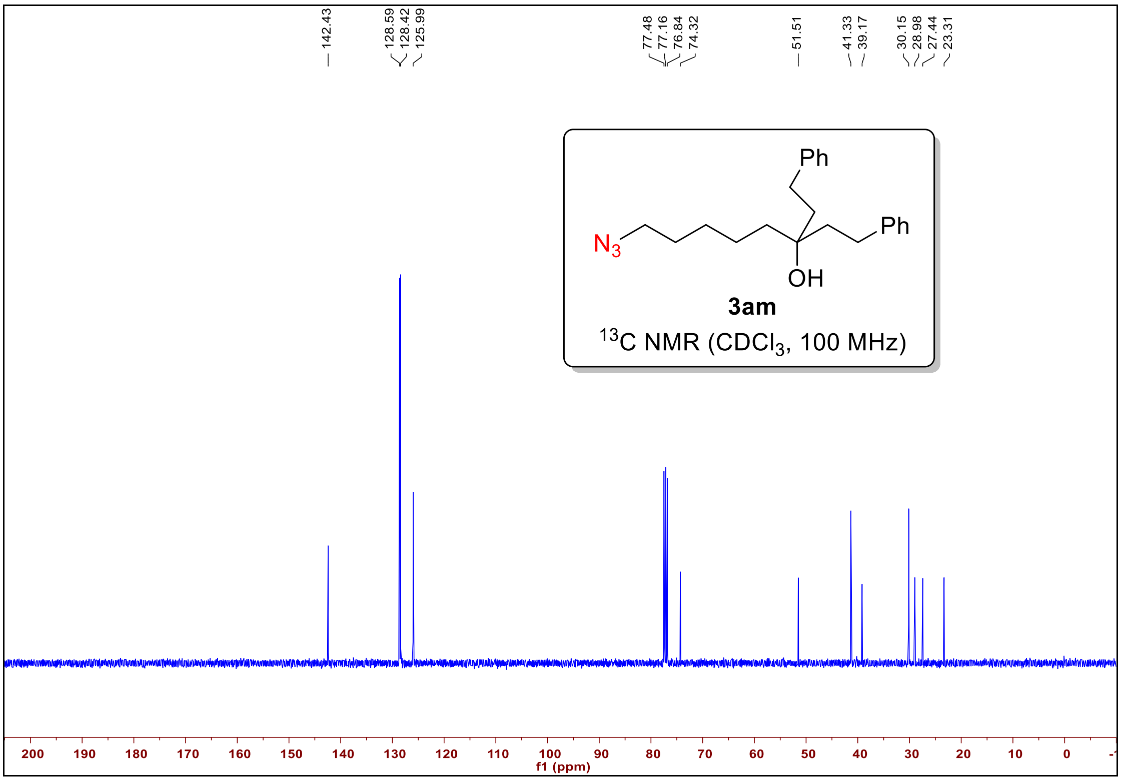

Supplementary Figure 95. ^{13}C NMR Spectrum of 3am

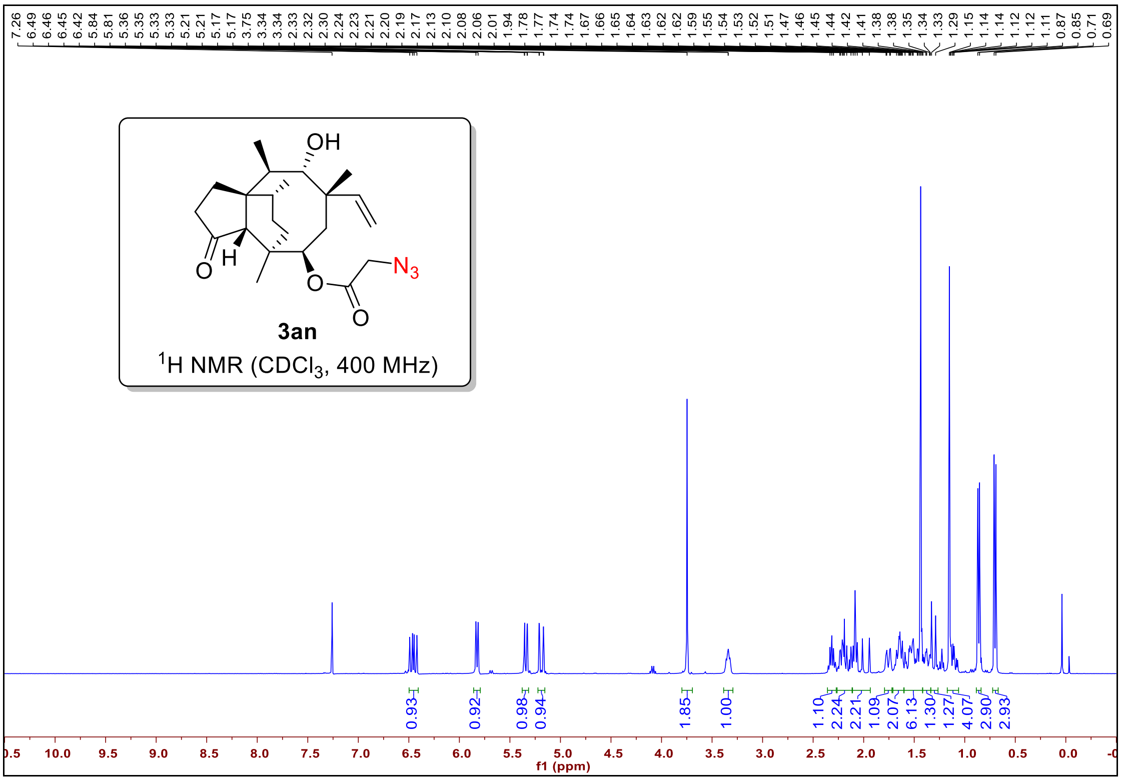

Supplementary Figure 96. ^{1}H NMR Spectrum of 3an

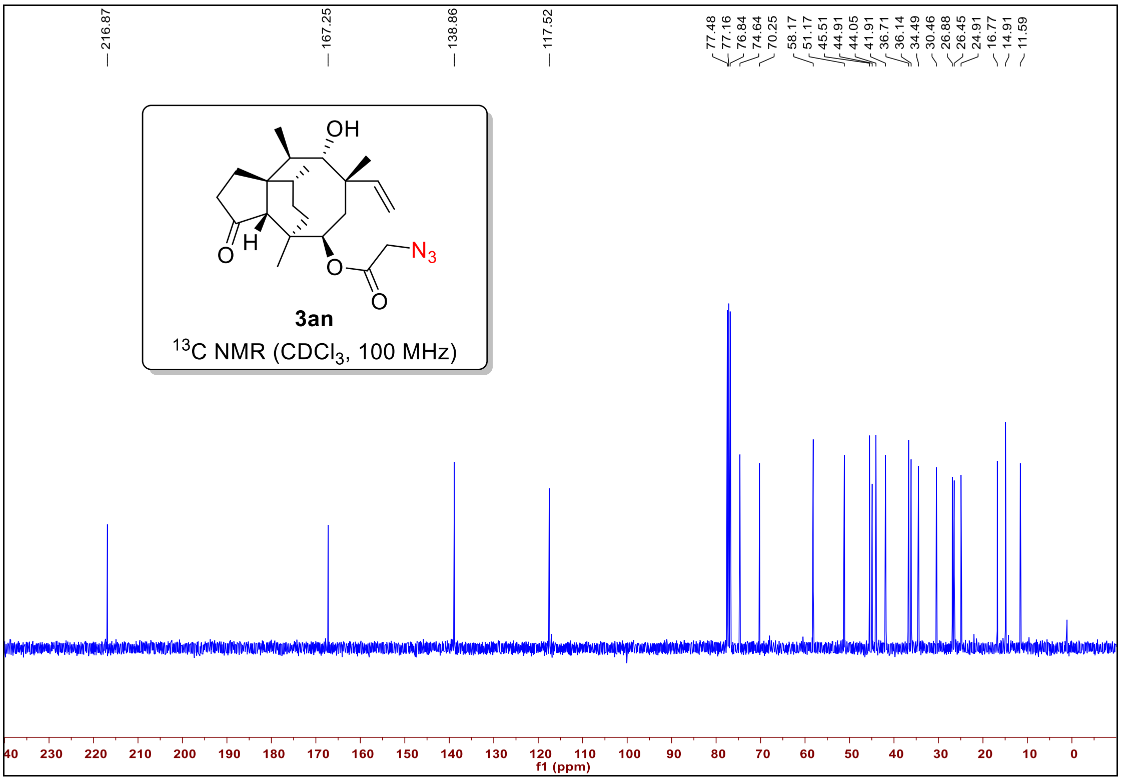

Supplementary Figure 97. ^{13}C NMR Spectrum of 3an

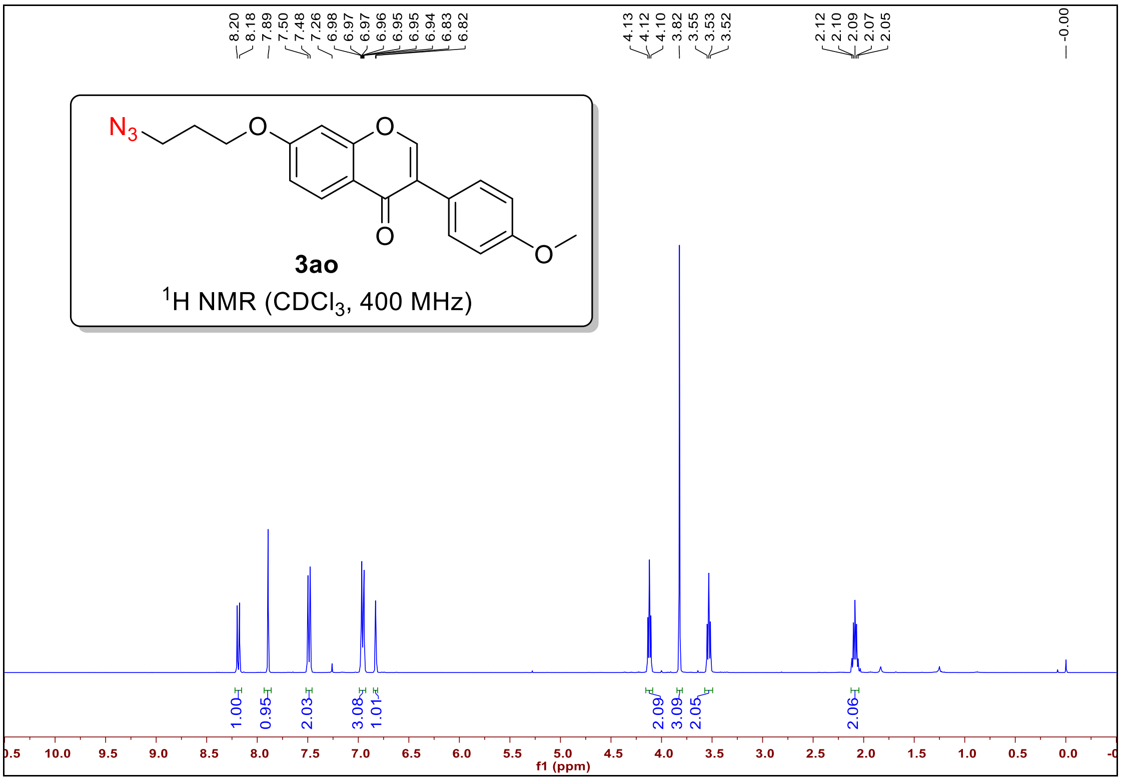

Supplementary Figure 98. ^{1}H NMR Spectrum of 3ao

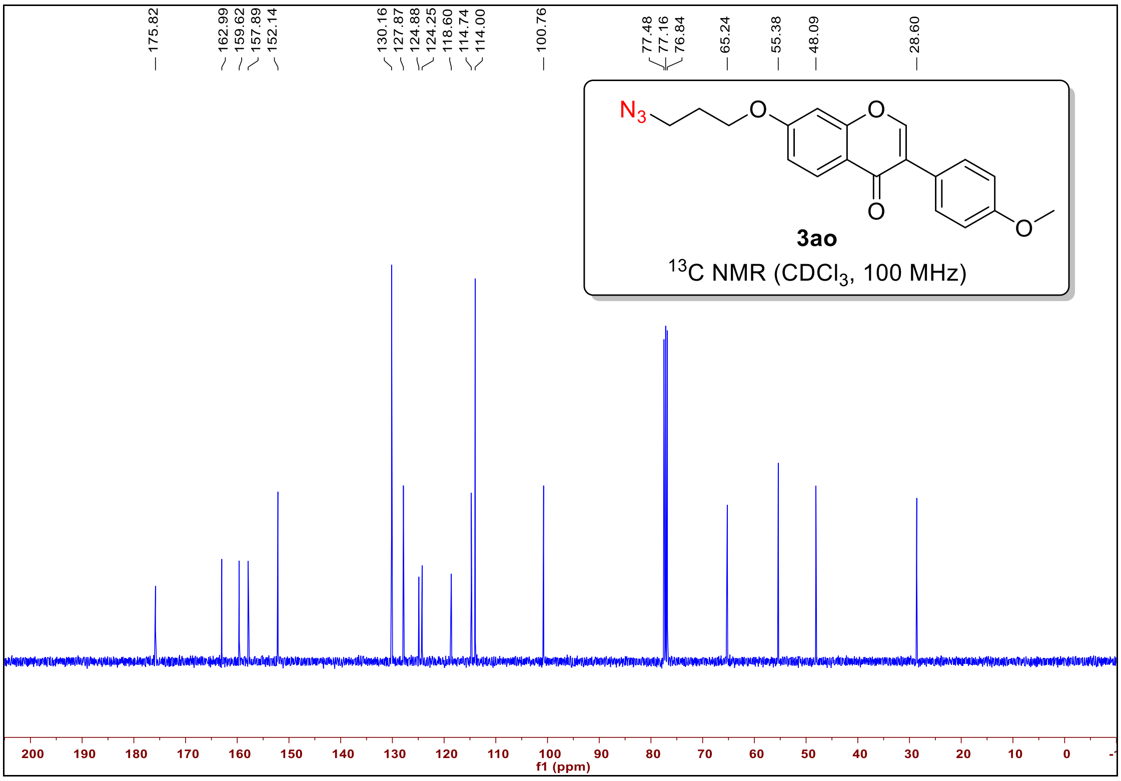

Supplementary Figure 99. ^{13}C NMR Spectrum of 3ao

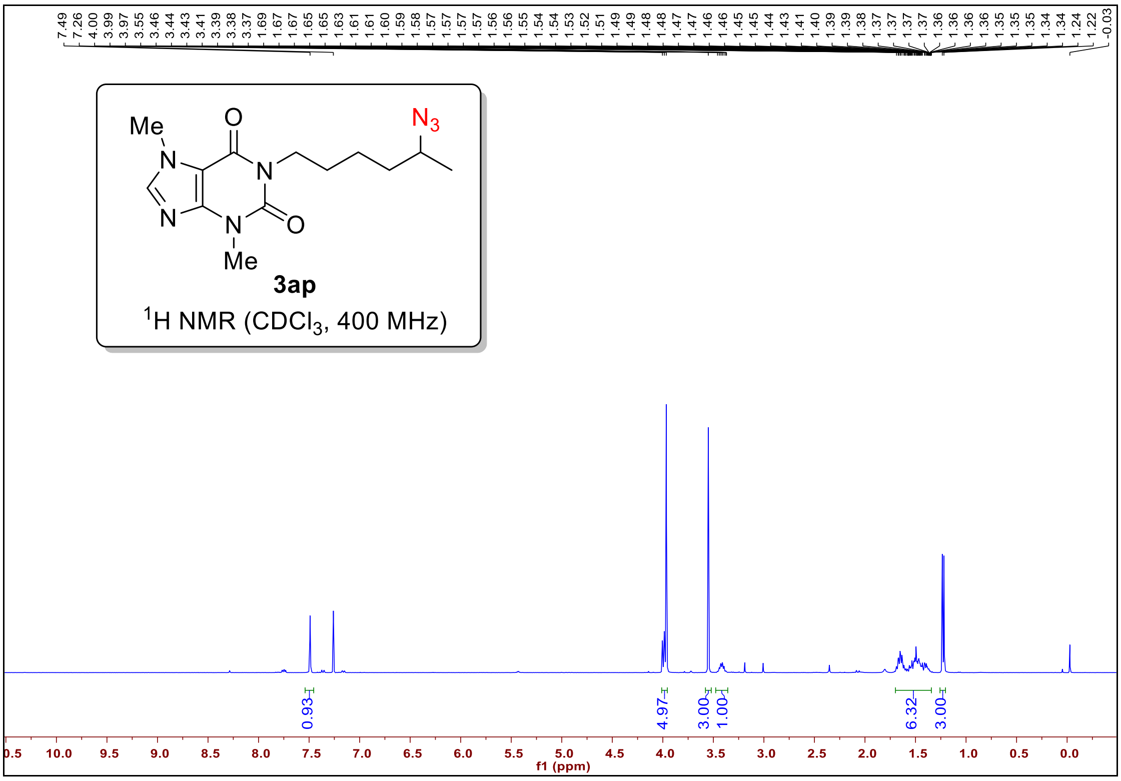

Supplementary Figure 100. ^{1}H NMR Spectrum of 3ap

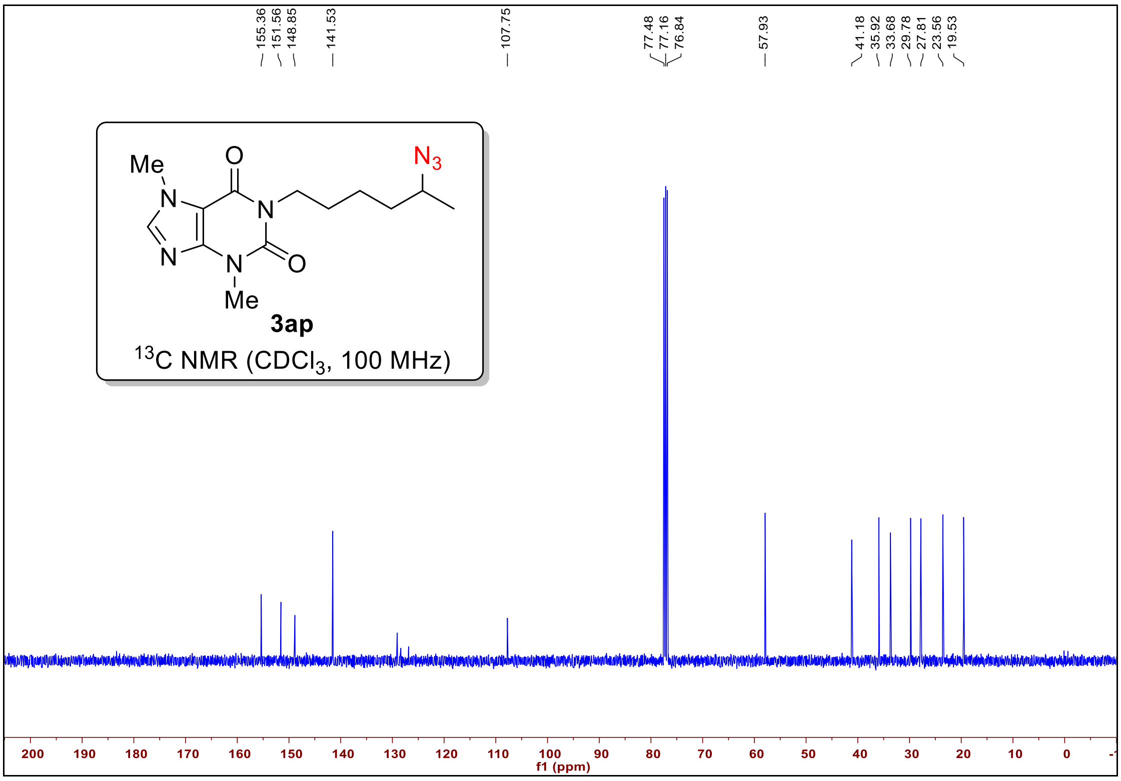

Supplementary Figure 101. ^{13}C **NMR Spectrum of 3ap**

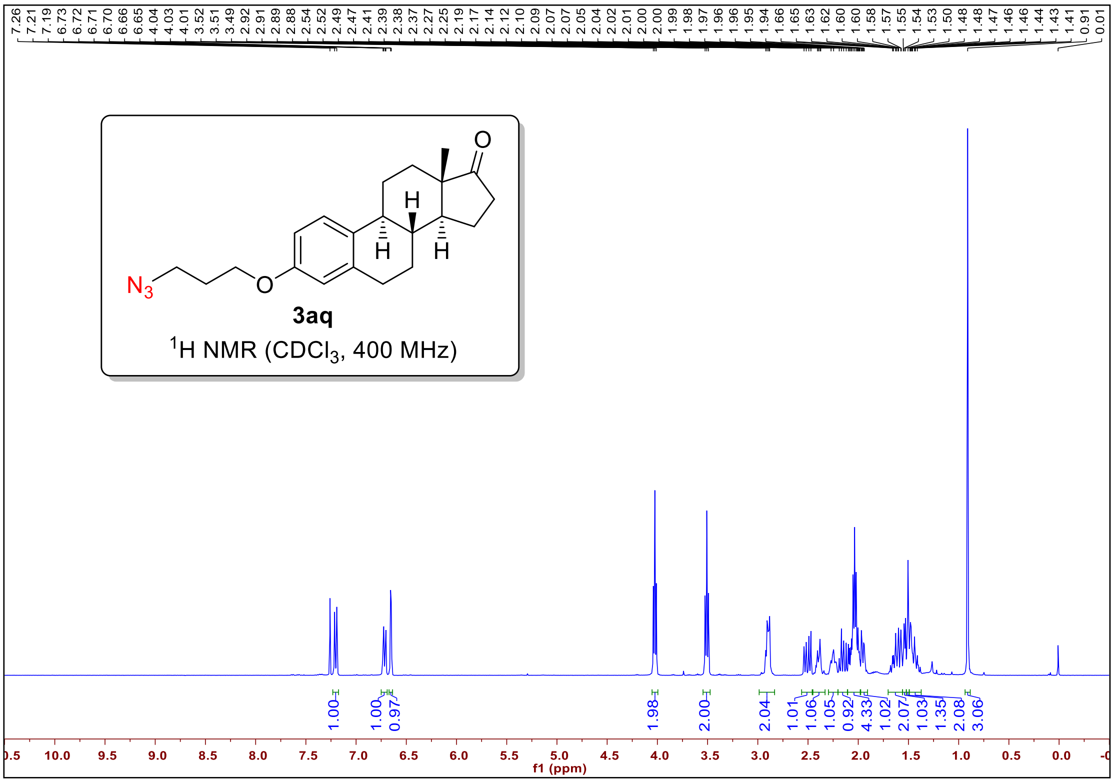

Supplementary Figure 102. ^{1}**H NMR Spectrum of 3aq**

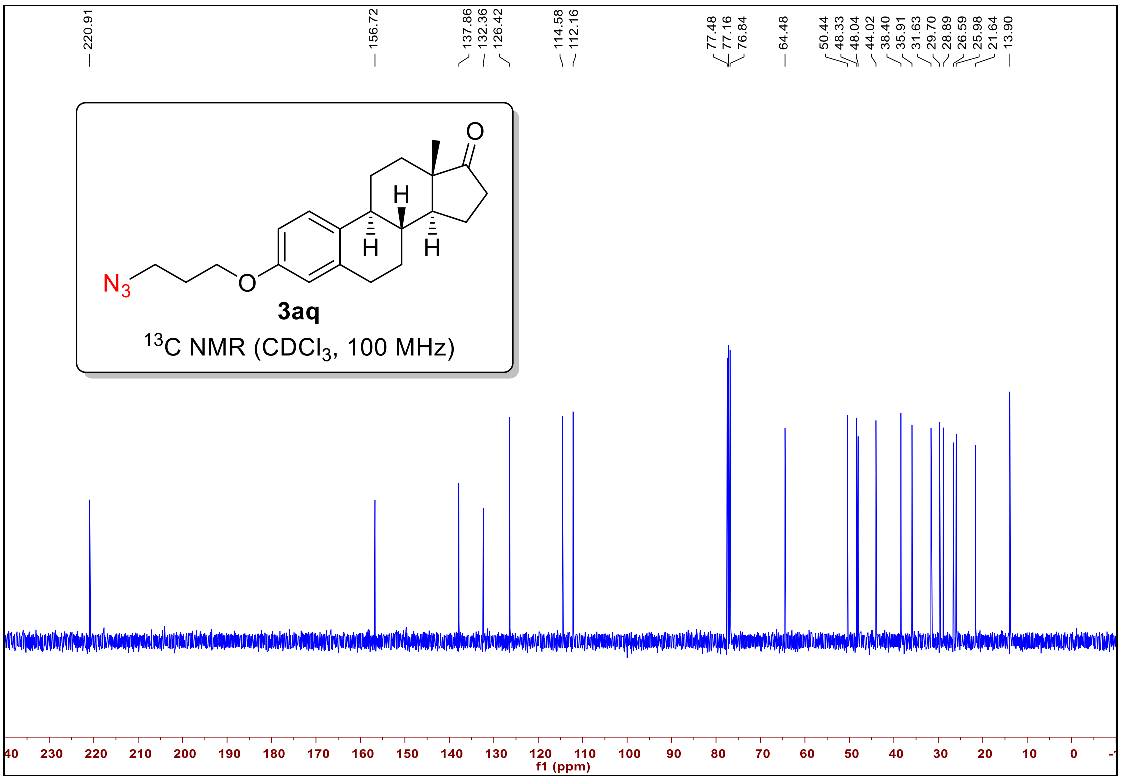

Supplementary Figure 103. ^{13}C NMR Spectrum of 3aq

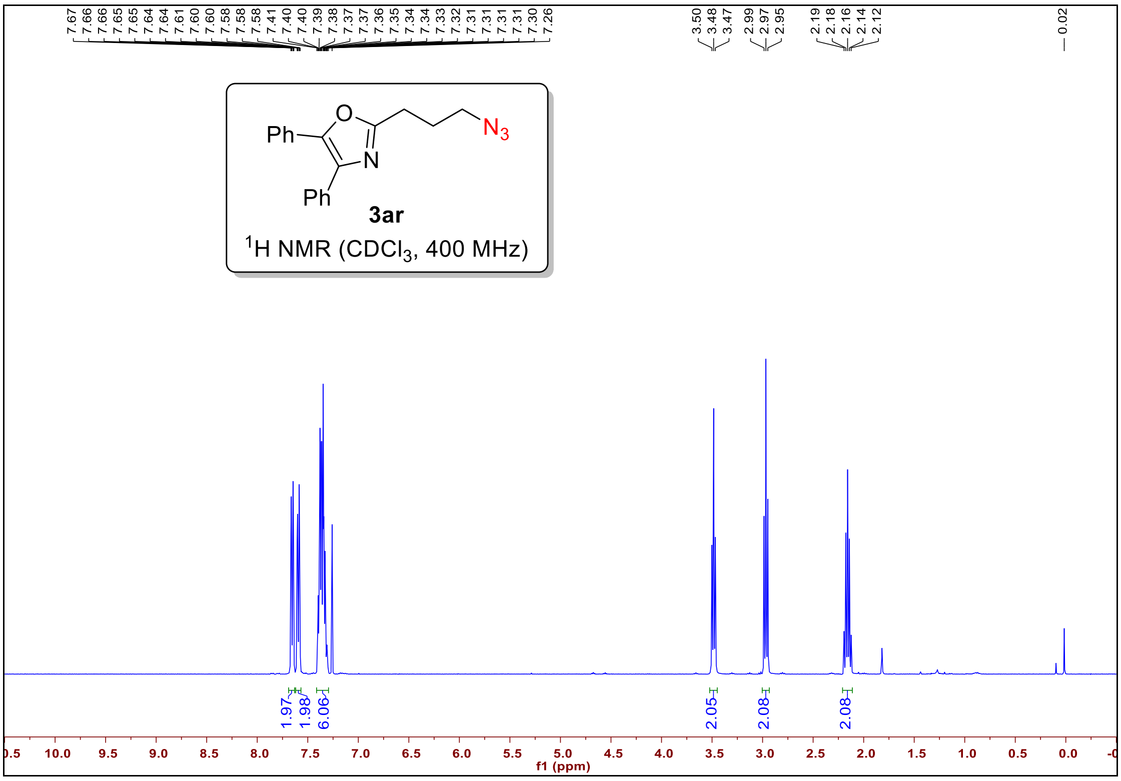

Supplementary Figure 104. ^{1}H NMR Spectrum of 3ar

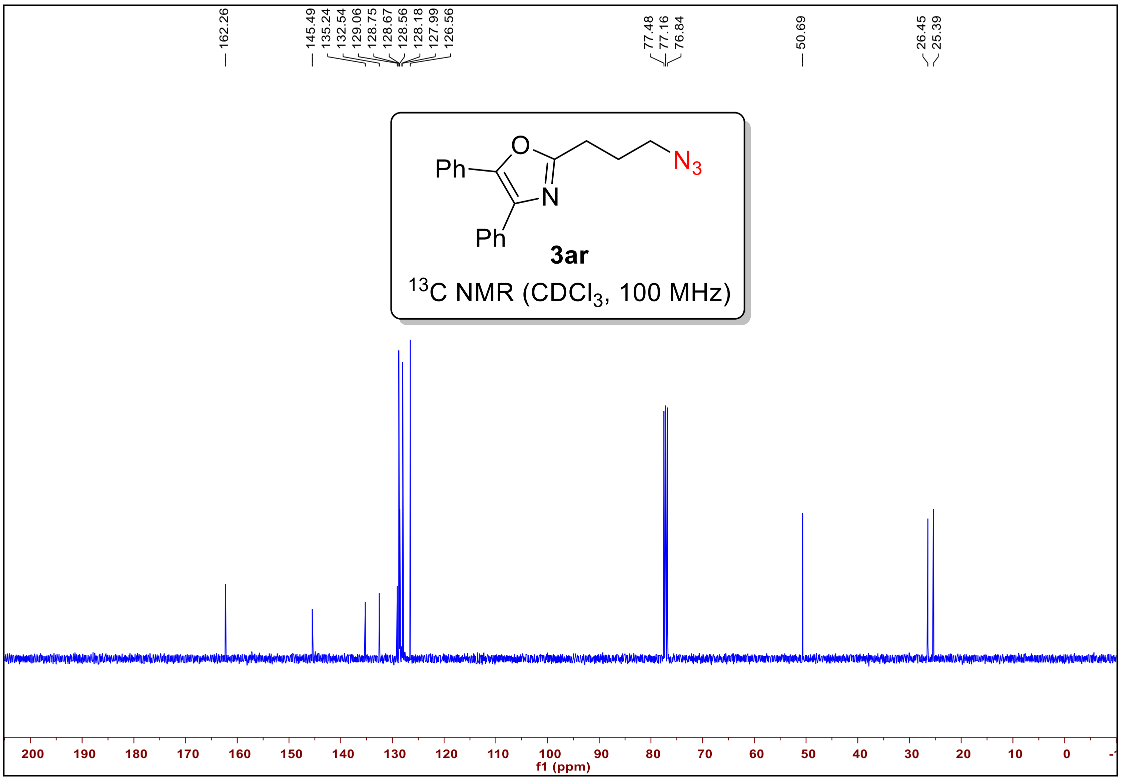

Supplementary Figure 105. ^{13}C NMR Spectrum of 3ar

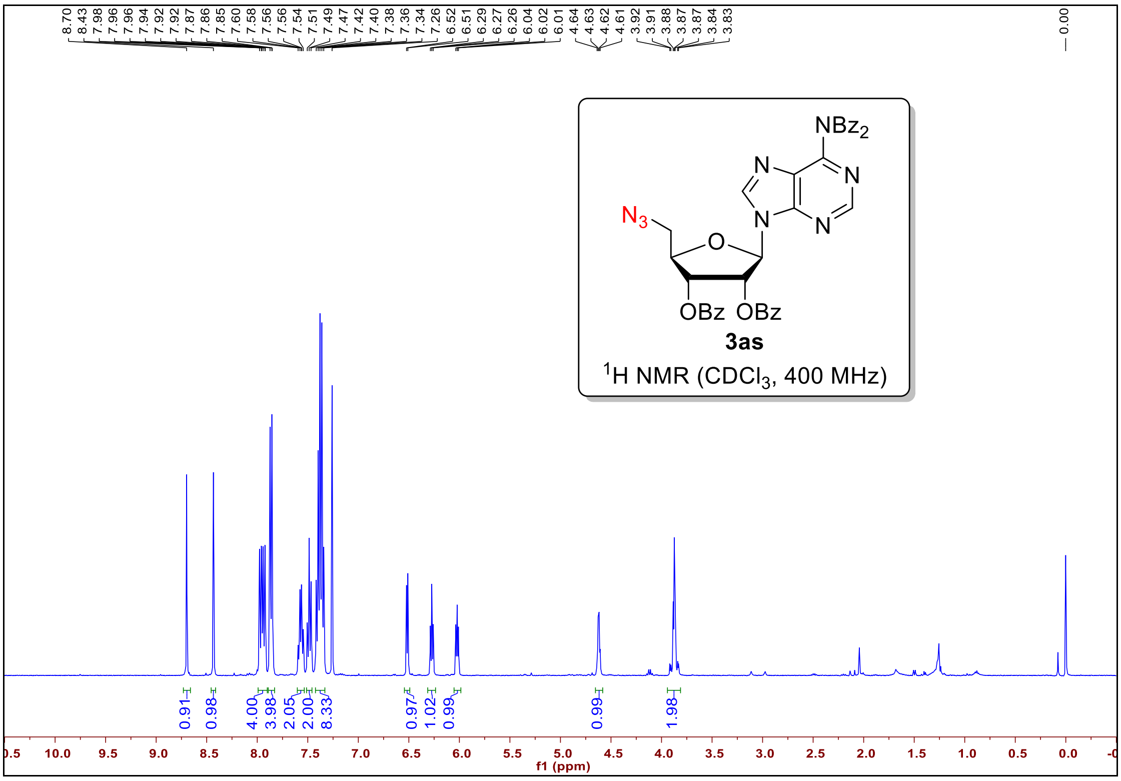

Supplementary Figure 106. ^{1}H NMR Spectrum of 3as

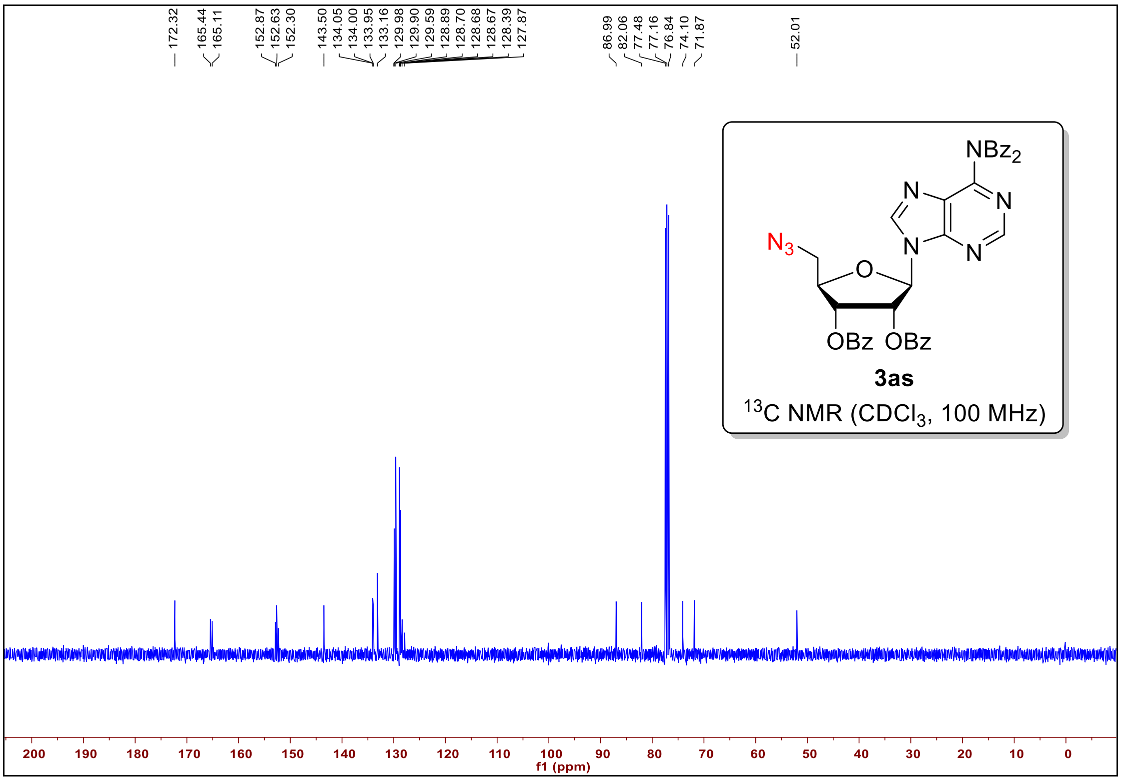

Supplementary Figure 107. ^{13}**C NMR Spectrum of 3as**

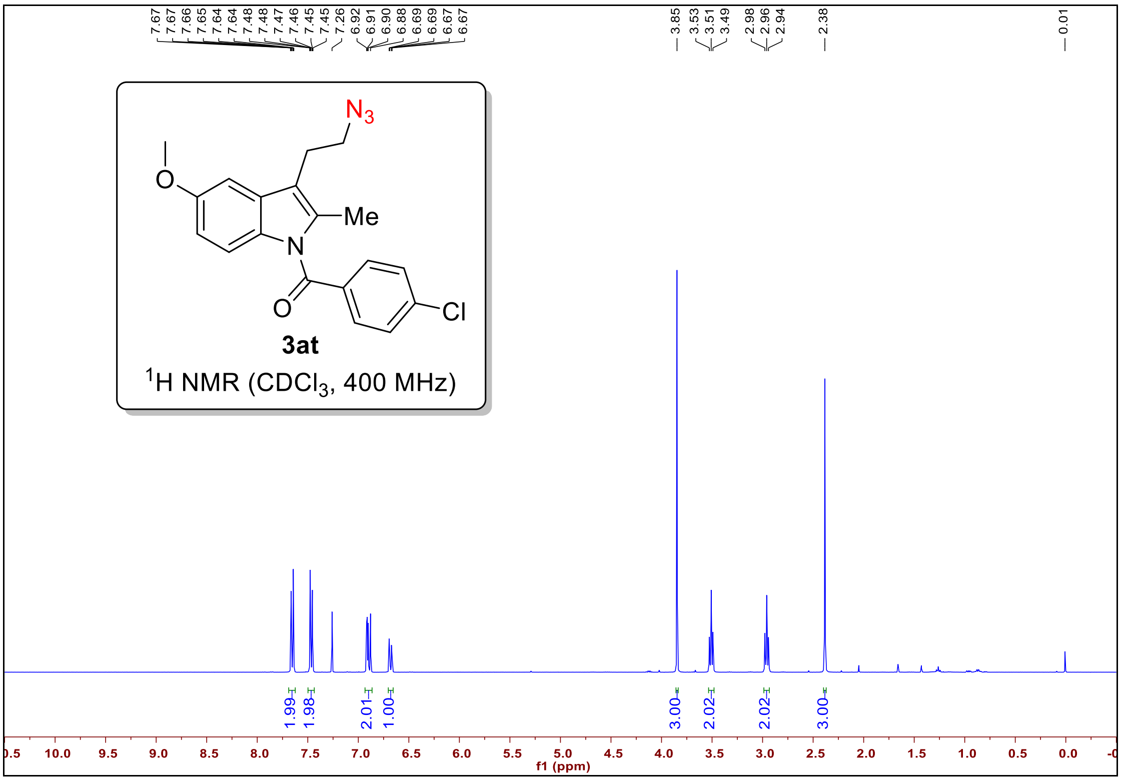

Supplementary Figure 108. ^{1}**H NMR Spectrum of 3at**

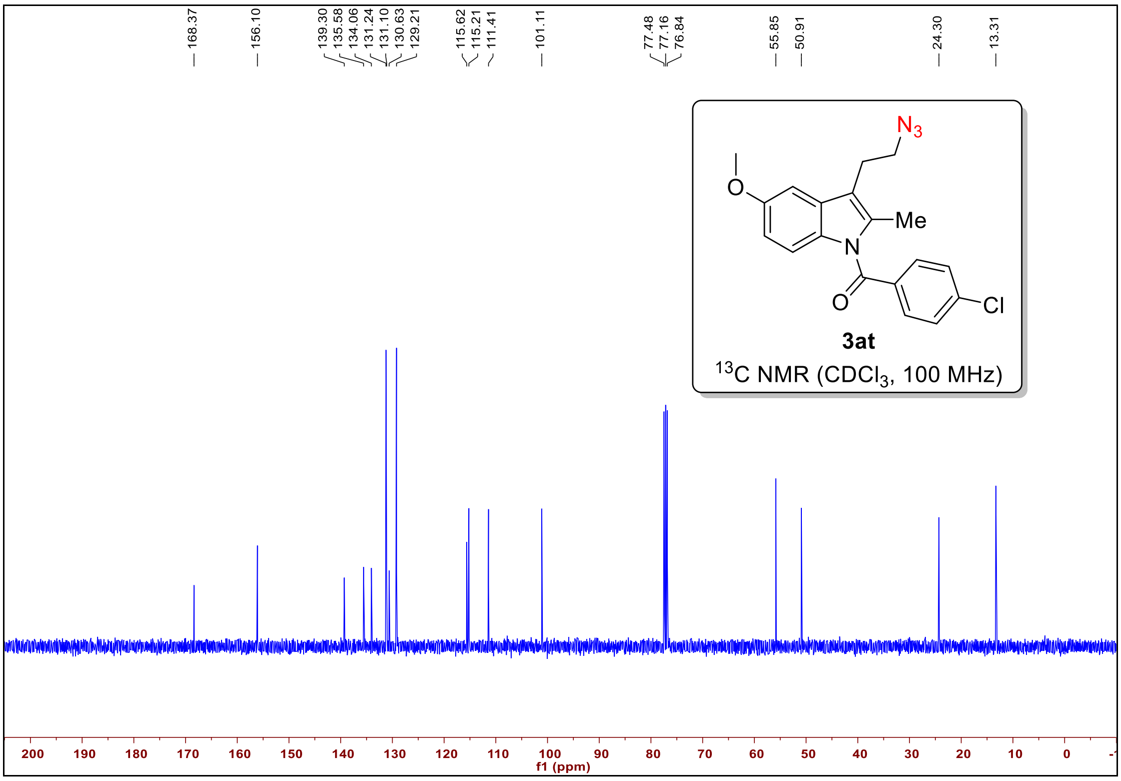

Supplementary Figure 109. ^{13}C NMR Spectrum of 3at

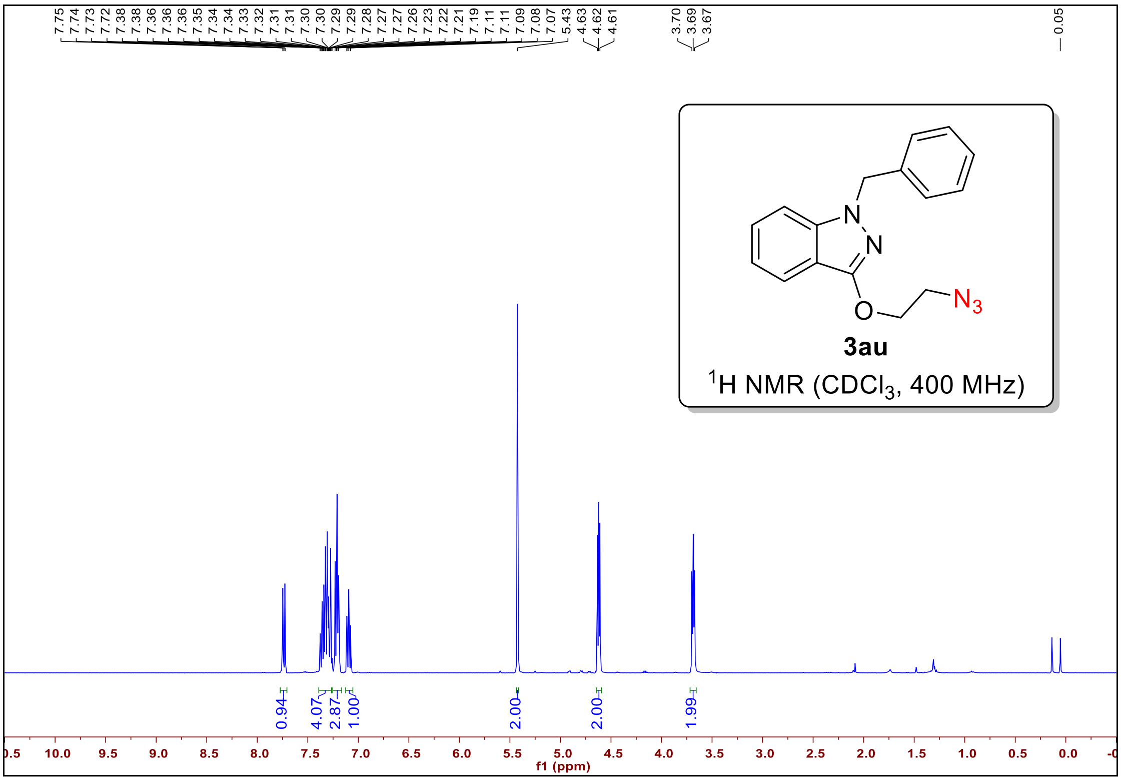

Supplementary Figure 110. ^{1}H NMR Spectrum of 3au

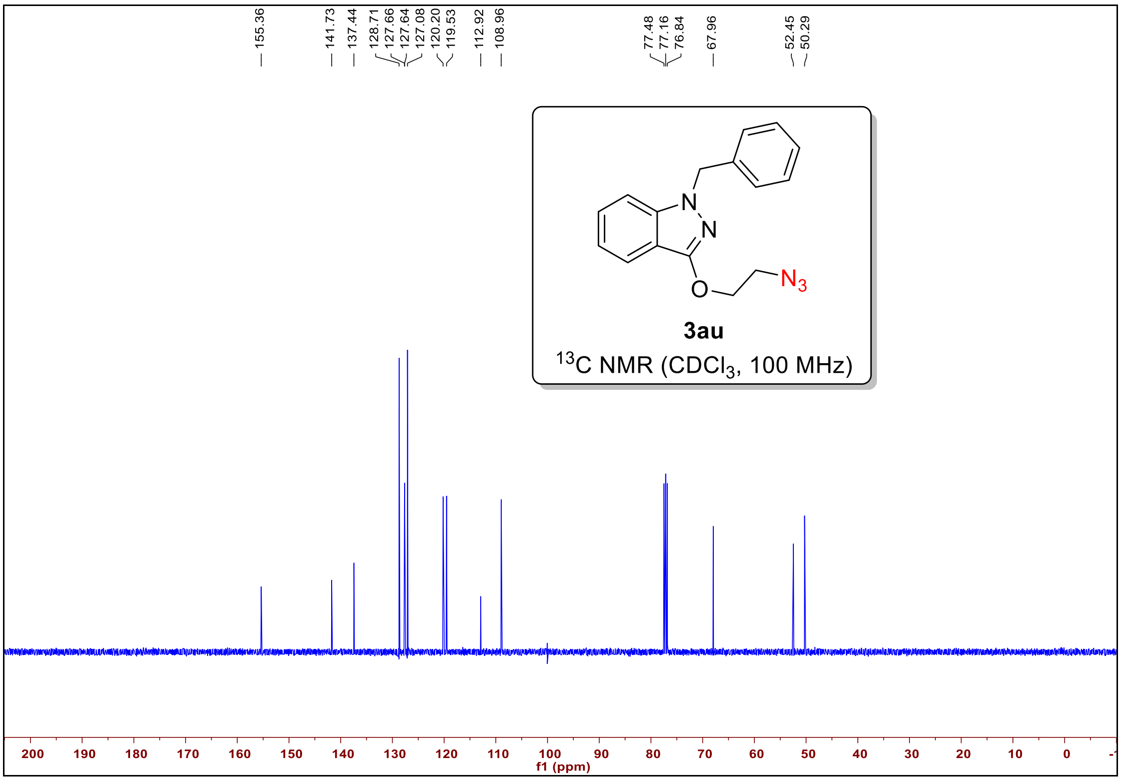

Supplementary Figure 111. ^{13}C NMR Spectrum of 3au

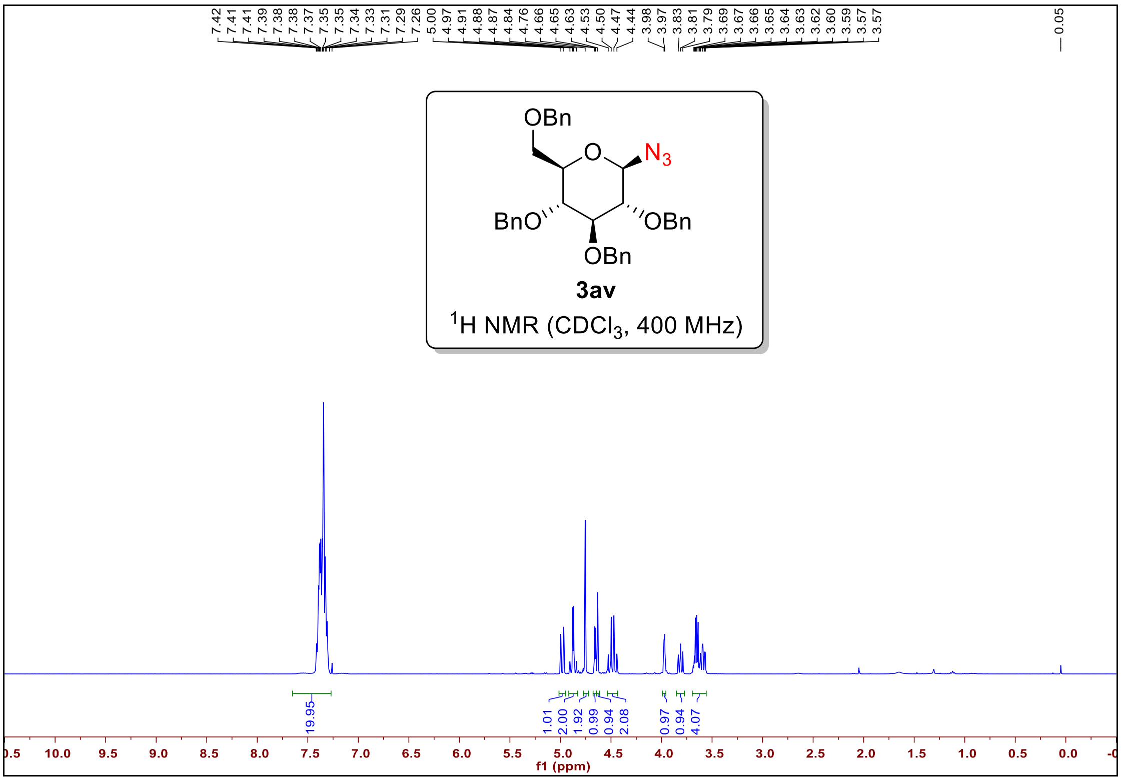

Supplementary Figure 112. ^{1}H NMR Spectrum of 3av

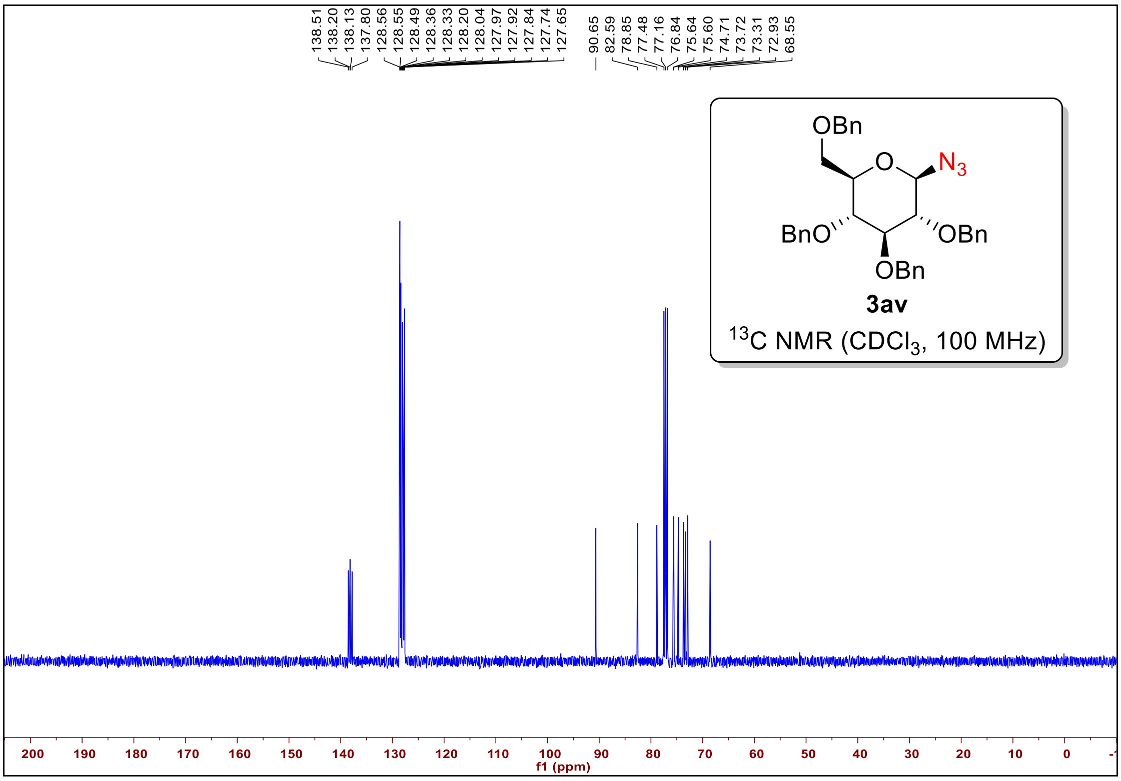

Supplementary Figure 113. ^{13}**C NMR Spectrum of 3av**

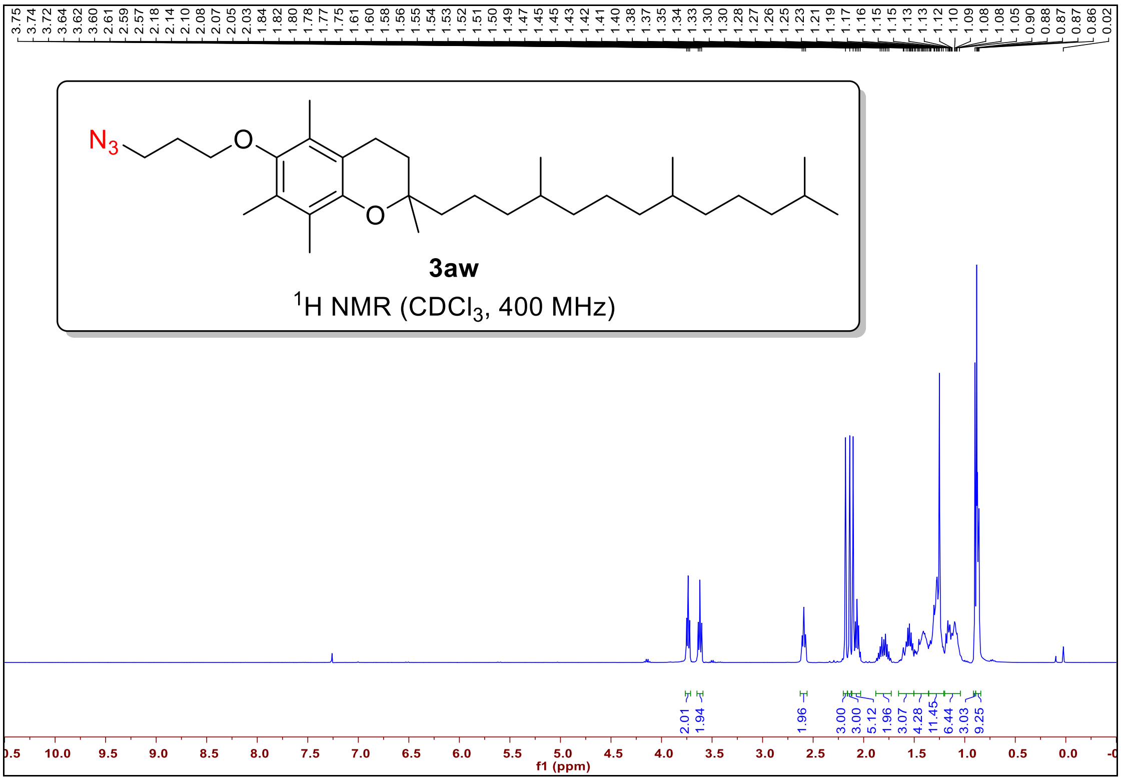

Supplementary Figure 114. ^{1}**H NMR Spectrum of 3aw**

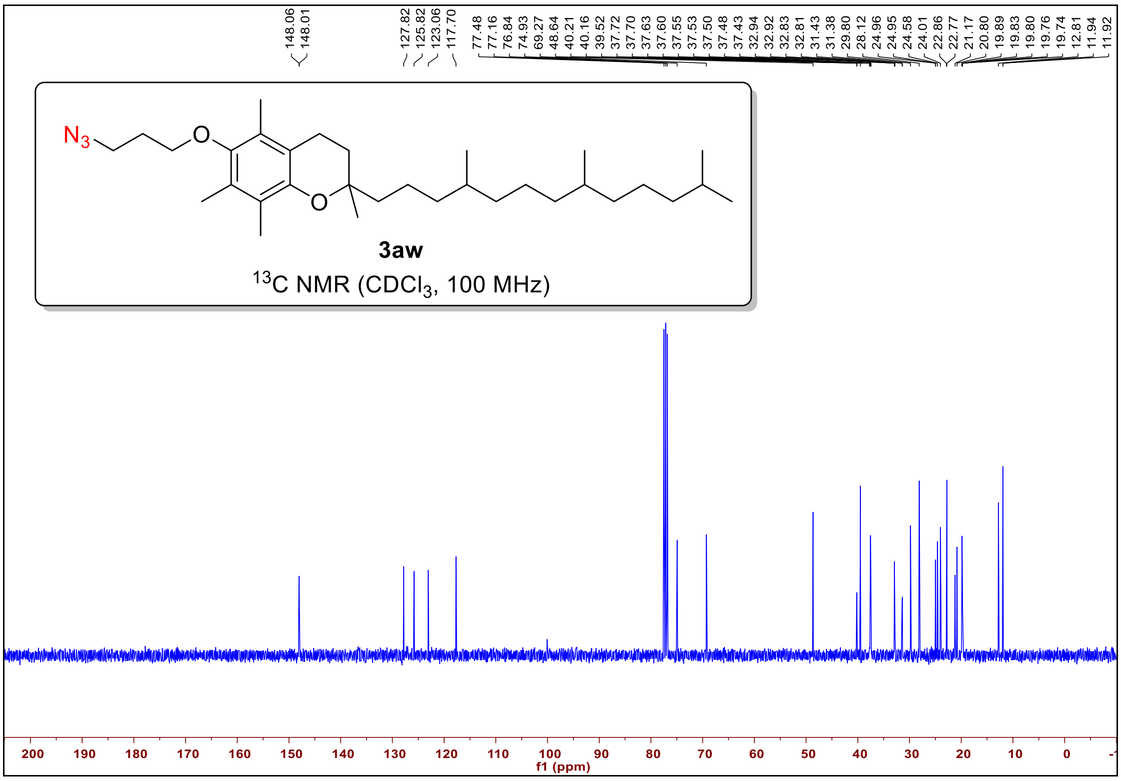

Supplementary Figure 115. ^{13}C NMR Spectrum of 3aw

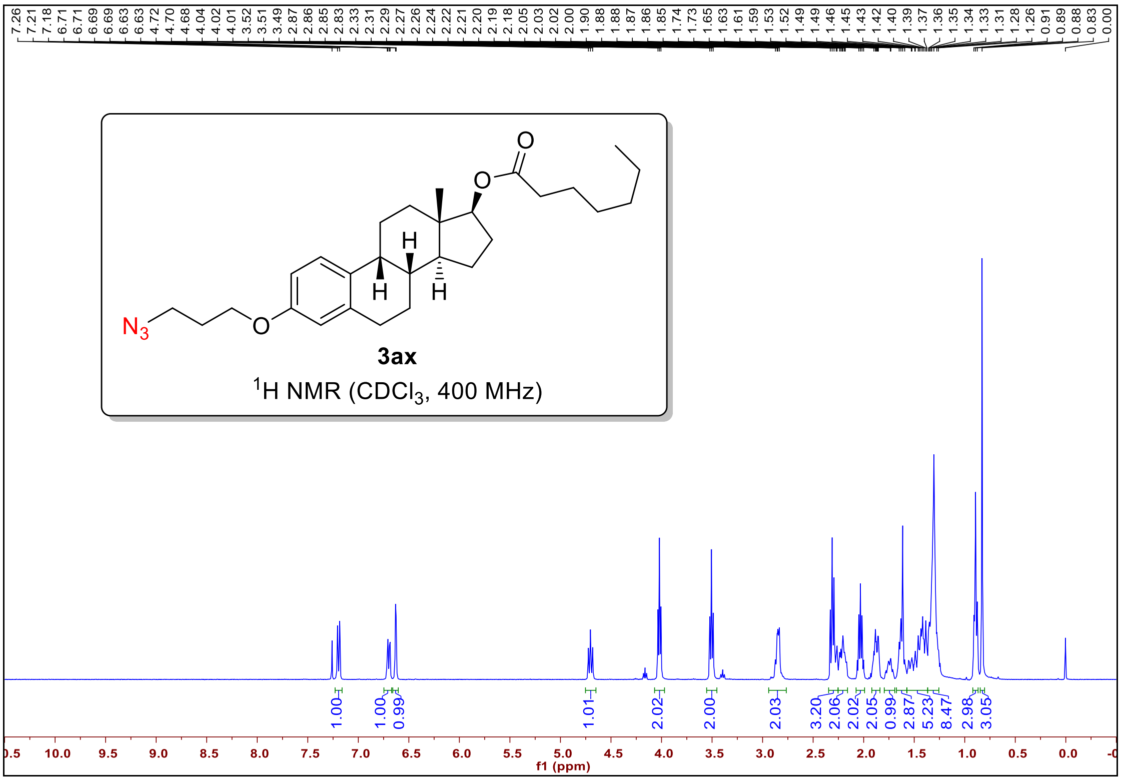

Supplementary Figure 116. ^{1}H NMR Spectrum of 3ax

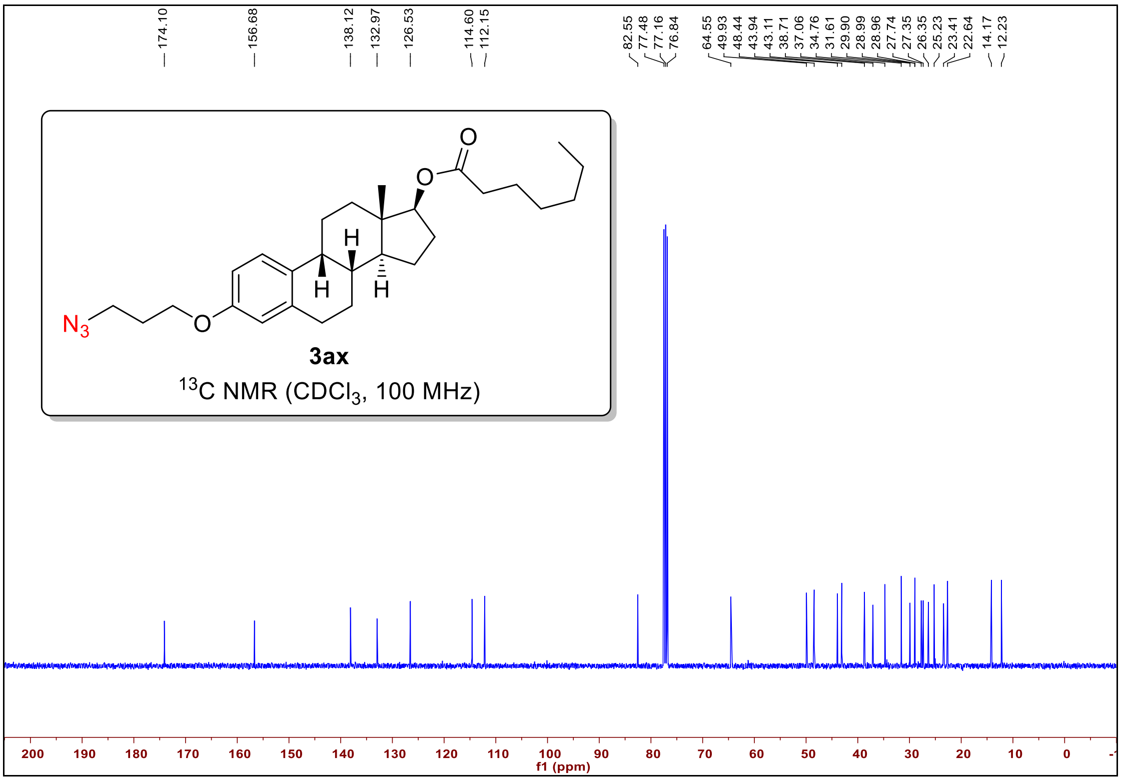

Supplementary Figure 117. ^{13}C NMR Spectrum of 3ax

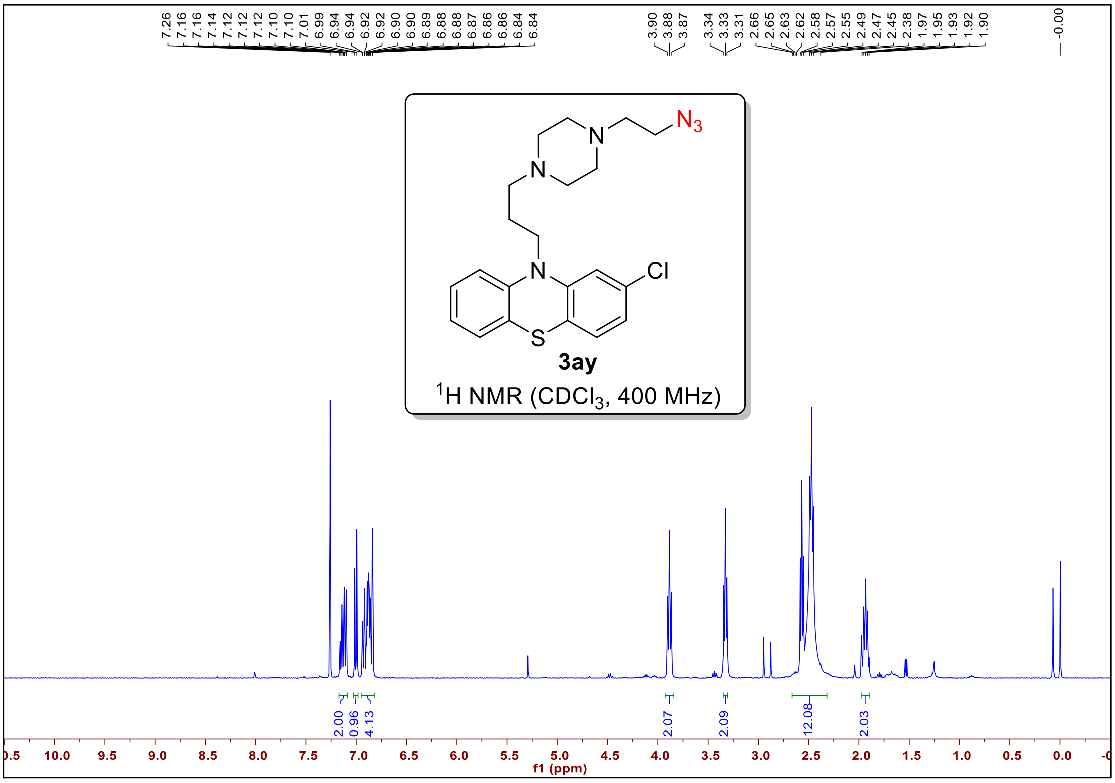

Supplementary Figure 118. ^{1}H NMR Spectrum of 3ay

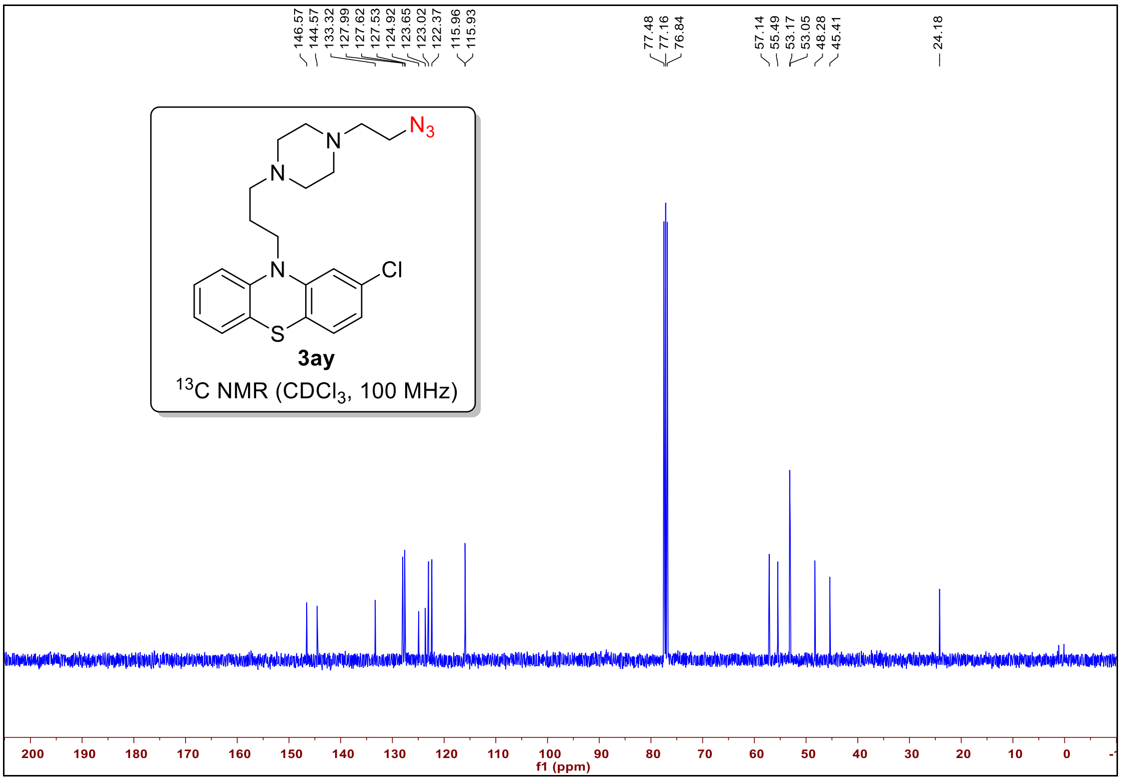

Supplementary Figure 119. ^{13}C NMR Spectrum of 3ay

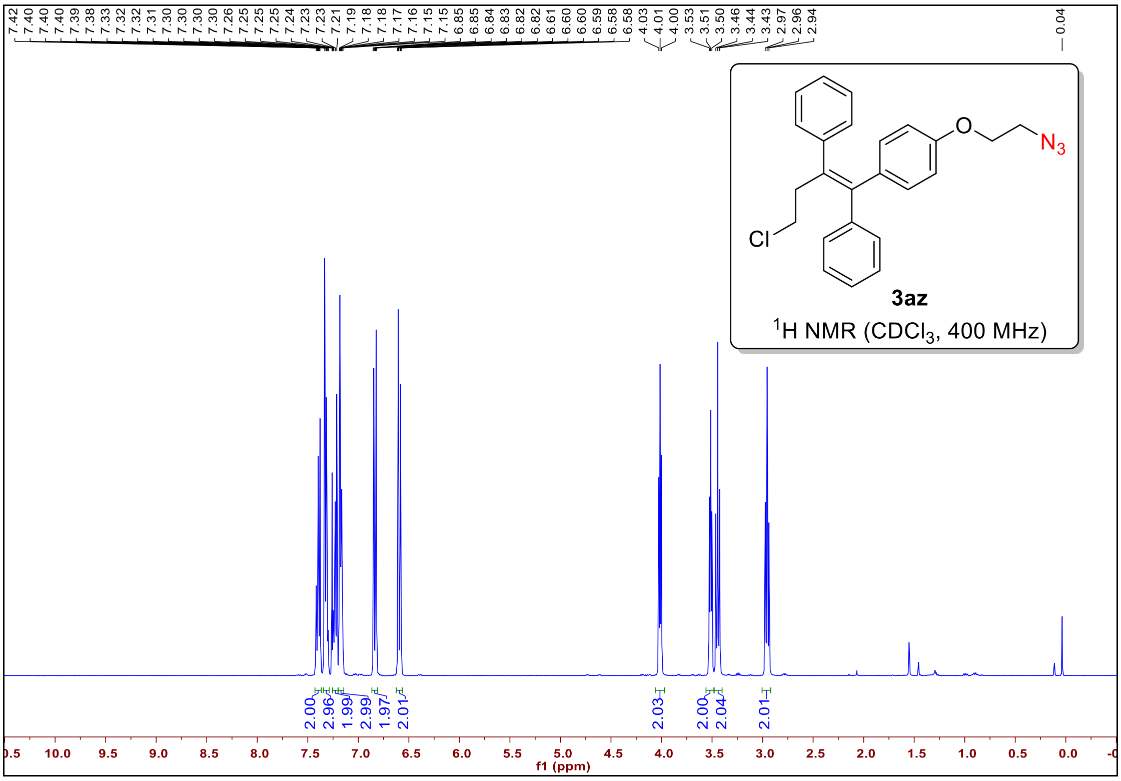

Supplementary Figure 120. ^{1}H NMR Spectrum of 3az

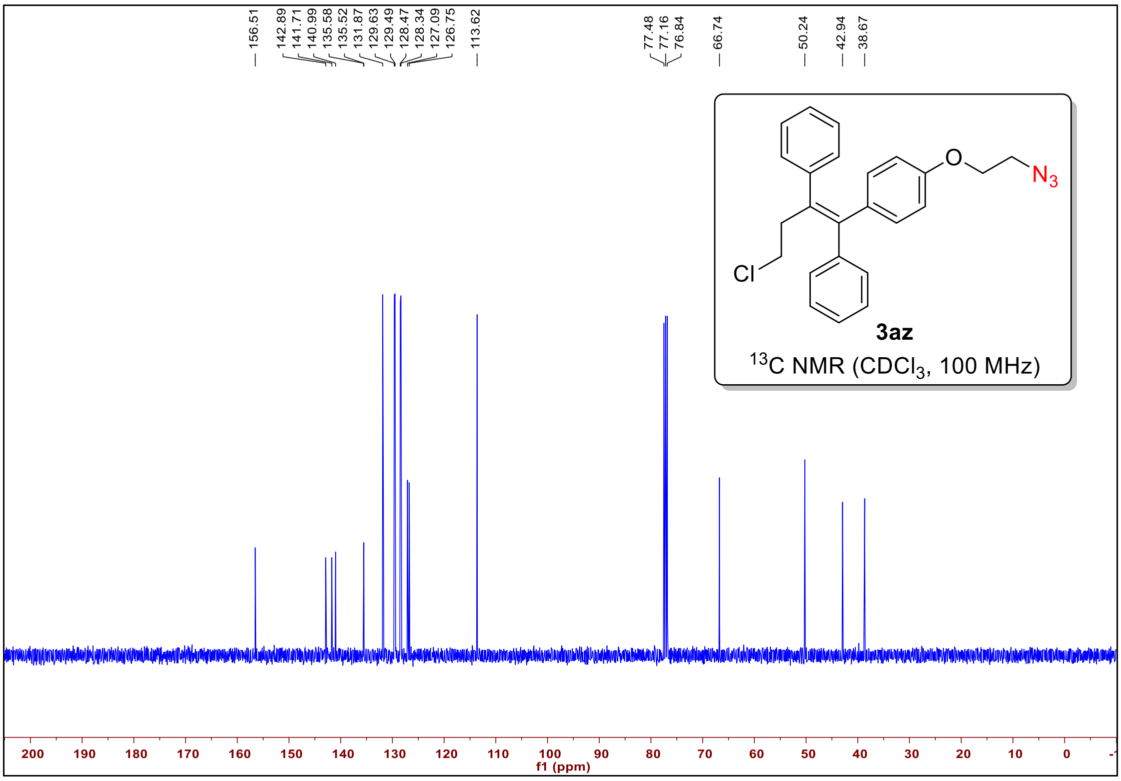

Supplementary Figure 121. ^{13}C NMR Spectrum of 3az

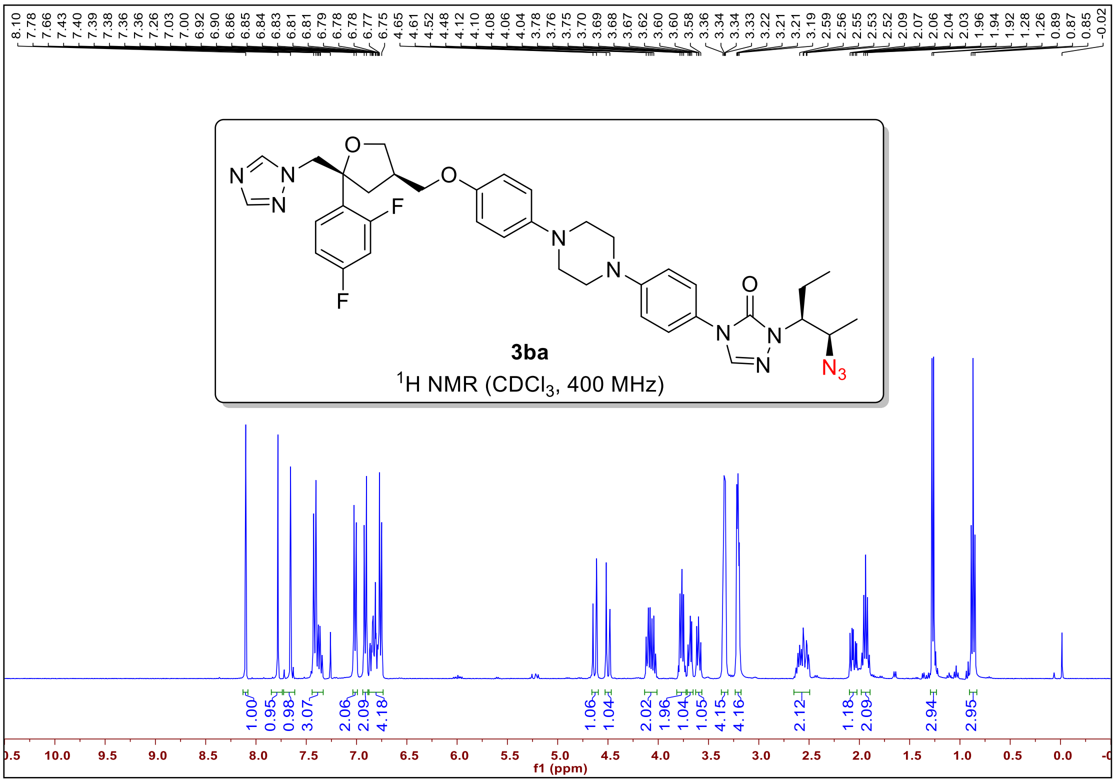

Supplementary Figure 122. ^{1}H NMR Spectrum of 3ba

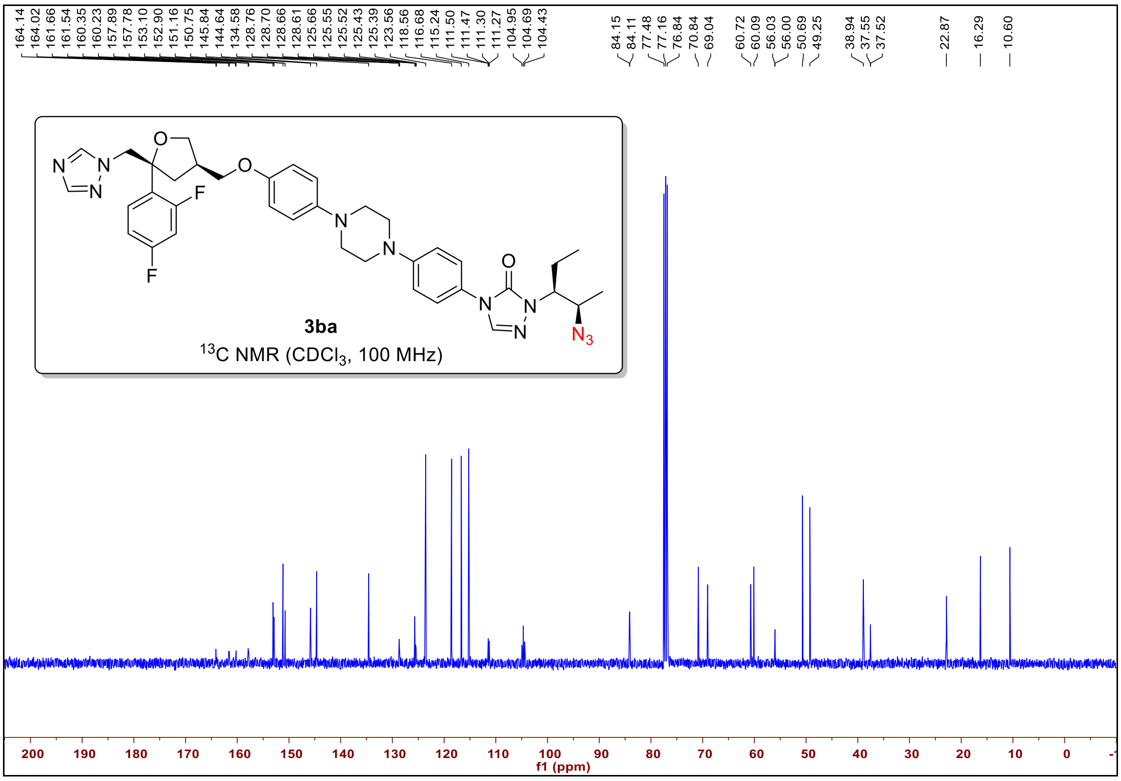

Supplementary Figure 123. ^{13}C NMR Spectrum of 3ba

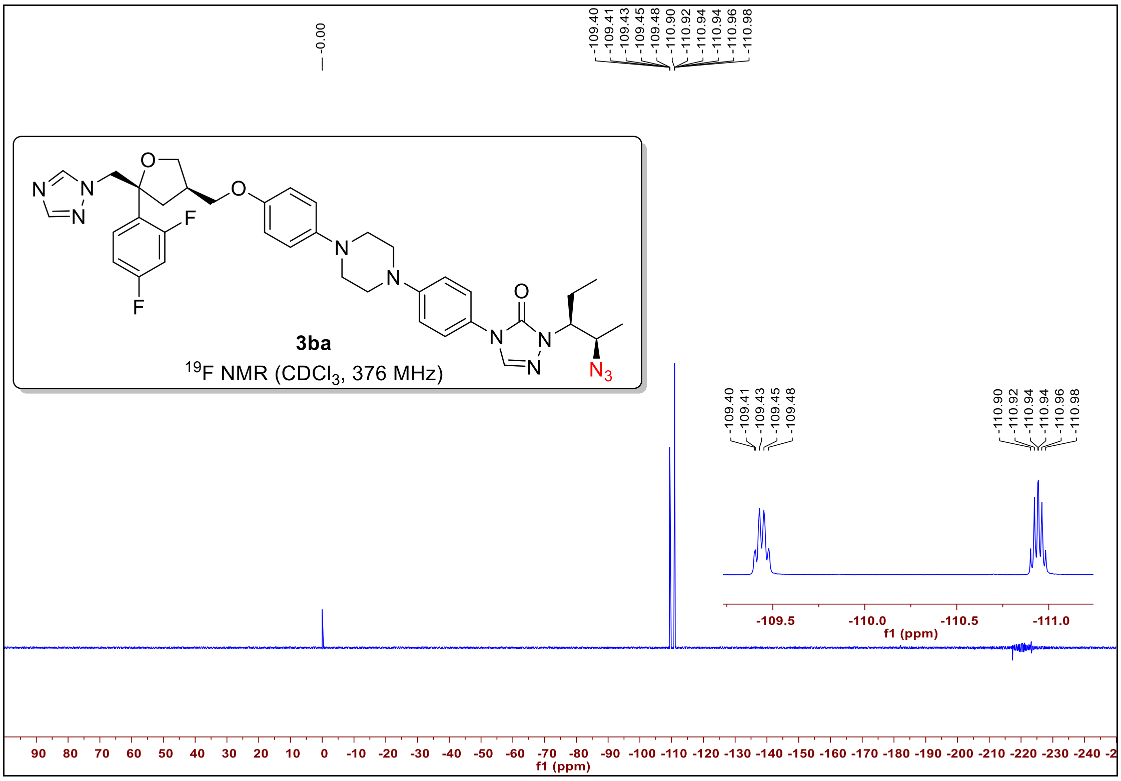

Supplementary Figure 124. ^{19}F NMR Spectrum of 3ba

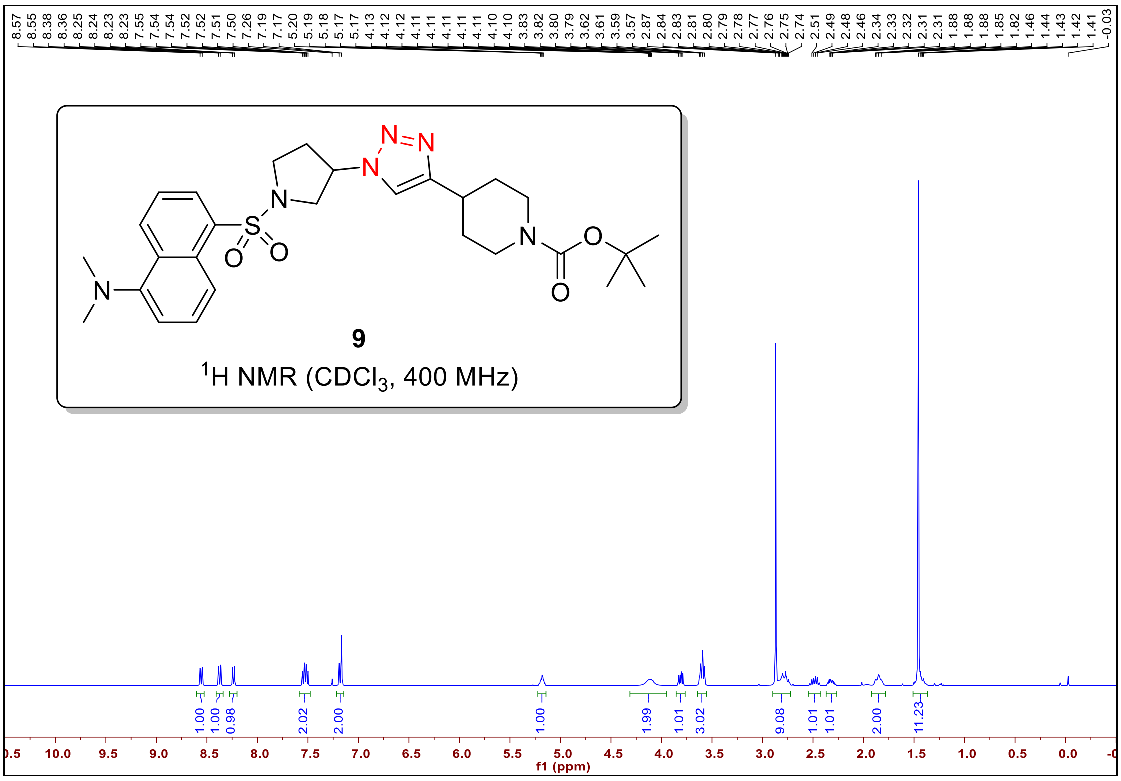

Supplementary Figure 125. ^{1}H NMR Spectrum of 9

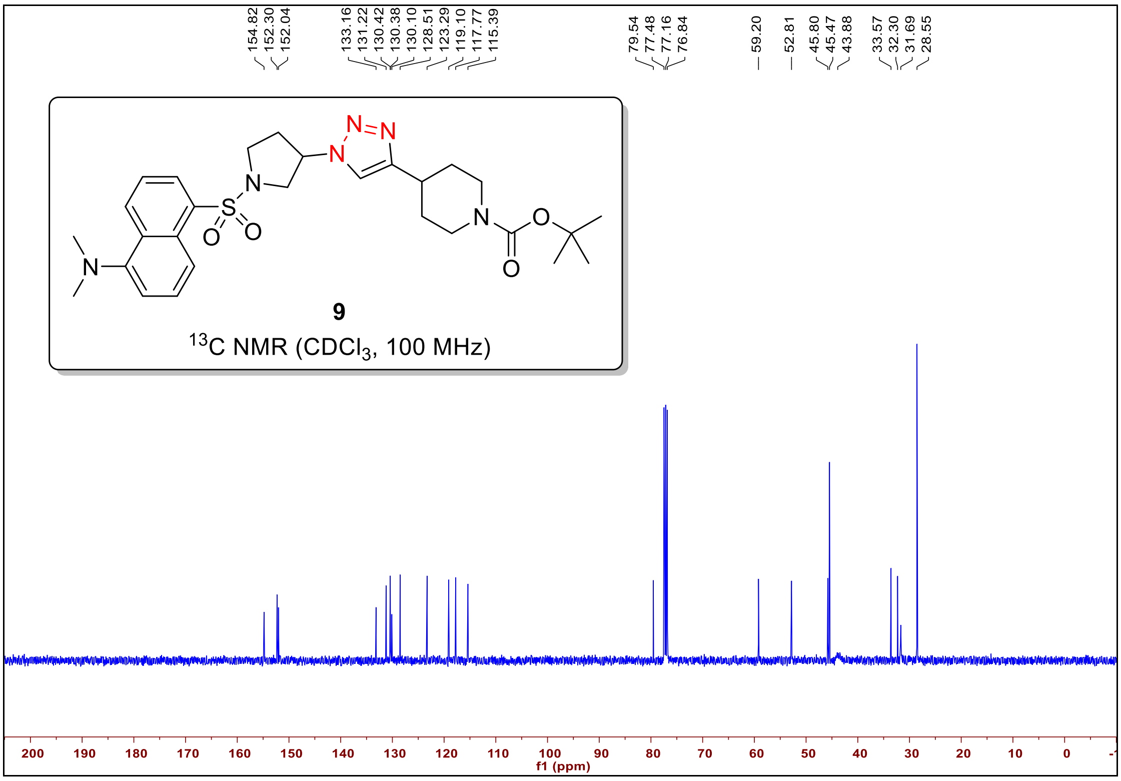

Supplementary Figure 126. ^{13}C NMR Spectrum of 9

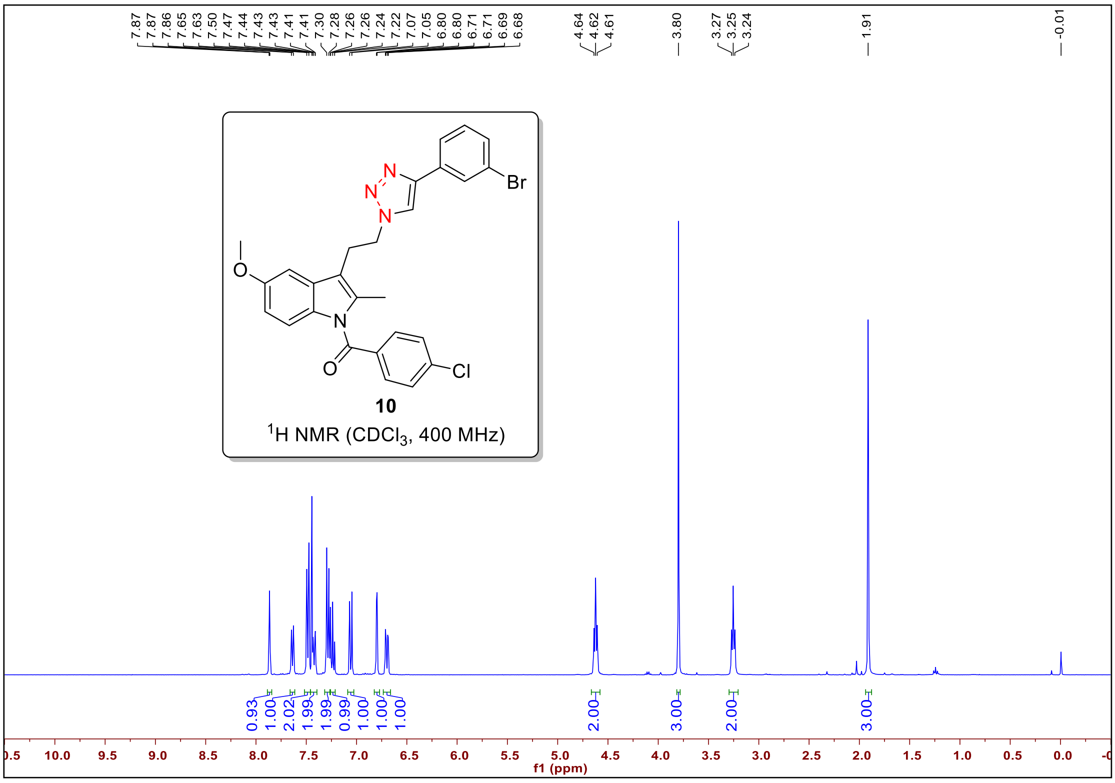

Supplementary Figure 127. ^{1}H NMR Spectrum of 10

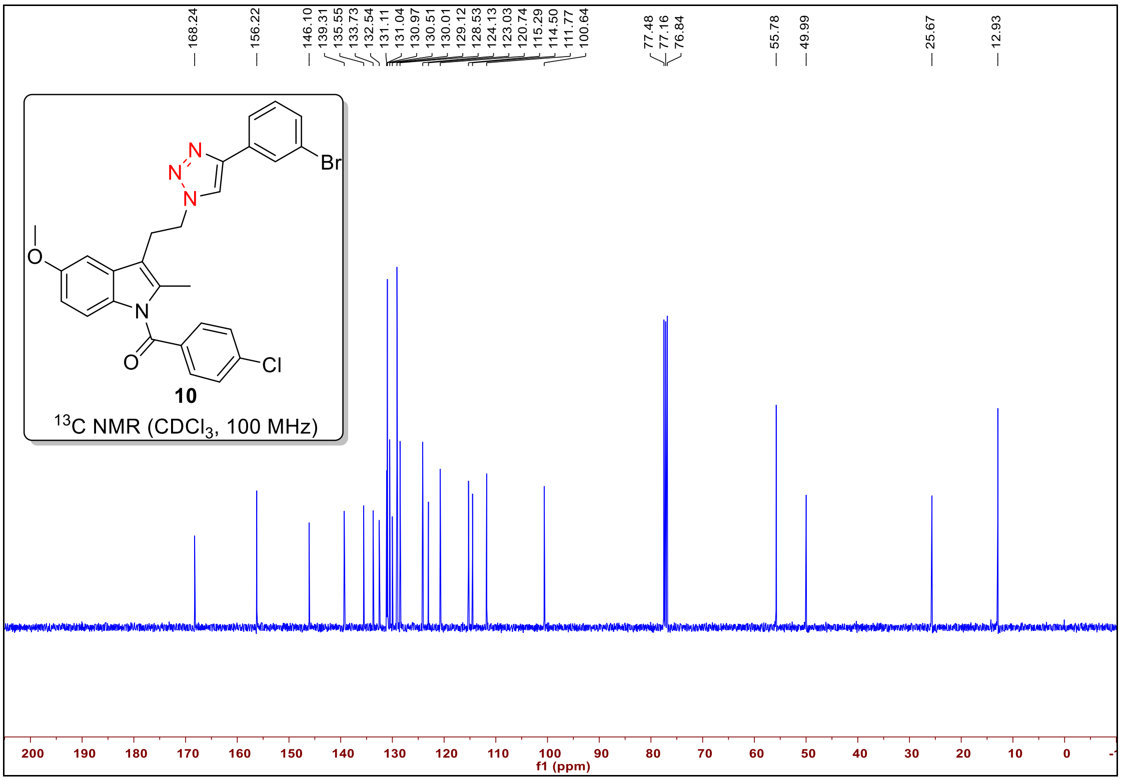

Supplementary Figure 128. ^{13}C NMR Spectrum of 10

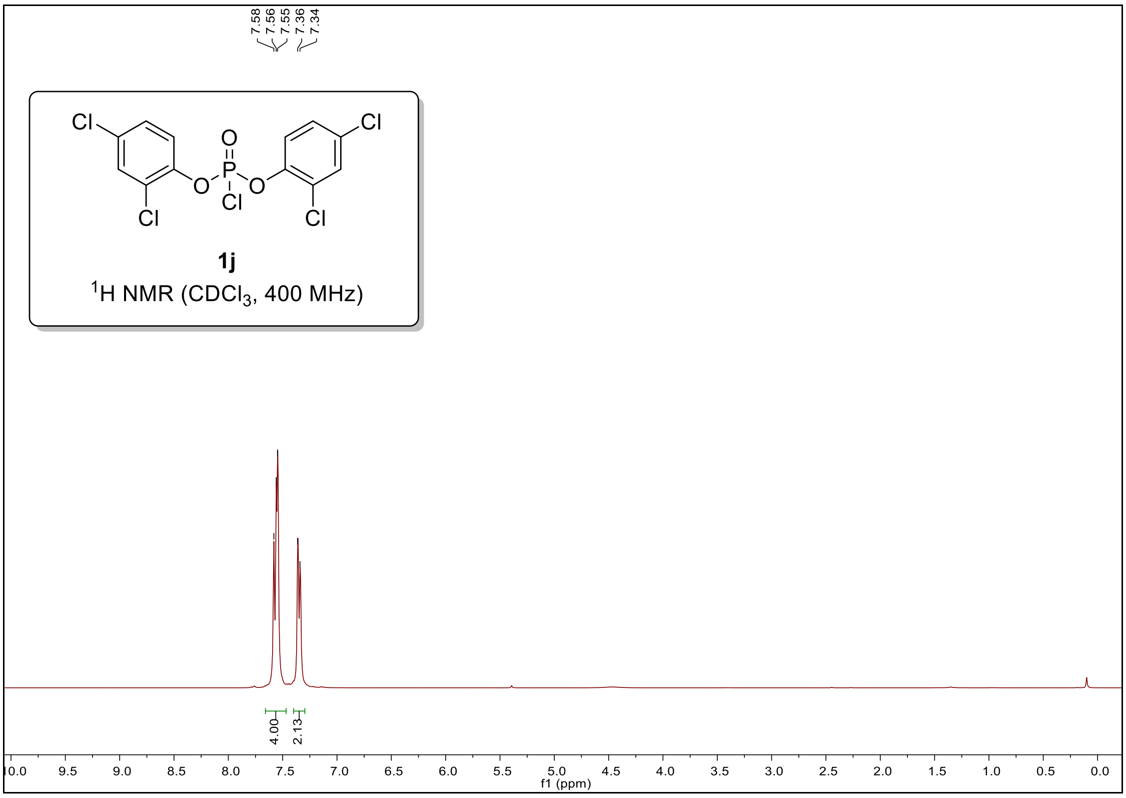

Supplementary Figure 129. ^{1}H NMR Spectrum of 1j

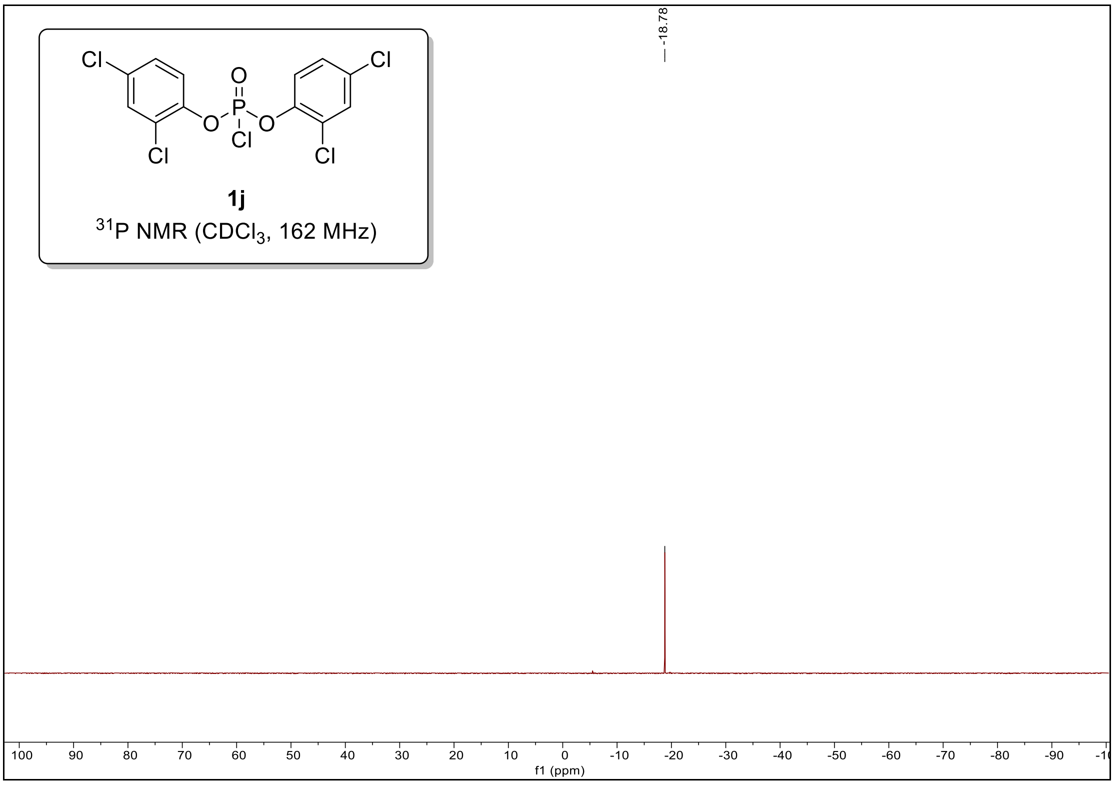

Supplementary Figure 130. ^{31}P NMR Spectrum of 1j

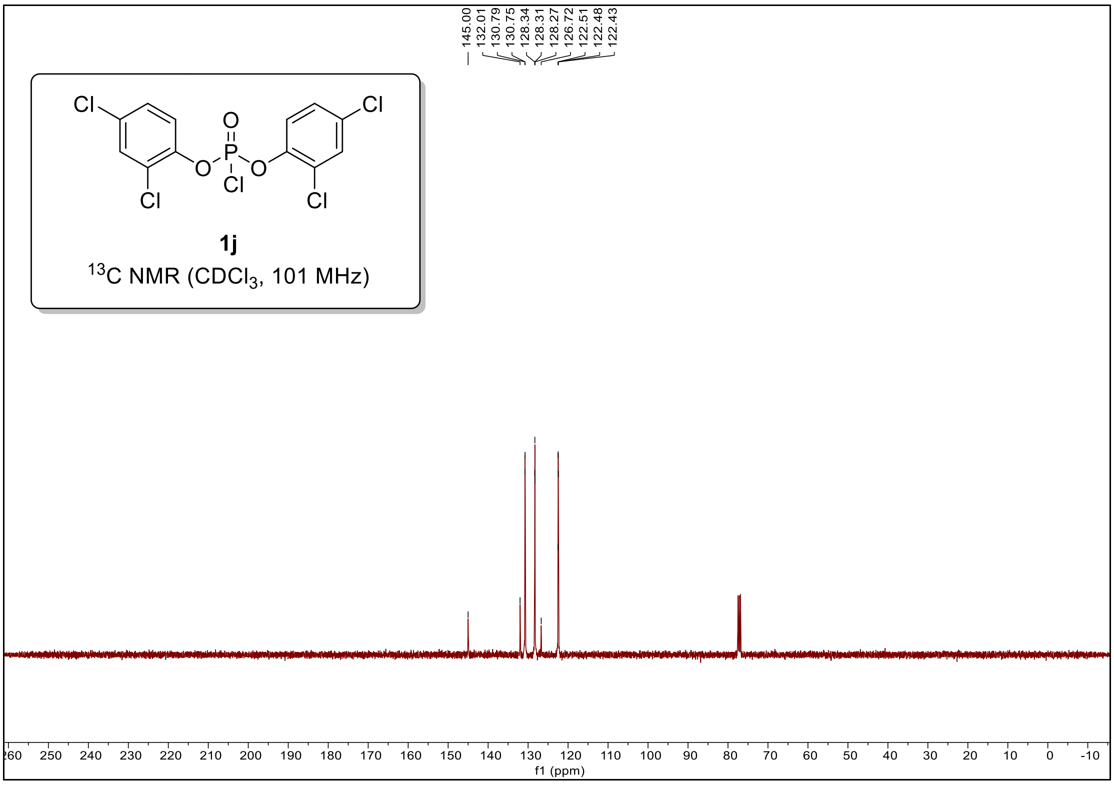

Supplementary Figure 131. ^{13}C NMR Spectrum of 1j

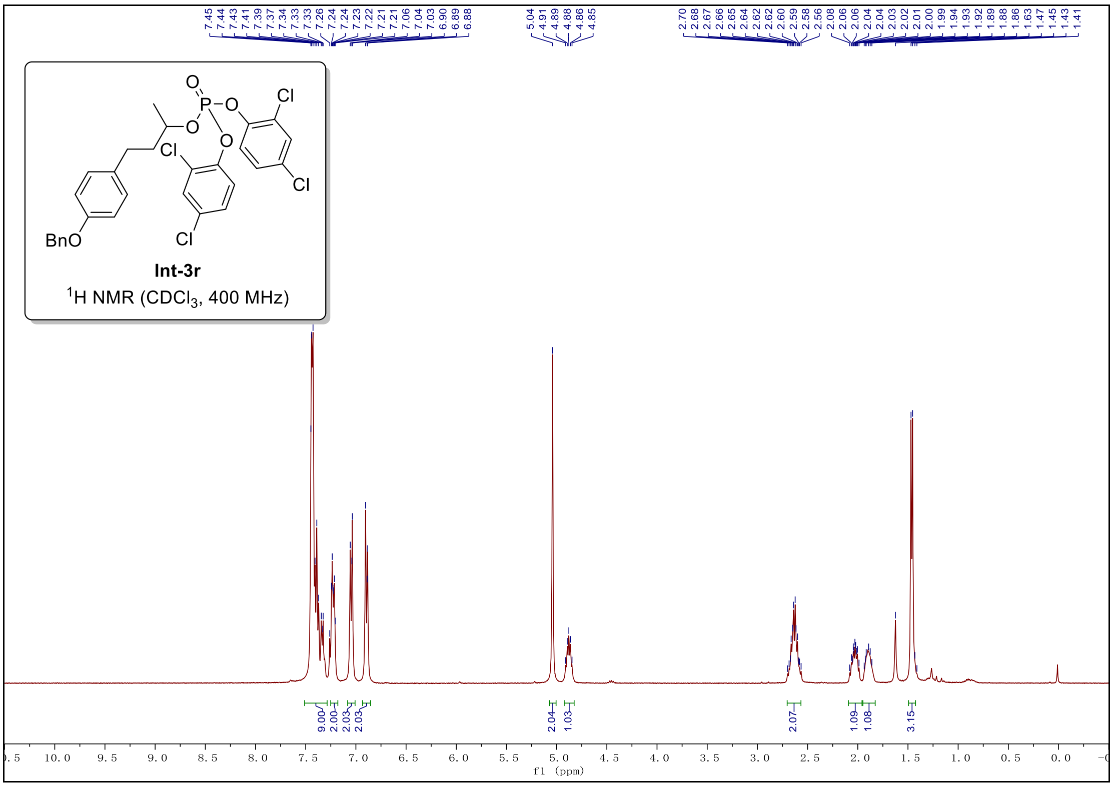

Supplementary Figure 132. ^{1}H NMR Spectrum of Int-3r

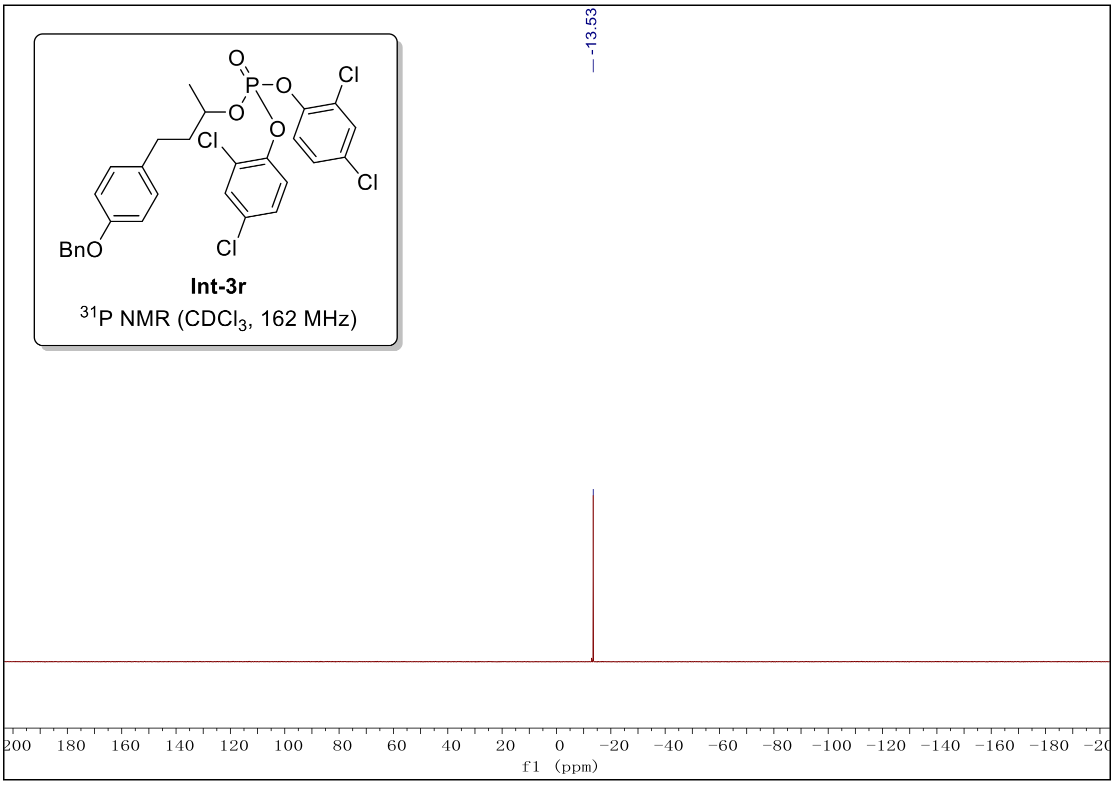

Supplementary Figure 133. ^{31}**P NMR Spectrum of Int-3r**

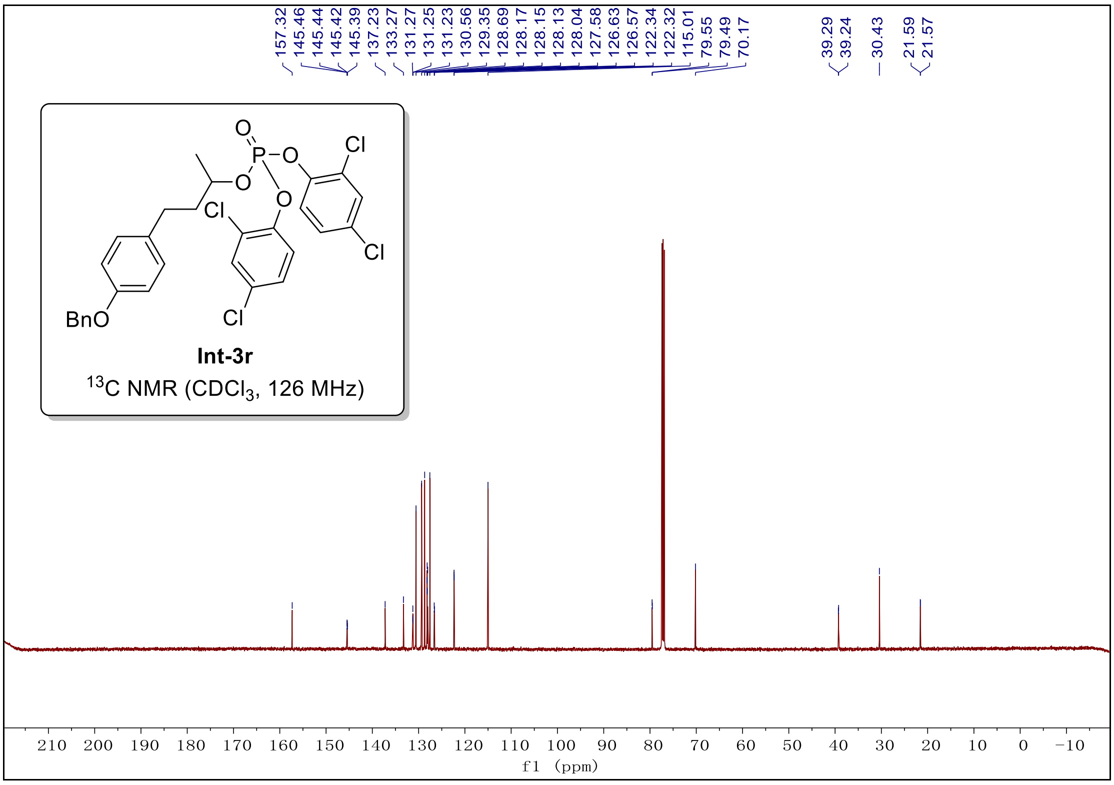

Supplementary Figure 134. ^{13}**C NMR Spectrum of Int-3r**

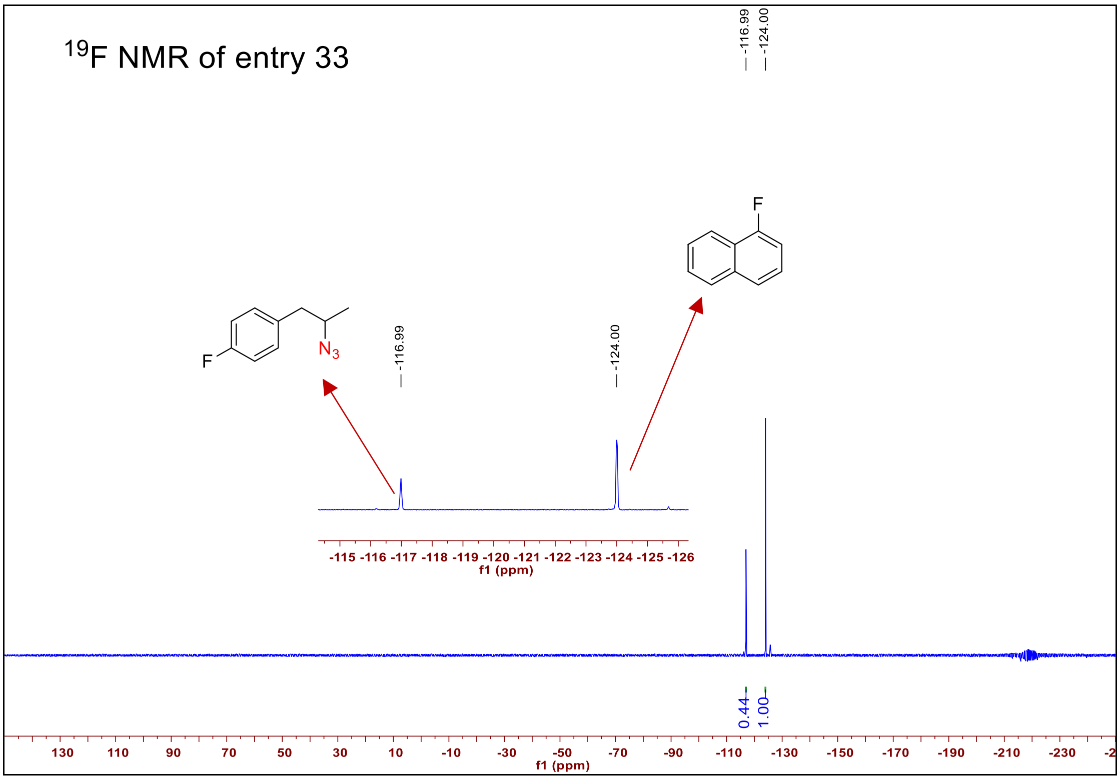

Supplementary Figure 135. ^{19}F **NMR Spectrum of Entry 33 in Supplementary Table 2**

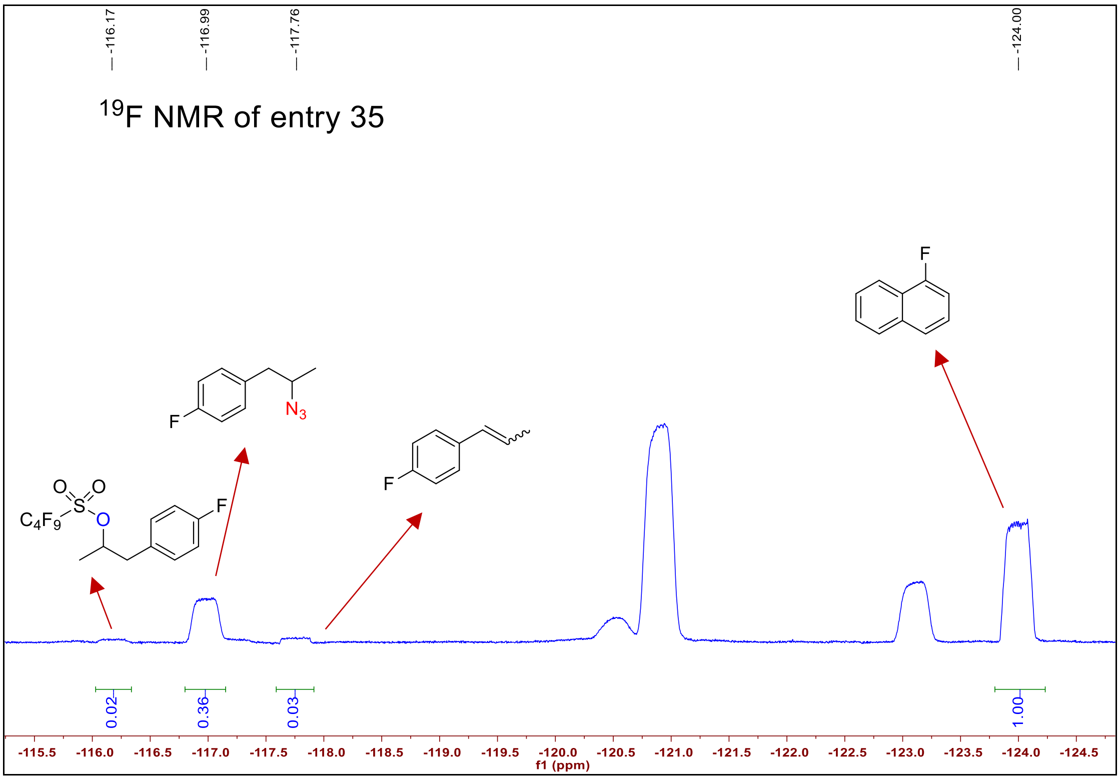

Supplementary Figure 136. ^{19}F **NMR Spectrum of Entry 35 in Supplementary Table 2**

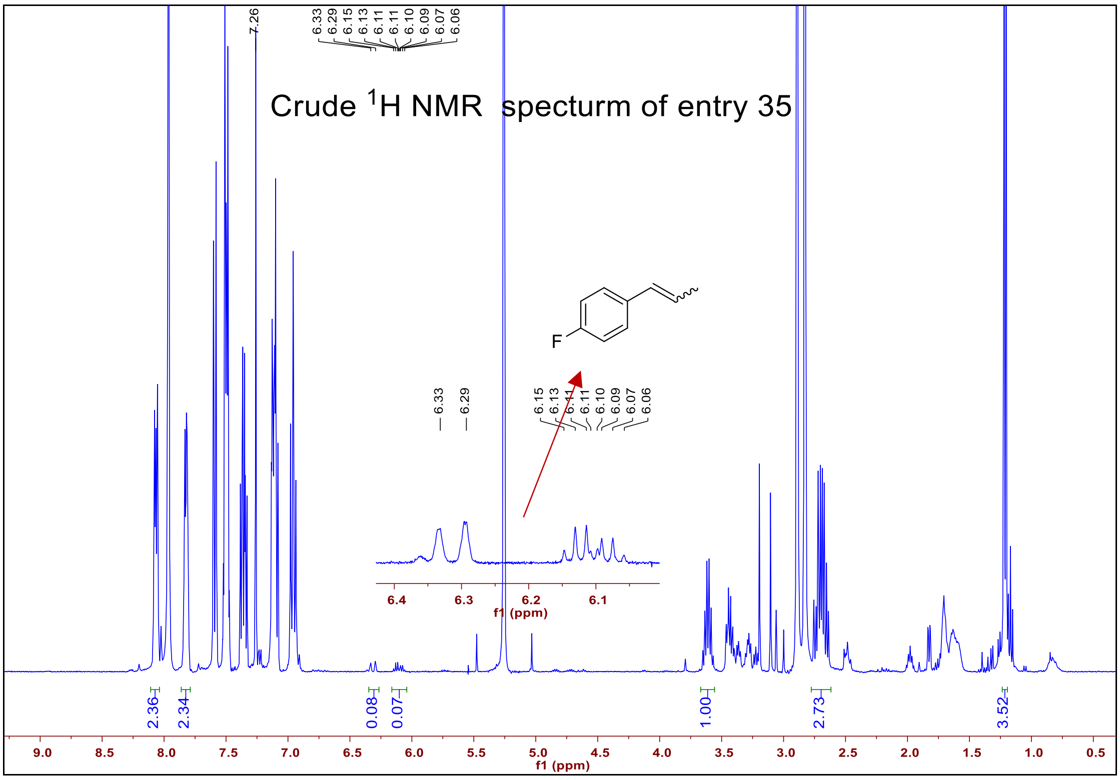

Supplementary Figure 137. Crude ^{1}H NMR Spectrum of Entry 35 in Supplementary Table 2

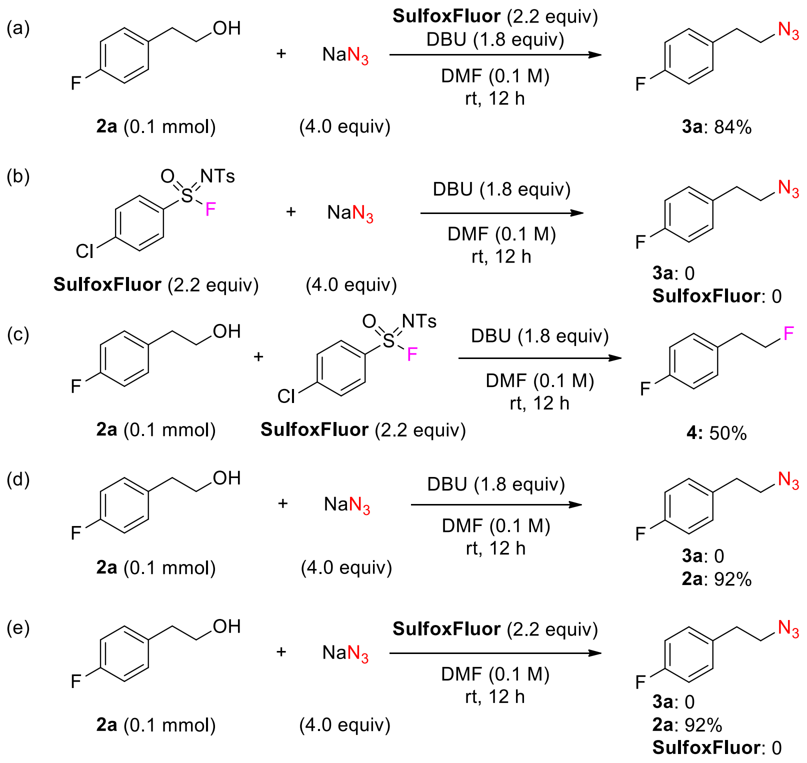

Supplementary Figure 138. Probing the Role of SulfoxFluor and DBU

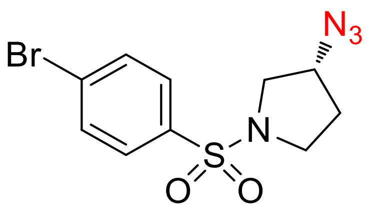

3v

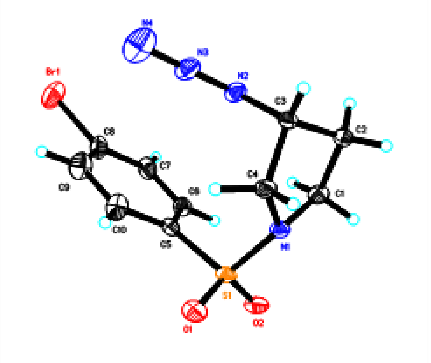

Supplementary Figure 139. ORTEP Image of the X-ray Crystal Structure of 3v (with thermal ellipsoids at the 30% probability level)

The crystallographic coordinates have been deposited with the Cambridge Crystallographic Data Centre; deposition No.: CCDC 2005774. These data can be obtained free of charge via from the Cambridge Crystallographic Data Centre, 12 Union Road, Cambridge CB2 1EZ, UK;fax: (+44)1223-336-033; via www.ccdc.cam.ac.uk/conts/retrieving.html or deposit@ccdc.cam.ac.uk. For crystal data and details on structure refinement for **3r**, see Supplementary Table 6.

Supplementary Tables

Supplementary Table 1. List of Non-commercial Reagents

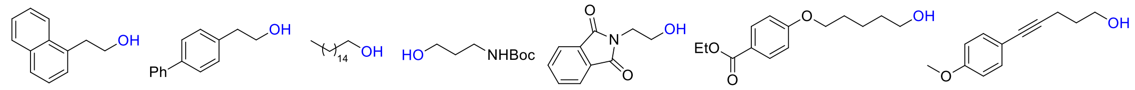

2d 2e 2f 2g 2h 2i 2j

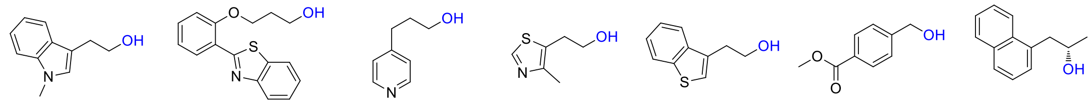

2k 2l 2m 2n 2o 2p 2q

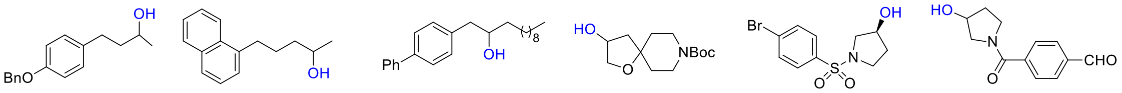

2r 2s 2t 2u 2v 2w

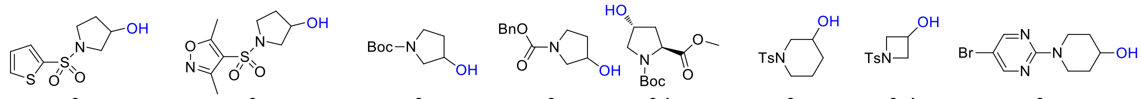

2x 2y 2z 2aa 2ab 2ac 2ad 2ae

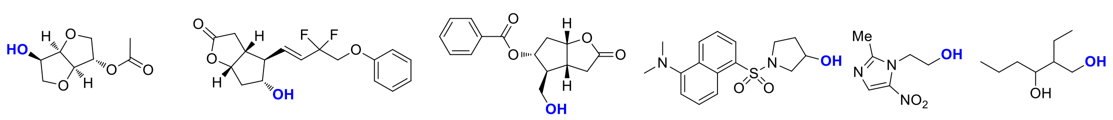

2af 2ag 2ah 2ai 2aj 2ak

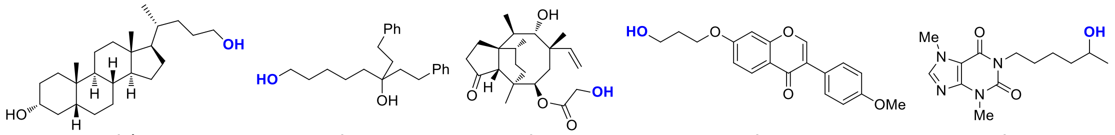

2al 2am 2an 2ao 2ap

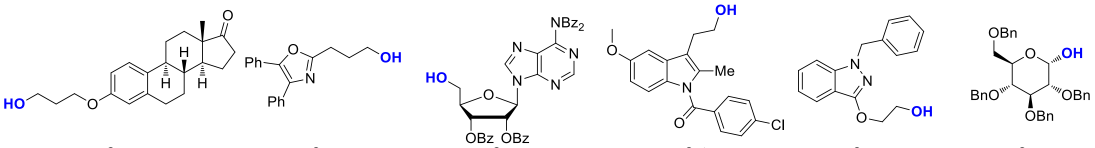

2aq 2ar 2as 2at 2au 2av

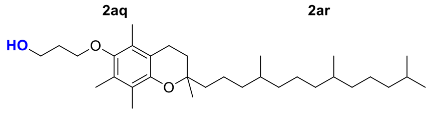

2aw

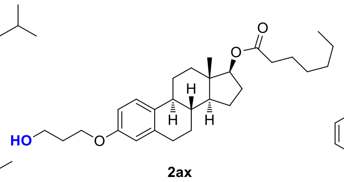

2ax

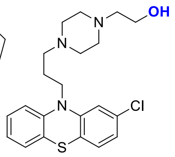

2ay

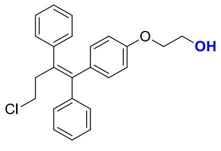

2az

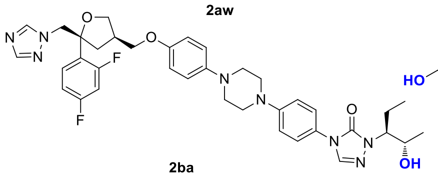

2ba

Supplementary Table 2. Deoxyazidation of Primary Alcohols[a]

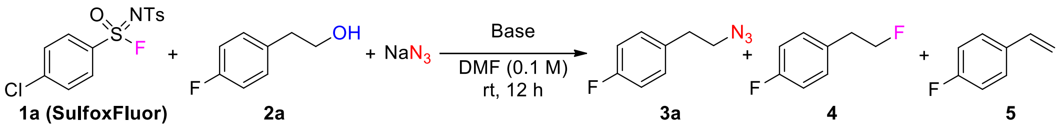

Entry	Base	**SulfoxFluor**/**2a**/NaN$_3$/base	**2a** (%)	**3a** (%)	**4** (%)	**5** (%)
1	DBU	1.0 : 1.0 : 1.0 : 1.0	31	59	2	0
2	DBU	1.0 : 1.3 : 4.0 : 1.8	17	66	trace	0
3	DBU	1.0 : 1.5 : 4.0 : 1.8	10	70	0	0
4	DBU	1.0 : 1.8 : 4.0 : 1.8	8	79	0	0
5	DBU	1.0 : 2.0 : 4.0 : 1.8	4	81	0	0
**6**	DBU	**1.0 : 2.2 : 4.0 : 1.8**	**2**	**84**	**0**	**0**
7[b]	DBU	1.0 : 2.2 : 4.0 : 1.8	4	82	2	0
8	DBU	1.0 : 2.5 : 4.0 : 1.8	2	82	2	0
9	DBU	1.0 : 3.0 : 4.0 : 1.8	0	63	15	0
10	DBU	1.0 : 2.2 : 2.0 : 1.8	trace	69	10	0
11	DBU	1.0 : 2.2 : 3.0 : 1.8	trace	75	8	0
12	DBU	1.0 : 2.2 : 4.0 : 1.3	10	79	0	0
13	DBU	1.0 : 2.2 : 4.0 : 1.5	8	79	0	0
14	DBU	1.0 : 2.2 : 4.0 : 2.0	2	81	0	0
15	NEt$_3$	1.0 : 2.2 : 4.0 : 1.8	92	0	0	0
16	Pyridine	1.0 : 2.2 : 4.0 : 1.8	90	0	0	0

[a]Reactions were conducted on 0.1 mmol under N_2 atmosphere. Yields were determined by ^{19}F NMR using 1-fluoronaphthalene as internal standard. [b]Reactions were conducted under air atmosphere.

Supplementary Table 3. Deoxyazidation of Secondary Alcohols[a]

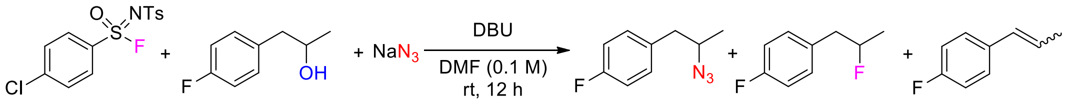

1a (SulfoxFluor) **2b** **3b** **6** **7**

Entry	**SulfoxFluor**/**2b**/NaN_3/ DBU	**2b** (%)	**3b** (%)	**5** (%)	**6** (%)
1	1.0 : 1.3 : 4.0 : 1.8	59	27	0	0
2	1.0 : 2.2 : 4.0 : 1.8	44	38	0	0
3	1.0 : 2.2 : 4.0 : 1.8 (24 h)	44	40	0	0
4	1.0 : 2.5 : 4.0 : 1.8	40	35	2	0
5	1.0 : 2.2 : 4.0 : 1.0	56	27	0	0
6	1.0 : 2.2 : 4.0 : 1.5	59	29	0	0
7	1.0 : 2.2 : 4.0 : 2.0	44	42	0	0
8	1.0 : 2.2 : 4.0 : 2.5	33	50	0	0
9	1.0 : 2.2 : 4.0 : 3.0	36	46	0	0
10	1.0 : 2.2 : 4.0 : 3.5	27	58	0	0
11	1.0 : 2.2 : 4.0 : 4.0	25	59	0	0
12	1.0 : 2.2 : 4.0 : 5.0	17	61	0	0
13	1.0 : 2.2 : 4.0 : 6.0	23	63	0	0
14	1.0 : 2.2 : 5.0 : 5.0	19	65	0	0
15	1.0 : 2.5 : 4.0 : 4.0	21	61	0	0
16	1.0 : 2.8 : 4.0 : 4.0	17	65	0	0
17	1.0 : 2.8 : 5.0 : 5.0	23	65	0	0
18[b]	1.0 : 2.8 : 2.5×2 : 2.5×2	17	65	0	0
19	1.0 : 2.2 : 4.0 : 4.0 ($n$$Bu_4NCl$)[c]	21	61	0	0
20	1.0 : 2.2 : 4.0 : 4.0 ($n$$Bu_4NBr$)[c]	19	65	0	0
21	1.0 : 2.5 : 4.0 : 4.0 ($n$$Bu_4NI$)[c]	23	58	0	0
22	1.0 : 2.2 : 4.0 : 4.0 ($n$$Bu_4NClO_4$)[c]	21	63	0	0
23	1.0 : 2.8 : 4.0 : 5.0	21	61	0	0
24	1.0 : 2.2 : 6.0 : 4.0 ($n$$Bu_4NBr$)[c]	23	63	0	0
25	1.0 : 2.8 : 6.0 : 4.0	31	56	0	0
26	1.0 : 2.8 : 5.0 : 4.0	27	58	0	0
27	1.0 : 2.8 : 4.0 : 4.0	17	65	0	0
28	1.0 : 2.8 : 3.8 : 4.0	21	65	0	0
29	1.0 : 2.8 : 3.5 : 4.0	19	67	0	0
30	1.0 : 2.8 : 3.0 : 4.0	15	73	0	0
31	1.0 : 2.8 : 2.8 : 4.0	8	81	0	0
32	1.0 : 2.8 : 2.5 : 4.0	2	82	0	0
33	**1.0 : 2.8 : 2.0 : 4.0**	**0**	**84**	**0**	**0**
34[d]	1.0 : 2.8 : 2.0 : 4.0	0	82	0	0
35	**1.0 : 2.8[e] : 2.0 : 4.0**	**0**	**68**	**0**	**7**
36	1.0 : 2.8 : 1.0 : 4.0	0	81	0	0
37	1.0 : 2.8 : 2.5 : 2.0	13	73	0	0
38	1.0 : 2.2 : 2.0 : 2.0	17	67	0	0

[a]Reactions were conducted on 0.1 mmol under N_2 atmosphere. Yields were determined by ^{19}F NMR using 1-fluoronaphthalene as internal standard. [b]DBU and NaN_3 were added in batches. [c]In parentheses: adding 2 mol% $n$$Bu_4NX$. [d]Reaction time: 6 h. The ester intermediate was detected by ^{19}F NMR in 2% yield. [e]**PBSF** was used instead of **SulfoxFluor**. Elimination side-product was detected in 7% yield. Average of three runs.

Supplementary Table 4. Optimization of the reaction conditions: Screening the solvents.[a]

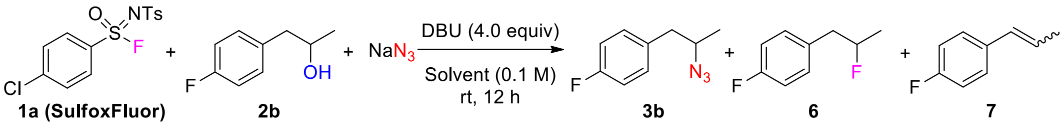

1a (SulfoxFluor) **2b** **3b** **6** **7**

Entry	Slovent	**2b** (%)	**3b** (%)	**5** (%)	**6** (%)	Sulfonimidoyl ester **12** (%)
1	DMF	0	84	0	0	0
2	DMSO	0	82	0	0	2
3	toluene	2	8	42	0	15
4	CH_3CN	2	17	21	0	31

[a]Reactions were conducted on 0.1-mmol scale using 2.8 equiv of **SulfoxFluor**, 4.0 equiv of DBU and 2.0 equiv of NaN_3. Yields were determined by ^{19}F NMR using 1-fluoronaphthalene as an internal standard.

Supplementary Table 5. Comparison of Various Sulfonyl Fluorides and Sulfoximidoyl Fluorides in Deoxyazidation of Alcohols

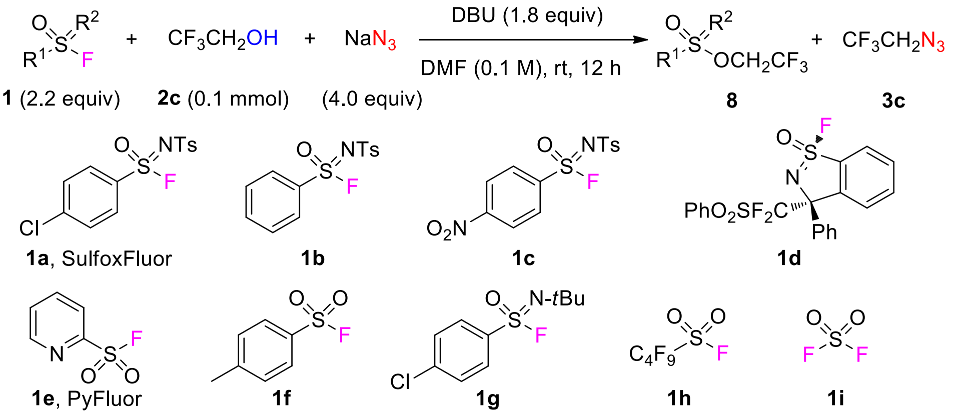

Entry	**1**	**2c** (%)	**8** (%)	**3c** (%)
1	**1a**	4	0	93
2	**1b**	4	12	73
3	**1c**	16	trace	67
4	**1d**	53	41	0
5	**1e**	0	99[b]	trace
6	**1f**	0	>99[b]	0
7	**1g**	82	trace	0
8	**1h**	2	0	82
9	**1i**	0	0	12

[a]Reactions were conducted on 0.1 mmol under N_2 atmosphere. Yields were determined by ^{19}F NMR using $PhCF_3$ as internal standard. [b]The existence of **B5** and **B6** were proved by GC-MS. [c]SO_2F_2 was dissolved in DMF at a concentration of 0.0616 M.

Supplementary Table 6. Crystal data and structure refinement for **3r**

Empirical formula	$C_{10}H_{11}BrN_4O_2S$	
Formula weight	331.20	
Temperature	193(2) K	
Wavelength	0.71073 Å	
Crystal system	Monoclinic	
Space group	P 21	
Unit cell dimensions	a = 7.4964(2) Å	α= 90°.
	b = 10.7218(4) Å	β= 95.8380(10)°.
	c = 16.1038(5) Å	γ = 90°.
Volume	1287.63(7) $Å^3$	
Z	4	
Density (calculated)	1.708 Mg/m^3	
Absorption coefficient	3.354 mm^{-1}	
F(000)	664	
Crystal size	0.170 x 0.140 x 0.100 mm^3	
Theta range for data collection	2.543 to 25.999°.	
Index ranges	-9<=h<=9, -13<=k<=13, -19<=l<=19	
Reflections collected	14784	
Independent reflections	4966 [R(int) = 0.0423]	
Completeness to theta = 25.242°	99.5 %	
Absorption correction	Semi-empirical from equivalents	
Max. and min. transmission	0.7456 and 0.4158	
Refinement method	Full-matrix least-squares on F^2	
Data / restraints / parameters	4966 / 1 / 325	
Goodness-of-fit on F^2	1.032	
Final R indices [I>2sigma(I)]	R1 = 0.0318, wR2 = 0.0660	
R indices (all data)	R1 = 0.0380, wR2 = 0.0682	
Absolute structure parameter	0.057(6)	
Extinction coefficient	n/a	
Largest diff. peak and hole	0.556 and -0.324 e.$Å^{-3}$	

Supplementary References

[1] Feuerstein, M., Doucet, H. & Santelli, M. Coupling reactions of aryl bromides with 1-alkynols catalysed by a tetraphosphine/palladium catalyst. *Tetrahedron Lett.* **45**, 1603–1606 (2004).

[2] Lozano, O., Blessley, G., Martinez del Campo, T., Thompson, A. L., Giuffredi, G. T., Bettati, M., Walker, M., Borman, R. & Gouverneur, V. Organocatalyzed Enantioselective Fluorocyclizations. *Angew. Chem. Int. Ed.* **50**, 8105–8109 (2011).

[3] Kruegel, A. C., Rakshit, S., Li, X. & Sames, D. Constructing *Iboga* Alkaloids via C–H Bond Functionalization: Examination of the Direct and Catalytic Union of Heteroarenes and Isoquinuclidine Alkenes. *J. Org. Chem.* **80**, 2062–2071 (2015).

[4] Inui, H. & Murata, S. Photochemistry of 2-(1-Naphthyl)-2H-azirines in Matrixes and in Solutions: Wavelength-Dependent C−C and C−N Bond Cleavage of the Azirine Ring. *J. Am. Chem. Soc.* **127**, 2628–2636 (2005).

[5] Bracher, F. & Litz, T. 2-Aryl-2-[1-(2-hydroxypropyl)]-1,3-dithianes as Versatile Building Blocks for the Preparation of Enantiomerically Pure Drugs. *Arch. Pharm.* **328**, 235–238 (1995).

[6] Li, L., Ni, C., Wang, F. & Hu, J. Deoxyfluorination of alcohols with 3,3-difluoro-1,2-diarylcyclopropenes. *Nat. Commun.* **7**, 13320 (2016).

[7] Chang, M.-Y., Wang, S.-Y. & Pai, C.-L. New synthesis of SKF 89976A. *Tetrahedron Lett.* **47**, 6389–6392 (2006).

[8] Ishida, N., Shimamoto, Y., Yano, T. & Murakami, M. 1,5-Rhodium Shift in Rearrangement of *N*-Arenesulfonylazetidin-3-ols into Benzosultams. *J. Am. Chem. Soc.* **135**, 19103–19106 (2013).

[9] Guo, J.-K., Kuang, C.-W., Rong, J., Li, L.-C., Ni, C.-F. & Hu, J.-B. Rapid Deoxyfluorination of alcohols with *N*-tosyl-4-chlorobenzenesulfonimidoyl fluoride (SulfoxFluor) at room temperature. *Chem. Eur. J.* **25**, 7259-7264 (2019).

[10]. Nielsen, M. K., Ahneman, D. T., Riera, O. & Doyle, A. G. J. Deoxyfluorination with Sulfonyl Fluorides: Navigating Reaction Space with Machine Learning. *Am. Chem. Soc.* **140**, 5004–5008 (2018).

[11] Debarge, S., Balzarini, J. & Maguire, A. R. Design and Synthesis of α-Carboxy Phosphononucleosides. *J. Org. Chem.* **76**, 105–126 (2011).

[12] Wey, S.-J., Augustyniak, M. E., Cochran, E. D., Ellis, J. L., Fang, X., Garvey, D. S., Janero, D. R., Letts, L. G., Martino, A. M., Melim, T. L., Murty, M. G., Richardson, S. K., Schroeder, J. D., Selig, W. M., Trocha, A. M., Wexler, R. S., Young, D. V., Zemsteva, I. S. & Zifcak, B. M. Structure-Based Design, Synthesis, and Biological Evaluation of Indomethacin Derivatives as Cyclooxygenase-2 Inhibiting Nitric Oxide Donors. *J. Med. Chem.* **50**, 6367–6382 (2007).

[13] Liu, R., Zhou, X.-C., He, X.-Y., Li, Y.-Q., Zheng, W., Wang, X, Guo, J., Ni, C. & Hu, J. Modified and Scalable Synthesis of *N*-tosyl-4-chlorobenzenesulfonimidoyl fluoride (SulfoxFluor): direct Imidation of sulfinyl chlorides with chloramine-T trihydrate. *Org. Process Res. Dev.* **26**, https://pubs.acs.org/doi/10.1021/acs.oprd.1c00431 (2022).

[14] Muthyala, M. K., Choudhary, S., Pandey, K., Shelke, G. M., Jha, M., & Kumar, A. Synthesis of Ionic-Liquid-Supported Diaryliodonium Salts. *Eur. J. Org. Chem.* **2014**, 2365–2370 (2014).

[15] Jadhav, V. H., Kim, J.-Y., Chi, D. Y., Lee, S. & Kim, D. W. Organocatalysis of nucleophilic substitution reactions by the combined effects of two promoters fused in a molecule: oligoethylene glycol substituted imidazolium salts. *Tetrahedron* **70**, 533–542 (2014).

[16] King, J. F., Loosmore, S. M. & Aslam, M. Betylates. 3. Preparative nucleophilic substitution by way of [2]-, [3]-, and [4]betylates. Stoichiometric phase transfer and substrate-reagent ion-pair (SRIP) reactions of betylates. *J. Am. Chem. Soc.* **104**, 7108–7122 (1982).

[17] Zhang, H., Tanimoto, H., Morimoto, T., Nishiyama, Y. & Kakiuchi, K. Regioselective Rapid Synthesis of Fully Substituted 1,2,3-Triazoles Mediated by Propargyl Cations. *Org. Lett.*, **15**, 5222–5225 (2013).

[18] Pyta, K., Klich, K., Domagalska, J. & Przybylski, P. Structure and evaluation of antibacterial and antitubercular properties of new basic and heterocyclic 3-formylrifamycin SV derivatives obtained via 'click chemistry' approach. *Eur. J. Med. Chem.* **84**, 651–676 (2014).

[19] Campi, E. M., Chong, J. M., Jackson, W. R. & Schoot, M. V. D. Synthesis of substituted (α-methylene lactams by rhodium catalysed carbonylation of acetylenic amines. *Tetrahedron* **50**, 2533–2542 (1994).

[20]. Washington, A. Z., Tapadar, S., George, A. & Oyelere, A. K. Exploiting translational stalling peptides in an effort to extend azithromycin interaction within the prokaryotic ribosome nascent peptide exit tunnel. *Bioorg. Med. Chem.* **23**, 5198–5209 (2015).

[21] Islam, I., Ng, K. Y., Chong, K. T., McQuade, T. J., Hui, J. O., Wilkinson, K. F., Rush, B. D., Ruwart, M. J., Borchardt, R. T. & Fisher, J. F. Evaluation of a vitamin-cloaking strategy for oligopeptide therapeutics: biotinylated HIV-1 protease inhibitors. *J. Med. Chem.* **37**, 293–304 (1994).

[22] Behera, H., Ramkumar, V. & Madhavan, N. Cation-Transporting Peptides: Scaffolds for Functionalized Pores? *Chem. Eur. J.* **21***,* 10179–10184 (2015).

[23] Jung, S. H., Choi, K., Pae, A. N., Lee, J. K., Choo, H., Keum, G., Cho, Y. S. & Min, S.-J. Facile diverted synthesis of pyrrolidinyl triazoles using organotrifluoroborate: discovery of potential mPTP blockers. *Org. Biomol. Chem.*, **12**, 9674–9682 (2014).

[24] Marusawa, H., Setoi, H., Sawada, A., Kuroda, A., Seki, J., Motoyama, Y. & Tanaka, H. Synthesis and Biological Activity of 1-Phenylsulfonyl-4-Phenylsulfonylaminopyrrolidine Derivatives as Thromboxane A_2 Receptor Antagonists. *Bioorg. Med. Chem.* **10**, 1399–1415 (2002).

[25] Hay, M. P., Lee, H. H., Wilson, W. R., Roberts, P. B. & Denny, W. A. Hypoxia-Selective Antitumor Agents. 10. Bis(nitroimidazoles) and Related Bis(nitroheterocycles): Development of Derivatives with Higher Rates of Metabolic Activation under Hypoxia and Improved Aqueous Solubility. *J. Med. Chem.* **38**, 1928–1941 (1995).

[26] Loner, C. M., Luzzio, F. A. & Demuth, D. R. Preparation of azidoaryl- and azidoalkyloxazoles for click chemistry. *Tetrahedron Letters* **53**, 5641–5644 (2012).

[27] Wey, S.-J., Augustyniak, M. E., Cochran, E. D., Ellis, J. L., Fang, X., Garvey, D. S., Janero, D. R., Letts, L. G., Martino, A. M., Melim, T. L., Murty, M. G., Richardson, S. K., Schroeder, J. D., Selig, W. M., Trocha, A. M., Wexler, R. S., Young, D. V., Zemtseva, I. S. & Zifcak, B. M. Structure-Based Design, Synthesis, and Biological Evaluation of

Indomethacin Derivatives as Cyclooxygenase-2 Inhibiting Nitric Oxide Donors. *J. Med. Chem.* **50**, 6367–6382 (2007).

[28] Li, C., Shih, T.-L., Jeong, J. U., Arasappan, A. & Fuchs, P. L. The use of tetramethylguanidinium azide in non-halogenated solvents avoids potential explosion hazards. *Tetrahedron Letters* **35**, 2645–2646 (1994).

[29] Kaur, G., Mahajan, M. P., Pandey, M. K., Singh, P., Ramisetti, S. R. & Sharma, A. K. Design, synthesis and evaluation of Ospemifene analogs as anti-breast cancer agents. *Eur. J. Med. Chem.* **86**, 211–218 (2014).

[30] Kim, H., Park, J., Kim, J. G. & Chang, S. Synthesis of phosphoramidates: a facile approach based on the C–N bond formation via Ir-catalyzed direct C–H amidation. *Org. Lett.* **16**, 5466–5469 (2014).

[31] Nakayama, K. & Thompson, W. J. A highly enantioselective synthesis of phosphate triesters. *J. Am. Chem. Soc.* **112**, 6936–6942 (1990).

[32] Yu, C., Liu, B. & Hu, L. A simple one-pot procedure for the direct conversion of alcohols to azides via phosphate activation. *Org. Lett.* **2**, 1959–1961 (2000).
